# A Random Field Theory of Electromagnetic Information

**DOI:** 10.3390/e28050481

**Published:** 2026-04-22

**Authors:** Said Mikki

**Affiliations:** Zhejiang University-University of Illinois Urbana-Champaign (ZJU-UIUC) Institute, Zhejiang University, Haining 314400, China; mikkisaid@intl.zju.edu.cn or said.m.mikki@gmail.com

**Keywords:** electromagnetic information, random fields, electromagnetic theory, Green’s functions, mutual information, electromagnetic information transmission systems

## Abstract

As a rigorous and comprehensive foundation for electromagnetic information theory (EIT), we develop a general theory that elucidates the universal stochastic structure of radiated electromagnetic (EM) fields and induced currents in generic EM information transmission systems. The framework encompasses arbitrary random scatterers, input information fields, and EM mutual coupling. The system is modeled as a multiply connected, arbitrary Riemannian manifold within the language of differential geometry. Our approach exploits exact Green’s functions (GFs) on manifolds to construct a novel electromagnetic random field theory (EM-RFT). Interpreted as response functions localized on the surfaces of transceivers and scatterers, the GFs allow us to treat the internal physical details of the EM system as a black box, redirecting analytical attention toward external input–output relations in line with signal processing and communication theory. This integration of random fields (RFs), electromagnetics, and GFs yields a unified framework for deriving and characterizing the stochastic structure of arbitrary EM information transmission systems. We rigorously establish that EM random fields satisfying Maxwell’s equations can always be constructed using system GFs driven by external information fields. The theory further decouples stochastic input RFs from random fluctuations associated with the communication medium (e.g., scatterers), and introduces general correlation propagators valid for arbitrary EM links. Using the Karhunen–Loève expansion, all EM random fields are represented as sums of random variables, providing both a simulation framework for arbitrary EM RFs and a basis for evaluating mutual information between input and output spatial domains at arbitrary locations in the system.

## 1. Introduction

### 1.1. Introductory Remarks

The fundamental problem addressed in this article is how to characterize the stochastic structure of a continuous source system emitting spatiotemporal fields. Estimating information in space–time (x,t), with $x\in R^{3}$ and t∈R, is considerably more complex than the original Shannon formulation, which was designed for point-to-point communication settings [1,2]. The physical input to information theory may arise from diverse domains, including electromagnetic (EM) theory (EMT) [3,4], quantum theory [5,6], molecular communications [7], or any other physical process in which momentum or energy can be harnessed to transfer information [8]. In electromagnetic information theory (EIT), the emphasis shifts away from the purely probabilistic approach that dominates conventional information theory, toward the explicit incorporation of deterministic physical degrees of freedom that may impose additional constraints beyond the Shannon limits on information transmission [4,8,9,10,11]. Until recently, most research on EIT has been concentrated at the intersection of information theory and electromagnetics, with significant contributions focusing on Shannon capacity from an EM perspective [3,12,13,14,15,16,17,18,19]. However, even within classical EMT, the development of a physics-informed information theory is highly desirable, not only for its intrinsic theoretical value but also for its potential to inspire innovative approaches to the practical design of communication systems and signal processing. For example, such a conceptual framework has already influenced advancements in capacity-driven design for wireless networks and antenna systems [20,21,22], machine-learning-based analysis and design of EM systems [23,24], EM models of MIMO (multiple-input multiple-output) channels [25], imaging applications [26], electromagnetic compatibility and interference studies [27,28], and the development of reconfigurable metasurfaces [29,30,31,32]. Most of these studies refrain from addressing the fundamental challenges of EIT from the comprehensive and foundational perspective adopted in this work. Consequently, they are best characterized as application-oriented investigations, albeit with some offering significant theoretical insights. In our view, a fully developed mathematical foundation for EIT—one that comprehensively and rigorously integrates probability theory with EM fields to uncover the universal structures underlying EM information transmission systems—remains an open and unmet challenge.

Alternatively, it is natural to examine EIT from the perspective of random field theory (RFT) [33], which treats the EM field as a random function on a differentiable manifold. This perspective intersects with the DoF approach but remains conceptually and computationally distinct. The resulting electromagnetic random field theory (EM-RFT), developed in detail below, combines deterministic electromagnetic principles with the laws of probability and stochastic analysis, establishing a novel and generalized framework for information theory. This synthesis is tailored to applications in physical information processing and communication systems, emphasizing physics-based degrees of freedom and the implicit or explicit constraints these may impose on signal processing and statistical modeling. Generally, it is expected that if a given information storage or transmission system explicitly takes into account the complex and subtle physical structures of the various material and energetic processes involved in its actual operation in real world scenarios, then the information-theoretic framework of the system as such will acquire additional constraints or limits that may help in analyzing and designing information processing schemes. While we agree with this view, we also believe that developing explicit physics-based information theories can also push the original field of information theory toward new directions. A notable example of past fruitful interaction with physics is the well-known development of quantum information theory [6,34].

Recent advances in wireless communications—particularly in massive MIMO, holographic MIMO (hMIMO), and continuous MIMO (cMIMO)—have underscored the need for channel models that faithfully capture the underlying EM physics, including mutual coupling, near-field effects, polarization diversity, and spatial correlation. Standardized models such as those in 3GPP, while computationally efficient, often rely on idealized assumptions (e.g., far-field plane waves, isotropic antennas, scalar coupling, and decoupled element responses) that break down in dense, electrically large, or near-field regimes. In contrast, the EM-RFT framework developed in this paper provides a rigorous, physics-compliant foundation for modeling stochastic information transmission systems under fully general conditions. Our framework treats the entire communication link as a composition of stochastic Green’s functions—namely, the transmitter, scatterer, and receiver antenna current Green’s functions (${\bar{\bar{F}}}_{t},{\bar{\bar{F}}}_{s},{\bar{\bar{F}}}_{r}$) and the medium propagation Green’s functions (${\bar{\bar{G}}}_{t},{\bar{\bar{G}}}_{s}$)^1^—all promoted to the status of second-order random fields. This yields a complete characterization of the received current as a polynomial functional of independent random variables, as shown in Equation (132). Crucially, even when all constituent Green’s functions are Gaussian, the resulting channel response is generally non-Gaussian due to the multiplicative structure of the LoS (4-factor) and NLoS (6-factor) pathways—a fact that invalidates common Gaussianity assumptions in classical information theory but is fully captured by our propagator formalism. This approach subsumes conventional models as special cases. For instance, the 3GPP channel model emerges as a limiting approximation when (i) the system is restricted to co-polarized scalar coupling (eliminating off-diagonal propagators), (ii) mutual coupling is neglected (collapsing the antenna current Green’s functions to identity-like responses), and (iii) the propagation Green’s function is replaced by its far-field asymptotic form. Thus, rather than contradicting existing standards, our framework rigorously embeds them within a broader physical theory, clarifying their domain of validity and quantifying their error when applied outside it. A comprehensive investigation of state-of-the-art communication systems will be pursued in companion papers. The present work focuses on establishing the fundamental mathematical, conceptual, and physical foundations of electromagnetic information theory.

### 1.2. Remarks on Some Previous Works

We now provide brief remarks on some relevant previous work on RFT. The most common method involves the degrees of freedom (DoF) approach, which has been particularly popular in EIT research. This approach is based on a generalization of the sampling theorem from signal processing and information theory, extending it from the time–frequency domain to the spacetime–wavenumber/frequency setting [8]. It is not inherently an RFT approach, and much of the research in this direction appears to avoid addressing the most general scenario involving spatially nonstationary RFTs. For example, the works in [35,36] generalize the sampling theorem to the spatial domain using a plane-wave spectrum representation. While these works reference certain random field models, they employ a scalar Green function and therefore do not constitute a full-wave electromagnetic solution. Additionally, the plane-wave spectrum is truncated to include only propagating modes (far-field approximation), which further limits the physical fidelity of the model. Finally, these approaches rely on spatial stationarity to apply Fourier transform techniques, an assumption that we do not make in our more general formulation below.

To the best of our knowledge, fully fledged RFT formulations of EIT are few. An exception to the relative scarcity of works on information theory for physical fields is the research primarily conducted by Torsten A. Enßlin and collaborators (see the review paper [37] and references therein) on what has come to be known as *information field theory* (IFT). This framework addresses RFs arising in physical applications and seeks to establish a probabilistic bridge between measurement and computation. IFT is built upon two foundational pillars: (1) the utilization of methods from quantum field theory (QFT) and statistical field theory (SFT), and (2) the application of Bayesian probability theory to describe probabilistic inferences and characterize physical fields. This synthesis enables a rigorous and systematic approach to the challenges posed by uncertainty and complexity in physical systems. The central mathematical structure in IFT is the *information Hamiltonian*, which encapsulates a comprehensive description of the system. However, the EM-RFT proposed in this paper diverges in its emphasis on information processing and transmission within a specific, though still broadly applicable, physical context—EM systems. EM systems are fundamentally linear, allowing them to be rigorously characterized using Green’s functions (GFs). Unlike IFT, which adopts a Bayesian framework, our approach relies on the general Kolmogorov formulation of probability theory for random processes and fields [33], thereby maintaining a broader theoretical foundation without imposing prior probabilistic assumptions. Both IFT and EM-RFT emphasize the correlation structure of fields, but their approaches differ significantly. IFT places particular focus on the Gaussian information Hamiltonian (referred to as the *free theory* in that context), while EM-RFT assumes only second-order statistics, accommodating arbitrarily distributed RFs. In IFT, non-Gaussian RFs are addressed through the intricate machinery of interacting QFT within SFT, employing tools such as Feynman diagrams. In contrast, EM-RFT, as a subset of EMT, is fundamentally linear. This linearity simplifies the framework, making it more accessible and practical, particularly for information theorists. Therefore, as our framework is fundamentally independent, we will not draw upon IFT in this paper.^2^

Therefore, investigations of EIT from the perspective of RFT, particularly for applications in communications, remain relatively scarce, despite the evident scope and necessity of EM-RFT in EIT. Notable exceptions include the study in [38], which explores applications to communication systems within the framework of traditional Gaussian RFT. That work focuses on the specific yet significant case of electromagnetic noise RF, employing a single GF to characterize the entire system. While this represents a step in the right direction, the approach is not the most mathematically and physical comprehensive and hence can be further expanded and generalized, as we aim to accomplish in our formulation of EM-RFT where five independent EM GFs are shown to be needed to fully describe a complete stochastic EM information transmission system.

From the perspective of the specific approach adopted in this paper, a comprehensive EM spacetime model for information transmission was developed for single-input–single-output (SISO) deterministic systems using the GF method [39]. This approach decomposes the communication system into three fundamental operational modes: Mode A (radiating current excitation), Mode B (generation of radiated fields), and Mode C (conversion of the received radiation field into the received current signal), with each mode assigned a unique physics-based GF [40,41,42]. While we adopt the above-mentioned response function formalism, our theory presented here is based on the RF model, which was not utilized in [39]. However, due to the complexity of time-domain analysis in EIT, this work employs a frequency-domain approach using Fourier analysis. Thus, only frequency-domain GFs will be deployed, leading to RFs whose main behavior is that of spatial RFs rather than the considerably more complex and less well-understood case of spatiotemporal RFs [43].

All results are derived under the assumption of excitation by a time-harmonic wave of frequency ω, where time-domain waveforms are proportional to exp(−iωt). Additionally, all EM processes are assumed to be linear. Under these conditions, the GF method provides a robust framework for analysis [44,45]. Lists of abbreviations and a description of the mathematical notations used in this paper can be found at the end.

### 1.3. On Random Field Theory and Electromagnetic Information Theory: Motivations and Outline

**Figure 1:**
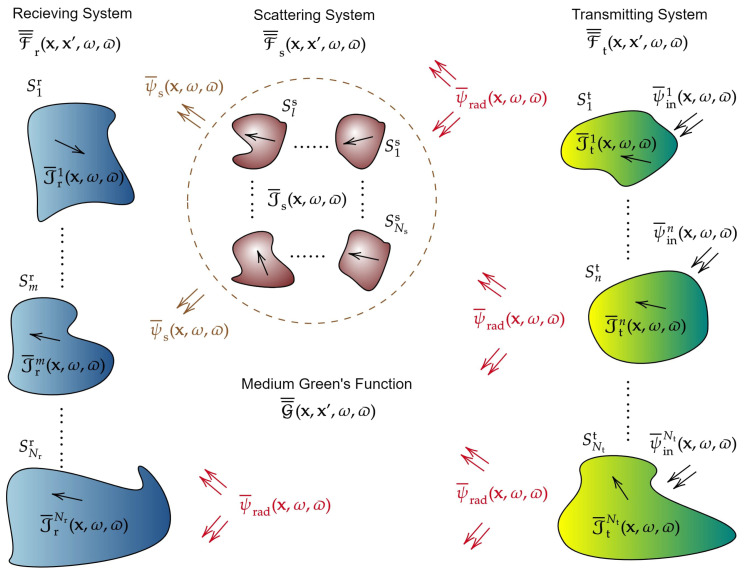
The general formal structure of an EM communication system involving transmitting, scattering, and receiving surfaces $\left(S_{t},S_{s},S_{r}\right)$ is characterized by the respective GFs ${\bar{\bar{F}}}_{t}\left(x,x^{\prime },\omega ,\varpi \right)$, ${\bar{\bar{F}}}_{s}\left(x,x^{\prime },\omega ,\varpi \right)$, ${\bar{\bar{F}}}_{r}\left(x,x^{\prime },\omega ,\varpi \right)$, and the radiation GFs $\bar{\bar{G}}\left(x,x^{\prime },\omega ,\varpi \right)$. For generality, we assume that these GFs may themselves become stochastic RFs [note that in this case the medium GF should be treated as two distinct transmitting and scattering GFs ${\bar{\bar{G}}}_{t}\left(x,x^{\prime },\omega ,\varpi \right)$ and ${\bar{\bar{G}}}_{s}\left(x,x^{\prime },\omega ,\varpi \right)$]. To express the received current RF $\bar{J}\left(x,\omega ,\varpi \right)$ in terms of the input information RF ${\bar{\psi }}_{\mathrm{in}}\left(x,\omega ,\varpi \right)$, all other EM GFs are needed. The expansion of surface fields and currents into individual components ${\bar{\psi }}_{\mathrm{in}}^{n}\left(x,\omega ,\varpi \right)$ and ${\bar{J}}_{b}^{n}\left(x,\omega ,\varpi \right)$ is illustrated by relations such as (A36).

Electromagnetic random fields (EM RFs) are generally neither isotropic nor spatially stationary (homogeneous), primarily due to the asymmetric distribution of scattering clusters, distinctive transmitter/receiver configurations, wireless channel obstacles, and other environmental factors [46]. One can readily see why an EM RF need not exhibit homogeneity, as illustrated in Figure 1. The behavior of EM waves in specific regions, such as those occupied by scatterers, denoted as $V_{s}$, can significantly differ from their behavior in the far-field zone. In fact, near-field (NF) correlation structures are well-known to exhibit distance-dependent characteristics [47,48,49,50,51,52]. The spatial inhomogeneity of EM RFs poses a significant challenge to the development of a general theory of EM RFs within the framework of information theory. This inhomogeneity complicates the application of established mathematical tools such as spectral measures, Fourier transforms, and traditional covariance models. However, this difficulty is mitigated by a considerable simplification in the mathematical proofs of various theorems, since in practical information transmission systems, it is typically sufficient to operate within *compact* subdomains of $R^{3}$. As a result, even without assuming spatial homogeneity, the theory presented here remains tractable and manageable in terms of complexity.

The EM-RFT developed in this work is framed within the general context of second-order RFT [53,54]. While the most prominent example of such a theory is Gaussian random fields [33], we deliberately construct our framework without the assumption of Gaussianity. In fact, we later demonstrate that, in the most general scenario—specifically, in stochastic EM information transmission systems—certain response fields are *not* Gaussian, even when all input random fields are Gaussian (see Corollary 4). This highlights the critical importance of avoiding the restrictive assumption of Gaussianity at the outset.

Our basic model starts with a minimal dataset consisting of mean, covariance and pseudo-covariance functions that can be independently varied without being constrained by joint restrictions imposed by electromagnetic theory and information theory. Following this, we will systematically apply EM-RFT to deduce universal structures governing the RF dynamics, subject to the constraints imposed by Maxwell’s equations and other relevant factors. In particular, all polarization components of the six-component complex RF vector

(1) $$\bar{\psi }\left(x,\omega ,\varpi \right):={\left[\begin{array}{cc} E(x,\omega ,\varpi ) & H(x,\omega ,\varpi ) \end{array}\right]}^{⊤},$$

where $x\in R^{3}$, $\omega \in R^{+}$ is the frequency, and ϖ∈Ω is an outcome where Ω is the space of outcomes or state space (see Appendix A and Appendix B), are intrinsically linked through Maxwell’s equations [44,55,56,57]. This generally implies that different field and current components exhibit correlations. The vector $\bar{\psi }\left(x,\omega ,\varpi \right)$ consists of six complex components, each of which must be represented as a 2×1 subarray of real and imaginary parts, resulting in a 12-component real-valued vector RF

(2) $$\psi :\Omega \times R^{3}\times R^{+}\to R^{12}.$$

As noted earlier, we cannot assume that any two components of this 12-dimensional vector field are uncorrelated, necessitating the introduction of cross-correlation functions for the entire vector. Moreover, not all of these cross-correlation functions are independent, as some can be derived from others via Maxwell’s equations and the EM boundary conditions governing the interactions between the input information and radiation fields. Consequently, when simulating EM RFs, one cannot assume arbitrary cross-correlation or cross-covariance functions [58,59]. Moreover, the presence of EM mutual coupling between components of systems in close proximity introduces an additional, higher level of interaction, contributing further to the coupling and resulting in new correlation phenomena [60,61,62]. These effects must be incorporated into the overall covariance functions used to model EM RFs [63]. Thus, the entire EM RF must be treated as a synchronized whole, evolving in a coordinated manner across both space and time (or equivalently, space and frequency).

Existing computational methods are unable to handle such immense complexity. For instance, one of the most straightforward approaches for simulating an EM information transmission system stochastically is through a variant of the Monte Carlo (MC) simulation method [64]. In this method, samples $\bar{\psi }\left(x,\omega ,\varpi_{i}\right)$ of the RF $\bar{\psi }\left(x,\omega ,\varpi \right)$, with prescribed statistical properties (typically chosen for ease of generation and physical relevance), are generated. For each sample, the deterministic boundary value problem for Maxwell’s equations is then numerically solved.^3^ From a sufficiently *large* set of obtained solutions, one can compute quantities of interest such as mean values, moments, and other statistical measures. However, the primary challenge with this method is the prohibitively large number of computational iterations required to estimate these moments accurately. This arises because solving the full-wave Maxwell’s equations for a transmitter/scatterer/receiver system is computationally intensive, even for a single instance [70].

An alternative approach involves the use of specialized stochastic numerical methods, such as the stochastic Galerkin method [71]. For example, the stochastic finite-element method has been widely applied in fields like civil engineering, mechanical engineering, and materials science [72,73,74,75]. However, the effectiveness of this method is highly dependent on the judicious selection of parameters to ensure convergence [76,77], making it problem-specific and not easily adaptable to a general formulation. In addition, although more efficient than the MC method, the stochastic finite element method remains computationally intensive, particularly when the number of terms in the RF expansion grows large [78,79]. Moreover, as a fully numerical approach, it often obscures the underlying mathematical structure of the problem, especially in contexts relevant to information theory. This complexity arises from the heavy reliance on combining computational linear algebra techniques with random number generators and statistical methods, making it difficult to gain deeper insight into the theoretical aspects of the system.

To address the complexity of directly solving Maxwell’s equations—a system of coupled linear partial differential equations that entails significant mathematical and computational challenges—we propose a streamlined two-stage frequency-domain approach:

- **Stage I**: Estimate key statistical properties, including mean, covariance, and pseudo-covariance functions, derived either from measurement campaigns or through the development and application of specialized computational EM algorithms and methods.
- **Stage II**: Construct, simulate, and analyze EM RFs using the response (Green) function method.

Utilizing the response function theory method offers several advantages. These Green functions (GFs) inherently “know all about EM structures” [80] and can therefore illuminate the interplay and mutual constraints between physical and information-theoretic structures in modern communication systems. The GF method in EM allows us to represent all systems and media facilitating EM information flow as effective input–output system functions, expressed in either space–time or spatial frequency domains [39,44,45,56,81]. The main advantages of using the response (Green’s) function method as the cornerstone of a unified framework integrating RFT and EMT—herein proposed as EM-RFT—are as follows:

1. The GF enables us to articulate the pertinent data structures required from a physical perspective to describe and comprehend how information flows in contemporary communication and signal processing systems.
2. The response function method allows us to rigorously construct all relevant currents and fields in a stochastic EM information transmission system through a systematic approach.
3. Utilizing the EM-RFT developed from the GF perspective, it becomes feasible to derive computational procedures that enable the self-consistent simulation of the entire process of information flow and interactions, wherein Maxwell’s equations are automatically enforced at all times.
4. By employing the Karhunen–Loève (KL) expansion of EM RFs, one can devise computational methods for the future simulation of practical wireless EM communication systems. These methods are anticipated to be of intermediate complexity, bridging the gap between MC methods and stochastic finite element methods (FEM).
5. Building on a comprehensive EM-RFT framework, one can anticipate the design and development of electromagnetically aware, physics-informed, capacity-driven optimization methods for application alongside emerging technologies, such as 6G wireless systems.

Overall, we envision that a comprehensive and complete end-to-end and self-consistent theoretical and computational analysis of generic EM information transmission system may become feasible through the application of our proposed methods. This approach will enable a depiction of the salient features characterizing the interrelations among essential processes, such as mutual coupling, correlation, near-field effects, and capacity-driven optimization of EM and geometric degrees of freedom, among others.

### 1.4. The General Scope of the Present Work

An overall description of the main content of this paper can be found in the summary placed in Section 5.1 toward the end. Here we provide some general remarks on the scope of this paper, which is mainly focused on the foundational theoretical, mathematical, and conceptual aspects of electromagnetic random field theory. For numerical verification and some applications, see [82].

The fundamental question addressed in this paper is as follows:

*Given a set of input mean and correlation data (covariance and pseudo-covariance) of complex EM GFs, how can we optimally construct an EM stochastic model that remains consistent with the laws of physics, particularly Maxwell’s equations and principles of information theory?*

We focus on classical EMT and classical information theory. Extending this framework to quantum EM information theory presents significant challenges, as the concept of GFs in the quantum regime diverges substantially from its classical counterpart. Notably, classical and quantum EM GFs coincide only in specific cases, such as coherent states and EM field operator correlations [83]. Nevertheless, we hope that the classical EIT and EM-RFT developed in this paper may inspire further research toward constructing a quantum analogue.

No fully analytical expressions are feasible in EM-RFT due to various limitations, primarily because EM Green’s functions are not always available in closed form. Furthermore, even when closed-form expressions exist, these Green’s functions often yield response integrals that resist analytical evaluation, except in highly symmetric, specialized cases which are generally of limited relevance to practical applications.^4^ Consequently, the present work focuses on developing broad general theorems that are rigorous and exact, deliberately avoiding specialized examples whose outcomes cannot be easily generalized. Indeed, the primary aim of this paper is to establish universal structures for the EM response RF (and all other relevant RFs) suited for applications in information theory, signal analysis, and processing across engineering, physics, and the statistical sciences.

We formulate our theory to take as input data the following objects: the RF’s (i) mean, (ii) covariance, and (iii) pseudo-covariance functions. These could ideally be obtained through measurement campaigns, specialized EM simulations, or numerical solutions. Note, however, that deriving specific correlation data from Maxwell’s equations or experimental measurements for special structures lies outside the scope of this paper; our primary focus is on constructing the EM response RFs starting from covariance and pseudo-covariance functions.

In addition, it should be noted that while the EM-RFT developed in this work primarily addresses wireless settings, where arbitrary transmitters and receivers communicate within a general random scattering channel, the fundamental principles of the theory could also be extended to waveguide scenarios. In such cases, information transmission would be governed by guided wave modes rather than by open medium radiation. GFs for waveguide structures are indeed available in the literature [84] and could be applied to formulate an EM-RFT framework for these types of EM communication channels. However, waveguide-based communication lies beyond the scope of this paper.

Another key aspect that must be carefully addressed in the frequency-domain EM-RFT presented in this paper is its nature as a complex-valued random field theory. Complex-valued information processing introduces unique subtleties that warrant special attention. Note that the entropy of a complex random vector can decrease only for two reasons: (1) non-Gaussianity, and (2) impropriety [85]. By extending these concepts to complex-valued RFs—a straightforward process as detailed in Appendix H—we conclude that a comprehensive EM-RFT is essential. This theory should precisely address how RFs can be both non-Gaussian and improper, in connection with the EM structure of the problem, to provide a deeper understanding of how EM information emerges, propagates, and is modified in modern communication and information processing systems. Notably, our proposed EM-RFT is second-order but not necessarily Gaussian, and the various EM RFs we work with are only assumed to have finite second-order moments and statistics. Furthermore, we always work with both covariance and pseudo-covariance matrices to highlight the improper case (where the pseudo-covariance matrix does not vanish) and to explicitly connect how EMT constrains the form of these matrices.

On the other hand, the modification of the correlation data structures, specifically the covariance and pseudo-covariance matrices, as RFs traverse the EM information transmission system is comprehensively characterized by the correlation/pseudo-covariance propagators, which we derive and express in the form of trace formulas (Theorem 4). These propagators can be interpreted as higher-order (many-point) GFs for correlation, allowing for the output covariance/pseudo-covariance data to be expressed in terms of the input covariance/pseudo-covariance data. This decoupling of correlation information in the form of an input/output relation is notably nontrivial; however, it is facilitated here through the strategic application of the appropriate generalized EM Green’s (response) functions. These functions encapsulate the complete information regarding the mutual coupling of various polarization degrees of freedom as RFs propagate through the physical structure.

To recap, the primary input to EM-RFT involves three essential types of stochastic data required for any complex second-order theory: the mean, covariance, and pseudo-covariance functions defined over various 2-manifolds and 3-dimensional subdomains. The output of this framework is a comprehensive characterization of EM fields, encompassing radiation, scattering fields, and current distributions. The stochastic GFs serve as an intermediate link between these input and output datasets. On one hand, second-order statistical data are necessary to define a particular EM stochastic GF. However, the structure and form of these response functions are strictly constrained by Maxwell’s equations, allowing the stochastic EM GFs to be viewed, to some extent, as an “output” generated by the EM-RFT approach developed in this paper.

### 1.5. Outline of the Present Work

This paper is structured as follows. Section 2 provides the mathematical details of the basic EM response function model utilized in this paper, starting with the geometric model (Section 2.1), followed by a presentation of the essential building blocks of the response function model (Section 2.2). In Section 2.3, we start deriving the general combined (effective) transfer system functions of generic deterministic information transmission systems using the key building blocks introduced earlier. This section serves as the first foundation upon which the subsequent treatment will be based. In Section 3, we begin the careful development of the main subject of this paper, which is EM-RFT. After some introductory remarks in Section 3.1, we present in Section 3.2 a summary of the basic mathematical signal model developed specifically in this paper (mainly in the appendices) to construct EM RFs. This prepares for the first essential step in the theory, which is the rigorous construction of RFs to represent EM fields, first in deterministic information transmission systems (Section 3.3), then in generic stochastic systems (Section 3.4). As preparation for moving to the next major step in our theory, that of the demonstration of the possibility of a system-theoretic treatment of correlation propagation in EM information systems, Section 3.6 provides some intermediate results. The effective decoupling of input and output correlation/pseudo-correlation data is achieved in Section 3.7. As an immediate application of the theory developed in the paper so far, Section 3.9 provides application of the EM-RFT to MIMO system analysis. The next (and final step) in our theory is provided in Section 4, which outlines the Karhunen–Loève (KL) expansion of EM RFs, forming the backbone for future simulation algorithms of EM information. First, we introduce the key concept of the KL mode of an EM RF, which considerably simplifies the subsequent development. Next, the KL theory of both deterministic and stochastic EM information systems is rigorously derived in Section 4.2 and Section 4.3. While not necessarily tied to the KL theory of EM RFs, we provide general definitions of entropy and mutual information based on EM-RFT in Section 4.4. Finally, in Section 5, we conclude with a summary, remarks, and anticipation of possible future extensions of the present theory.

## 2. The Deterministic Formulation of the Electromagnetic Theory of Information Transmission

### 2.1. The Geometric Model

Let us assume that our transmitting system, the continuous surface $S_{t}\subset R^{3}$, is modeled as a closed (compact with no boundary) smooth 2-manifold. The system $S_{t}$ is excited by an information source comprised of the frequency-domain electric field e(x,ω) and magnetic field h(x,ω), which are ultimately treated as complex-valued vector spatial-frequency RFs tangential to $S_{t}∋x$, where $\omega \in R^{+}$ is the frequency.^5^ The surface $S_{t}$ is endowed with a Riemannian structure with a Riemannian metric $g_{t}\left(x,x^{\prime }\right)$ defined by its multilinear map on the tangent space $TS_{t}\left(x\right)$ of the 2-manifold $S_{t}$ at x, with $g_{t}\left(x\right)\left[{\hat{\alpha }}_{1},{\hat{\alpha }}_{2}\right]:={\hat{\alpha }}_{1}\left(x\right)\cdot {\hat{\alpha }}_{2}\left(x\right)$ [86]. Here, $TS_{t}\left(x\right)$ is the tangent space of $S_{t}$ based at $x\in S_{t}$, and ${\hat{\alpha }}_{1}\left(x\right),{\hat{\alpha }}_{2}\left(x\right)\in TS_{t}\left(x\right)\subset R^{3}$ are two Euclidean unit vectors tangential to $S_{t}$ at x. The local system of tangent vectors ${\hat{\alpha }}_{s}:={\hat{\alpha }}_{s}\left(x\right),s=1,2,$ satisfies the conditions

(3) $${\hat{\alpha }}_{s}\cdot \hat{N}=0,\;\;{\hat{\alpha }}_{s}\cdot {\hat{\alpha }}_{s^{\prime }}=\delta_{ss^{\prime }},\;\;∥{\hat{\alpha }}_{s}∥=1,\;\;\;s,s^{\prime }=1,2,$$

where $\hat{N}:=\hat{N}\left(x\right)$ is the local normal to the surface $S_{t}$ at x while $\delta_{ss^{\prime }}$ is the Kronecker delta function. Note that the dot product operation ‘·’ is the standard Euclidean inner product induced by embedding the surface $S_{t}$ into the ambient space $R^{3}$ [87].

The metric $g_{t}$ is induced by the embedding of $S_{t}$ into $R^{3}$. In what follows, we will not explicitly mention the original Riemannian metric tensor $g_{t}$ itself, preferring to work instead with the inner product form ${\hat{\alpha }}_{1}\left(x\right)\cdot {\hat{\alpha }}_{2}\left(x\right)$, where the position vector x will be systematically emphasized to take into account geometric effects, e.g., position-dependent variations of curvature, shape, orientation, etc. However, note that for the rigorous construction of EM GFs on 2-manifolds like $S_{t}$, one needs to explicitly use the metric $g_{t}$; see [39,41,88].

In addition to the transmitting systems, whose geometrical model, the 2-manifold $S_{t}$, was described above, we also have two additional systems, the scattering and receiving systems, which are geometrically modeled as distinct 2-manifolds $S_{s}$ and $S_{r}$, respectively. These manifolds are endowed with their own Riemannian metric tensors $g_{s}$ and $g_{r}$. For simplicity, however, we denote the local coordinate systems on $S_{s}$ and $S_{r}$ corresponding to (3) using the same notation, without superscripts or subscripts. Thus, in this paper, the system ${\hat{\alpha }}_{i}\left(x\right),i=1,2,$ is defined on $x\in {⋃}_{b}S_{b}$, where b∈{t,s,r}. Physically, $S_{r}$ represents the receive surface or antenna array system deployed to interact with the transmitted fields produced by $S_{t}$. On the other hand, $S_{s}$ provides the geometrical model for all scattering objects in the environment *exterior* to the transmitter and receiver.

Let $V_{t}$, $V_{s}$, and $V_{r}$ be three *open* sets defined as the topological interiors of the regions whose topological boundaries are the closed 2-manifolds $S_{t}$, $S_{s}$, and $S_{r}$, respectively [89,90]. Then the total exterior region is the unbounded set

(4) $$V_{\mathrm{ex}}:=R^{3}\setminus \underset{b\in \{t,s,r\}}{⋃}V_{b}=R^{3}\setminus \left(V_{t}\cup V_{s}\cup V_{r}\right).$$

In this paper, we concentrate on the region $V_{\mathrm{ex}}$, which encompasses the surfaces $S_{t}$, $S_{s}$, and $S_{r}$, as well as the open region exterior to them.

**Remark** **1.** *For future work, it may be necessary to restrict the focus to compact subsets of $V_{\mathrm{ex}}$. To formalize this, we define the EM field region of interest $V_{f}\subset V_{\mathrm{ex}}$ as*

(5) $$V_{f}:=\left\{x\in R^{3}|∥x∥\leq \kappa ,\;x\notin V_{t}\cup V_{s}\cup V_{r}\right\},$$

*where κ>0 is a sufficiently large number such that $V_{b}\subset \left\{∥x∥\leq \kappa \right\}$ for b∈{t,s,r}. In other words, $V_{\mathrm{ex}}$ is the compact region obtained by removing the open set $V_{t}\cup V_{s}\cup V_{r}$ from a closed three-dimensional ball of radius κ. Indeed, we are primarily concerned with the fields radiated by the transmitting and scattering systems $S_{t}$ and $S_{s}$ as they reach the receiving system $S_{r}$. Therefore, we can restrict our focus to the compact set $V_{f}$, particularly in numerical computations.*

Technically, it is possible to apply the surface or volume equivalence theorems from EMT to describe the internal dynamics of any EM system, including the transmitter, scatterers, and receiver, leading to descriptions of how EM fields behave *inside* the regions *V*_t_, *V*_s_, *V*_r_, [91,92,93]. However, the GFs used in this paper are derived for the exterior region only. To describe the dynamics within the interior region, a different set of *current*-based GFs is required, as discussed in [88]. For all practical applications, we are seldom, if ever, concerned with the behavior of the fields inside the transmitters, scatterers, or receivers. The primary focus of the present work is the transmission of information from the surface $S_{t}$ to the surface $S_{r}$ while traversing the scattering system $S_{s}$. This reduction of the entire dynamics to surface currents and exterior regions is made possible by the fundamental surface equivalence theorem in EMT [91,92,93].

In the most general case of interest for applications, the transmitter, scattering, and receiver manifolds can be decomposed as

(6) $$S_{t}=\overset{N_{t}}{\underset{n=1}{⋃}}S_{n}^{t},\;\;S_{s}=\overset{N_{s}}{\underset{n=1}{⋃}}S_{n}^{s},\;\;S_{r}=\overset{N_{r}}{\underset{n=1}{⋃}}S_{n}^{r},$$

where we further impose the conditions

(7) $$\underset{b\in \{t,s,r\}}{⋂}S_{b}=\emptyset ,\;\;\overset{N_{t}}{\underset{n=1}{⋂}}S_{n}^{t}=\emptyset ,\;\;\overset{N_{s}}{\underset{n=1}{⋂}}S_{n}^{s}=\emptyset ,\;\;\overset{N_{r}}{\underset{n=1}{⋂}}S_{n}^{r}=\emptyset .$$

The relations (6) and (7) together imply that the manifolds $S_{t}$, $S_{s}$, and $S_{r}$ are the union of completely separated (non-intersecting) closed 2-submanifolds ${\left\{S_{n}^{t}\right\}}_{n=1}^{N_{t}}$, ${\left\{S_{n}^{s}\right\}}_{n=1}^{N_{s}}$, ${\left\{S_{n}^{r}\right\}}_{n=1}^{N_{r}}$. In other words, the parent manifolds are multiply connected with a finite number of components each, here $N_{t}$, $N_{s}$, and $N_{r}$ subsystems for the transmitter, scattering, and receiving systems, respectively, a representation that is broad enough to cover most manifolds of interest in applications [89]. The decompositions of $S_{t}$ and $S_{r}$ can be taken to correspond to the division of the transmitters and receivers into multiple antenna array systems as in MIMO, SIMO, or MISO, in addition to some nearby passive scattering objects coupled through the near field zone to either the transmitting or receiving arrays [94,95]. On the other hand, the decomposition of $S_{s}$ corresponds to the distribution of multiple passive or active objects intervening between the transmitter and receivers, such as random scatters, buildings, reconfigure intelligent surface (RIS), and so on [8,96,97]. In general, while the manifold $S_{s}$ can be extremely complicated, we continue to adhere to the global condition (7) in which there are no intersection points to avoid complications due to possible lack of regularity (continuity, differentiability, etc.) at such points.

Table 1 provides a comprehensive description of the key geometric spaces utilized in the theoretical framework developed herein for modeling EM fields in communication systems. It is important to note that, within the formalism of differential topology, tangent spaces, tensors, and the corresponding vector and tensor fields are inherently independent of the choice of metric [98,99,100,101,102]. Nonetheless, the precise construction of the surface current GFs applied in this study necessitates the incorporation of a metric and differential *geometry* [103], not topology alone, becomes needed [39,41,88].

### 2.2. Basic Electromagnetic Input–Output Formulation Using Individual Response Functions

We now develop descriptions of the EM response functions of all the individual components of the information transmission system comprised of $S_{t}$, $S_{s}$, and $S_{r}$. Working in the frequency domain, we assume that all fields and currents have the time-harmonic excitation form $\exp\left(-i\omega t\right)$, where ω is the radian frequency. Consequently, all EM currents, fields, and GFs become complex [69,93]. The information source electric and magnetic fields e(x,ω) and h(x,ω) can be expressed as

(8) $$\begin{array}{c} e\left(x,\omega \right)=e^{1}\left(x,\omega \right){\hat{\alpha }}_{1}\left(x\right)+e^{2}\left(x,\omega \right){\hat{\alpha }}_{2}\left(x\right),\\ h\left(x,\omega \right)=h^{1}\left(x,\omega \right){\hat{\alpha }}_{1}\left(x\right)+h^{2}\left(x,\omega \right){\hat{\alpha }}_{2}\left(x\right), \end{array}$$

respectively. Here, the two complex-valued spatiotemporal maps $e^{i}:S_{t}\times R^{+}\to C$ and $h^{i}:S_{t}\times R^{+}\to C$ serve as field excitations along the directions specified by the orthonormal tangential vectors ${\hat{\alpha }}_{i}\left(x\right)$ for i=1,2, where $\omega \in R^{+}$. Since we are only interested in the values the source fields take on the transmitting surface, we assume that $x\in S_{t}$ and that e(x,ω)=h(x,ω)=0 for $x\notin S_{t}$. In other words, we think of e(x,ω) and h(x,ω) as essentially tangential functions on the 2-manifold $S_{t}$ and hence their range is effectively $C^{2}$ when the expansion (8) in terms of the system ${\hat{\alpha }}_{i}\left(x\right),i=1,2$ is utilized.^6^

It should be noted that, strictly speaking, the input information fields e(x,ω) and h(x,ω) are rigorously defined as *restrictions* of the full 3-dimensional external EM fields: $e\left(x,\omega \right):={\left.E^{\mathrm{ex}}\left(x,\omega \right)\right|}_{x\in S_{t}}$ and $h\left(x,\omega \right):={\left.H^{\mathrm{ex}}\left(x,\omega \right)\right|}_{x\in S_{t}}$, where the external fields $E^{\mathrm{ex}}\left(x,\omega \right)$ and $H^{\mathrm{ex}}\left(x,\omega \right)$ are 3-dimensional fields satisfying the frequency–space Maxwell’s equations for all $x\in R^{3}$. However, based on the surface equivalence theorem [91,93] formulation of the current GFs adopted in this paper, only the *tangential* components of the field over the radiating system $S_{t}$ are relevant to the calculations of the radiation fields in the system [88].

Table 2 provides a description of all Cartesian vector and dyadic field quantities essential for a complete formulation of EIT in general communication systems. Information about the mathematical structure of the various superarrays (supervectors and superdyads; see Appendix A) utilized in this paper is summarized in Table 3. These higher-order structures build upon the lower-order geometric (Table 1) and physical (Table 2) structures previously developed. The main complexity arises from the need to conceptualize quantities operating in both the 3-dimensional ambient space $R^{3}$ and the three multiply connected smooth 2-manifolds $S_{t}$, $S_{s}$, and $S_{r}$.

We assume that the input information is carried into the system by a single RF, specifically the RF interacting with the transmitting system, $S_{t}$. The complete set of input RFs is denoted by I, which, for the purposes of this paper, consists of only a single member supervector (see Table 4). That is, for the purpose of full generality and compactness, all source fields interacting with $S_{t}$ are combined into one structure given by the supervector.^7^

(9) $${\bar{\psi }}_{\mathrm{in}}\left(x,\omega \right)={\left[\begin{array}{cc} e(x,\omega ) & h(x,\omega ) \end{array}\right]}^{⊤},$$

where T is the transpose operation. Without loss of generality, we assume that all of the information intended for transmission through the system is carried inside ${\bar{\psi }}_{\mathrm{in}}\left(x,\omega \right)$.^8^ Note that for now ${\bar{\psi }}_{\mathrm{in}}$ is a deterministic function, not an RF.

Upon interaction with the input information field ${\bar{\psi }}_{\mathrm{in}}\left(x,\omega \right)$, the transmit system will produce total surface electric and magnetic currents $J_{e}^{t}\left(x,\omega \right)$ and $J_{m}^{t}\left(x,\omega \right)$, respectively, represented by the supervector

(10) $${\bar{J}}_{t}\left(x,\omega \right)={\left[\begin{array}{cc} J_{e}^{t}\left(x,\omega \right) & J_{m}^{t}\left(x,\omega \right) \end{array}\right]}^{⊤}.$$

Since Maxwell’s equations and the EM boundary condition of the transmitting system $S_{t}$ are both linear, we can find a response function in space–time or space–frequency describing the input–output relation [106,107,108], where here the input is the information field ${\bar{\psi }}_{\mathrm{in}}\left(x,\omega \right)$ and the output is the current ${\bar{J}}_{t}\left(x,\omega \right)$ [45]. The general structure of this response function is the following superdyad^9^ [88]:

(11) $${\bar{\bar{F}}}_{t}\left(x,x^{\prime },\omega \right)=\left[\begin{array}{cc} {\bar{F}}_{\mathrm{ee}}^{t}\left(x,x^{\prime },\omega \right) & {\bar{F}}_{\mathrm{em}}^{t}\left(x,x^{\prime },\omega \right)\\ {\bar{F}}_{\mathrm{me}}^{t}\left(x,x^{\prime },\omega \right) & {\bar{F}}_{\mathrm{mm}}^{t}\left(x,x^{\prime },\omega \right) \end{array}\right],$$

where ${\bar{F}}_{\mathrm{ee}}$, ${\bar{F}}_{\mathrm{em}}$, ${\bar{F}}_{\mathrm{me}}$, and ${\bar{F}}_{\mathrm{mm}}$ are surface dyadic functions defined for $x,x^{\prime }\in S_{t}$.^10^ In terms of this 2×2 array of surface dyads (i.e., a superdyad^11^), the induced transmitting current can be computed with the help of the following formula:

(12) $${\bar{J}}_{t}\left(x,\omega \right)=\int_{x^{\prime }\in S_{t}}d^{2}x^{\prime }\;{\bar{\bar{F}}}_{t}\left(x,x^{\prime },\omega \right)\cdot {\bar{\psi }}_{\mathrm{in}}\left(x^{\prime },\omega \right).$$

For a rigorous proof of (12), see [41,88]. Physically, the relation (12) is a compact representation of two response function equations, one involving the induced electric current while the other involves the magnetic currents. In each case, *both* the information electric and magnetic fields e and h contribute to the production of the radiating current in the most general scenario.^12^ For example, Green’s (transfer) function ${\bar{F}}_{\mathrm{ee}}$ represents the response function describing how an input electric field will produce electric current, while ${\bar{F}}_{\mathrm{em}}$ represents the other response function dictating how an input magnetic field can produce an electric current, and similarly with the other response functions.

Next, the transmitter’s induced current distribution ${\bar{J}}_{t}\left(x,\omega \right)$ will act like an input to another EM system, that of radiation into the exterior region $V_{\mathrm{ex}}$, with the corresponding output now being the electric and magnetic radiation fields $E_{\mathrm{rad}}\left(x,\omega \right)$ and $H_{\mathrm{rad}}\left(x,\omega \right)$, conveniently gathered into the supervector

(13) $${\bar{\psi }}_{\mathrm{rad}}\left(x,\omega \right)={\left[\begin{array}{cc} E_{\mathrm{rad}}\left(x,\omega \right) & H_{\mathrm{rad}}\left(x,\omega \right) \end{array}\right]}^{⊤}.$$

The proper response function is the medium radiation GF, whose general structure is the following:

(14) $$\bar{\bar{G}}\left(x,x^{\prime },\omega \right)=\left[\begin{array}{cc} {\bar{G}}_{\mathrm{ee}}\left(x,x^{\prime },\omega \right) & {\bar{G}}_{\mathrm{em}}\left(x,x^{\prime },\omega \right)\\ {\bar{G}}_{\mathrm{me}}\left(x,x^{\prime },\omega \right) & {\bar{G}}_{\mathrm{mm}}\left(x,x^{\prime },\omega \right) \end{array}\right].$$

Here, the entries of this 2×2 arrays of 3-dimensional dyads—the quantities ${\bar{G}}_{\mathrm{ee}}$, ${\bar{G}}_{\mathrm{em}}$, ${\bar{G}}_{\mathrm{em}}$, and ${\bar{G}}_{\mathrm{mm}}$—serve as the electric/electric, electric/magnetic, magnetic/electric, and magnetic/magnetic dyadic GFs, respectively [44,109]. We interpret them as the transfer or response function connecting information in electric/magnetic currents with their radiated electric/magnetic currents.^13^ Their meaning will become more manifest when the following radiation integral is considered:

(15) $${\bar{\psi }}_{\mathrm{rad}}\left(x,\omega \right)=\int_{x^{\prime }\in S_{t}}d^{2}x^{\prime }\;\bar{\bar{G}}\left(x,x^{\prime },\omega \right)\cdot {\bar{J}}_{t}\left(x^{\prime },\omega \right),$$

which is essentially the EM radiation system input–output relation.

The medium dyadic GFs appearing as entries in the superarray (14) include, most notably, the vacuum GFs, which capture the process of EM radiation from a source into free space (see Appendix D and references [44,56,57,93,104,105]). However, it should be noted that more complex scenarios can also be accommodated, such as media consisting of lossy ground planes and planar multilayer structures [44,104], or random environments [96]. The analysis presented in this paper intentionally keeps the details of the medium GFs $\bar{\bar{G}}\left(x,x^{\prime },\omega \right)$ as general as possible to maintain the broadest scope for the theory. Nevertheless, in both theory and computational models, the vacuum GF (Appendix D) remains the most important special case.

In the next stage, the radiation fields produced in accordance with (15) will reach the scattering system and produces an induced current over the surface $S_{s}$. Again, the mechanism is identical to that involving $\bar{J}$ in (10). The induced current is written as the supervector

(16) $${\bar{J}}_{s}\left(x,\omega \right)={\left[\begin{array}{cc} J_{e}^{s}\left(x,\omega \right) & J_{m}^{s}\left(x,\omega \right) \end{array}\right]}^{⊤},$$

where $J_{e}^{s}\left(x,\omega \right)$ and $J_{m}^{s}\left(x,\omega \right)$ are the electric and magnetic currents on the scatterers, respectively. Similar to (11), we also have a scattering current GF whose general structure is given by the superdyad

(17) $${\bar{\bar{F}}}_{s}\left(x,x^{\prime },\omega \right)=\left[\begin{array}{cc} {\bar{F}}_{\mathrm{ee}}^{s}\left(x,x^{\prime },\omega \right) & {\bar{F}}_{\mathrm{em}}^{s}\left(x,x^{\prime },\omega \right)\\ {\bar{F}}_{\mathrm{me}}^{s}\left(x,x^{\prime },\omega \right) & {\bar{F}}_{\mathrm{mm}}^{s}\left(x,x^{\prime },\omega \right) \end{array}\right].$$

In terms of this response function, the induced current can be computed by means of the relation

(18) $${\bar{J}}_{s}\left(x,\omega \right)=\int_{x^{\prime }\in S_{s}}d^{2}x^{\prime }\;{\bar{\bar{F}}}_{s}\left(x,x^{\prime },\omega \right)\cdot {\bar{\psi }}_{\mathrm{rad}}\left(x^{\prime },\omega \right).$$

Next, the induced surface current distribution ${\bar{J}}_{s}\left(x,\omega \right)$ will radiate the following scattering fields into the exterior region $V_{\mathrm{ex}}$:

(19) $${\bar{\psi }}_{s}\left(x,\omega \right)={\left[\begin{array}{cc} E_{s}\left(x,\omega \right) & H_{s}\left(x^{\prime },\omega \right) \end{array}\right]}^{⊤},$$

where $E_{s}\left(x,\omega \right)$ and $H_{s}\left(x,\omega \right)$ are scattered electric and magnetic fields, respectively. The same medium GFs that were used before to describe the radiation of the $S_{t}$ system into the exterior region $V_{\mathrm{ex}}$ will now be deployed for the computation of EM radiation by scatterers, leading to

(20) $${\bar{\psi }}_{s}\left(x,\omega \right)=\int_{x^{\prime }\in S_{s}}d^{2}x^{\prime }\;\bar{\bar{G}}\left(x,x^{\prime },\omega \right)\cdot {\bar{J}}_{s}\left(x^{\prime },\omega \right).$$

Consequently, the total fields now in the region $V_{\mathrm{ex}}$ are the superposition of the direct fields produced by the transmitter and the indirect fields radiated by the scattering system:

(21) $$\bar{\psi }\left(x,\omega \right)={\bar{\psi }}_{\mathrm{rad}}\left(x,\omega \right)+{\bar{\psi }}_{s}\left(x,\omega \right).$$

Finally, this total field $\bar{\psi }\left(x,\omega \right)$ will impinge on the receiving system $S_{r}$, whose current GF is given by

(22) $${\bar{\bar{F}}}_{r}\left(x,x^{\prime },\omega \right)=\left[\begin{array}{cc} {\bar{F}}_{\mathrm{ee}}^{r}\left(x,x^{\prime },\omega \right) & {\bar{F}}_{\mathrm{em}}^{r}\left(x,x^{\prime },\omega \right)\\ {\bar{F}}_{\mathrm{me}}^{r}\left(x,x^{\prime },\omega \right) & {\bar{F}}_{\mathrm{mm}}^{r}\left(x,x^{\prime },\omega \right) \end{array}\right],$$

which upon interaction with its boundary condition will produce an induced surface current

(23) $${\bar{J}}_{r}\left(x,\omega \right)={\left[\begin{array}{cc} J_{e}^{r}\left(x,\omega \right) & J_{m}^{r}\left(x,\omega \right) \end{array}\right]}^{⊤}$$

via the relation

(24) $${\bar{J}}_{r}\left(x,\omega \right)=\int_{x^{\prime }\in S_{r}}d^{2}x^{\prime }\;{\bar{\bar{F}}}_{r}\left(x,x^{\prime },\omega \right)\cdot \bar{\psi }\left(x^{\prime },\omega \right).$$

The received current distribution, ${\bar{J}}_{r}\left(x,\omega \right)$, is often regarded as the terminal signal in a generic EM information transmission system. Consequently, the radiation field generated by this current is typically not of interest, as the extraction of information is generally performed directly from ${\bar{J}}_{r}$ itself.

### 2.3. The Combined Response Function of the Generic Deterministic Electromagnetic Information Transmission System

We now proceed to combine all the geometric and EM structures outlined in Section 2.1 and Section 2.2. We begin with a rigorous definition of an EM information transmission system. Following this, we derive the single total end-to-end response function for the entire system, which starts at the transmitter $S_{t}$, traverses the scattering environment $S_{s}$, and concludes at the receiver $S_{r}$.

**Definition** **1** (the generic deterministic electromagnetic information transmission system)**.**  *Consider the transmitter, scattering, and receiver systems, geometrically modeled as closed, multiply connected, smooth 2-manifolds $S_{t}$, $S_{s}$, and $S_{r}$, as described in *(6)* and *(7)*. The EM information transmission system $\left(S_{t},S_{s},S_{r}\right)$ consists of these three manifolds, along with the following associated data:*

*1.*  *Three current GFs ${\bar{\bar{F}}}_{t}\left(x,x^{\prime },\omega \right)$, ${\bar{\bar{F}}}_{s}\left(x,x^{\prime },\omega \right)$, and ${\bar{\bar{F}}}_{r}\left(x,x^{\prime },\omega \right)$, which are associated with the three smooth 2-manifolds $S_{t}$, $S_{s}$, $S_{r}$, respectively, whose structures are as specified by *(11)*, *(17)*, and *(22)*.*
*2.*  *A radiation GF $\bar{\bar{G}}\left(x,x^{\prime },\omega \right)$, with a structure as given by *(14)*, which is associated with the medium exterior to the closed manifolds $S_{t}$, $S_{s}$, $S_{r}$, i.e., defined on the exterior region $V_{\mathrm{ex}}=R^{3}\setminus \left(V_{t}\cup V_{s}\cup V_{r}\right)$.*

*Information is injected into the system through an input external excitation field ${\bar{\psi }}_{\mathrm{in}}\left(x,\omega \right)$ as given by *(9)*, which interacts with the transmitter system S. Information propagates from $S_{t}$ to $S_{r}$ possibly through $S_{s}$.*

For brevity, we will omit explicit references to the four EM GFs mentioned in Definition 1. Instead, we will refer to the information transmission or communication system simply as $\left(S_{t},S_{s},S_{r}\right)$, with the intention being that those omitted EM response functions are implied in the original reference.

**Figure 2:**
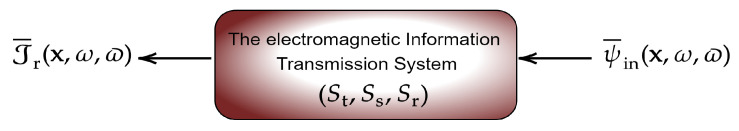
The general form of an $\left(S_{t},S_{s},S_{r}\right)$ electromagnetic communication system including the transmitting, scattering, and receiving subsystems. The input in general is an RF defined on $x\in S_{t}$ with frequency ω and state (outcome) ϖ∈Ω, where Ω is the state space (space of outcomes); see Definition A1.

Figure 2 illustrates the general concept of the response function approach to EIT. Input information is injected into the transmission system $\left(S_{t},S_{s},S_{r}\right)$ via the input RF ${\bar{\psi }}_{\mathrm{in}}\left(x,\omega ,\varpi \right)$, while the output is the receiving system current distribution $\bar{J_{r}}\left(x,\omega ,\varpi \right)$. Note that ϖ∈Ω represents the probabilistic state (outcome), as defined in Definition A1 in Appendix B. This highlights the stochastic nature of these fields, even though we have been working with deterministic fields so far. A more careful introduction to RFT will be provided in Section 3 and after.

We now need to explicitly characterize the flow of information in the EM system under consideration. We begin with the following result, which expresses the receiver current as a sum of six-fold and three-fold integrals involving all associated EM GFs and the input information field.

**Theorem** **1.** *Consider an EM transmission system $\left(S_{t},S_{s},S_{r}\right)$ as in Definition 1. Assume that information is injected into the transmitting system $S_{t}$ through an excitation field ${\bar{\psi }}_{\mathrm{in}}\left(x,\omega \right)$. Then the receiver-induced surface current distribution can be computed by Formula *(30)*.*

**Proof.**  We note that the total received current can be written as the sum of two components as follows:

(25) $${\bar{J}}_{r}\left(x,\omega \right)={\bar{J}}_{r}^{\mathrm{los}}\left(x,\omega \right)+{\bar{J}}_{r}^{\mathrm{nlos}}\left(x,\omega \right),$$

where ${\bar{J}}_{r}^{\mathrm{los}}\left(x,\omega \right)$ is the line-of-sight (LoS) component obtained by the interaction of the receiving system $S_{r}$ with the component of the radiation field produced directly by the transmitter current ${\bar{J}}_{t}$, i.e., the field ${\bar{\psi }}_{\mathrm{rad}}$. On the other hand, ${\bar{J}}_{r}^{\mathrm{nlos}}\left(x,\omega \right)$ is the current component induced in the receiving system $S_{r}$ due to non-line-of-sight (NLoS) fields scattered by the intervening scattering system $S_{s}$. The relation (25) can be easily deduced by substituting (21) into (24) and is a direct consequence of the linearity of Maxwell’s equations. In detail, we find

(26) $${\bar{J}}_{r}^{\mathrm{los}}\left(x,\omega \right)=\int_{x^{\prime }\in S_{r}}d^{2}x^{\prime }\;{\bar{\bar{F}}}_{r}\left(x,x^{\prime },\omega \right)\cdot {\bar{\psi }}_{\mathrm{rad}}\left(x^{\prime },\omega \right)$$

and

(27) $${\bar{J}}_{r}^{\mathrm{nlos}}\left(x,\omega \right)=\int_{x^{\prime }\in S_{r}}d^{2}x^{\prime }\;{\bar{\bar{F}}}_{r}\left(x,x^{\prime },\omega \right)\cdot {\bar{\psi }}_{s}\left(x^{\prime },\omega \right).$$

**Figure 3:**
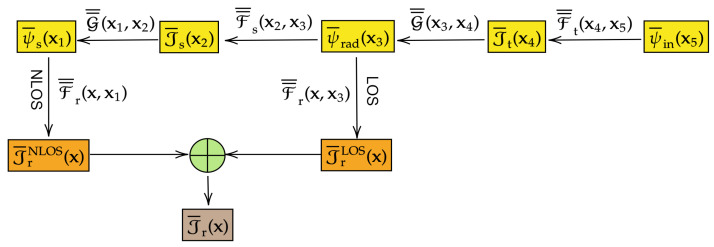
The general formal structure of an $\left(S_{t},S_{s},S_{r}\right)$ EM communication system characterized by the transmitting, scattering, and receiving GFs ${\bar{\bar{F}}}_{t}$, ${\bar{\bar{F}}}_{s}$, and ${\bar{\bar{F}}}_{r}$, and radiation GF $\bar{\bar{G}}$. Six distinct coordinate systems are needed in order to express the received current $\bar{J}\left(x\right)$ in terms of the input information field $\bar{\psi }\left(x_{5}\right)$. The global coordinate system is x.

The following calculations are significantly simplified by considering the formal structure of the field transmission problem, which is encapsulated in terms of all relevant EM GFs. Figure 3 illustrates the overall structure. Let us begin by computing ${\bar{J}}_{r}^{\mathrm{nlos}}$. A total of six distinct coordinate systems, represented by the dummy variables $x_{i}$, where i∈{1,2,3,4,5}, in addition to the original global frame x, are required to fully characterize the dynamics of EM information flow throughout the communication system via the corresponding response functions. In particular, in order to determine the NLoS component of the receiver’s current at position x, we need to use all the dummy coordinate systems from $x_{1}$ to $x_{5}$ shown in Figure 3. By substituting (12), (15), (18), and (20) into (27), and then converting repeated integrals into multiple integrals using the Fubini’s theorem [111,112], we immediately arrive at the following expression:

(28) $$\begin{array}{c} {\bar{J}}_{r}^{\mathrm{nlos}}\left(x\right)=\int_{S_{r}}d^{2}x_{1}\int_{S_{s}}d^{2}x_{2}\int_{S_{s}}d^{2}x_{3}\int_{S_{t}}d^{2}x_{4}\int_{S_{t}}d^{2}x_{5}\;{\bar{\bar{F}}}_{r}\left(x,x_{1}\right)\cdot \bar{\bar{G}}\left(x_{1},x_{2}\right)\cdot {\bar{\bar{F}}}_{s}\left(x_{2},x_{3}\right)\cdot \bar{\bar{G}}\left(x_{3},x_{4}\right)\cdot {\bar{\bar{F}}}_{t}\left(x_{4},x_{5}\right)\cdot {\bar{\psi }}_{\mathrm{in}}\left(x_{5}\right). \end{array}$$

Next, for the LoS component, only the coordinate systems $x_{1}$, $x_{4}$, and $x_{5}$ are required. This is because the field impinging on the receiver system $S_{r}$ consists solely of the radiation fields ${\bar{\psi }}_{\mathrm{rad}}$ directly produced by the transmitter system $S_{t}$, without interacting with the scattering system $S_{s}$ (see Figure 3). The results are given by the following expression:

(29) $$\begin{array}{cc} {\bar{J}}_{r}^{\mathrm{los}}\left(x,\omega \right) & =\int_{S_{r}}d^{2}x_{1}\int_{S_{t}}d^{2}x_{4}\int_{S_{t}}d^{2}x_{5}\;{\bar{\bar{F}}}_{r}\left(x,x_{1},\omega \right)\cdot \bar{\bar{G}}\left(x_{1},x_{4},\omega \right)\cdot {\bar{\bar{F}}}_{t}\left(x_{4},x_{5},\omega \right)\cdot {\bar{\psi }}_{\mathrm{in}}\left(x_{5},\omega \right). \end{array}$$

Substituting (28) and (29) into (25), the final expression for the received current (30) is obtained.    □

(30) $$\begin{array}{c} {\bar{J}}_{r}\left(x\right)=\int_{S_{r}}d^{2}x_{1}\int_{S_{s}}d^{2}x_{2}\int_{S_{s}}d^{2}x_{3}\int_{S_{t}}d^{2}x_{4}\int_{S_{t}}d^{2}x_{5}\;{\bar{\bar{F}}}_{r}\left(x,x_{1}\right)\cdot \bar{\bar{G}}\left(x_{1},x_{2}\right)\cdot {\bar{\bar{F}}}_{s}\left(x_{2},x_{3}\right)\cdot \bar{\bar{G}}\left(x_{3},x_{4}\right)\cdot {\bar{\bar{F}}}_{t}\left(x_{4},x_{5}\right)\cdot {\bar{\psi }}_{\mathrm{in}}\left(x_{5}\right)\\ \;\;\;\;\;\;\;\;\;\;\;\;\;\;\;\;\;\;\;\;+\int_{S_{r}}d^{2}x_{3}\int_{S_{t}}d^{2}x_{4}\int_{S_{t}}d^{2}x_{5}\;{\bar{\bar{F}}}_{r}\left(x,x_{3}\right)\cdot \bar{\bar{G}}\left(x_{3},x_{4}\right)\cdot {\bar{\bar{F}}}_{t}\left(x_{4},x_{5}\right)\cdot {\bar{\psi }}_{\mathrm{in}}\left(x_{5}\right). \end{array}$$

As an application of Theorem 1, we may invoke the Fubini theorem again to rearrange the six-fold and three-fold multiple integrals in (30) in order to derive the following equivalent but more memorable intuitive form:

(31) $$\begin{array}{cc} {\bar{J}}_{r}\left(x,\omega \right) & =\int_{S_{t}}d^{2}x^{\prime }\;{\bar{\bar{B}}}^{\mathrm{nlos}}\left(x,x^{\prime },\omega \right)\cdot {\bar{\psi }}_{\mathrm{in}}\left(x^{\prime },\omega \right)+\int_{S_{t}}d^{2}x^{\prime }\;{\bar{\bar{B}}}^{\mathrm{los}}\left(x,x^{\prime },\omega \right)\cdot {\bar{\psi }}_{\mathrm{in}}\left(x^{\prime },\omega \right). \end{array}$$

The expression (31) models the system in Figure 2 in terms of two response functions, one associated with NLoS propagation and the other with the LoS path. It is interesting to notice that the integation operations in (31) are performed only with respect to the transmitting surface $S_{t}$. In other words, we have managed to encode all information about the details of EM interactions with the subsystems $S_{s}$ and $S_{r}$ into the two response functions ${\bar{\bar{B}}}^{\mathrm{nlos}}\left(x,x^{\prime }\right)$ and ${\bar{\bar{B}}}^{\mathrm{los}}\left(x,x^{\prime }\right)$. The LoS response function is expressed as

(32) $$\begin{array}{ccc} \; & {\bar{\bar{B}}}^{\mathrm{los}}\left(x,x^{\prime },\omega \right) & :=\int_{S_{r}}d^{2}x_{1}\int_{S_{t}}d^{2}x_{4}\;{\bar{\bar{F}}}_{r}\left(x,x_{1},\omega \right)\cdot \bar{\bar{G}}\left(x_{1},x_{4},\omega \right)\cdot {\bar{\bar{F}}}_{t}\left(x_{4},x^{\prime },\omega \right), \end{array}$$

while the NLoS response function can be written as

(33) $$\begin{array}{cc} {\bar{\bar{B}}}^{\mathrm{nlos}} & \left(x,x^{\prime },\omega \right):=\int_{S_{r}}d^{2}x_{1}\int_{S_{s}}d^{2}x_{2}\int_{S_{s}}d^{2}x_{3}\int_{S_{t}}d^{2}x_{4}\;{\bar{\bar{F}}}_{r}\left(x,x_{1},\omega \right)\cdot \bar{\bar{G}}\left(x_{1},x_{2},\omega \right)\cdot {\bar{\bar{F}}}_{s}\left(x_{2},x_{3},\omega \right)\cdot \;\bar{\bar{G}}\left(x_{3},x_{4},\omega \right)\cdot {\bar{\bar{F}}}_{t}\left(x_{4},x^{\prime },\omega \right). \end{array}$$

Physically, both ${\bar{\bar{B}}}^{\mathrm{nlos}}\left(x,x^{\prime }\right)$ and ${\bar{\bar{B}}}^{\mathrm{los}}\left(x,x^{\prime }\right)$ can be understood as the effective proper EM response functions equivalent to cascade connection of multiple processes. For example, the expression of ${\bar{\bar{B}}}^{\mathrm{los}}\left(x,x^{\prime }\right)$ in (32) captures the effective transfer of information from every point $x^{\prime }\in S_{t}$ to every other point $x_{4}\in S_{t}$ mediated by the transmitter antenna/surface current GF ${\bar{\bar{F}}}_{t}\left(x_{4},x^{\prime }\right)$, followed by coupling the point $x_{4}\in S_{t}$ with another generic point $x_{1}\in S_{r}$ on the receiving system through the radiation GF $\bar{\bar{G}}\left(x_{1},x_{4}\right)$, followed by finally coupling each point $x_{1}\in S_{r}$ with another point $x\in S_{r}$ where information (observation) will be connected, this time mediated by ${\bar{\bar{F}}}_{r}\left(x,x_{3}\right)$. As the reader can easily notice, this logic is very intuitive and can be applied again to the corresponding analysis of ${\bar{\bar{B}}}^{\mathrm{nlos}}\left(x,x^{\prime }\right)$ via (33).

If the detailed split of the total response function into LoS and NLoS components is not important, one can combine the two integrals in the RHS of (31) into a single integral as follows:

(34) $$\begin{array}{c} {\bar{J}}_{r}\left(x,\omega ,\varpi \right)=\int_{S_{t}}d^{2}x^{\prime }\;\bar{\bar{B}}\left(x,x^{\prime },\omega ,\varpi \right)\cdot {\bar{\psi }}_{\mathrm{in}}\left(x^{\prime },\omega ,\varpi \right), \end{array}$$

where the *reduced total system function* is defined as

(35) $$\bar{\bar{B}}\left(x,x^{\prime },\omega ,\varpi \right):={\bar{\bar{B}}}^{\mathrm{nlos}}\left(x,x^{\prime },\omega ,\varpi \right)+{\bar{\bar{B}}}^{\mathrm{los}}\left(x,x^{\prime },\omega ,\varpi \right).$$

**Figure 4:**
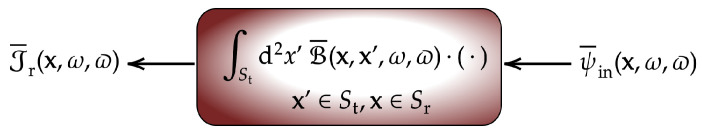
A specific realization of the general stochastic EM information system (Definition 3), illustrated in Figure 2, can be constructed by utilizing the reduced system function as defined by relation (35).

For completeness, and anticipating future development in the theory, it is important to note that the reduced system function $\bar{\bar{B}}$ in (34) and (35) is assumed to be stochastic (i.e., promoted to an RF status), which will be rigorously developed starting in Section 3.3 and onwards. The reduced model is illustrated in Figure 4, representing one possible realization of the global input–output relation for the general EM information transmission system depicted in Figure 2. However, it is important to note that the reduced system function in (4) is just one approach to realizing the general system $\left(S_{t},S_{s},S_{r}\right)$ and is by no means unique. More detailed system models can be developed, as will be demonstrated in the forthcoming sections.

## 3. Electromagnetic Random Field Theory

### 3.1. Introduction to Random Field Theory and Why We Need It

We now turn to the development of a suitable stochastic EMT for information and communication. Our main tools remain the EM GF formalism developed earlier, but now we augment it with a stochastic dimension, integrating the structure of the physical problem of information transfer and flow through RFT, which is a multidisciplinary branch of science intersecting mathematics, engineering, physics, and other research areas such as earth science and meteorology [113,114]. As mentioned earlier, the integration of RFT and EMT attempted here is dubbed EM-RFT.

RFT models radiating currents and radiated fields as stochastic processes defined on a space *M*, whose dimensionality is often greater than one. This space, *M*, can be as general as a topological space, typically equipped with a metric [115]. For example, RFT can be developed for spaces even more general than smooth manifolds, such as piecewise smooth manifolds, stratified manifolds, or Gaussian spaces. These frameworks intersect significantly with both integral and differential geometry, particularly in the context of Gaussian RFs [115,116,117].

At a general level, the EM field can be modeled from a stochastic perspective as the sum of three contributions: trend (average value), fluctuations, and noise. Typically, we represent an empirical RF f(x,ϖ) defined on a manifold M∋x as [118]

(36) f(x,ϖ)=E{f(x,ϖ)}+δf(x,ϖ)+n(x,ϖ),

where the trend or average value E{f(x,ϖ)} represents the deterministic component of the field, while the fluctuation δf(x,ϖ) models f(x,ϖ)−E{f(x,ϖ)} for noiseless fields [43]. Here, n(x,ϖ) is the noise field injected into the system and is typically due to random thermal fluctuations in the electronics (thermal noise).

This paper primarily focuses on the fluctuations, which we consider the *intrinsic* component of randomness inherent in the physics of the RF under consideration. The distinction between the noise process n(x,ϖ) and RFs f(x,ϖ) lies in their correlation properties. RFs exhibit nontrivial correlation properties, behaving as structured randomness, whereas noise fields are modeled as white stochastic processes with maximal uncorrelation. Specifically, for $x\neq x^{\prime }$, the random variables n(x,ϖ) and $n(x^{\prime },\varpi )$ tend to be uncorrelated [118]. However, it should be noted that, in practice, it is not always clear how to separate the noise component n(x,ϖ) from the fluctuations δf(x,ϖ). As a result, many researchers tend to combine them, at least in the initial stages of modeling the RF structure of a given physical problem [118].

In information transmission systems, which are the primary focus of this study, the stochastic structure of the EM fields can be attributed to well-defined and distinct sources, primarily randomness in the propagation environment, especially due to the interaction of EM fields with random clusters of scatterers [96]. Random scattering theory leads to stochastic structures different from thermal noise induced, for example, by the random motion of electrons in devices and materials [8]. This latter type of randomness can be modeled as a separate additive white Gaussian noise (AWGN), represented as the random process n(x,ϖ) in (36). Consequently, in this paper, we intentionally avoid incorporating the noise term n(x,ϖ) unless we explicitly deal with Gaussian noisy channels, such as the standard MIMO system. Instead, we focus on the fluctuation term δf(x,ϖ), which here becomes the main subject of investigation in EM-RFT.

### 3.2. Unified Representation of Complex-Valued Random Fields in Electromagnetic Information Theory

As discussed in Section 2, particularly upon reviewing tables such as Table 2 and Table 3, it becomes evident that all EM fields in our formulation are, in general, complex (refer to the frequency–space model [44,92]). Furthermore, several distinct data types are employed, including Cartesian vectors, dyadic GFs, supervectors, and superdyads (see Appendix A). Consequently, working with all these types simultaneously presents several challenges:

1. The formulation of mathematical theorems regarding RFs is contingent upon their representation. When multiple data types (such as vectors, dyads, superdyads, etc.) are present, it becomes challenging to develop the theory in an efficient and concise manner for the purposes of information theory and signal processing.
2. The computational implementation and simulation of RFs are critically dependent on their structural nature, whether they are real or complex vectors, matrices, etc.
3. In the advanced stages of the theory, to be elaborated in subsequent sections, a comprehensive characterization of correlation propagation and information flow will necessitate the introduction of even larger and more complex types of RFs, such as the correlation propagators listed in Table 5.

Therefore, there is a need to develop a unified method to represent all EM RFs in a consistent manner. While there is no single unique approach to achieve this, we propose the following procedure:

1. Construct a comprehensive theory of real-valued vector RFs with arbitrary dimensions.
2. Transform all frequency-domain complex-valued EM RFs into equivalent real-valued vector RFs.
3. Perform computations, establish mathematical definitions, and prove theorems ultimately in terms of the equivalent real-valued vector RF representation above.
4. Transform back to the complex-valued RFs, whether they are vectors, supervectors, dyads, superdyads, etc.

The real vector RF model presented in Appendix B establishes a framework for ultimately grounding all complex vector, dyadic, supervector, and superdyadic RFs developed in Section 2 in a unified manner suitable for both theoretical analysis and computational applications. The details of this process are elaborated in Appendix H–Appendix J. Algorithm A1 describes how each generic EM RF g(x,ω,ϖ)∈V, where V is defined in Table 2, can be transformed into (and from) its complex and real vector representations $g^{\left(c\right)}$ and $g^{\left(r\right)}$, respectively. In this way, each EM RF can be treated as one of the following data types:

1. **Level I**: Original generic forms g(x,ω,ϖ)∈V: This form is most suitable for establishing connections with the physics of the EM information system. Here, this is the frequency–space representation.
2. **Level II**: Complex-valued vector form $g^{\left(c\right)}$: This representation is optimal for presenting aspects of the mathematical treatment in EIT, as complex-valued RFs are fundamental to information theory and signal processing [2,94]. For example, complex-valued signal processing leverages the structure of complex variables underlying frequency-domain RFs to simplify the presentation of certain theorems or devise novel communication and signal processing methods [119].
3. **Level III**: Real-valued vector form $g^{\left(r\right)}$: This is the essential form for constructing mathematical definitions and proving theorems. Concepts such as probability density functions, entropy, and mutual information are ultimately defined in terms of this real form. Furthermore, computational models and simulations are constructed at the level of the real vector representation.

To illustrate the hierarchical representation levels, consider the example of a stochastic GF such as ${\bar{\bar{F}}}_{b}$, rigorously constructed as an RF in Section 3.4. This GF represents a superdyadic array consisting of four complex-valued dyads defined on the 2-manifold $S_{b}$. From a physical perspective, it is most instructive to capture this object through its defining equations—such as (11), (17), or (22)—constituting the **Level I** representation. However, to elevate this response function to the status of an RF, we must introduce second-order statistical information, primarily through correlation data such as covariance and pseudo-covariance functions. In EMT, these quantities are most effectively computed in the frequency domain [47,59,61,120]. This necessitates a complex vector representation of the superdyad ${\bar{\bar{F}}}_{b}$, specifically the complex vector ${\bar{\bar{F}}}_{b}^{\left(c\right)}$ at **Level II**, from which mean, covariance, and pseudo-covariance can be readily constructed (see Appendix H). Finally, to compute and rigorously define all derived quantities, such as entropy and mutual information, we employ **Level III**, the real vector form ${\bar{\bar{F}}}_{b}^{\left(r\right)}$, which serves as the foundational data type for *all* quantities considered in EM-RFT.

### 3.3. Construction of Electromagnetic Random Fields and Currents in Deterministic Information Transmission Systems

In a deterministic EM information system, as defined in Definition 1, information is injected through an input RF ${\bar{\psi }}_{\mathrm{in}}\left(x\right)$ with mean function $E\left[{\bar{\psi }}_{\mathrm{in}}\left(x,\omega ,\varpi \right)\right]$, covariance function function $C_{{\bar{\psi }}_{\mathrm{in}}}\left(x,x^{\prime },\omega \right)$, and pseudo-covariance function $C_{{\bar{\psi }}_{\mathrm{in}}}^{\prime }\left(x,x^{\prime },\omega \right)$. Note that all of these statistical functions are constructed relative to the complex vector representation. That is, throughout this paper, the notation of Definition A10 will be consistently applied (see also Remark A2).

While the various EM GFs remain deterministic, we now examine the EM response to the input excitation RF ${\bar{\psi }}_{\mathrm{in}}$. The induced currents ${\bar{J}}_{t}$, ${\bar{J}}_{s}$, ${\bar{J}}_{r}$, and the radiation and scattered fields ${\bar{\psi }}_{\mathrm{rad}}$ and ${\bar{\psi }}_{s}$ become RFs, as they depend linearly on the input RF ${\bar{\psi }}_{\mathrm{in}}\left(x,\omega ,\varpi \right)$ according to the relations developed in Section 2.2. Our main objective now is to examine the generic response field $\bar{R}\left(x,\omega ,\varpi \right)$ induced in response to the generic input field $\bar{E}\left(x,\omega ,\varpi \right)$, i.e., the transformation process

(37) $$\bar{E}\left(x,\omega ,\varpi \right)\overset{\mathrm{EM}\;\mathrm{interaction}\;\mathrm{and}\;\mathrm{radiation}}{\underset{\mathrm{GF}}{\to }}\bar{R}\left(x,\omega ,\varpi \right),$$

where

(38) $$\begin{array}{ccc} \; & \bar{E}\in \left\{{\bar{\psi }}_{\mathrm{in}},{\bar{\psi }}_{\mathrm{rad}},{\bar{\psi }}_{s},{\bar{J}}_{t},{\bar{J}}_{s}\right\},\; & \bar{R}\in \left\{{\bar{\psi }}_{\mathrm{rad}},{\bar{\psi }}_{s},{\bar{J}}_{t},{\bar{J}}_{s},{\bar{J}}_{r}\right\}. \end{array}$$

The following theorem asserts that, under appropriate regularity conditions, if the input information source field ${\bar{\psi }}_{\mathrm{in}}$ is an RF, then all corresponding response fields, collectively denoted by $\bar{R}$ [see (38)], in the generic deterministic problem described by (37), are also RFs, and satisfy the same regularity conditions as the input RF.

**Theorem** **2.**  *Consider a deterministic EM information system as specified in Definition 1 such that for all $\omega \in R^{+}$:*

*1.*  *Information is injected through an input RF ${\bar{\psi }}_{\mathrm{in}}\left(x\right)$ satisfying:*
(39) $$\left\{\begin{array}{c} E\left[{\bar{\psi }}_{\mathrm{in}}\left(x,\omega ,\varpi \right)\right]\;\mathrm{is}\;\mathrm{continuous}\;\mathrm{on}\;S_{t},\\ C_{{\bar{\psi }}_{\mathrm{in}}}\left(x,x^{\prime },\omega \right)\;\mathrm{is}\;\mathrm{continuous}\;\mathrm{on}\;S_{t}\times S_{t}. \end{array}\right.$$
*2.*  *The EM medium is deterministic. Furthermore, it is regular in the sense that the medium Green function (GF) $\left(x,x^{\prime }\right)\mapsto \bar{\bar{G}}\left(x,x^{\prime },\omega \right)$ is at least piecewise continuous and bounded for $(x,x^{\prime })\in V\times S$, where*
(40) $$V:=R^{3}\setminus ⋃V_{b},\;S:=⋃S_{b}.$$
*Here, $V_{b}$, b∈{t,s,r}, is a (topologically) closed 3-dimensional region whose boundary is the closed manifold $S_{b}$ (the latter term means a compact manifold that has no boundary). In other words, V is the exterior region of the GF’s domain and is the same as $V_{\mathrm{ex}}$ defined in *(4)* (however, see Remark 2).*

*The response fields, denoted generically as $\bar{R}\left(x,\omega ,\varpi \right)$, are the induced currents ${\bar{J}}_{b}\left(x,\omega ,\varpi \right)$ for $x\in S_{b},b\in \left\{t,s,r\right\}$, the radiation and scattered fields ${\bar{\psi }}_{\mathrm{rad}}\left(x,\omega ,\varpi \right)$ and ${\bar{\psi }}_{s}\left(x,\omega ,\varpi \right)$ for $x\in V_{\mathrm{ex}}$. Then we have the following:*

*1.*  *All response fields become RFs $\bar{R}\left(x,\omega ,\varpi \right)$ on their respective domains.*
*2.*  *All of the above RFs have spatially continuous mean, covariance, and pseudo-covariance functions on their respective domains.*

**Proof.**  See Appendix K.    □

We note from the proof of Theorem 2 that all GF response integrals of the form (A89), encompassing Formulas (12), (15), (18), (20), and (24), are built up via the concept of m.s. stochastic integration defined in Appendix B (Definition A5). The rigorous construction of EM RFs then hinges on the regularity of (1) the input stochastic RF correlation properties, and (2) the relevant GFs.

We summarize the formal structure associated with Theorem 2 through the three transformations given by (41), (42), and (43). In the deterministic LoS process described by (41), the input information field is elevated from the deterministic field ${\bar{\psi }}_{\mathrm{in}}\left(x,\omega \right)$ to the RF ${\bar{\psi }}_{\mathrm{in}}\left(x,\omega ,\varpi \right)$. On the other hand, the two related EM GFs ${\bar{\bar{F}}}_{t}\left(x,x^{\prime },\omega \right)$ and $\bar{\bar{G}}\left(x,x^{\prime },\omega \right)$ are *not* treated as RFs. In this context, Theorem 2 asserts that the response fields are RFs, while the GFs remain deterministic here. Similarly, for the deterministic NLoS and receiver processes described in (42) and (43), all EM GFs are maintained as deterministic, while the input from the previous stage is an RF. In summary, these transformations encapsulate the complete causal (chain-like) structure of EM interactions in deterministic information transmission systems, effectively captured through the EM response function method.

Deterministic LoS Process:

(41) $$\begin{array}{c} {\bar{\psi }}_{\mathrm{in}}\left(x,\omega ,\varpi \right)\overset{{\bar{\bar{F}}}_{t}\left(x,x^{\prime },\omega \right)}{\to }{\bar{J}}_{t}\left(x,\omega ,\varpi \right)\overset{\bar{\bar{G}}\left(x,x^{\prime },\omega \right)}{\to }{\bar{\psi }}_{\mathrm{rad}}\left(x,\omega ,\varpi \right) \end{array}$$

Deterministic NLoS Process:

(42) $$\begin{array}{c} {\bar{\psi }}_{\mathrm{in}}\left(x,\omega ,\varpi \right)\overset{{\bar{\bar{F}}}_{t}\left(x,x^{\prime },\omega \right)}{\to }{\bar{J}}_{t}\left(x,\omega ,\varpi \right)\overset{\bar{\bar{G}}\left(x,x^{\prime },\omega \right)}{\to }{\bar{\psi }}_{\mathrm{rad}}\left(x,\omega ,\varpi \right)\overset{{\bar{\bar{F}}}_{s}\left(x,x^{\prime },\omega \right)}{\to }{\bar{J}}_{s}\left(x,\omega ,\varpi \right)\overset{\bar{\bar{G}}\left(x,x^{\prime },\omega \right)}{\to }{\bar{\psi }}_{s}\left(x,\omega ,\varpi \right) \end{array}$$

Deterministic Receiver Interaction Process:

(43) $$\begin{array}{c} {\bar{\psi }}_{\mathrm{rad}}\left(x,\omega ,\varpi \right)+{\bar{\psi }}_{s}\left(x,\omega ,\varpi \right)\overset{{\bar{\bar{F}}}_{r}\left(x,x^{\prime },\omega \right)}{\to }{\bar{J}}_{r}\left(x,\omega ,\varpi \right)={\bar{J}}_{r}^{\mathrm{los}}\left(x,\omega ,\varpi \right)+{\bar{J}}_{r}^{\mathrm{nlos}}\left(x,\omega ,\varpi \right) \end{array}$$

**Remark** **2.** *The region $V=V_{\mathrm{ex}}$ invoked in Theorem 2, as originally defined by *(4)*, represents the exterior domain for the medium GF $\bar{\bar{G}}\left(x,x^{\prime },\omega \right)$. It is important to note that this GF exhibits a singularity at $x=x^{\prime }$ [44,104,109]. The regularity condition imposed on the medium GF in Theorem 2 is neither stringent nor difficult to satisfy in practical applications. Specifically, in the case of a vacuum—the most commonly encountered medium in practice—the GF $\left(x,x^{\prime }\right)\mapsto \bar{\bar{G}}\left(x,x^{\prime },\omega \right)$ is smooth for $x\neq x^{\prime }$, which is automatically satisfied when x∈V. For other media types, such as those described by multilayered GFs, the GFs are typically at least piecewise smooth [44,104].*

### 3.4. Construction of Electromagnetic Random Fields in Stochastic Information Transmission Systems

Before developing the full stochastic EM system theory, we must introduce a technical distinction between two types of medium GFs, which, in the stochastic case, must be treated as separate.

**Definition** **2** (transmitting and scattering stochastic medium Green’s functions)**.**  *For a stochastic medium GF, we distinguish between RFs ${\bar{\bar{G}}}_{t}$ and ${\bar{\bar{G}}}_{s}$, with the primary difference lying in their respective domains:*

(44) $$\begin{array}{cc} \; & {\bar{\bar{G}}}_{b^{\prime }}\left(x,x^{\prime },\omega ,\varpi \right)={\left.\bar{\bar{G}}\left(x,x^{\prime },\omega ,\varpi \right)\right|}_{x^{\prime }\in S_{b^{\prime }}}, \end{array}$$

*where $b^{\prime }\in \left\{t,s\right\}$, while the codomain continues to be $V_{\mathrm{ex}}∋x$.*

Note that in the deterministic analysis of EM system theory presented in Section 3.3, we did not need to distinguish between the two cases where the radiating current points $x^{\prime }$ reside either on the transmitting $S_{t}$ or scattering $S_{s}$ systems. On the other hand, for the stochastic EM system, this distinction becomes crucial. The reason for this necessity will become clear later in Section 4.3, when we perform the KLE analysis of stochastic EM systems. In that case, we will need to differentiate the random variables in the two expansions, depending on whether the source originates from scattering or transmitting systems. For now, we assume that these two RFs are independent (see Definition 3 below).

The following definition encapsulates the most characteristic features of what we intuitively understand as a generic stochastic EM transmission system. As with all other constructions presented in this paper, our primary focus remains on directly leveraging the structure of the EM response function, as it offers a rigorous general input–output description of the problem at hand.

**Definition** **3** (stochastic electromagnetic transmission system)**.**  *Consider an EM transmission system $\left(S_{t},S_{s},S_{r}\right)$ as in Definition 1. Assume that information is injected into the transmitting system $S_{t}$ through an RF information source ${\bar{\psi }}_{\mathrm{in}}\left(x,\omega ,\varpi \right)$. We say that the system $\left(S_{t},S_{s},S_{r}\right)$ is a stochastic EM transmission system when one or all of its EM GFs become RFs, i.e., in the most general case we write*

(45) $$\begin{array}{c} {\bar{\bar{F}}}_{b}={\bar{\bar{F}}}_{b}\left(x,x^{\prime },\omega ,\varpi \right),\;\;{\bar{\bar{G}}}_{b^{\prime }}={\bar{\bar{G}}}_{b^{\prime }}\left(x,x^{\prime },\omega ,\varpi \right), \end{array}$$

*where $b\in \left\{t,s,r\right\},b^{\prime }\in \left\{t,s\right\}.$*

Definition 3 is, in fact, quite broad. In applications to information and communication theories, we anticipate that more restricted forms will be more pertinent to mathematical analysis, particularly in cases where some or all of the EM GFs become statistically dependent. Here, we primarily focus on the scenario where all the stochastic GFs ${\bar{\bar{F}}}_{b},b\in \left\{t,s,r\right\},$ and ${\bar{\bar{G}}}_{b^{\prime }},b^{\prime }\in \left\{t,s\right\}$, in (45), are *stochastically independent*. We argue that this case is of paramount importance and does not represent a loss of generality. The justification for this choice is as in the following two Remarks 3 and 4.

**Remark** **3** (stochastic independence of the EM current response functions)**.**  *First, the transmitter and receiver GFs, ${\bar{\bar{F}}}_{t}$ and ${\bar{\bar{F}}}_{r}$, should exhibit mutually independent intrinsic random fluctuations. This independence is essential in communication theory, where the very concept of transmitting information necessitates that the transmitter and receiver are separate systems, divided or separated by a communication channel. If the transmitter and receiver were strongly coupled, effectively constituting a single system, the transmission of information would offer no real advantage. Regarding the scattering GF ${\bar{\bar{F}}}_{s}$, we assume without loss of generality that it is stochastically independent of both the transmitter and receiver. Otherwise, any part of $S_{s}$ strongly coupled with either the transmitter or receiver can be regrouped with the corresponding system $S_{t}$ or $S_{s}$.*

**Remark** **4** (stochastic independence of the EM medium Green functions)**.**  *The channel (medium) GFs ${\bar{\bar{G}}}_{t}$ and ${\bar{\bar{G}}}_{s}$ are obviously independent of the transmitter and receiver systems, as the very definition of information communication requires this independence. While the channel can be correlated with the scattering system $S_{s}$, in scattering theory the medium GFs ${\bar{\bar{G}}}_{s}$ is typically assumed to be independent of the scattering EM system [44,97]. If part of $S_{s}$ contributes to ${\bar{\bar{G}}}_{b^{\prime }},b^{\prime }\in \left\{t,s\right\}$, we may reassign it as part of ${\bar{\bar{G}}}_{b^{\prime }}$. Finally, that the transmitting and scattering medium GFs ${\bar{\bar{G}}}_{t}$ and ${\bar{\bar{G}}}_{s}$ are assumed to be stochastically independent can be justified on the grounds that the transmitting $S_{t}$ and scattering $S_{s}$ systems are independent (see Remark 3).*

In addition to the stochastic independence of the various random GFs in a given stochastic information transmission system, we also require certain regularity conditions to ensure that the induced RFs exhibit desirable properties such as continuity, integrability, and differentiability. The following definition delineates the most salient properties necessary for these RFs to exhibit well-behaved characteristics. It is intended as a natural generalization of the deterministic EM information transmission system outlined in Definition 1 and Theorem 2.

**Definition** **4** (standard stochastic electromagnetic transmission system)**.**  *Consider a stochastic EM transmission system $\left(S_{t},S_{s},S_{r}\right)$ as defined in Definition 3. We have the following input data:*

*1.*  *Information is injected into the transmitting system $S_{t}$ through an RF information source ${\bar{\psi }}_{\mathrm{in}}\left(x,\omega ,\varpi \right)$ with a mean function, covariance function and pseudo-covariance functions:*
(46) $$\begin{array}{cc} \; & \mu_{{\bar{\psi }}_{\mathrm{in}}}\left(x,\omega \right)=E\left\{{\bar{\psi }}_{\mathrm{in}}\left(x,\omega ,\varpi \right)\right\},\;C_{{\bar{\psi }}_{\mathrm{in}}}\left(x,x^{\prime },\omega \right),\;C_{{\bar{\psi }}_{\mathrm{in}}}^{\prime }\left(x,x^{\prime },\omega \right). \end{array}$$
*2.*  *A complete set of system functions, where all of these GFs are promoted to the status of second-order stochastic RFs, i.e., we have ${\bar{\bar{F}}}_{b},b\in \left\{t,s,r\right\},$ and ${\bar{\bar{G}}}_{b^{\prime }},b^{\prime }\in \left\{t,s\right\}$, as in *(45)*, with the following data:*
  - *A set of EM GFs’ mean functions*
(47) $$\begin{array}{cc} \; & \mu_{{\bar{\bar{F}}}_{b}}\left(x,x^{\prime },\omega \right)=E\left\{{\bar{\bar{F}}}_{b}\left(x,x^{\prime },\omega ,\varpi \right)\right\},\;\mu_{{\bar{\bar{G}}}_{b^{\prime }}}\left(x,x^{\prime },\omega \right)=E\left\{{\bar{\bar{G}}}_{b^{\prime }}\left(x,x^{\prime },\omega ,\varpi \right)\right\}. \end{array}$$
  - *EM GFs’ covariance functions*
(48) $$C_{{\bar{\bar{F}}}_{b}}\left(x,x^{\prime },\omega \right),\;C_{{\bar{\bar{G}}}_{b^{\prime }}}\left(x,x^{\prime },\omega \right).$$
  - *EM GFs’ pseudo-covariance functions*
(49) $$C_{{\bar{\bar{F}}}_{b}}^{\prime }\left(x,x^{\prime },\omega \right),\;C_{{\bar{\bar{G}}}_{b^{\prime }}}^{\prime }\left(x,x^{\prime },\omega \right).$$

*We then say that the stochastic EM information transmission system $\left(S_{t},S_{s},S_{r}\right)$ is a stochastic EM transmission system in the standard form when the following two additional conditions are satisfied for all $\omega \in R^{+}$:*

*1.*  *The five EM GF RFs ${\bar{\bar{F}}}_{b},b\in \left\{t,s,r\right\},$ and ${\bar{\bar{G}}}_{b^{\prime }},b^{\prime }\in \left\{t,s\right\}$ are mutually stochastically independent.*
*2.*  *The covariance/pseudo-covariance functions satisfy the following spatial continuity conditions:*
  - *The current GF covariance and pseudo-covariance functions*
(50) $$\begin{array}{c} C_{{\bar{\bar{F}}}_{b}}:S_{b}\times S_{b}\times R^{+}\to C,\\ C_{{\bar{\bar{F}}}_{b}}^{\prime }:S_{b}\times S_{b}\times R^{+}\to C, \end{array}$$
    *are continuous over $\left(x,x^{\prime }\right)\in S_{b}\times S_{b}$ for b∈{t,s,r}.*
  - *The medium GF covariance and pseudo-covariance functions*
(51) $$\begin{array}{c} C_{{\bar{\bar{G}}}_{b^{\prime }}}:V_{\mathrm{ex}}\times S_{b^{\prime }}\times R^{+}\to C,\\ C_{{\bar{\bar{G}}}_{b^{\prime }}}^{\prime }:V_{\mathrm{ex}}\times S_{b^{\prime }}\times R^{+}\to C, \end{array}$$
    *are continuous over $\left(x,x^{\prime }\right)\in V_{\mathrm{ex}}\times S_{b^{\prime }}$ for $b^{\prime }\in \left\{t,s\right\}$.*

Recall that, as established in Definition 4 and consistently throughout this paper, all complex-valued covariance and pseudo-covariance functions adhere to the convention outlined in Definition A10.

The following proposition demonstrates that the regularity condition assumed by Definition 4—specifically, the continuity of the mean, covariance, and pseudo-covariance functions of the input information field—is sufficiently robust to guarantee that all relevant RFs characterizing fixed aspects of the system (input information and GF RFs) exhibit m.s. continuity.

**Proposition** **1.** *In a standard stochastic EM transmission system (see Definition 4), the input information RF $\psi_{\mathrm{in}}\left(x,\omega ,\varpi \right)$ and the stochastic GFs ${\bar{\bar{F}}}_{b}\left(x,x^{\prime },\omega ,\varpi \right)$ for b∈{t,s,r} and ${\bar{\bar{G}}}_{b^{\prime }}\left(x,x^{\prime },\omega ,\varpi \right)$ for $b^{\prime }\in \left\{t,s\right\}$ are all m.s. continuous with respect to x and $x^{\prime }$ over their respective domains. The specific domains for these functions are detailed in Table 3.*

**Proof.**  See Appendix L.    □

It is worth noting that the condition of continuity of the mean, covariance, and pseudo-covariance functions is sufficiently strong to ensure that, for a reasonably broad class of response functions, the *response* RF—calculated through a generic superposition integral such as (A89)—also possesses continuous mean, covariance, and pseudo-covariance functions. This significant fact is encapsulated in Proposition A6 and is used to prove the key Theorem 3.

From this point forward, we will always assume we are operating with a stochastic EM system as specified in Definition 4. In this scenario, while information will be modeled by the input RF ${\bar{\psi }}_{\mathrm{in}}\left(x,\omega ,\varpi \right)$, the EM system itself may introduce its own randomness, such as fluctuations and noise. The main “output” RF, resulting from the interaction of the injected information field ${\bar{\psi }}_{\mathrm{in}}\left(x,\omega ,\varpi \right)$ with the transmitter $S_{t}$, scattering system $S_{s}$, and receiver $S_{r}$, will be the received current ${\bar{J}}_{r}\left(x,\omega ,\varpi \right)$.

The following theorem rigorously establishes that in standard stochastic EM information systems, as specified in Definition 4, all induced currents, as well as the radiation and scattered fields, are indeed RFs. This theorem can be interpreted as a stochastic generalization of Theorem 2.

**Theorem** **3.** *Consider a standard stochastic EM information system as specified in Definition 4 where information is injected through an input RF ${\bar{\psi }}_{\mathrm{in}}\left(x,\omega ,\varpi \right)$. The response RFs, denoted generically as $\bar{R}\left(x,\omega ,\varpi \right)\in Q$ (see Table 4), are the induced currents ${\bar{J}}_{b}\left(x,\omega ,\varpi \right)$ for $x\in S_{b},b\in \left\{t,s,r\right\}$, the radiation and scattered fields ${\bar{\psi }}_{\mathrm{rad}}\left(x,\omega ,\varpi \right)$ and ${\bar{\psi }}_{s}\left(x,\omega ,\varpi \right)$ for $x\in V_{\mathrm{ex}}$. Then we have the following:*

*1.*  *All response fields become RFs $\bar{R}\left(x,\omega ,\varpi \right)$ on their respective domains.*
*2.*  *All of the above RFs have spatially continuous mean, covariance, and pseudo-covariance functions on their respective domains.*

**Proof.**  See Appendix M.    □

The primary structure addressed in Theorem 3 is summarized in the three processes (52), (53), and (54). These processes represent the stochastic generalization of the corresponding deterministic processes (41), (42), and (43), respectively. The key distinction lies in the fact that, in this context, the EM system itself is treated as stochastic. Mathematically, this is accomplished in our framework by promoting the EM GFs to RFs, a dependency we explicitly highlight by indicating the dependence of an RF on ϖ.

Stochastic LoS Process:

(52) $$\begin{array}{c} {\bar{\psi }}_{\mathrm{in}}\left(x,\omega ,\varpi \right)\overset{{\bar{\bar{F}}}_{t}\left(x,x^{\prime },\omega ,\varpi \right)}{\to }{\bar{J}}_{t}\left(x,\omega ,\varpi \right)\overset{{\bar{\bar{G}}}_{t}\left(x,x^{\prime },\omega ,\varpi \right)}{\to }{\bar{\psi }}_{\mathrm{rad}}\left(x,\omega ,\varpi \right) \end{array}$$

Stochastic NLoS Process:

(53) $$\begin{array}{c} {\bar{\psi }}_{\mathrm{in}}\left(x,\omega ,\varpi \right)\overset{{\bar{\bar{F}}}_{t}\left(x,x^{\prime },\omega ,\varpi \right)}{\to }{\bar{J}}_{t}\left(x,\omega ,\varpi \right)\overset{{\bar{\bar{G}}}_{t}\left(x,x^{\prime },\omega ,\varpi \right)}{\to }{\bar{\psi }}_{\mathrm{rad}}\left(x,\omega ,\varpi \right)\overset{{\bar{\bar{F}}}_{s}\left(x,x^{\prime },\omega ,\varpi \right)}{\to }{\bar{J}}_{s}\left(x,\omega ,\varpi \right)\overset{{\bar{\bar{G}}}_{s}\left(x,x^{\prime },\omega ,\varpi \right)}{\to }{\bar{\psi }}_{s}\left(x,\omega ,\varpi \right) \end{array}$$

Stochastic Receiver Interaction Process:

(54) $$\begin{array}{c} {\bar{\psi }}_{\mathrm{rad}}\left(x,\omega ,\varpi \right)+{\bar{\psi }}_{s}\left(x,\omega ,\varpi \right)\overset{{\bar{\bar{F}}}_{r}\left(x,x^{\prime },\omega ,\varpi \right)}{\to }{\bar{J}}_{r}\left(x,\omega ,\varpi \right)={\bar{J}}_{r}^{\mathrm{los}}\left(x,\omega ,\varpi \right)+{\bar{J}}_{r}^{\mathrm{nlos}}\left(x,\omega ,\varpi \right) \end{array}$$

### 3.5. Summary of Regularity Conditions for Electromagnetic Random Field Construction

To summarize the foundational assumptions underlying our EM-RFT, we consolidate all regularity conditions required by Theorems 2 and 3 into two summary tables. Importantly, our framework imposes *no assumption of spatial stationarity*, *no requirement of Gaussianity*, and *no restriction to proper (circular) complex RFs*; the theory accommodates arbitrary second-order statistics, including potentially improper (noncircular) RFs characterized by nonzero pseudo-covariance. Table 6 details the deterministic case, explicitly distinguishing the singularity structure of the medium Green’s functions $\bar{\bar{G}}$—which are singular only at coincident field and source points $x=x^{\prime }$—from the everywhere-nonsingular current Green’s functions ${\bar{\bar{F}}}_{b}$, whose continuity (including on the diagonal $x=x^{\prime }$) follows from Theorem A7. These conditions together guarantee mean-square integrability and continuity of the induced response fields. Table 7 extends this summary to the standard stochastic system (Definition 4), incorporating the critical assumption of mutual stochastic independence among the five Green functions and the continuity of their second-order moments. This unified presentation resolves implicit assumptions in the original proofs and provides a transparent checklist for the well-posedness of EM random field constructions under both deterministic and stochastic regimes, without imposing restrictive statistical or structural constraints such as stationarity, Gaussianity, or properness.

### 3.6. Additional Remarks on Stochastic Electromagnetic LoS and NLoS Green’s (Response) Functions

To facilitate computations and reveal more fundamental response functions (i.e., GFs) relevant to EIT, we introduce the following RF GFs (response functions). First, the *line-of-sight (LoS) stochastic system function* is defined as

(55) $$\begin{array}{c} {\bar{\bar{S}}}_{\mathrm{los}}\left(x,x_{1},x_{4},x_{5},\omega ,\varpi \right):={\bar{\bar{F}}}_{r}\left(x,x_{1},\omega ,\varpi \right)\cdot {\bar{\bar{G}}}_{t}\left(x_{1},x_{4},\omega ,\varpi \right)\cdot {\bar{\bar{F}}}_{t}\left(x_{4},x_{5},\omega ,\varpi \right). \end{array}$$

Second, *the non-line-of-sight (NLoS) stochastic response function* is defined by the formula

(56) $$\begin{array}{cc} {\bar{\bar{S}}}_{\mathrm{nlos}} & \left(x,x_{1},x_{2},x_{3},x_{4},x_{5},\omega ,\varpi \right):={\bar{\bar{F}}}_{r}\left(x,x_{1},\omega ,\varpi \right)\cdot {\bar{\bar{G}}}_{s}\left(x_{1},x_{2},\omega ,\varpi \right)\cdot {\bar{\bar{F}}}_{s}\left(x_{2},x_{3},\omega ,\varpi \right)\cdot {\bar{\bar{G}}}_{t}\left(x_{3},x_{4},\omega ,\varpi \right)\cdot {\bar{\bar{F}}}_{t}\left(x_{4},x_{5},\omega ,\varpi \right). \end{array}$$

In terms of these two response functions we may express the total LoS of the receive current RF as

(57) $$\begin{array}{cc} {\bar{J}}_{r}^{\mathrm{los}}\left(x,\omega ,\varpi \right) & =\int_{S_{r}}d^{2}x_{1}\int_{S_{t}}d^{2}x_{4}\int_{S_{t}}d^{2}x_{5}\;{\bar{\bar{S}}}_{\mathrm{los}}\left(x,x_{1},x_{4},x_{5},\omega ,\varpi \right)\cdot {\bar{\psi }}_{\mathrm{in}}\left(x_{5},\omega ,\varpi \right), \end{array}$$

while the total NLoS components is given by

(58) $$\begin{array}{cc} {\bar{J}}_{r}^{\mathrm{nlos}}(x & ,\omega ,\varpi )=\int_{S_{r}}d^{2}x_{1}\int_{S_{s}}d^{2}x_{2}\int_{S_{s}}d^{2}x_{3}\int_{S_{t}}d^{2}x_{4}\int_{S_{t}}d^{2}x_{5}\;{\bar{\bar{S}}}_{\mathrm{nlos}}\left(x,x_{1},x_{2},x_{3},x_{4},x_{5},\omega ,\varpi \right)\cdot {\bar{\psi }}_{\mathrm{in}}\left(x_{5},\omega ,\varpi \right). \end{array}$$

The relations (57) and (58) follow directly from Theorem 1 and the definitions provided in (55) and (56).

The response functions ${\bar{\bar{S}}}_{\mathrm{los}}$ and ${\bar{\bar{S}}}_{\mathrm{nlos}}$ have intuitive physical interpretations. The LoS stochastic system function is a 4-point GF that links the input information field ${\bar{\psi }}_{\mathrm{in}}$ to the received RF current distribution ${\bar{J}}_{r}$ via the radiation GF $\bar{\bar{G}}$. In contrast, the NLoS stochastic GF is a 6-point GF that also connects ${\bar{\psi }}_{\mathrm{in}}$ to ${\bar{J}}_{r}$, but through the full EM interaction with the scattering system $S_{s}$. The distinction between the 4-point structure of ${\bar{\bar{S}}}_{\mathrm{los}}$ and the more complex 6-point form of ${\bar{\bar{S}}}_{\mathrm{nlos}}$ is evident from Figure 1 and Figure 3.

The inherently stochastic nature of the LoS and NLoS response functions, ${\bar{\bar{S}}}_{\mathrm{los}}$ and ${\bar{\bar{S}}}_{\mathrm{nlos}}$, is explicitly highlighted in the defining relations (55) and (56), underscoring the broad generality of the formulation presented. Each factor in the definitions of these generalized response functions—especially the fundamental response functions ${\bar{\bar{F}}}_{b}$ and ${\bar{\bar{G}}}_{b^{\prime }}$, with b∈{t,s,r} and $b^{\prime }\in \left\{t,s\right\}$—can independently become an RF (a random superdyad of the appropriate type). In practice, various physical models contribute to any given stochastic model. For example, environmental fluctuations may induce randomness in ${\bar{\bar{G}}}_{b^{\prime }}$, uncertainties in the shape or boundary conditions of the scattering cluster may cause randomness in ${\bar{\bar{F}}}_{s}$, and incomplete knowledge of the details of the transmit/receiving system—such as in random networks or other modern wireless communication scenarios—could imply that ${\bar{\bar{F}}}_{t}$ and/or ${\bar{\bar{F}}}_{r}$ themselves might become random superdyads.

### 3.7. The Stochastic Correlation Propagator

We established that the surface current distribution induced in the system $S_{b},b\in \left\{t,s,r\right\},$ under the influence of an external field excitation is represented by the RF ${\bar{J}}_{b}\left(x,\omega ,\varpi \right)$, which is a 2×1 array of surface vector fields (see Table 3 and Appendix C). This supervector can ultimately be expressed as a 4×1 complex array [see Equation (A31)]. Consequently, the correlation information of the current necessarily involves a 4×4 matrix. This observation motivates the following definition for the correlation of RFs composed of arrays of vectors (supervectors) of the form ${\bar{J}}_{b}$.

**Definition** **5.** *Consider a stochastic EM transmission system $\left(S_{t},S_{s},S_{r}\right)$ as in Definition 3. Assume that information is injected into the transmitting system $S_{t}$ through an excitation field ${\bar{\psi }}_{\mathrm{in}}\left(x,\omega ,\varpi \right)$. We can extract the individual components of the current distribution in each one of the three systems $S_{t},S_{s},S_{r}$ as follows. Define the ith component of the current supervector $\bar{J_{b}},b\in \left\{t,s,r\right\},$ as the quantity*

(59) $$J_{i}^{b}\left(x,\omega \right):={\bar{a}}_{i}^{⊤}\left(x\right)\cdot {\bar{J}}_{b}\left(x,\omega ,\varpi \right),$$

*where the four direction real vector fields ${\bar{a}}_{i}\left(x\right),i=1,2,3,4,$ are defined as^14^*

(60) $${\bar{a}}_{1}\left(x\right):=\left[\begin{array}{c} {\hat{\alpha }}_{1}\left(x\right)\\ 0 \end{array}\right],\;{\bar{a}}_{2}\left(x\right):=\left[\begin{array}{c} {\hat{\alpha }}_{2}\left(x\right)\\ 0 \end{array}\right],\;{\bar{a}}_{3}\left(x\right):=\left[\begin{array}{c} 0\\ {\hat{\alpha }}_{1}\left(x\right) \end{array}\right],\;{\bar{a}}_{4}\left(x\right):=\left[\begin{array}{c} 0\\ {\hat{\alpha }}_{2}\left(x\right) \end{array}\right].$$

*Then the ijth element of the correlation matrix of the random vector field ${\bar{J}}_{b}$, i.e., the correlation between the ith and jth components of ${\bar{J}}_{b}$, i,j=1,2,3,4, is defined as*

(61) $$\begin{array}{cc} R_{ij}^{b} & \left(x,x^{\prime },\omega \right):=E\left\{J_{i}^{b}\left(x,\omega \;\varpi \right)J_{j}^{b*}\left(x^{\prime },\omega \;\varpi \right)\right\}=E\left\{{\bar{J}}_{b}^{†}\left(x^{\prime },\omega \;\varpi \right)\cdot {\bar{a}}_{j}\left(x^{\prime }\right)\;{\bar{a}}_{i}{\left(x\right)}^{†}\cdot {\bar{J}}_{b}\left(x,\omega \;\varpi \right)\right\}. \end{array}$$

*The ijth element of the pseudo-correlation matrix, on the other hand, is defined as*

(62) $$\begin{array}{cc} R_{ij}^{\prime b} & \left(x,x^{\prime },\omega \right):=E\left\{J_{i}^{b}\left(x,\omega ,\varpi \right)J_{j}^{b}\left(x^{\prime },\omega ,\varpi \right)\right\}=E\left\{{\bar{J}}_{b}^{⊤}\left(x^{\prime },\omega ,\varpi \right)\cdot {\bar{a}}_{j}\left(x^{\prime }\right)\;{\bar{a}}_{i}^{⊤}\left(x\right)\cdot {\bar{J}}_{b}\left(x,\omega ,\varpi \right)\right\}. \end{array}$$

**Remark** **5.** *Note that the quantities ${\bar{a}}_{j}\left(x^{\prime }\right)\;{\bar{a}}_{i}^{⊤}\left(x\right)$ represents a 2×2 dyad whose general elements are of the form ${\hat{\alpha }}_{l}{\hat{\alpha }}_{l^{\prime }},l,l^{\prime }\in \left\{1,2\right\}$, or zero. For example, one of the 16 possible combinations of ${\bar{a}}_{j}\left(x^{\prime }\right)\;{\bar{a}}_{i}^{⊤}\left(x\right)$ reads as*

(63) $${\bar{a}}_{1}\left(x^{\prime }\right)\;{\bar{a}}_{2}^{⊤}\left(x\right)=\left[\begin{array}{cc} {\hat{\alpha }}_{1}\left(x\right){\hat{\alpha }}_{2}\left(x^{\prime }\right) & 0\\ 0 & 0 \end{array}\right].$$

*After that, the quadratic form in the RHS of Equations *(61)* and *(62)* can be computed as usual according to superdyad–supervector multiplication rules (see Appendix A). Alternatively, one may also utilize the dyadic identity $\left(\bar{A}\bar{B}\right)\cdot \bar{C}=\bar{A}\left(\bar{B}\cdot \bar{C}\right)$ to directly compute the same quadratic form as multiplication of the ith and jth supervector components.*

For the relation between correlation/pseudo-correlation from one side, and covariance/ pseudo-covariance, see Remark A3. In general, since knowledge of the mean functions make correlation and covariance properties equivalent, in this section we focus on correlation for brevity. Also, see Remark A4 for formulas for covariance/correlation of the most important key EM RFs we work with in this paper. Note that the convention introduced in Definition A10 has been applied, allowing all correlation matrices of complex vector representations to be written without the superscript “(c).”

We now turn our attention to the primary induced current in the information transmission system, the final received current, denoted as ${\bar{J}}_{r}\left(x,\omega ,\varpi \right)$. This quantity is central to communication system applications, as it serves to extract information from the system upon injecting a specified input field, ${\bar{\psi }}_{\mathrm{in}}\left(x,\omega ,\varpi \right)$. The theorem below encapsulates one of the essential results of second-order EM-RFT, providing a comprehensive characterization of how the mean, covariance, and pseudo-covariance of an input flow are transformed as the EM field propagates through the system. Notably, the theorem expresses the output data solely in terms of EM response functions.

**Theorem** **4.** *Consider a stochastic EM transmission system $\left(S_{t},S_{s},S_{r}\right)$ and assume that information is injected into the transmitting system $S_{t}$ through an excitation field ${\bar{\psi }}_{\mathrm{in}}\left(x,\omega ,\varpi \right)$ as in Definition 3. Then the received current RF ${\bar{J}}_{r}\left(x,\omega ,\varpi \right)$ mean, covariance, and pseudo-covariance can be computed as follows:*

*1.*  *The mean function can be computed as*
(64) $$\begin{array}{cc} E\left\{{\bar{J}}_{r}\left(x,\omega ,\varpi \right)\right\}=\int_{S_{t}} & d^{2}x^{\prime }\;\bar{\bar{B}}\left(x,x^{\prime },\omega ,\varpi \right)\cdot \;E\left\{{\bar{\psi }}_{\mathrm{in}}\left(x^{\prime },\omega ,\varpi \right)\right\}, \end{array}$$
  *where $\bar{\bar{B}}$ is given by *(35)*.*
*2.*  *The matrix elements of $R_{{\bar{J}}_{r}}$ can be computed via the expression*
(65) $$\begin{array}{c} R_{{\bar{J}}_{r}}^{ij}\left(x,x^{\prime },\omega \right)=R_{{\bar{J}}_{r}}^{ij,\mathrm{nlos}}\left(x,x^{\prime },\omega \right)+R_{{\bar{J}}_{r}}^{ij,\mathrm{los}}\left(x,x^{\prime },\omega \right)+R_{{\bar{J}}_{r}}^{ij,\mathrm{los}/\mathrm{nlos}}\left(x,x^{\prime },\omega \right)+R_{{\bar{J}}_{r}}^{ij,\mathrm{nlos}/\mathrm{los}}\left(x,x^{\prime },\omega \right), \end{array}$$
  *where $R_{{\bar{J}}_{r}}^{ij,\mathrm{nlos}}$, $R_{{\bar{J}}_{r}}^{ij,\mathrm{los}}$, $R_{{\bar{J}}_{r}}^{ij,\mathrm{los}/\mathrm{nlos}}$, and $R_{{\bar{J}}_{r}}^{ij,\mathrm{nlos}/\mathrm{los}}$ are given by *(A116)*, *(A118)*, *(A120)*, and *(A130)*, respectively.*
*3.*  *For the pseudo-matrix $R_{{\bar{J}}_{r}}^{\prime }$, we have*
(66) $$R_{{\bar{J}}_{r}}^{\prime \;ij}\left(x,x^{\prime },\omega \right)=R_{{\bar{J}}_{r}}^{\prime \;ij,\mathrm{nlos}}\left(x,x^{\prime },\omega \right)+R_{{\bar{J}}_{r}}^{\prime \;ij,\mathrm{los}}\left(x,x^{\prime },\omega \right)+R_{{\bar{J}}_{r}}^{\prime \;ij,\mathrm{los}/\mathrm{nlos}}\left(x,x^{\prime },\omega \right)+R_{{\bar{J}}_{r}}^{\prime \;ij,\mathrm{nlos}/\mathrm{los}}\left(x,x^{\prime },\omega \right).$$
  *where $R_{{\bar{J}}_{r}}^{\prime \;ij,\mathrm{nlos}}$, $R_{{\bar{J}}_{r}}^{\prime \;ij,\mathrm{los}}$, $R_{{\bar{J}}_{r}}^{\prime \;ij,\mathrm{los}/\mathrm{nlos}}$, and $R_{{\bar{J}}_{r}}^{\prime \;ij,\mathrm{nlos}/\mathrm{los}}$ are given by expressions identical to *(A116)*, *(A118)*, *(A120)*, and *(A130*), respectively, with the following two modifications:*
  - *The hermitian operation *†* is replaced by the transpose operation *⊤*.*
  - *The input information field ${\bar{\psi }}_{\mathrm{in}}$ superdyadic correlation ${\bar{\bar{R}}}_{{\bar{\psi }}_{\mathrm{in}}}$ is replaced by the pseudo-correlation ${\bar{\bar{R}}}_{{\bar{\psi }}_{\mathrm{in}}}^{\prime }$ defined by *(A78)*.*

**Proof.**  See Appendix N.    □

**Figure 5:**
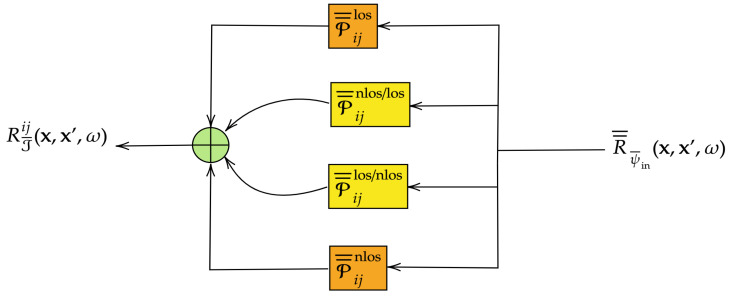
An illustration of the general mathematical structure governing the propagation of stochastic correlation from the input to the output in a general stochastic EM information transmission system, as established by Theorem 4. Here i,j∈{1,2,3,4}. The input data are the superdyadic correlation matrix ${\bar{\bar{R}}}_{{\bar{\psi }}_{\mathrm{in}}}$ defined by (A78). A closely analogous diagram can also be constructed to represent the mathematical framework underlying the production of the pseudo-correlation matrix $R_{{\bar{J}}_{r}}^{\prime }\left(x,x^{\prime },\omega \right)$ in response to the input pseudo-correlation superdyad ${\bar{\bar{R}}}_{{\bar{\psi }}_{\mathrm{in}}}^{\prime }$ but is omitted here for brevity.

The overall structure of input–output decoupling in the propagation of correlation throughout a generic stochastic EM information transmission system is illustrated in Figure 5. Building on this framework, we can make the following general observations regarding the structure of correlation propagation in such systems, as detailed in Theorem 4:

1. The input correlation matrix needed to compute the output second-order statistics are not the 4×4 correlation matrices $R_{{\bar{\psi }}_{\mathrm{in}}}\left(x,x^{\prime },\omega \right)$ and $R_{{\bar{\psi }}_{\mathrm{in}}}^{\prime }\left(x,x^{\prime },\omega \right)$, but the dyadic-valued 2×2 matrices or superdyads ${\bar{\bar{R}}}_{{\bar{\psi }}_{\mathrm{in}}}\left(x,x^{\prime },\omega \right)$ and ${\bar{\bar{R}}}_{{\bar{\psi }}_{\mathrm{in}}}^{\prime }\left(x,x^{\prime },\omega \right)$ defined by (A78).
2. The *entire* 2×2 correlation superdyad ${\bar{\bar{R}}}_{{\bar{\psi }}_{\mathrm{in}}}\left(x,x^{\prime },\omega \right)$ is required to compute a single element, the *ij*th element, of the output 4×4 correlation $R_{{\bar{J}}_{r}}\left(x,x^{\prime },\omega \right)$. An analogous statement applies to the relation between the pseudo-correlation superdyad ${\bar{\bar{R}}}_{{\bar{\psi }}_{\mathrm{in}}}^{\prime }\left(x,x^{\prime },\omega \right)$ and the output pseudo-correlation matrix $R_{{\bar{J}}_{r}}^{\prime }\left(x,x^{\prime },\omega \right)$.
3. In Figure 5, each box represents the linear transformation associated with the propagator within it. These mathematical operations are explicitly defined by the relations (A117), (A119), (A121), and (A131). A structurally analogous diagram could be constructed to illustrate the production of the pseudo-correlation matrix $R_{{\bar{J}}_{r}}^{\prime }\left(x,x^{\prime },\omega \right)$; however, it is omitted here for brevity.
4. Note that the eight propagators comprising the set
  (67) $$C:=\left\{{\bar{\bar{P}}}_{ij}^{\mathrm{los}},\;{\bar{\bar{P^{\prime }}}}_{ij}^{\mathrm{los}},\;{\bar{\bar{P}}}_{ij}^{\mathrm{nlos}},\;{\bar{\bar{P^{\prime }}}}_{ij}^{\mathrm{nlos}},\;{\bar{\bar{P}}}_{ij}^{\mathrm{los}/\mathrm{nlos}},\;{\bar{\bar{P^{\prime }}}}_{ij}^{\mathrm{los}/\mathrm{nlos}},\;{\bar{\bar{P}}}_{ij}^{\mathrm{nlos}/\mathrm{los}},\;{\bar{\bar{P^{\prime }}}}_{ij}^{\mathrm{nlos}/\mathrm{los}}\right\},$$
  are themselves derived from the LoS and NLoS system response functions ${\bar{\bar{S}}}^{\mathrm{los}}$ and ${\bar{\bar{S}}}^{\mathrm{nlos}}$, as specified in formulas (A117), (A119), (A121), and (A131). The only distinction between a correlation propagator $\bar{\bar{P}}$ and a pseudo-correlation propagator $\bar{\bar{P^{\prime }}}$ is that the latter employs the transpose operation *⊤* in place of the Hermitian operation † in the corresponding expressions.
5. According to Theorem 4, the total correlation and pseudo-correlation *ij*th matrix element comprises four fundamental types of contributions: LoS/LoS, NLoS/NLoS, LoS/NLoS, and NLoS/LoS. For instance, the LoS/LoS term quantifies the correlation between the LoS *i*th component of the received current and the LoS *j*th component. The NLoS/LoS contribution captures the net correlation between the NLoS *i*th component and the LoS *j*th component. The remaining contributions follow analogous interpretations.
6. Unlike the system response function analyzed in Theorem 1, correlation is inherently a second-order phenomenon. Consequently, it involves cross-interactions between the LoS and NLoS components. Note that Theorem 1 and the associated system response functions (55) and (56) describe first-order quantities, offering information about the trend (mean or average value) of the RFs. Thus, the structure of the correlation problem is intrinsically more complex. This complexity accounts for the necessity of eight distinct propagators (enumerated in Table 5) to comprehensively characterize the propagation and dynamics of correlation within the EM information transmission system.

According to Table 5, addressing the general correlation problem necessitates the use of eight distinct response functions, termed correlation propagators. Each propagator is a superdyad, represented as an array of complex dyadic GFs, and is further indexed by a pair of indices i,j∈{1,2,3,4}. Consequently, the total number of superdyad propagators required is 8×16, yielding a substantial 128 propagators. This considerable complexity is both intrinsic and universal, reflecting the fundamental nature of the correlation problem within the EM information transmission system and its inherently multifaceted structure. The advantages gained by working with these 128 propagators can be summarized as follows:

1. The information flow problem achieves a complete decoupling into two primary components: the *input* field (superdyadic) correlation and pseudo-correlation matrices, ${\bar{\bar{R}}}_{{\bar{\psi }}_{\mathrm{in}}}\left(x,x^{\prime },\omega \right)$ and ${\bar{\bar{R}}}_{{\bar{\psi }}_{\mathrm{in}}}^{\prime }\left(x,x^{\prime },\omega \right)$, and the *output* current–current correlation and pseudo-correlation matrices, $R_{{\bar{J}}_{r}}\left(x,x^{\prime },\omega \right)$ and $R_{{\bar{J}}_{r}}^{\prime }\left(x,x^{\prime },\omega \right)$.
2. The output correlation/pseudo-correlation characteristics are determined in terms of the input information source’s correlation and pseudo-correlation properties through convolution-type integrations, a hallmark of the response function methodology in signal and information theories. Specifically, input correlation/pseudo-correlation data propagate from the system’s input terminal, $S_{t}$, to its output terminus, $S_{r}$, traversing all possible interactions mediated by the radiation and scattering channels.
3. All intermediate EM interactions responsible for generating or modifying an existing correlation/pseudo-correlation structure are thoroughly detailed through the explicit knowledge of the various multiple (nested) surface integrals that appear in the propagator set C expressions (see Appendix N).
4. The 128 correlation propagators listed in Table 5 serve as the mathematical apparatus enabling the algorithmic computation of the net transfer of correlation. These propagators encapsulate comprehensive and precise information on how each LoS and NLoS component interacts with others and detail the contributions of the transmitting, scattering, and receiving surfaces to the overall correlation dynamics of the system.

We should note, however, that the eight types of propagators listed in Table 5, amounting to a total of 128 superdyads, present the *maximal* structure of the correlation propagation processes with the presence of scattering objects between the transmitter and receiver. On the other hand, it is possible to derive customized or specialized propagators based on these 128 propagators, similar to how we previously introduced the reduced LoS and NLoS response functions ${\bar{\bar{B}}}^{\mathrm{los}}$ and ${\bar{\bar{B}}}^{\mathrm{nlos}}$ [see Equations (32), (33) and (35)]. This approach involves applying averaging operations to the more complex and fundamental system response functions ${\bar{\bar{S}}}^{\mathrm{los}}$ and ${\bar{\bar{S}}}^{\mathrm{nlos}}$ [see Equations (55) and (56)]. The following theorem provide an illustration of such simplification.

**Theorem** **5** (Reduced Propagation Kernel)**.**  *Consider the the setting described in Theorem 4. Then the ijth elements of the correlation and pseudo-correlation matrices of the induced receive current are given by*

(68) $$\begin{array}{cc} R_{{\bar{J}}_{r}}^{ij}\left(x,x^{\prime },\omega \right) & =\mathrm{Tr}\int_{S_{t}}d^{2}x^{\prime \prime }\int_{S_{t}}d^{2}x^{\prime \prime \prime }\;{\bar{\bar{K}}}_{ij}\left(x,x^{\prime },x^{\prime \prime },x^{\prime \prime \prime },\omega \right)\cdot {\bar{\bar{R}}}_{{\bar{\psi }}_{\mathrm{in}}}\left(x^{\prime \prime },x^{\prime \prime \prime },\omega \right),\\ R_{{\bar{J}}_{r}}^{\prime ij}\left(x,x^{\prime },\omega \right) & =\mathrm{Tr}\int_{S_{t}}d^{2}x^{\prime \prime }\int_{S_{t}}d^{2}x^{\prime \prime \prime }\;{\bar{\bar{K}}}_{ij}^{\prime }\left(x,x^{\prime },x^{\prime \prime },x^{\prime \prime \prime },\omega \right)\cdot {\bar{\bar{R}}}_{{\bar{\psi }}_{\mathrm{in}}}^{\prime }\left(x^{\prime \prime },x^{\prime \prime \prime },\omega \right), \end{array}$$

*where i,j∈{1,2,3,4} and ${\bar{\bar{K}}}_{ij}\left(x,x^{\prime },x^{\prime \prime },x^{\prime \prime \prime },\omega \right)$ is the reduced total correlation propagator of the EM information system under consideration, given by the expression *(A132)*. The kernel ${\bar{\bar{K}}}_{ij}^{\prime }$ propagates the input pseudo-correlation to produce the output pseudo-correlation. The expressions for ${\bar{\bar{K}}}_{ij}^{\prime }$ are the same as those for ${\bar{\bar{K}}}_{ij}$, but with Hermitian operations *†* replaced by transpose operations *⊤* (see Appendix O). The trace operator *Tr* is defined by Formula *(A112)*.*

**Proof.**  See Appendix O.    □

The reduced response functions ${\bar{\bar{B}}}^{\mathrm{los}}$ and ${\bar{\bar{B}}}^{\mathrm{nlos}}$ represent effective two-point Green’s functions that encapsulate the complete electromagnetic cascade from the transmit surface $S_{t}$ to the receive surface $S_{r}$, including all mutual coupling, boundary interactions, and scattering effects. Specifically, ${\bar{\bar{B}}}^{\mathrm{los}}$ models the direct (line-of-sight) path as a composition of transmitter, free-space, and receiver Green’s operators, while ${\bar{\bar{B}}}^{\mathrm{nlos}}$ incorporates an additional scattering stage via the intermediary surface $S_{s}$. On the other hand, the reduced propagators ${\bar{\bar{K}}}_{ij}$ are second-order statistical kernels that map the input field correlation (or pseudo-correlation) to the output current correlation at the receiver. They inherit the full physics of the underlying stochastic Green’s functions but operate on the reduced input–output manifold ($S_{t}\times S_{t}\to S_{r}\times S_{r}$), thereby enabling efficient computation and clear interpretation of spatial correlation in MIMO systems.

Theorem 5 indicates that one can bypass the need to handle the eight different types of propagators for LoS and NLoS correlation and pseudo-correlation when the primary interest lies in evaluating the total correlation and pseudo-correlation. This approach circumvents the necessity of dissecting the contributions of the scattering and receiving subsystems to each specific type of LoS and NLoS interaction. Specifically, the expression (68), along with its counterpart for pseudo-correlation, elucidates the generation of total correlation and pseudo-correlation by each pair of points $\left(x^{\prime \prime },x^{\prime \prime \prime }\right)\in S_{t}\times S_{t}$. Integrating over all such pairs across the entire transmitting system surface $S_{t}$ allows for the derivation of all potential pairs of current–current correlation and pseudo-correlation $\left(x,x^{\prime }\right)\in S_{r}\times S_{r}$ at the receiver side.

It is important to emphasize that this formulation comprehensively incorporates scattering effects and all EM mutual coupling processes. Although scattering data are not explicitly presented in expressions like (68), this information is embedded within the correlation/pseudo-correlation propagator kernels ${\bar{\bar{K}}}_{ij}\left(x,x^{\prime },x^{\prime \prime },x^{\prime \prime \prime },\omega \right)$ and ${\bar{\bar{K^{\prime }}}}_{ij}\left(x,x^{\prime },x^{\prime \prime },x^{\prime \prime \prime },\omega \right)$. This is evident from expressions such as (A126)–(A132), and others. The EM response function remains exact and rigorous, with no approximations introduced in the proof of Theorem 5.

**Figure 6:**
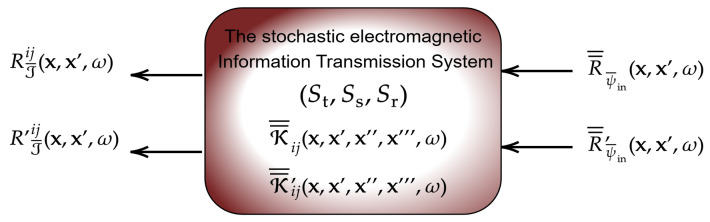
The general structure of the input–output decoupling of correlation propagation in a generic stochastic $\left(S_{t},S_{s},S_{r}\right)$ EM communication system, encompassing the transmitting, scattering, and receiving subsystems. The reduced kernel propagator will transfer the correlation/pseudo-correlation data of the input field to the corresponding correlation/pseudo-correlation data of the output, which, in this context, is defined as the induced current ${\bar{J}}_{r}$ on the receiving system $S_{r}$. Compare also with Figure 5.

To recap, the general structure of the simplified correlation/pseudo-correlation problem is illustrated by the block diagram in Figure 6, which aims to illustrate the decoupling achieved through the trace formulation used in Theorems 4 and 5. Specifically, the results obtained in the latter theorem suggest that the entire stochastic EM information system $\left(S_{t},S_{s},S_{r}\right)$ can be represented as an input–output problem. In this framework, the input is represented by the correlation/pseudo-correlation matrix of the input (source) information field ${\bar{\psi }}_{\mathrm{in}}$, denoted by ${\bar{\bar{R}}}_{{\bar{\psi }}_{\mathrm{in}}}$ and ${\bar{\bar{R}}}_{{\bar{\psi }}_{\mathrm{in}}}^{\prime }$, respectively. The output, in turn, is represented by the correlation/pseudo-correlation matrix elements of the receive current–current.

Finally, recall that a complex-valued EM RF is termed *proper* if its pseudo-correlation matrix is zero (see Appendix H and Definition A9). The following result is a direct consequence of Theorem 4:

**Corollary** **1** (Preservation of properness of complex-valued RFs)**.**  *Consider the standard stochastic EM information transmission system as treated in Theorem 4. If the input information RF ${\bar{\psi }}_{\mathrm{in}}$ is proper (improper), then the output received current RF ${\bar{J}}_{r}$ is also proper (improper).*

In other words, properness is preserved in both deterministic and stochastic EM information systems. This outcome is expected, as properness is known to be preserved under affine transformations of RVs [85]. It is important to note though that circularity (see Definition A9) is *not* preserved in stochastic EM systems because Gaussianity is not maintained (see Theorem 4). However, circularity is preserved in deterministic EM systems, as established in Theorem 2.

### 3.8. Reduction of Propagator Count Under Physical Symmetries

While the general second-order theory requires 128 correlation and pseudo-correlation propagators (8 types indexed by i,j∈{1,2,3,4}), many of these vanish or become redundant under physically realistic assumptions. Table 8 summarizes common symmetry and modeling conditions that collapse this count. These reductions stem from (i) reciprocity in passive media, (ii) properness of fields (zero pseudo-covariance), (iii) identical or co-located transceiver ports, and (iv) polarization decoupling (e.g., scalar or co-polarized systems). In the most symmetric case—reciprocal, proper, co-polarized, and identical Tx/Rx ports—only the 16 LoS/LoS and NLoS/NLoS correlation propagators survive, and all pseudo-propagators vanish identically. This dramatic simplification underscores that the full 128-propagator structure is a worst-case upper bound; practical systems often require far fewer. In what follows we discuss some of these cases with more details.

#### 3.8.1. Reciprocity Reduction Considerations

We first discuss the most complex and significant reduction, that due to the invocation of the Lorentz reciprocity theorem. We will apply the two versions mentioned in the appendix—one for the FSDGF, and one for the ACGF. To the best of our knowledge, the ACGF reciprocity form of the superdyad is presented here for the first time; accordingly, the derivation is provided in some detail.

**Theorem** **6** (Reciprocity-Induced Symmetry of Correlation Propagators)**.**  *Consider a standard stochastic electromagnetic transmission system (Definition 4) in which the medium Green’s function satisfies Lorentz reciprocity $\bar{\bar{G}}\left(x,x^{\prime },\omega \right)={\bar{\bar{G}}}^{⊤}\left(x^{\prime },x,\omega \right),$ and all antenna current Green’s functions ${\bar{\bar{F}}}_{b}$, b∈{t,s,r} satisfy the corresponding ACGF reciprocity relations (Theorem A8). Then the correlation propagators defined in *(A121)* and *(A131)* obey the following symmetry:*

(69) $$P_{ij}^{\mathrm{los}/\mathrm{nlos}}\left(x,x_{1},x_{4},x_{5};x^{\prime },x_{1}^{\prime },x_{2}^{\prime },x_{3}^{\prime },x_{4}^{\prime },x_{5}^{\prime },\omega \right)={\left[P_{ji}^{\mathrm{nlos}/\mathrm{los}}\left(x^{\prime },x_{1}^{\prime },x_{4}^{\prime },x_{5}^{\prime };x,x_{1},x_{2},x_{3},x_{4},x_{5},\omega \right)\right]}^{\;*},$$

for all i,j∈{1,2,3,4}, where the superscript * denotes complex conjugation.

**Proof.**  We begin from the definitions of the cross-propagators given in (A121) and (A131):

(70) $$\begin{array}{cc} P_{ij}^{\mathrm{los}/\mathrm{nlos}} & :=E\left\{{\bar{\bar{S}}}_{\mathrm{nlos}}^{†}\left(x^{\prime },x_{1}^{\prime },x_{2}^{\prime },x_{3}^{\prime },x_{4}^{\prime },x_{5}^{\prime },\omega ,\varpi \right)\cdot {\bar{a}}_{j}\;{\bar{a}}_{i}^{†}\cdot {\bar{\bar{S}}}_{\mathrm{los}}\left(x,x_{1},x_{4},x_{5},\omega ,\varpi \right)\right\}, \end{array}$$

(71) $$\begin{array}{cc} P_{ji}^{\mathrm{nlos}/\mathrm{los}} & :=E\left\{{\bar{\bar{S}}}_{\mathrm{los}}^{†}\left(x,x_{1},x_{4},x_{5},\omega ,\varpi \right)\cdot {\bar{a}}_{i}\;{\bar{a}}_{j}^{†}\cdot {\bar{\bar{S}}}_{\mathrm{nlos}}\left(x^{\prime },x_{1}^{\prime },x_{2}^{\prime },x_{3}^{\prime },x_{4}^{\prime },x_{5}^{\prime },\omega ,\varpi \right)\right\}. \end{array}$$

Taking the Hermitian transpose of the integrand in (71) yields

(72) $${\left({\bar{\bar{S}}}_{\mathrm{los}}^{†}\cdot {\bar{a}}_{i}\;{\bar{a}}_{j}^{†}\cdot {\bar{\bar{S}}}_{\mathrm{nlos}}\right)}^{†}={\bar{\bar{S}}}_{\mathrm{nlos}}^{†}\cdot {\bar{a}}_{j}\;{\bar{a}}_{i}^{†}\cdot {\bar{\bar{S}}}_{\mathrm{los}},$$

which is precisely the integrand in (70). Therefore,

(73) $$P_{ij}^{\mathrm{los}/\mathrm{nlos}}=E\left\{{\left(X_{ji}\right)}^{†}\right\}={\left(E\{X_{ji}\}\right)}^{\;*}={\left(P_{ji}^{\mathrm{nlos}/\mathrm{los}}\right)}^{\;*},$$

where we used the linearity of expectation and the identity $E\left\{Z^{†}\right\}={\left(E\left\{Z\right\}\right)}^{\;*}$ for any complex random matrix *Z*. However, this algebraic relation alone does not yet incorporate reciprocity. To establish that the propagators are well-defined under the physical assumption of a reciprocal medium, we must verify that the stochastic system superdyadics ${\bar{\bar{S}}}_{\mathrm{los}}$ and ${\bar{\bar{S}}}_{\mathrm{nlos}}$ inherit the appropriate symmetry from the underlying Green’s functions. This is guaranteed by Proposition A3 in Appendix G, which shows that under reciprocity and mutual independence of the Green’s functions,

(74) $$\begin{array}{cc} {\bar{\bar{S}}}_{\mathrm{los}}^{⊤}\left(x,x_{1},x_{4},x_{5}\right) & \overset{d}{=}\bar{\bar{\Omega }}\cdot {\bar{\bar{S}}}_{\mathrm{los}}\left(x_{5},x_{4},x_{1},x\right)\cdot \bar{\bar{\Omega }}, \end{array}$$

(75) $$\begin{array}{cc} {\bar{\bar{S}}}_{\mathrm{nlos}}^{⊤}\left(x,x_{1},\ldots ,x_{5}\right) & \overset{d}{=}\bar{\bar{\Omega }}\cdot {\bar{\bar{S}}}_{\mathrm{nlos}}\left(x_{5},x_{4},\ldots ,x\right)\cdot \bar{\bar{\Omega }}. \end{array}$$

Although these distributional symmetries do not alter the pointwise Hermitian relationship used above, they ensure that the statistical structure of the propagators is consistent with physical reciprocity—i.e., that the joint distributions of the forward and reverse paths are compatible. Consequently, the equality (69) holds.    □

Note that $P_{ij}^{\mathrm{los}/\mathrm{nlos}}={\left(P_{ji}^{\mathrm{nlos}/\mathrm{los}}\right)}^{\;*}$ holds not only as a formal consequence of Hermitian conjugation but also as a physically meaningful statement grounded in the reciprocity of the underlying EM medium and antenna systems.

#### 3.8.2. Interpretation of Identical Tx/Rx Ports in Propagator Reduction

The assumption of *identical transmitter and receiver ports*—realized in full-duplex monostatic configurations where the same physical aperture serves both transmission and reception—introduces a powerful statistical symmetry into the electromagnetic information system. In such scenarios, the distinction between LoS and NLoS propagation paths becomes blurred from the perspective of second-order statistics. Specifically, because the transmit and receive Green’s functions are drawn from the same stochastic ensemble (i.e., ${\bar{\bar{F}}}_{t}\overset{d}{=}{\bar{\bar{F}}}_{r}$) and the spatial support of the excitation and observation manifolds coincides ($S_{t}=S_{r}$), the LoS and NLoS components of the received current become statistically indistinguishable. This leads to the equality of their respective auto-correlation propagators: $P_{ij}^{\mathrm{los}}=P_{ij}^{\mathrm{nlos}}$. Similarly, the cross-propagators between LoS and NLoS paths become symmetric under exchange, yielding $P_{ij}^{\mathrm{los}/\mathrm{nlos}}=P_{ij}^{\mathrm{nlos}/\mathrm{los}}$. These identities follow directly from the invariance of the joint distribution of the system superdyadics ${\bar{\bar{S}}}_{\mathrm{los}}$ and ${\bar{\bar{S}}}_{\mathrm{nlos}}$ under the interchange of transmit and receive roles when the ports are identical.^15^ While this reduction is not universal—it depends on the precise geometric and statistical equivalence of the Tx/Rx apertures—it is frequently applicable in radar, backscatter communication, and monostatic sensing systems. Under this condition, the number of independent propagators is reduced by nearly half, as the LoS and NLoS branches collapse into a single statistical channel. This symmetry, combined with reciprocity and properness, can dramatically simplify the correlation structure, enabling tractable analysis of otherwise intractable stochastic EM systems.

#### 3.8.3. Scalar (Co-Polarized) Systems and the Collapse of Propagator Index Structure

In many practical electromagnetic communication scenarios—such as wirelessly connected antenna arrays operating in a single linear polarization or systems employing orthomode transducers that isolate orthogonal polarizations—the full vectorial nature of Maxwell’s equations can be reduced to an effective scalar model. Specifically, when only one field component (e.g., the *x*-polarized electric field $E_{x}$) is actively excited and observed, all cross-polarization couplings become negligible. This situation is referred to as a *scalar* or *co-polarized* system. Within the framework of EM-RFT, this assumption manifests as a dramatic simplification of the correlation structure of the received current supervector ${\bar{J}}_{r}$. Recall that the index pair (i,j) in the propagator $P_{ij}^{•}$ corresponds to the selection of components from the 4-dimensional complex representation of ${\bar{J}}_{r}$, which encodes both electric/magnetic character and tangential directions on the surface (see Appendix C). In a co-polarized system where only, say, the first tangential electric component is active, the induced current supervector effectively reduces to a scalar random process: ${\bar{J}}_{r}\left(x,\omega ,\varpi \right)\approx {\left[\begin{array}{cccc} J_{e}^{1}\left(x,\omega ,\varpi \right) & 0 & 0 & 0 \end{array}\right]}^{⊤}$. Consequently, any correlation between distinct components vanishes identically: $E\left\{J_{i}\left(x\right)J_{j}^{*}\left(x^{\prime }\right)\right\}=0\;\mathrm{for}\;i\neq j$, which directly implies that all off-diagonal propagators are zero: $P_{ij}^{•}\equiv 0\;\mathrm{for}\;\mathrm{all}\;i\neq j$. Only the four diagonal index pairs (i,i), with i∈{1,2,3,4}, remain potentially nonzero. However, under strict co-polarization (e.g., only $E_{x}$–$E_{x}$ coupling), even among these four, only one component survives—typically corresponding to i=1 if $E_{x}$ aligns with the first local tangent vector ${\hat{\alpha }}_{1}$. Nevertheless, the table entry conservatively retains all four diagonal terms to accommodate systems where multiple but uncoupled polarizations may be present (e.g., dual-polarized MIMO with no cross-polar discrimination loss). Given that the eight propagator types (LoS, NLoS, LoS/NLoS, NLoS/LoS, and their pseudo-counterparts) collapse to only the four proper correlation types under properness, and further that only diagonal indices survive, the total number of independent propagators reduces to: 4 (diag. index pairs) × 4 (types: LoS, NLoS, LoS/NLoS, NLoS/LoS) =16. This reduction reflects a fundamental decoupling of the electromagnetic degrees of freedom, enabling the use of scalar Green’s functions and dramatically lowering computational and analytical complexity. Such models are widely employed in classical MIMO theory, ray-tracing simulations, and channel modeling standards, where polarization is either controlled or assumed statistically independent. The EM-RFT framework rigorously includes this simplification as a special case of the general vectorial theory, valid under the physical assumption of polarization isolation.

### 3.9. An Electromagnetic Derivation of the MIMO Channel Matrix and Shannon Information Capacity

As an application of the deterministic and stochastic theories of information transmission systems presented above, we provide a detailed derivation of the channel matrix $H_{mn}$ for MIMO communication systems. The derivation is intentionally kept at a very general level, consistent with the level described in Definition 3.

Since the MIMO channel matrix $H_{mn}$ (where $m=1,\ldots ,N_{r}$ and $n=1,\ldots ,N_{t}$) is defined as the ratio between the *m*th and *n*th port signals (with all other signals turned off) [95,121], it is necessary to first develop a careful model of the input/output ports to facilitate the application of our response function theory of communication. To achieve this, we will assume the simplest possible model, that of point source ports. However, generalization to more complex models is relatively straightforward, and in fact, one of the main advantages of the response function approach is that it makes such generalizations feasible.

We start with the following model of the input excitation information source field:

(76) $${\bar{\psi }}_{\mathrm{in}}\left(x,\omega ,\varpi \right)=\sum_{n=1}^{N_{t}}{\bar{b}}_{\mathrm{in},n}b_{\mathrm{in},n}\left(\omega ,\varpi \right)\delta \left(x-x_{n}\right),$$

where

(77) $${\bar{b}}_{\mathrm{in},n}={\left[\begin{array}{cc} {\hat{b}}_{1}\left(x_{n}\right)\; & {\hat{b}}_{2}\left(x_{n}\right) \end{array}\right]}^{⊤}.$$

Here, the positions $x_{n}\in S_{t}$ are the locations of the point-like excitation fields applied at the transmit system $S_{t}$. ${\hat{b}}_{i}\left(x_{n}\right),i=1,2,$ are two unit vectors in $R^{3}$. The complex random variable $b_{\mathrm{in},n}\left(\omega ,\varpi \right)\in C$ represents the excitation or information to be transmitted through the system.^16^

For the receiving system, information is usually extracted through current measurement, which can be modeled as the integration of the induced current against a measurement kernel. The point source model of observation is easily realized when we assume that the observation kernel takes the simple form of a Dirac delta function, leading to the following form for the received signal:

(78) $$I\left(x,\omega ,\varpi \right)=\sum_{m=1}^{N_{r}}I_{m}\left(\omega ,\varpi \right)\delta \left(x-x_{m}\right)=\sum_{m=1}^{N_{r}}\left[{\bar{b}}_{\mathrm{out},n}^{†}\left(\omega \right)\cdot \bar{J}\left(x_{m},\omega ,\varpi \right)\right]\delta \left(x-x_{m}\right),$$

where

(79) $$I_{m}\left(\omega ,\varpi \right):={\bar{b}}_{\mathrm{out},n}^{†}\cdot \bar{J}\left(x_{m},\omega ,\varpi \right)$$

is a complex random variable. Here, the positions $x_{m}\in S_{r}$ are the locations of the point-like observation signals at the output (receive) system $S_{r}$. The supervector

(80) $${\bar{b}}_{\mathrm{out},m}\left(\omega \right)={\left[\begin{array}{cc} {\hat{b}}_{1}\left(x_{m}\right)\;b_{\mathrm{out},m}^{1}\left(\omega \right) & {\hat{b}}_{2}\left(x_{m}\right)b_{\mathrm{out},m}^{2}\left(\omega \right) \end{array}\right]}^{⊤}$$

provides information about the direction and gain of the measurements of both the electric and magnetic currents in the receiving system $S_{r}$. Specifically, ${\hat{b}}_{i}\left(x_{m}\right),i=1,2,$ are two unit vectors in $R^{3}$ providing information on the directions of the components extracted from the receiver’s system induce current, while $b_{\mathrm{out},m}^{i}\left(\omega \right),i=1,2,$ are two complex numbers capturing the measurement device gain, conversion ratios, calibration, and other factors.

Based on the discrete input and output models (76) and (78), we may now define the following arrays:

(81) $$\begin{array}{c} \mathrm{Input}\;\mathrm{Vector}:\;\;\;{\bar{x}}_{\mathrm{in}}\left(\omega ,\varpi \right)={\left[\begin{array}{ccc} b_{\mathrm{in},1} & \ldots & b_{\mathrm{in},N_{t}} \end{array}\right]}^{⊤},\\ \mathrm{Output}\;\mathrm{Vector}:\;\;\;{\bar{x}}_{\mathrm{out}}\left(\omega ,\varpi \right)={\left[\begin{array}{ccc} I_{1} & \ldots & I_{N_{r}} \end{array}\right]}^{⊤}. \end{array}$$

The MIMO channel matrix $\bar{\bar{H}}$ is then defined by the relation

(82) $${\bar{x}}_{\mathrm{out}}\left(\omega ,\varpi \right)=\bar{\bar{H}}\left(\omega ,\varpi \right)\;{\bar{x}}_{\mathrm{in}}\left(\omega ,\varpi \right).$$

Consequently, the general channel matrix element in our point-port input/output model is given by the following expression:

(83) $$H_{mn}\left(\omega ,\varpi \right)={\left.\frac{I_{m}\left(\omega ,\varpi \right)}{b_{\mathrm{in},n}\left(\omega ,\varpi \right)}\right|}_{b_{\mathrm{in},l}=0,\forall l\neq n}.$$

The following theorem provides our main result on the EM origin of the MIMO channel matrix.

**Theorem** **7** (MIMO channel matrix derivation)**.**  *Consider the stochastic EM information transmission system specified by Definition 3. We construct a MIMO communication system using the input/output point-port model described by *(76)* and *(78)*, with the decomposition *(6)*. Then the MIMO channel matrix $H_{mn}$ can be expressed in terms of the EM GFs of the system $\left(S_{t},S_{s},S_{r}\right)$ and the port model information, where*

(84) $$H_{mn}\left(\omega ,\varpi \right)=H_{mn}^{\mathrm{los}}\left(\omega ,\varpi \right)+H_{mn}^{\mathrm{nlos}}\left(\omega ,\varpi \right),$$

*with the LoS and NLoS channel matrices general elements $H_{mn}^{\mathrm{los}}\left(\omega ,\varpi \right)$ and $H_{mn}^{\mathrm{nlos}}\left(\omega ,\varpi \right)$ given by Formulas *(A136)* and *(A139)*.*

**Proof.**  See Appendix P.    □

Note that it was assumed in Theorem 7 that $x_{n}\in S_{n}^{t}$ for $n=1,\ldots ,N_{t}$, and $x_{m}\in S_{m}^{r}$ for $m=1,\ldots ,N_{r}$. This implies that each individual antenna in the transmitter and receiver arrays contains exactly one transmitting or receiving port. While this assumption aligns with the conventional operation of MIMO systems and offers intuitive clarity, it is not a necessary condition for the proof of the theorem as detailed in Appendix P.

Because we assumed the EM information transmission system $\left(S_{t},S_{s},S_{r}\right)$ forming the backbone of the MIMO communication link is stochastic, the channel matrix $H_{mn}$ is a random variable. This randomness is not due to noise (as we have not introduced noise yet), but rather due to inherent intrinsic fluctuations in the system, which cause one or more of the various EM GFs to become RFs. Consequently, the channel matrix itself becomes a random matrix (a matrix of random variables). One possible way to characterize this matrix is to compute its expected value $E\{H_{mn}\}$.

We offer the following general remarks on the expressions (A136) and (A139) to clarify their importance:

1. Formulas (A136) and (A139) for the MIMO channel matrix are general, rigorous, and exact. Notably, no approximations, such as neglecting mutual coupling between objects $S_{n}^{b},n=1,\ldots ,N_{b},b\in \left\{t,s,r\right\}$ of the same type, have been made. The use of the general and complete EM GFs in expressing the channel matrix element $H_{mn}$ inherently accounts for all self- and cross-interactions. This is demonstrated by the three expansions of the transmitter, scattering, and receiver current GFs provided in (A38), which apply to all types of GFs (electric/electric, electric/magnetic, etc.).
2. By substituting (A38) into (A136) and (A139), we obtain a highly detailed expression for the MIMO channel matrix that reveals how each pair of possible electromagnetic mutual couplings contributes to the channel matrix, whether originating from the transmitter arrays, receiver arrays, or scattering clusters. However, due to the extreme length and complexity of these expressions, this paper refrains from providing the complete and fully detailed mutual-coupling expressions that are implicit in our original derivation in Theorem 7.
3. The same analysis can also be applied to the production and propagation of stochastic correlation by combining Theorems 4 and 7, and subsequently using (A38) in the general expressions for the stochastic propagators of MIMO links, as given in (A117), (A119), (A121), and (A131).

With the channel matrix derived from EM-RFT, evaluating the Shannon capacity in standard communication system examples becomes straightforward. In Remark (6), we present an example illustrating the application of Theorem 7 to the significant special case of the Gaussian channel.

**Remark** **6** (Deterministic vs. Ergodic Fading Capacity in Stochastic EM Channels)**.**  *Consider the additive white Gaussian noise (AWGN) flat-frequency narrow-band MIMO model*

(85) $${\bar{x}}_{\mathrm{out}}\left(\omega ,\varpi \right)=\bar{\bar{H}}\left(\omega ,\varpi \right)\;{\bar{x}}_{\mathrm{in}}\left(\omega ,\varpi \right)+\bar{w}\left(\omega ,\varpi \right),$$

*where ${\bar{x}}_{\mathrm{in}}\in C^{N_{t}}$ and ${\bar{x}}_{\mathrm{out}}\in C^{N_{r}}$ are the input and output signal vectors, and $\bar{\bar{H}}\left(\omega ,\varpi \right)\in C^{N_{r}\times N_{t}}$ is the channel matrix as defined in *(82)*. The noise vector $\bar{w}\in C^{N_{r}}$ is modeled as circularly symmetric complex Gaussian:*

$$\bar{w}={\left[\begin{array}{ccc} w_{1} & \ldots & w_{N_{r}} \end{array}\right]}^{⊤},\;w_{j}∼\mathrm{CN}\left(0,\sigma_{n_{j}}\right),\;j=1,\ldots ,N_{r},$$

*with mutually independent components, so that $\bar{w}∼\mathrm{CN}\left(0,\mathrm{diag}[\sigma_{w_{1}},\ldots ,\sigma_{w_{N_{r}}}]\right)$. In the AWGN flat-frequency narrow-band MIMO model *(85)*, the noise vector $\bar{w}$ has the same dimension as ${\bar{x}}_{\mathrm{out}}$, implying $N=N_{r}$, while the length of the input vector ${\bar{x}}_{\mathrm{in}}$ is $N_{t}$. For this model, the Shannon capacity per sample depends critically on whether the channel matrix $\bar{\bar{H}}$ is deterministic or stochastic, leading to two distinct notions:*

*1.*  *Deterministic (non-fading) capacity: When all electromagnetic Green’s functions are deterministic, the channel matrix is non-random, i.e., $\bar{\bar{H}}\left(\omega ,\varpi \right)=\bar{\bar{H}}\left(\omega \right)$. The channel is time-invariant and perfectly known, and the capacity is given by [122]*
(86) $$C_{\det}\left(\omega \right)=\log\det\left[1_{N_{r}}+\bar{\bar{H}}\left(\omega \right)\;{\bar{\bar{C}}}_{{\bar{x}}_{\mathrm{in}}}\left(\omega \right)\;{\bar{\bar{H}}}^{†}\left(\omega \right)\;{\bar{\bar{C}}}_{\bar{w}}^{-1}\left(\omega \right)\right],$$
  *where $1_{N_{r}}$ is the $N_{r}\times N_{r}$ identity matrix, ${\bar{\bar{C}}}_{{\bar{x}}_{\mathrm{in}}}$ is the input covariance, and ${\bar{\bar{C}}}_{\bar{w}}=\mathrm{diag}\left[\sigma_{w_{1}},\ldots ,\sigma_{w_{N_{r}}}\right]$.*
*2.*  *Ergodic fading capacity: When one or more Green’s functions are stochastic—e.g., due to random scatterer positions or uncertain surface impedances—the channel matrix becomes a random operator $\bar{\bar{H}}\left(\omega ,\varpi \right)$. In this case, the relevant performance metric is the ergodic capacity, obtained by averaging over the channel ensemble:*
(87) $$C_{\mathrm{erg}}\left(\omega \right)=E_{\varpi }\left\{\log\det\left[1_{N_{r}}+\bar{\bar{H}}\left(\omega ,\varpi \right)\;{\bar{\bar{C}}}_{{\bar{x}}_{\mathrm{in}}}\left(\omega \right)\;{\bar{\bar{H}}}^{†}\left(\omega ,\varpi \right)\;{\bar{\bar{C}}}_{\bar{w}}^{-1}\left(\omega \right)\right]\right\}.$$

*By Theorem 7, both $\bar{\bar{H}}\left(\omega \right)$ and $\bar{\bar{H}}\left(\omega ,\varpi \right)$ are fully determined by the underlying electromagnetic physics. Moreover, the propagator framework developed in Section 4 provides the second-order statistics of $\bar{\bar{H}}\left(\omega ,\varpi \right)$—including its correlation and pseudo-correlation structure—enabling analytical or semi-analytical evaluation of $C_{\mathrm{erg}}$ without resorting to Monte Carlo simulation.*

## 4. The Karhunen–Loève Expansion of Electromagnetic Random Fields

Without loss of generality, unless stated otherwise all EM RFs in this section are assumed to have zero mean. This assumption simplifies the mathematical expressions without sacrificing generality. According to the original vector RF KLE Theorem A3 and expression (A16), a constant (the mean value) can simply be added to the zero-mean KLE to account for nonzero-mean cases.

### 4.1. The Concept of the Electromagnetic Karhunen–Loève Mode: Basic Formulation

Consider a generic EM RF *g* defined on $x\in M\subset R^{d}$. We can apply Algorithm A1 to construct the real vector representation RF $g^{\left(r\right)}\left(x,\omega ,\varpi \right)$. Let us further suppose that we are operating within the standard EM communication system framework described by Definition 4. Applying Theorem A3 to the RF $g^{\left(r\right)}$, we may obtain the following generic Karhunen–Loève expansion (KLE):

(88) $$g^{\left(r\right)}\left(x,\omega ,\varpi \right)=\sum_{n\in N}X_{n}\left(g,\varpi \right)\sqrt{\lambda_{n}\left(g\right)}\;g^{\mathrm{KL},r}\left(x,\omega ,n\right),$$

where the random variables $X_{n}\left(g,\varpi \right)$ are uncorrelated and have unit variance, i.e., they satisfy (A18), while $\lambda_{n}\left(g\right)$ represents the monotonically decreasing sequence of real and non-negative eigenvalues of the covariance operator associated with *g* (see Definition A4). The deterministic field $g^{\mathrm{KL},r}\left(x,\omega ,n\right)$ is the *n*th real KL mode.

Physically, the KL mode represents the most fundamental EM waveform—essentially a solution of the governing EM wave equation—that can independently convey information about the original RF. This mode is closely linked to the concept of degrees of freedom; for a detailed discussion, see [8]. Indeed, note that the original orthonormality relation satisfied by the KL modes is as follows:

(89) $$\begin{array}{c} \int_{x\in M}d^{d}x{\left[g^{\mathrm{KL},r}\left(x,\omega ,n\right)\right]}^{⊤}g^{\mathrm{KL},r}\left(x,\omega ,m\right)=\delta_{nm}, \end{array}$$

for all n,m∈N and $\omega \in R^{+}$. Here, we used the inner product ${\langle \bar{u}\left(x\right),\bar{v}\left(x\right)\rangle }_{L^{2}\left(M,R^{q}\right)}$ defined by (A3). Furthermore, Theorem A4 on the optimality of the KLE provides strong theoretical justification for treating the KL modes as fundamental degrees of freedom in EM wave propagation problems. This holds across all types of EM RFs (vectors, supervectors, dyads, superdyads, etc.).

This latter generalization can be readily achieved as follows. In Algorithm 1, we provide an explanation of how to obtain the following KLE in terms of the original EM RF g(x,ω,ϖ):

(90) $$g\left(x,\omega ,\varpi \right)=\sum_{n\in N}X_{n}\left(g,\varpi \right)\sqrt{\lambda_{n}\left(g\right)}\;g^{\mathrm{KL}}\left(x,\omega ,n\right).$$

The transformation (92) is based on transforming $g^{\left(r\right)}\left(x,\omega ,\varpi \right)$ first to $g^{\left(c\right)}\left(x,\omega ,\varpi \right)$ then the latter to the original form of the RF, i.e., we have

(91) $$g^{\mathrm{KL},r}\left(x,\omega ,\varpi \right)⟶g^{\mathrm{KL},c}\left(x,\omega ,\varpi \right)⟶g^{\mathrm{KL}}\left(x,\omega ,\varpi \right).$$

The reverse transformation can also be constructed, resulting in an effective injective (bidirectional) mapping between the generic KL modes $g^{\mathrm{KL}}\left(x,\omega ,\varpi \right)$ and the equivalent real vector KL modes $g^{\mathrm{KL},r}\left(x,\omega ,\varpi \right)$ by way of the complexified vector KL modes $g^{\mathrm{KL},c}\left(x,\omega ,\varpi \right)$.

**Remark** **7.** *The obtained mode $g^{\mathrm{KL}}$ has the same data type as g. Recall that the original KLE in *(88)* converges in the norm of the function space $L^{2}\left(M,R^{q}\right)$. While it is possible to define specialized norms for the complex vector spaces naturally associated with $g^{\left(c\right)}$ and g, such as $L^{2}\left(M,C^{q}\right)$ or $L^{2}\left(M,C^{q\times p}\right)$, the convergence of the expansion *(90)* should be understood as a shorthand for the convergence of the expansion *(88)*, whose original mode is that of $L^{2}\left(M,R^{q}\right)$. While one could indeed use this real-valued convergence to establish corresponding convergence results in complexified spaces such as $L^{2}\left(M,C^{q}\right)$ or $L^{2}\left(M,C^{q\times p}\right)$, such an approach will not be pursued here.*

**Remark** **8.** *Unfortunately, the KL modes $g^{\mathrm{KL}}\left(x,\omega ,n\right),n\in N$, do not generally satisfy orthonormality relations similar to *(89)*. This arises from the fact that the inner product spaces for complex vectors, such as $L^{2}\left(M,C^{q}\right)$, differ from their real vector space counterparts. Nevertheless, this does not present a significant issue, as one can revert to the original expansion *(88)* for orthonormality relations if needed and then transition back to *(90)*.*

**Algorithm 1** Computation of of the KL Modes of a Generic EM-RF *g*
1.Starting with a generic EM RF g(x,ω,ϖ), obtain its real vector representation $g^{\left(r\right)}\left(x,\omega ,\varpi \right)$ using the maps (A66) (Algorithm A1).2.Obtain the KLE of $g^{\left(r\right)}\left(x,\omega ,\varpi \right)$ given by Equation (88) by applying the procedure of Theorem A3.3.Construct the injective map(92)$$g^{\mathrm{KL},r}\left(x,\omega ,n\right)⟷g^{\mathrm{KL}}\left(x,\omega ,n\right)$$
to transform from (and to) the real representation $g^{\mathrm{KL},r}$ to (and from) the EM form (vector, dyadic, supervector, superdyadic), i.e., the generic form $g^{\mathrm{KL}}$.4.Form the new KLE given in (90) in terms of the KL modes $g^{\mathrm{KL}}\left(x,\omega ,n\right)$.


To summarize, the primary advantage of working with the KLE in the form (90) rather than the original real vector form (88) lies in the ease of calculation within the framework of general EM response theory developed in this paper (particularly the EM GFs discussed in Section 2.2). While the KL modes are rigorously orthogonal only in the real vector form, the expansion (88) is difficult to work with in practice—especially when proving theorems or deriving new results—because EM theorems and equations are typically expressed in a form where the generic EM RF belongs to the set V [see Table 2], a form closely tied to the KLE (90).

Next, we observe that for the most general stochastic EM information transmission system, as described by Definition 4, there exists a maximum of *six* independent KLEs in total. These expansions correspond to the input information field ${\bar{\psi }}_{\mathrm{in}}$ and the five EM Green’s functions. Specifically, we present the following six instantiations of (90), listed in detail for convenience and later reference:

(93) $${\bar{\psi }}_{\mathrm{in}}\left(x,\omega ,\varpi \right)=\sum_{n\in N}X_{n}^{\mathrm{in}}\sqrt{\lambda_{n}^{\mathrm{in}}}\;{\bar{\psi }}_{\mathrm{in}}^{\mathrm{KL}}\left(x,\omega ,n\right),$$

for the input information RF. The three current RF GFs can be expanded as

(94) $$\begin{array}{cc} {\bar{\bar{F}}}_{b}\left(x,x^{\prime },\omega ,\varpi \right)= & \sum_{n\in N}X_{n}^{b}\sqrt{\lambda_{n}^{b}}\;\;{\bar{\bar{F}}}_{b}^{\mathrm{KL}}\left(x,x^{\prime },\omega ,n\right), \end{array}$$

where b∈{t,s,r}. For the medium RF GF, we have two distinct functions, denoted previously as ${\bar{\bar{G}}}_{b^{\prime }},$ where $b^{\prime }\in \left\{t,s\right\}$. For these we have

(95) $$\begin{array}{cc} {\bar{\bar{G}}}_{b^{\prime }}\left(x,x^{\prime },\omega ,\varpi \right)= & \sum_{n\in N}X_{n}^{g_{b^{\prime }}}\sqrt{\lambda_{n}^{g_{b^{\prime }}}}\;\;{\bar{\bar{G}}}_{b^{\prime }}^{\mathrm{KL}}\left(x,x^{\prime },\omega ,n\right). \end{array}$$

Note that for deterministic EM information systems, there is no need to introduce two distinct medium GFs ${\bar{\bar{G}}}_{s}$ and ${\bar{\bar{G}}}_{t}$.

In the KLE expansions above, we have utilized the notation introduced in Equation (90). Drawing on Equations (93)–(95), we identify a total of six statistically independent RVs, defined as follows:

(96) $$X_{n}\left({\bar{\psi }}_{\mathrm{in}},\varpi \right):=X_{n}^{\mathrm{in}},\;X_{n}\left({\bar{\bar{F}}}_{b},\varpi \right):=X_{n}^{b},\;X_{n}\left({\bar{\bar{G}}}_{b^{\prime }},\varpi \right):=X_{n}^{g_{b^{\prime }}},$$

where $b\in \left\{t,s,r\right\},b^{\prime }\in \left\{t,s\right\}$, and another six independent KLE-related non-negative eigenvalues:

(97) $$\lambda_{n}\left({\bar{\psi }}_{\mathrm{in}}\right):=\lambda_{n}^{\mathrm{in}},\;\lambda_{n}\left({\bar{\bar{F}}}_{b}\right):=\lambda_{n}^{b},\;\lambda_{n}\left({\bar{\bar{G}}}_{b^{\prime }}\right):=\lambda_{n}^{g_{b^{\prime }}},$$

all arranged relative to n∈N in descending order of magnitude.

### 4.2. KLE for Deterministic EM Information Transmission Systems

We begin with the KLE of the input information field ${\bar{\psi }}_{\mathrm{in}}$ and proceed to derive the KLEs for all resulting RFs. Note that the EM GFs remain deterministic functions throughout this analysis, meaning we operate within the EM communication system outlined by Definition 1. The following theorem provides a complete characterization of the KL modes for all EM RFs involved.

**Theorem** **8** (KLEs for deterministic EM information systems)**.**  *Consider the detemrinstic information transmission system described in Definition 1. Then the input information field has the KLE given in *(93)*. Moreover, the induced currents, the radiation and scattering fields have KLEs given by*

(98) $${\bar{J}}_{b}\left(x,\omega ,\varpi \right)=\sum_{n\in N}X_{n}^{\mathrm{in}}\sqrt{\lambda_{n}^{\mathrm{in}}}\;\;{\bar{J}}_{b}^{\mathrm{KL}}\left(x,\omega ,n\right),$$

*for b∈{t,s,r} and*

(99) $${\bar{\psi }}_{c}\left(x,\omega ,\varpi \right)=\sum_{n\in N}X_{n}^{\mathrm{in}}\sqrt{\lambda_{n}^{\mathrm{in}}}\;\;{\bar{\psi }}_{c}^{\mathrm{KL}}\left(x,\omega ,n\right),$$

*for c∈{rad,s}. The corresponding KL modes can be computed using the following formulas:*

(100) $$\begin{array}{c} {\bar{J}}_{t}^{\mathrm{KL}}\left(x,\omega ,n\right)=\int_{S_{t}}d^{2}x_{5}\;{\bar{\bar{F}}}_{t}\left(x,x_{5},\omega \right)\cdot {\bar{\psi }}_{\mathrm{in}}^{\mathrm{KL}}\left(x_{5},\omega ,n\right), \end{array}$$

(101) $${\bar{\psi }}_{\mathrm{rad}}^{\mathrm{KL}}\left(x,\omega ,n\right)=\int_{S_{t}}d^{2}x_{4}\int_{S_{t}}d^{2}x_{5}\;\bar{\bar{G}}\left(x,x_{4},\omega \right)\cdot {\bar{\bar{F}}}_{t}\left(x_{4},x_{5},\omega \right)\cdot {\bar{\psi }}_{\mathrm{in}}^{\mathrm{KL}}\left(x_{5},\omega ,n\right),$$

(102) $${\bar{J}}_{s}^{\mathrm{KL}}\left(x,\omega ,n\right)=\int_{S_{s}}d^{2}x_{3}\int_{S_{t}}d^{2}x_{4}\int_{S_{t}}d^{2}x_{5}\;{\bar{\bar{F}}}_{s}\left(x,x_{3},\omega \right)\cdot \bar{\bar{G}}\left(x_{3},x_{4},\omega \right)\cdot {\bar{\bar{F}}}_{t}\left(x_{4},x_{5},\omega \right)\cdot {\bar{\psi }}_{\mathrm{in}}^{\mathrm{KL}}\left(x_{5},\omega ,n\right),$$

(103) $$\begin{array}{c} {\bar{\psi }}_{s}^{\mathrm{KL}}\left(x,\omega ,n\right)=\int_{S_{s}}d^{2}x_{2}\int_{S_{s}}d^{2}x_{3}\int_{S_{t}}d^{2}x_{4}\int_{S_{t}}d^{2}x_{5}\;\bar{\bar{G}}\left(x,x_{2},\omega \right)\cdot {\bar{\bar{F}}}_{s}\left(x_{2},x_{3},\omega \right)\cdot \bar{\bar{G}}\left(x_{3},x_{4},\omega \right)\cdot {\bar{\bar{F}}}_{t}\left(x_{4},x_{5},\omega \right)\cdot {\bar{\psi }}_{\mathrm{in}}^{\mathrm{KL}}\left(x_{5},\omega ,n\right), \end{array}$$

(104) $$\begin{array}{c} {\bar{J}}_{r}^{\mathrm{KL}}\left(x,\omega ,n\right)=\int_{S_{t}}d^{2}x^{\prime }\;\bar{\bar{B}}\left(x,x^{\prime },\omega \right)\cdot {\bar{\psi }}_{\mathrm{in}}^{\mathrm{KL}}\left(x^{\prime },\omega ,n\right), \end{array}$$

*where $\bar{\bar{B}}$ is given by *(32)*, *(33)*, and *(35)* but we only use the deterministic version of these latter formulas.*

**Proof.**  The original KLE (90) can be directly established using Theorem A3, as all conditions of the theorem are satisfied within a standard EM system. The remainder of the proof follows directly from the Lebesgue dominated convergence theorem and Fubini’s theorem. Fubini’s theorem allows for the interchange of the order of integrals, while the Lebesgue dominated convergence theorem permits the interchange of integration and summation. The applicability of the dominated convergence theorem arises because all integrals are performed over compact regions, and the integrands are continuous (for further details, see the proof of Theorem 2). As a result, functions dependent on parameters within these integrals can be replaced by constants, specifically their upper bounds over the integration regions, leading to integrable bound functions and thereby ensuring the applicability of the dominated convergence condition.    □

Theorem 8 provides a powerful and essentially complete characterization of EM RFs within a deterministic information transmission system. As an application, consider the special but significant case where the input information field ${\bar{\psi }}_{\mathrm{in}}$ is Gaussian. In this scenario, the RVs $X_{l}^{\mathrm{in}}$ in (93) can also be taken as Gaussian RVs. It then follows, through the KLEs of all induced RFs in the system, specifically Equations (98) and (99), that these RFs are also Gaussian. Consequently, we arrive at the following immediate but fundamental corollary to Theorem 8.

**Corollary** **2** (Gaussianity in deterministic EM information transmission systems)**.**  *Consider the general deterministic EM information system as described in Definition 1. If the input information RF ${\bar{\psi }}_{\mathrm{in}}$ is Gaussian, then all response RFs ${\bar{J}}_{b}$ and ${\bar{\psi }}_{c}$, where b∈{t,s,r} and c∈{in,s}, are also Gaussian RFs.*

Corollary 2 is unsurprising at first glance. Intuitively, since the EM information system is linear—as evidenced by the fact that all EM interactions are described via GFs—one might expect the output of any process to be expressible as a linear superposition of samples of the input. Consequently, if the input is Gaussian, the output should also be Gaussian. This reasoning aligns with numerous studies of stochastic information transmission systems, where Gaussianity is often assumed [38,94]. However, this intuitive conclusion is not universally valid. In Section 4.3, we demonstrate that in the most general stochastic EM information transmission systems, response RFs generated in reaction to Gaussian input fields are not necessarily Gaussian. This result holds despite the fact that all processes within the system remain linear.

### 4.3. KLE or Stochastic EM Information Transmission Systems

We now derive the complete set of KLEs for a stochastic system, where the five independent EM GFs are modeled as RFs and are themselves expanded using the KLEs. The KLE for the input information field is given by (93), while the expansions for the EM GFs are provided in (94), (95). We start at the transmitting system $S_{t}$. By substituting (93) into (12) and employing (94) for b=t, and then interchanging the order of summation and integration,^17^ we derive the following KLE for the induced transmitting current:

(105) $${\bar{J}}_{t}\left(x,\omega ,\varpi \right)=\sum_{m\in N}\sum_{n\in N}X_{m}^{t}X_{n}^{\mathrm{in}}\sqrt{\lambda_{m}^{t}}\sqrt{\lambda_{n}^{t}}\int_{S_{t}}d^{2}x_{5}\;{\bar{\bar{F}}}_{t}^{\mathrm{KL}}\left(x,x_{5},\omega ,m\right)\cdot {\bar{\psi }}_{\mathrm{in}}^{\mathrm{KL}}\left(x_{5},\omega ,n\right).$$

Next, we now map the pairs $\left(m,n\right)\in N^{2}$ to a single index l∈N. To achieve that, we utilize the following device:

**Lemma** **1.** *There exists an injective map between $N^{q}$ and N for any q∈N. We write this map as $\theta_{q}:N^{q}\to N$.*

For a proof and explicit constructions of these maps, see [123,124]. With a slight abuse of notation, we sometime write l(n,m,…) instead of $l=\theta_{q}\left(n,m,\ldots \right)$. Also, we express m,n,… in terms of *l* by writing $m_{l},n_{l},\ldots ,$ or n(l),m(l),…, etc. The following proposition will be used in what follows.

**Proposition** **2.** *Let $\left(n_{1},\ldots ,n_{p}\right)\in N^{p}$ for some p∈N. Then the following identity holds:*

(106) $$\prod_{i=1}^{p}\delta_{n_{i}\left(l\right)n_{i}\left(l^{\prime }\right)}=\delta_{ll^{\prime }},$$

*where $\delta_{nm}$ is the Kronecker delta function and $n_{i}\left(l\right)$ is the inverse of the map $\theta_{p}$ in Lemma 1 followed by projection into the ith position, i.e., $n_{i}\left(l\right):=P_{i}\circ \theta_{p}^{-1}\left(l\right)$, where $P_{i}\left[(\ldots ,n_{i},\ldots )\right]=n_{i}$.*

Applying Lemma 1 to (105), we can combine the double index (m,n) into a single index, leading to a single series like (90):

(107) $${\bar{J}}_{t}\left(x,\omega ,\varpi \right)=\sum_{l\in N}X_{l}\left({\bar{J}}_{t},\varpi \right)\sqrt{\lambda_{l}\left({\bar{J}}_{t}\right)}\;{\bar{J}}_{t}^{\mathrm{KL}}\left(x,\omega ,l\right),$$

with

(108) $$l=\theta_{2}\left(m,n\right),\;\lambda_{l}\left({\bar{J}}_{t}\right)=\sqrt{\lambda_{m_{l}}^{t}\lambda_{n_{l}}^{\mathrm{in}}},\;X_{l}\left({\bar{J}}_{t},\varpi \right)=X_{m_{l}}^{t}X_{n_{l}}^{\mathrm{in}}.$$

Now in order to compare (107) with the KLE of Equation (90), the effective random variables $X_{l}$ and eigenvalues $\lambda_{l}$ specified by (108) must be shown to satisfy the same conditions (A18) in the original KLE Theorem A3. This, in fact, follows from the general Proposition 3 below. Therefore, the corresponding *l*th KL mode can be expressed as

(109) $${\bar{J}}_{t}^{\mathrm{KL}}\left(x,\omega ,l\right)=\int_{S_{t}}d^{2}x_{5}\;{\bar{\bar{F}}}_{t}^{\mathrm{KL}}\left(x,x_{5},\omega ,m_{l}\right)\cdot {\bar{\psi }}_{\mathrm{in}}^{\mathrm{KL}}\left(x_{5},\omega ,n_{l}\right).$$

The KLE for the radiation field can be similarly derived by substituting (95) and (105) into (15), resulting in

(110) $$\begin{array}{c} {\bar{\psi }}_{\mathrm{rad}}\left(x,\omega ,\varpi \right)=\sum_{p\in N}\sum_{m\in N}\sum_{n\in N}X_{p}^{g_{t}}X_{m}^{t}X_{n}^{\mathrm{in}}\sqrt{\lambda_{p}^{g_{t}}}\sqrt{\lambda_{m}^{t}}\sqrt{\lambda_{n}^{\mathrm{in}}}\int_{S_{t}}d^{2}x_{4}\int_{S_{t}}d^{2}x_{5}\;{\bar{\bar{G}}}_{t}^{\mathrm{KL}}\left(x,x_{4},\omega ,p\right)\cdot {\bar{\bar{F}}}_{t}^{\mathrm{KL}}\left(x_{4},x_{5},\omega ,m\right)\cdot {\bar{\psi }}_{\mathrm{in}}^{\mathrm{KL}}\left(x_{5},\omega ,n\right). \end{array}$$

Again, applying Lemma 1 to the triplet (p,m,n), we can rewrite (110) as a single series as follows:

(111) $${\bar{\psi }}_{\mathrm{rad}}\left(x,\omega ,\varpi \right)=\sum_{l\in N}X_{l}\left({\bar{\psi }}_{\mathrm{rad}},\varpi \right)\sqrt{\lambda_{l}\left({\bar{\psi }}_{\mathrm{rad}}\right)}\;{\bar{\psi }}_{\mathrm{rad}}^{\mathrm{KL}}\left(x,\omega ,l\right),$$

where

(112) $$l=\theta_{3}\left(p,m,n\right),\;\lambda_{l}\left({\bar{\psi }}_{\mathrm{rad}}\right)=\lambda_{p_{l}}^{g_{t}}\lambda_{m_{l}}^{t}\lambda_{n_{l}}^{\mathrm{in}},\;X_{l}\left({\bar{\psi }}_{\mathrm{rad}},\varpi \right)=X_{p_{l}}^{g_{t}}X_{m_{l}}^{t}X_{n_{l}}^{\mathrm{in}},$$

while after applying Proposition 3 we conclude that the *l*th KL mode is

(113) $${\bar{\psi }}_{\mathrm{rad}}^{\mathrm{KL}}\left(x,\omega ,l\right)=\int_{S_{t}}d^{2}x_{4}\int_{S_{t}}d^{2}x_{5}\;{\bar{\bar{G}}}_{t}^{\mathrm{KL}}\left(x,x_{4},\omega ,p_{l}\right)\cdot {\bar{\bar{F}}}_{t}^{\mathrm{KL}}\left(x_{4},x_{5},\omega ,m_{l}\right)\cdot {\bar{\psi }}_{\mathrm{in}}^{\mathrm{KL}}\left(x_{5},\omega ,n_{l}\right).$$

The radiation field in the previous step will next interact with the scattering system $S_{s}$. Substituting (111) into (18) and applying (94), b=s, yields

(114) $$\begin{array}{cc} {\bar{J}}_{s}\left(x,\omega ,\varpi \right) & =\sum_{q\in N}\sum_{p\in N}\sum_{m\in N}\sum_{n\in N}X_{q}^{s}X_{p}^{g_{t}}X_{m}^{t}X_{n}^{\mathrm{in}}\sqrt{\lambda_{q}^{s}\lambda_{p}^{g_{t}}\lambda_{m}^{t}\lambda_{n}^{\mathrm{in}}}\\ \; & \;\times \int_{S_{s}}d^{2}x_{3}\int_{S_{t}}d^{2}x_{4}\int_{S_{t}}d^{2}x_{5}\;{\bar{\bar{F}}}_{s}^{\mathrm{KL}}\left(x,x_{3},\omega ,q\right)\cdot {\bar{\bar{G}}}_{t}^{\mathrm{KL}}\left(x_{3},x_{4},\omega ,p\right)\cdot {\bar{\bar{F}}}_{t}^{\mathrm{KL}}\left(x_{4},x_{5},\omega ,m\right)\cdot {\bar{\psi }}_{\mathrm{in}}^{\mathrm{KL}}\left(x_{5},\omega ,n\right). \end{array}$$

Again, applying Lemma 1 to the 4-tuple (q,p,m,n), we can rewrite (114) as a single series as follows:

(115) $${\bar{J}}_{s}\left(x,\omega ,\varpi \right)=\sum_{l\in N}X_{l}\left({\bar{J}}_{s},\varpi \right)\sqrt{\lambda_{l}\left({\bar{J}}_{s}\right)}\;{\bar{J}}_{s}^{\mathrm{KL}}\left(x,\omega ,l\right),$$

where

(116) $$l=\theta_{4}\left(q,p,m,n\right),\;\lambda_{l}\left({\bar{J}}_{s}\right)=\lambda_{q_{l}}^{s}\lambda_{p_{l}}^{g_{t}}\lambda_{m_{l}}^{t}\lambda_{n_{l}}^{\mathrm{in}},\;X_{l}\left({\bar{J}}_{s},\varpi \right)=X_{q_{l}}^{s}X_{p_{l}}^{g_{t}}X_{m_{l}}^{t}X_{n_{l}}^{\mathrm{in}},$$

and by Proposition 3 the *l*th KL mode can be expressed as

(117) $${\bar{J}}_{s}^{\mathrm{KL}}\left(x,\omega ,l\right)=\int_{S_{s}}d^{2}x_{3}\int_{S_{t}}d^{2}x_{4}\int_{S_{t}}d^{2}x_{5}\;{\bar{\bar{F}}}_{s}^{\mathrm{KL}}\left(x,x_{3},\omega ,q_{l}\right)\cdot {\bar{\bar{G}}}_{t}^{\mathrm{KL}}\left(x_{3},x_{4},\omega ,p_{l}\right)\cdot {\bar{\bar{F}}}_{t}^{\mathrm{KL}}\left(x_{4},x_{5},\omega ,m_{l}\right)\cdot {\bar{\psi }}_{\mathrm{in}}^{\mathrm{KL}}\left(x_{5},\omega ,n_{l}\right).$$

We now can derive the scattered field by radiating the induced scattering current calculated in the the previous step through the medium GF. Indeed, after substituting (115) into (20) and using (95), we obtain

(118) $$\begin{array}{cc} {\bar{\psi }}_{s}\left(x,\omega ,\varpi \right) & =\sum_{r\in N}\sum_{q\in N}\sum_{p\in N}\sum_{m\in N}\sum_{n\in N}X_{r}^{g_{s}}X_{q}^{s}X_{p}^{g_{t}}X_{m}^{t}X_{n}^{\mathrm{in}}\sqrt{\lambda_{r}^{g_{s}}\lambda_{q}^{s}\lambda_{p}^{g_{t}}\lambda_{m}^{t}\lambda_{n}^{\mathrm{in}}}\\ \; & \;\times \int_{S_{s}}d^{2}x_{2}\int_{S_{s}}d^{2}x_{3}\int_{S_{t}}d^{2}x_{4}\int_{S_{t}}d^{2}x_{5}\\ \; & \;\times \;{\bar{\bar{G}}}_{s}^{\mathrm{KL}}\left(x,x_{2},\omega ,r\right)\cdot {\bar{\bar{F}}}_{s}^{\mathrm{KL}}\left(x_{2},x_{3},\omega ,q\right)\cdot {\bar{\bar{G}}}_{t}^{\mathrm{KL}}\left(x_{3},x_{4},\omega ,p\right)\cdot {\bar{\bar{F}}}_{t}^{\mathrm{KL}}\left(x_{4},x_{5},\omega ,m\right)\cdot {\bar{\psi }}_{\mathrm{in}}^{\mathrm{KL}}\left(x_{5},\omega ,n\right). \end{array}$$

By applying Lemma 1 to the 5-tuple (r,q,p,m,n), we can rewrite (118) as a single series as follows:

(119) $${\bar{\psi }}_{s}\left(x,\omega ,\varpi \right)=\sum_{l\in N}X_{l}\left({\bar{\psi }}_{s},\varpi \right)\sqrt{\lambda_{l}\left({\bar{\psi }}_{s}\right)}\;{\bar{\psi }}_{s}^{\mathrm{KL}}\left(x,\omega ,l\right),$$

where

(120) $$l=\theta_{5}\left(r,q,p,m,n\right),\;\lambda_{l}\left({\bar{\psi }}_{s}\right)=\lambda_{r_{l}}^{g_{s}}\lambda_{q_{l}}^{s}\lambda_{p_{l}}^{g_{t}}\lambda_{m_{l}}^{t}\lambda_{n_{l}}^{\mathrm{in}},\;X_{l}\left({\bar{\psi }}_{s},\varpi \right)=X_{r_{l}}^{g_{s}}X_{q_{l}}^{s}X_{p_{l}}^{g_{t}}X_{m_{l}}^{t}X_{n_{l}}^{\mathrm{in}}.$$

As above, Proposition 3 implies that the *l*th KL mode is given by

(121) $$\begin{array}{cc} {\bar{\psi }}_{s}^{\mathrm{KL}}\left(x,\omega ,l\right) & =\int_{S_{s}}d^{2}x_{2}\int_{S_{s}}d^{2}x_{3}\int_{S_{t}}d^{2}x_{4}\int_{S_{t}}d^{2}x_{5}\\ \; & \;\times \;{\bar{\bar{G}}}_{s}^{\mathrm{KL}}\left(x,x_{2},\omega ,r_{l}\right)\cdot {\bar{\bar{F}}}_{s}^{\mathrm{KL}}\left(x_{2},x_{3},\omega ,q_{l}\right)\cdot {\bar{\bar{G}}}_{t}^{\mathrm{KL}}\left(x_{3},x_{4},\omega ,p_{l}\right)\cdot {\bar{\bar{F}}}_{t}^{\mathrm{KL}}\left(x_{4},x_{5},\omega ,m_{l}\right)\cdot {\bar{\psi }}_{\mathrm{in}}^{\mathrm{KL}}\left(x_{5},\omega ,n_{l}\right). \end{array}$$

Finally, we arrive at the received current, which is composed of two components, the NLoS and the LoS contributions [see Equation (30)]. The NLoS received current is induced by the interaction of the scattered field found in the previous step into with the receiving system $S_{r}$. Plugging (119) into (24) and making use of (94), b=r, we arrive at

(122) $$\begin{array}{cc} {\bar{J}}_{r}^{\mathrm{nlos}}\left(x,\omega ,\varpi \right) & =\sum_{s\in N}\sum_{r\in N}\sum_{q\in N}\sum_{p\in N}\sum_{m\in N}\sum_{n\in N}X_{s}^{r}X_{r}^{g_{s}}X_{q}^{s}X_{p}^{g_{t}}X_{m}^{t}X_{n}^{\mathrm{in}}\sqrt{\lambda_{s}^{r}\lambda_{r}^{g_{s}}\lambda_{q}^{s}\lambda_{p}^{g_{t}}\lambda_{m}^{t}\lambda_{n}^{\mathrm{in}}}\\ \; & \;\times \int_{S_{r}}d^{2}x_{1}\int_{S_{s}}d^{2}x_{2}\int_{S_{s}}d^{2}x_{3}\int_{S_{t}}d^{2}x_{4}\int_{S_{t}}d^{2}x_{5}\\ \; & \;\times \;{\bar{\bar{F}}}_{r}^{\mathrm{KL}}\left(x,x_{1},\omega ,s\right)\cdot {\bar{\bar{G}}}_{s}^{\mathrm{KL}}\left(x_{1},x_{2},\omega ,r\right)\cdot {\bar{\bar{F}}}_{s}^{\mathrm{KL}}\left(x_{2},x_{3},\omega ,q\right)\cdot {\bar{\bar{G}}}_{t}^{\mathrm{KL}}\left(x_{3},x_{4},\omega ,p\right)\cdot {\bar{\bar{F}}}_{t}^{\mathrm{KL}}\left(x_{4},x_{5},\omega ,m\right)\cdot {\bar{\psi }}_{\mathrm{in}}^{\mathrm{KL}}\left(x_{5},\omega ,n\right). \end{array}$$

By applying Lemma 1 to the 6-tuple (s,r,q,p,m,n), we can rewrite (122) as a single series as follows:

(123) $${\bar{J}}_{r}^{\mathrm{nlos}}\left(x,\omega ,\varpi \right)=\sum_{l\in N}X_{l}\left({\bar{J}}_{r}^{\mathrm{nlos}},\varpi \right)\sqrt{\lambda_{l}\left({\bar{J}}_{r}^{\mathrm{nlos}}\right)}\;{\bar{J}}_{r}^{\mathrm{nlos},\mathrm{KL}}\left(x,\omega ,l\right),$$

where

(124) $$l=\theta_{6}\left(s,r,q,p,m,n\right),\;\lambda_{l}\left({\bar{J}}_{r}^{\mathrm{nlos}}\right)=\lambda_{s_{l}}^{r}\lambda_{r_{l}}^{g_{s}}\lambda_{q_{l}}^{s}\lambda_{p_{l}}^{g_{t}}\lambda_{m_{l}}^{t}\lambda_{n_{l}}^{\mathrm{in}},\;X_{l}\left({\bar{J}}_{r}^{\mathrm{nlos}},\varpi \right)=X_{s_{l}}^{r}X_{r_{l}}^{g_{s}}X_{q_{l}}^{s}X_{p_{l}}^{g_{t}}X_{m_{l}}^{t}X_{n_{l}}^{\mathrm{in}},$$

where an application of Proposition 3 leads to the fact that the *l*th KL mode is

(125) $$\begin{array}{c} {\bar{J}}_{r}^{\mathrm{nlos},\mathrm{KL}}\left(x,\omega ,l\right)=\int_{S_{r}}d^{2}x_{1}\int_{S_{s}}d^{2}x_{2}\int_{S_{s}}d^{2}x_{3}\int_{S_{t}}d^{2}x_{4}\int_{S_{t}}d^{2}x_{5}\\ \times \;{\bar{\bar{F}}}_{r}^{\mathrm{KL}}\left(x,x_{1},\omega ,s_{l}\right)\cdot {\bar{\bar{G}}}_{s}^{\mathrm{KL}}\left(x_{1},x_{2},\omega ,r_{l}\right)\cdot {\bar{\bar{F}}}_{s}^{\mathrm{KL}}\left(x_{2},x_{3},\omega ,q_{l}\right)\cdot {\bar{\bar{G}}}_{t}^{\mathrm{KL}}\left(x_{3},x_{4},\omega ,p_{l}\right)\cdot {\bar{\bar{F}}}_{t}^{\mathrm{KL}}\left(x_{4},x_{5},\omega ,m_{l}\right)\cdot {\bar{\psi }}_{\mathrm{in}}^{\mathrm{KL}}\left(x_{5},\omega ,n_{l}\right). \end{array}$$

To complete the derivation of the received current, the LoS received current is calculated as the due process of the interaction of the radiation field ${\bar{\psi }}_{\mathrm{rad}}$, already found in a previous step, with the receiving system $S_{r}$. Plugging (111) into (24) and making use of (94), b=r, we arrive at

(126) $$\begin{array}{cc} {\bar{J}}_{r}^{\mathrm{los}}\left(x,\omega ,\varpi \right) & =\sum_{s\in N}\sum_{p\in N}\sum_{m\in N}\sum_{n\in N}X_{s}^{r}X_{p}^{g_{t}}X_{m}^{t}X_{n}^{\mathrm{in}}\sqrt{\lambda_{s}^{r}\lambda_{p}^{g_{t}}\lambda_{m}^{t}\lambda_{n}^{\mathrm{in}}}\\ \; & \;\times \int_{S_{r}}d^{2}x_{1}\int_{S_{t}}d^{2}x_{4}\int_{S_{t}}d^{2}x_{5}\;{\bar{\bar{F}}}_{r}^{\mathrm{KL}}\left(x,x_{1},\omega ,s\right)\cdot {\bar{\bar{G}}}_{t}^{\mathrm{KL}}\left(x_{1},x_{4},\omega ,p\right)\cdot {\bar{\bar{F}}}_{t}^{\mathrm{KL}}\left(x_{4},x_{5},\omega ,m\right)\cdot {\bar{\psi }}_{\mathrm{in}}^{\mathrm{KL}}\left(x_{5},\omega ,n\right). \end{array}$$

By applying Lemma 1 to the 4-tuple (s,p,m,n), we may rewrite (126) as a single series as follows:

(127) $$\begin{array}{cc} {\bar{J}}_{r}^{\mathrm{los}} & \left(x,\omega ,\varpi \right)=\sum_{l\in N}X_{l}\left({\bar{J}}_{r}^{\mathrm{los}},\varpi \right)\sqrt{\lambda_{l}\left({\bar{J}}_{r}^{\mathrm{los}}\right)}\;{\bar{J}}_{r}^{\mathrm{los},\mathrm{KL}}\left(x,\omega ,l\right), \end{array}$$

where

(128) $$l=\theta_{4}\left(s,p,m,n\right),\;\lambda_{l}\left({\bar{J}}_{r}^{\mathrm{los}}\right)=\lambda_{s_{l}}^{r}\lambda_{p_{l}}^{g_{t}}\lambda_{m_{l}}^{t}\lambda_{n_{l}}^{\mathrm{in}},\;X_{l}\left({\bar{J}}_{r}^{\mathrm{los}},\varpi \right)=X_{s_{l}}^{r}X_{p_{l}}^{g_{t}}X_{m_{l}}^{t}X_{n_{l}}^{\mathrm{in}}.$$

From Proposition 3, the RVs $X_{l}\left({\bar{J}}_{r}^{\mathrm{los}},\varpi \right)$ are orthonormal while the non-negative eigenvalues $\lambda_{l}\left({\bar{J}}_{r}^{\mathrm{los}}\right)$ are decreasing relative to *l*, allowing us to express the corresponding *l*th KL mode as

(129) $${\bar{J}}_{r}^{\mathrm{los},\mathrm{KL}}\left(x,\omega ,l\right)=\int_{S_{r}}d^{2}x_{1}\int_{S_{t}}d^{2}x_{4}\int_{S_{t}}d^{2}x_{5}\;{\bar{\bar{F}}}_{r}^{\mathrm{KL}}\left(x,x_{1},\omega ,s_{l}\right)\cdot {\bar{\bar{G}}}_{t}^{\mathrm{KL}}\left(x_{1},x_{4},\omega ,p_{l}\right)\cdot {\bar{\bar{F}}}_{t}^{\mathrm{KL}}\left(x_{4},x_{5},\omega ,m_{l}\right)\cdot {\bar{\psi }}_{\mathrm{in}}^{\mathrm{KL}}\left(x_{5},\omega ,n_{l}\right).$$

**Lemma** **2.** *The six stochastic EM RVs defined in *(96)*, namely $X_{s}^{r},X_{r}^{g_{s}},X_{q}^{s},X_{p}^{g_{t}},X_{m}^{t},$ and $X_{n}^{\mathrm{in}}$, are all mutually statistically independent.*

**Proof.**  The RVs above correspond to the KLE’s RVs of the respective RFs ${\bar{\bar{F}}}_{r},{\bar{\bar{G}}}_{s},{\bar{\bar{F}}}_{s},{\bar{\bar{G}}}_{t},{\bar{\bar{F}}}_{t},$ and ${\bar{\psi }}_{\mathrm{in}}$. These RFs are stochastically independent in standard stochastic EM information transmission systems (see Definition 4). Consequently, by the definition of the KLE’s RVs, given in Equation (A17), it follows that all these RVs are also stochastically independent.    □

**Proposition** **3.** *Let $X_{l}$ stand for any of the following effective (product) RVs, followed by the corresponding KLE eigenvalues:*

*1.*  *$X_{l}\left({\bar{J}}_{t},\varpi \right)$, $\lambda_{l}\left({\bar{J}}_{t}\right)$ [Equation *(108)*].*
*2.*  *$X_{l}\left({\bar{\psi }}_{\mathrm{rad}},\varpi \right)$, $\lambda_{l}\left({\bar{\psi }}_{\mathrm{rad}}\right)$ [Equation *(112)*].*
*3.*  *$X_{l}\left({\bar{J}}_{s},\varpi \right)$, $\lambda_{l}\left({\bar{J}}_{s}\right)$ [Equation *(116)*].*
*4.*  *$X_{l}\left({\bar{\psi }}_{s},\varpi \right)$, $\lambda_{l}\left({\bar{\psi }}_{s}\right)$ [Equation *(120)*].*
*5.*  *$X_{l}\left({\bar{J}}_{r}^{\mathrm{nlos}},\varpi \right)$, $\lambda_{l}\left({\bar{J}}_{r}^{\mathrm{nlos}}\right)$ [Equation *(124)*].*
*6.*  *$X_{l}\left({\bar{J}}_{r}^{\mathrm{los}},\varpi \right)$, $\lambda_{l}\left({\bar{J}}_{r}^{\mathrm{los}}\right)$ [Equation *(128)*].*

*The index l for each case is as defined in the corresponding equation between brackets. Then the following holds:*

*1.*  *The RVs are zero-mean and mutually uncorrelated:*
(130) $$\begin{array}{ccc} \; & E\left\{X_{l}\right\}=0,\;\;\;\; & E\left\{X_{l}X_{l}^{\prime }\right\}=\delta_{ll^{\prime }}, \end{array}$$
  *for all l∈N.*
*2.*  *All of the six non-negative eigenvalues $\lambda_{l}$ listed above are arranged as decreasing sequence relative to l.*

**Proof.**  For case 1, the relations in (130) follow from applying Lemma 2 and the conditions (A18) and subsequently utilizing the identity (106). Case 2 is an immediate consequence from the fact that the six eigenvalue sequences $\lambda_{s}^{r},\lambda_{r}^{g_{s}},\lambda_{q}^{s},\lambda_{p}^{g_{t}},\lambda_{m}^{t},$ and $\lambda_{n}^{\mathrm{in}}$ are decreasing (Theorem A3), while the product of two non-negative decreasing sequences is decreasing.    □

We now gather all of the previous development into the following key theorem that provide a complete description of the stochastic structure of deterministic EM information processing systems.

**Theorem** **9** (The KLE for standard stochastic EM information transmission system)**.**  *Consider the standard stochastic EM information system described in Definition 4. Using the notation provided in Table 4, we assume that input covariance and pseudo-covariance matrices are available for all five EM GFs in the set W, as well as for the input information field ${\bar{\psi }}_{\mathrm{in}}$. Additionally, assume all means are zero. Then, the following holds:*

*1.*  *The input information RF ${\bar{\psi }}_{\mathrm{in}}$ and the five EM GFs ${\bar{\bar{F}}}_{b},b\in \left\{t,s,r\right\}$ and ${\bar{\bar{G}}}_{b}^{\prime },b^{\prime }\in \left\{t,s\right\}$ can be expressed using the KLE expansions given in *(93)*, *(94)*, and *(95)*, respectively.*
*2.*  *The five response RFs g∈Q (see Table 4) can be represented through the KLE expansions provided in *(107)*, *(115)*, *(111)*, and *(119)*.*
*3.*  *The final received current RF ${\bar{J}}_{r}$ can be expressed as*
(131) $${\bar{J}}_{r}\left(x,\omega ,\varpi \right)={\bar{J}}_{r}^{\mathrm{nlos}}\left(x,\omega ,\varpi \right)+{\bar{J}}_{r}^{\mathrm{los}}\left(x,\omega ,\varpi \right),$$
  *where ${\bar{J}}_{r}^{\mathrm{nlos}}$ and ${\bar{J}}_{r}^{\mathrm{los}}$ are given by *(123)* and *(127)*, respectively.*

**Corollary** **3** (The general stochastic structure of the received RF in standard stochastic EM information systems)**.**  *Consider the scenario described in Theorem 9. The received RF, ${\bar{J}}_{r}\left(x,\omega ,\varpi \right)$, can be expressed in the following general form:*

(132) $$\begin{array}{cc} \; & {\bar{J}}_{r}\left(x,\omega ,\varpi \right)=\sum_{l_{1}\in N}{\bar{\beta }}_{l_{1}}\left(x,\omega \right)V_{l_{1}}^{\mathrm{nlos}}\left(\varpi \right)+\sum_{l_{2}\in N}{\bar{\gamma }}_{l_{2}}\left(x,\omega \right)V_{l_{2}}^{\mathrm{los}}\left(\varpi \right), \end{array}$$

*where $V_{l_{1}}^{\mathrm{nlos}}$ and $V_{l_{2}}^{\mathrm{los}}$ are RVs, the general structural form of which is given by:*

(133) $$V_{l_{1}}^{\mathrm{nlos}}:=X_{s_{l_{1}}}^{r}X_{r_{l_{1}}}^{g_{s}}X_{q_{l_{1}}}^{s}X_{p_{l_{1}}}^{g_{t}}X_{m_{l_{1}}}^{t}X_{n_{l_{1}}}^{\mathrm{in}},\;V_{l_{1}}^{\mathrm{nlos}}:=X_{s_{l_{1}}}^{r}X_{p_{l_{2}}}^{g_{t}}X_{m_{l_{2}}}^{t}X_{n_{l_{2}}}^{\mathrm{in}}.$$

*where $l_{1}=\theta_{6}\left(s,r,q,p,m,n\right)$ and $l_{2}=\theta_{4}\left(s,p,m,n\right)$. The coefficients of the RV polynomial expansion are given by:*

(134) $${\bar{\beta }}_{l_{1}}\left(x,\omega \right):={\bar{J}}_{r}^{\mathrm{nlos},\mathrm{KL}}\left(x,\omega ,\varpi ,l_{1}\right)\sqrt{\lambda_{l_{1}}\left({\bar{J}}_{r}^{\mathrm{nlos}}\right)},\;{\bar{\gamma }}_{l_{2}}\left(x,\omega \right):={\bar{J}}_{r}^{\mathrm{los},\mathrm{KL}}\left(x,\omega ,\varpi ,l_{2}\right)\sqrt{\lambda_{l_{2}}\left({\bar{J}}_{r}^{\mathrm{los}}\right)}.$$

*Regarding the RVs appearing in *(133)*, namely the set $\left\{V_{l_{1}}^{\mathrm{nlos}},V_{l_{2}}^{\mathrm{los}}\right\}$, first consider the collections defined as follows:*

(135) $$R_{a}:={\left\{X_{n}^{a}\right\}}_{n\in N},\;\;\forall a\in \left\{r,g_{s},s,g_{t},t,\mathrm{in}\right\},\;R:={\left\{X_{n}^{r},X_{n}^{g_{s}},X_{n}^{s},X_{n}^{g_{t}},X_{n}^{t},X_{n}^{\mathrm{in}}\right\}}_{n\in N}=\underset{a}{⋃}R_{a}.$$

*Then these sets of RVs satisfy the following conditions:*

*1.*  *The set R consists of zero-mean real RVs.*
*2.*  *Any two RVs chosen from any two of the six subsets $R_{a}$ of RVs above are mutually statistically independent.*
*3.*  *Within each subset of the six subsets $R_{a}$, any two RVs are mutually uncorrelated.*

*Additionally, the two RVs $V_{l_{1}}^{\mathrm{nlos}}$ and $V_{l_{2}}^{\mathrm{los}}$ satisfy the following relation:*

(136) $$E\left\{V_{l}^{\mathrm{nlos}}V_{l^{\prime }}^{\mathrm{nlos}}\right\}=E\left\{V_{l}^{\mathrm{los}}V_{l^{\prime }}^{\mathrm{los}}\right\}=\delta_{ll^{\prime }},\;E\left\{V_{l}^{\mathrm{nlos}}V_{l^{\prime }}^{\mathrm{los}}\right\}=0,$$

*for all $l,l^{\prime }\in N$.*

**Proposition** **4** (Correlation and pseudo-correlation matrices of received current in terms of KL modes)**.**  *Consider a standard stochastic EM information transmission system where Corollary 3 applies. The correlation and pseudo-correlation matrices of the received current in a generic stochastic EM information system can be computed using the following expansion:*

(137) $$\begin{array}{cc} R_{{\bar{J}}_{r}}\left(x,x^{\prime },\omega \right) & =\sum_{l_{1}\in N}{\bar{\beta }}_{l_{1}}\left(x,\omega \right){\bar{\beta }}_{l_{1}}^{†}\left(x^{\prime },\omega \right)+\sum_{l_{2}\in N}{\bar{\gamma }}_{l_{2}}\left(x,\omega \right){\bar{\gamma }}_{l_{2}}^{†}\left(x^{\prime },\omega \right),\\ R_{{\bar{J}}_{r}}^{\prime }\left(x,x^{\prime },\omega \right) & =\sum_{l_{1}\in N}{\bar{\beta }}_{l_{1}}\left(x,\omega \right){\bar{\beta }}_{l_{1}}^{⊤}\left(x^{\prime },\omega \right)+\sum_{l_{2}\in N}{\bar{\gamma }}_{l_{2}}\left(x,\omega \right){\bar{\gamma }}_{l_{2}}^{⊤}\left(x^{\prime },\omega \right), \end{array}$$

*where the notation and definitions in Corollary 3 were utilized.*

Proposition 4 serves as a consistency check on the content of Corollary 3. Specifically, relation (137) expresses the well-known fact that correlation/covariance can be expanded in terms of the eigenfunctions of the correlation/covariance operators (see Appendix B). These relations demonstrate that this fact not only holds for the real vector representation and its KL modes $g^{\mathrm{KL},r}$, but also extends to the complex-valued KL modes $g^{\mathrm{KL}}$. Furthermore, the importance of this proposition is enhanced when we substitute (125) and (129) into (134), and subsequently into (137). This process yields expressions for the correlation/pseudo-correlation of the final received current in terms of “atomic” constituents, namely the statistics of the input information field and the data of the five EM GFs. In this regard, relations (137) provide an alternative method for computing the final signal correlation, distinct from the correlation propagator method outlined in Section 3.7.

**Proposition** **5** (Cross-covariance/pseudo-covariance in stochastic EM information systems)**.**  *Consider a standard stochastic EM information transmission system to which Corollary 3 applies but without assuming zero-mean RFs. Let g∈Q represent a generic response RF (as defined in Table 4). Then the cross-covariance and pseudo-covariance functions are governed by the following relations:*

(138) $$C_{g{\bar{\psi }}_{\mathrm{in}}}=C_{g{\bar{\psi }}_{\mathrm{in}}}^{\prime }=C_{{\bar{\psi }}_{\mathrm{in}}g}=C_{{\bar{\psi }}_{\mathrm{in}}g}^{\prime }=0,$$

*for all g∈Q and across their entire domains.*

Proposition 5 highlights a significant distinction between deterministic and stochastic EM information systems, as no analogous result exists in the deterministic case. This proposition is a direct consequence of Corollary 3 and serves as a defining characteristic of the stochastic (generic) scenario. For zero-mean RFs, the relations in (138) indicate that the input RF and any system response (output RF) are uncorrelated. This seemingly counterintuitive result becomes more transparent when considered in light of Corollary 4, which explicitly prohibits a general stochastic EM system from necessarily producing a Gaussian response RF when the input RF is Gaussian. Importantly, Proposition 5 does not imply that the input and output RFs are stochastically independent, despite their possible lack of correlation (see also Remark 9).

From the exact expression (132), we can deduce that the general structural form of the received current RF in a stochastic EM information system is significantly more complex than the corresponding result for a deterministic system, as worked out in Section 4.2. In the stochastic case, the received current is represented as an infinite series of several RVs, with two types of terms. One term (the NLoS case) consists of a monomial composed of six independent product RVs, while the LoS monomial consists of four independent product RVs. While all of these RVs are zero-mean and real, the coefficients of the monomials are generally complex. When the KLE is truncated to $N_{\mathrm{KL}}$ terms, the final received RF can be expressed (at each position $x\in S_{r}$) as a complex-weighted polynomial with $N_{\mathrm{KL}}$ monomials, each involving independent product RVs—two types, with six and four RVs, respectively.

Let us now observe that due to the nonlinear RV structure described above, an analog of Corollary 2 does not hold for the general case of the stochastic EM information processing system outlined in Theorem 9. In fact, the following corollary can be deduced.

**Corollary** **4** (Gaussianity in stochastic EM information transmission systems)**.**  *Consider the general stochastic EM information system as described in Definition 4. If the input information RF ${\bar{\psi }}_{\mathrm{in}}$ and the five EM GF RFs, the elements of the set W (see Table 4), are all Gaussian RFs, then it is not necessarily the case that all response RFs, the elements of the set Q (see Table 4), are also Gaussian RFs.*

**Proof.**  The proof is established through counterexamples. It is well known that the product of two Gaussian RVs, for instance, is not Gaussian, except in trivial cases (see Appendix Q). This property can be easily generalized to monomials involving any number of RVs. Furthermore, the finite sum of non-Gaussian RVs is, in general, not Gaussian. As a result, it is relatively straightforward to construct numerous examples of EM information systems where all input statistical RFs are Gaussian, but the received current RF is not. In particular, this becomes evident when we approximate the KLE with a finite number of terms, since polynomials of Gaussian RVs are generally not Gaussian.    □

The implications of Corollary 4 are significant for EIT and communication systems, where the assumption of Gaussianity is commonly adopted. Our analysis, however, demonstrates that when one or more of the EM GFs is modeled as an RF, the circulating information RFs generally cannot be modeled as Gaussian RFs. Moreover, our theory provides the exact form of the RV structure at each point and shows that it can be approximated to any desired accuracy as a special polynomial of several RVs, with only two types of monomials (six and four RVs, respectively). These conclusions are derived for the most general EM information system and are not based on specific examples.

**Remark** **9.** *Proposition 5 offers a clear rationale for the validity of Corollary 4. Specifically, in the case of zero-mean RFs, if all response RFs of the system excited by a Gaussian RF were also Gaussian, then, according to Proposition 5, they would necessarily be uncorrelated. This uncorrelated state would imply statistical independence between the input and output RFs, leading to a contradiction, as the output in any system depends on the input. Consequently, Proposition 5 imposes a restriction on the permissible probability density functions of response RFs in general stochastic EM information systems. For the zero-mean RF case, these response RFs must maintain statistical dependence on their input excitation RF even when they are uncorrelated with it. Clearly a Gaussian pdf for a response RF is ruled out by such restriction.*

Another interesting result that can be derived from Corollary 4 is the following.

**Proposition** **6.** *Consider the situation addressed by Corollary 4. Let a stochastic EM system be a MIMO communication system with a point-port model, as described in Section 3.9. If all stochastic EM GFs in the MIMO system are modeled as Gaussian RFs, the elements of the channel matrix $\bar{\bar{H}}$ are not necessarily Gaussian.*

**Proof.**  From Formulas (A135) and (A138) and the KLEs of the stochastic EM GFs (94) and (95), we find that the NLoS and LoS components of the MIMO channel matrix can be written as polynomials of six and four RVs, respectively, i.e., leading to an expression similar to (132). Therefore, following the logic of Corollary 4, we deduce that the MIMO channel matrix cannot be Gaussian because a polynomial of Gaussian RVs is not necessarily Gaussian.    □

Crucially, this non-Gaussianity persists under practical truncation because the number of significant KL modes is physically limited by the EM degrees of freedom (DoF), which scale with electrical size and bandwidth but remain finite. Unlike the classical Central Limit Theorem (CLT), which requires a large number of identically distributed, comparable-magnitude contributions, the KL spectrum in realistic EM systems is rapidly decaying: higher-order modes carry negligible energy and do not contribute meaningfully to the sum. Thus, the CLT does not apply, and the output statistics retain strong non-Gaussian features even when all underlying fields are Gaussian. We conclude from this proposition that the common assumption of modeling channel matrices as Gaussian random matrices imposes severe restrictions on the statistics of the EM GFs underlying the physics of the problem. Therefore, we suggest that a more appropriate approach is to assume the EM GFs are Gaussian RFs and then attempt to estimate the pdf of the channel matrix using the form (132).

To illustrate this result—and to emphasize its practical relevance despite its apparent simplicity—we provide a concrete synthetic example in Appendix S. This example explicitly constructs a point-port MIMO channel using the KL expansions of the underlying stochastic Green’s functions, with two modes for the LoS path (each a product of four independent Gaussian random variables) and three modes for the NLoS path (each a product of six independent Gaussian random variables). Although all constituent random variables are Gaussian and mutually independent across physical sources—as guaranteed by Proposition 3—the resulting channel entries are demonstrably non-Gaussian due to the polynomial structure of the response functions.

### 4.4. Entropy and Mutual Information for Electromagnetic Random Fields in Communication Systems

We here present a concise discussion of entropy and mutual information in EM-RFT. While this topic extends beyond the KL theory of EM-RFs developed earlier, the general form of the received current, expressed as a polynomial of multiple RVs, e.g., Equation (132), is closely tied to the analysis of mutual information in EM communication systems. Accordingly, we briefly address it here. In Appendix R, we introduce a general definition for the approximation hierarchy of entropy in RFT, inspired by the recent framework proposed in [19] for interpreting Shannon capacity in radiating EM systems. The central idea is to define the entropy of a generic EM RF g∈V over a region *D* by evaluating *g* at *n* positions x∈D. This approach yields the quantity $H_{g}^{n}\left(\bar{x},D\right)$, whose construction is outlined in Definition A12. The *n*-level description encapsulates the entropy content of the RF g(x) on x∈D by selecting *n* degrees of freedom from the originally infinite set. The definition clearly depends on the distribution of these *n* positions, encoded in the vector $\bar{x}$. To obtain an intrinsic definition independent of the specific positions in *D*, we average over all possible *n* positions, resulting in the quantity $H_{g}^{n}\left(D\right)$. For every given region *D* and RF *g*, this quantity depends solely on *n* and represents the entropy of *n* degrees of freedom in the RF *g* over *D*. These concepts naturally extend to define an (n,m)-point joint entropy for two EM RFs, as formalized in Definition A13. This joint entropy, denoted as $H_{g_{1}g_{2}}^{n,m}\left(\bar{x},\bar{y};D_{1},D_{2}\right)$, involves the vectors $\bar{x}$ and $\bar{y}$, which specify the locations of *n* and *m* positions in $D_{1}$ and $D_{2}$, where the RFs $g_{1}$ and $g_{2}$ are evaluated, respectively. Furthermore, by averaging this quantity over all *m* and *n* positions in $D_{1}$ and $D_{2}$, we obtain $H_{g_{1}g_{2}}^{n,m}\left(D_{1},D_{2}\right)$, which represents the intrinsic joint entropy content when *n* and *m* degrees of freedom are selected in $D_{1}$ and $D_{2}$, respectively. The generalization of this approach to derive an intrinsic measure of mutual entropy $I_{g_{1}g_{2}}^{n,m}\left(D_{1}:D_{2}\right)$ is provided in Definition A14.

It is natural to expect that this approximation hierarchy will eventually converge, such that the limits $\lim_{n\to \infty }H_{g}^{n}\left(D\right)$, $\lim_{n,m\to \infty }H_{g_{1}g_{2}}^{n,m}\left(D_{1},D_{2}\right)$, $\lim_{n,m\to \infty }I_{g_{1}g_{2}}^{n,m}\left(D_{1}:D_{2}\right)$, and similar expressions exist or that reasonable approximations of them can be defined. An empirical study presented in [19] investigated this question for a specific instance of $I_{g_{1}g_{2}}^{n,m}\left(D_{1}:D_{2}\right)$ and demonstrated that for several basic geometric regions $D_{1}$ and $D_{2}$, a practical limit can be computed when the densities $n/|D_{1}|$ and $m/|D_{2}|$ are sufficiently large.^18^ However, since constructing numerical examples requires extensive preparation and cannot provide a universally valid answer for all cases, we do not pursue such an investigation here. Instead, we offer additional comments below to connect the evaluation of mutual information with the KL theory of EM RFs.

As an illustrative example of these definitions, let us see how the conventional definition of mutual information can be reconstucted from our EM-RFT approach. Let us deploy the point-like port model described by Equation (81) in Section 3.9. In this scenario, $N_{t}$ excitations are applied at locations specified by the $3N_{t}\times 1$ vector ${\bar{x}}_{t}$ on a connected surface $S_{t}$. Simultaneously, $N_{r}$ observation points are collected at locations represented by the $3N_{r}\times 1$ vector ${\bar{x}}_{r}$ on the connected output surface $S_{r}$. The objective is to compute the mutual information between the input RF system $\left({\bar{\psi }}_{\mathrm{in}},S_{t}\right)$ and the output RF system $\left({\bar{J}}_{r},S_{r}\right)$ when the Gaussian noise $\bar{w}$ is set to zero; i.e., we are interested in the mutual information between two RFs that is completely due to intrinsic fluctuations in the RFs circulating in a deterministic EM system. The conventional Shannon mutual information $I[{\bar{x}}_{\mathrm{out}}^{\left(r\right)}:{\bar{x}}_{\mathrm{in}}^{\left(r\right)}]$ can be identified with the quantity defined in Definition A14 and is expressed as follows:

(139) $$I\left[{\bar{x}}_{\mathrm{out}}^{\left(r\right)}:{\bar{x}}_{\mathrm{in}}^{\left(r\right)}\right]=I_{{\bar{J}}_{r}{\bar{\psi }}_{\mathrm{in}}}^{N_{t},N_{r}}\left({\bar{x}}_{t}:{\bar{x}}_{r};S_{t}:S_{r}\right).$$

The proof of (139) is straightforward and is therefore omitted here.

If the EM system is deterministic, the calculations in (139) coincide with the entropy of ${\bar{x}}_{\mathrm{out}}^{\left(r\right)}$, which is identical to the entropy of ${\bar{x}}_{\mathrm{in}}^{\left(r\right)}$.^19^ It is important to note that, in the Gaussian case, the mutual information between the input and output RFs on arbitrary input and output manifolds is fully solvable for deterministic EM systems. In this specific example, the mutual information result primarily depends on the input information field statistics and is independent of the EM GFs. The more interesting problem arises in the context of stochastic EM systems, where the situation is markedly different. To the best of our knowledge, determining the pdf of the received current under Gaussian illumination remains an open problem.^20^ As a result, mutual information in this case can only be estimated through Monte Carlo simulations or other numerical approximation methods.

Consider as an example the case where the KLEs of the system are truncated to a finite number of terms. In the case of Gaussian input information and EM GF RFs, the final value for mutual information will depend on (1) the mean, covariance, and pseudo-covariance functions of the input field and GF RFs, (2) the deterministic details of the EM GFs, including the manifolds $S_{b},b\in \left\{t,s,r\right\}$, and (3) the pdf of the polynomial (132). Therefore, despite the progress made in our EM-RFT at multiple levels, when it comes to mutual information the final result still cannot be expressed analytically in terms of the EM GFs and their statistics, even though the highly nontrivial form (132) is derived here for the first time. This motivates our introduction of what we term the “fundamental problem of EIT” in Section 5.1 as a promising direction for future research.

## 5. Conclusions and Future Work

### 5.1. Summary and Final Remarks: Universal Structures in the RFT of EM Information

This paper addressed two fundamental types of systems: deterministic and stochastic information transmission systems. Deterministic systems were characterized by four basic EM GFs, whereas stochastic systems required five fundamentally distinct GFs—three associated with the transmitting, scattering, and receiving systems, and two with the channel medium. A comprehensive description was provided of how EM fields and currents, modeled as frequency-dependent spatial RFs, were modified as they propagated through these systems. In deterministic EM systems, all EM GFs were deterministic, with randomness arising solely from the input information RF. In stochastic EM systems, the five EM GFs themselves exhibited stochasticity, resulting in an interaction between two distinct sources of randomness: the externally supplied information fields injected by users and the inherent randomness of the system, as captured by the GFs.

The paper aimed not only to identify and highlight universal themes in the conceptual and formal structure of EM information in modern information transmission systems but also to provide a completely general mathematical framework—an explicit formal apparatus—for computing and simulating second-order RFs in generic communication and other physical systems. This apparatus utilized as input the mean and covariance functions of (1) the input information RF and (2) all the EM RF GFs of the system, subsequently enabling the simulation of all RFs via the KL expansion. This approach avoided the computationally prohibitive method of employing MC techniques with repetitive solutions of deterministic full-wave EM to calculate ensemble averages and moments. More importantly, the proposed method inherently accounted for the correlation and mutual coupling between all components (polarization degrees of freedom) of the RFs involved, as this coupling was embedded within the formalism and mathematical constructs of the EM-RFT itself.

All mathematical formulas derived in this paper were presented either in closed-analytical form or as integrals involving only compact integration intervals and well-behaved (non-singular) kernels, making the theory readily implementable on digital computing platforms. In other words, with a sufficient number of quadrature points, all integrals could be accurately approximated by sums or, ultimately, by matrix/vector multiplication operations, which can be efficiently executed on specialized hardware such as GPUs and many-core CPUs. Consequently, this theory offers both fundamental theoretical and computational applications and can serve as a foundation for multiple research directions in the field of EIT.

Given the essential role of mean, covariance, and pseudo-covariance functions in EM-RFT and information theory, it is crucial to establish a robust procedure for tracking the propagation of input second-order correlation information through the information transmission system to the output. By employing the response function method, trace formulas were derived to decouple input correlation data from output correlation data effectively. This approach revealed that the relationship between input and output correlation functions could be expressed in terms of higher-order correlation propagators, or correlation GFs. Using these trace formulas, complex interactions between different polarizations were fully captured, mutual coupling effects were comprehensively accounted for, while the formalism was valid for both near- and far-field regions. In other words, a primary contribution of the EM-RFT developed in this work was the derivation and presentation of a significant simplification in the structure of correlation phenomena within general EM information systems. Specifically, the trace formulas in Theorem 4 revealed the most general and universal abstract structures underlying *all* EM information systems.

One of the notable features of the proposed RFT of EM information is its ability to precisely determine the universal structure of received RFs in completely generic stochastic EM information transmission systems. For arbitrarily shaped transmitter, scattering, and receiver systems, with fully generic EM GFs, we found that the received signal can be expressed as a polynomial of several RVs, where each monomial involves at most six RVs for the NLoS component and four RVs for the LoS component. Based on this analysis, the fundamental problem of EIT can be stated as follows: Given a completely generic stochastic EM information transmission system (see Definition 4), determine the pdf of the received current distribution at a generic point. Specifically, find the pdf of the complex-valued random vector ${\bar{J}}_{r}\left(x_{0}\right)$ for any fixed $x_{0}\in S_{r}$. Interestingly, we have demonstrated that this problem is equivalent to a related mathematical problem under the assumption that the KLE can be truncated:

**The Fundamental Problem of EIT:** Let the KLE of all RFs in a standard stochastic EM information transmission system be truncated to *N* terms. Then, estimating the pdf of a component of the received current RF at any fixed point on the receiving system is equivalent to addressing the following problem (the general stochastic structure of the received RF in standard stochastic EM information systems; see Corollary 3): Find the pdf of the polynomial

$$\sum_{l}^{N}\beta X_{l}Y_{l}Z_{l}W_{l}L_{l}V_{l}+\sum_{l}^{N}\gamma X_{l}W_{l}L_{l}V_{l},$$

where the coefficients β and γ are complex, and X,Y,Z,W,L,V are zero-mean, mutually independent real RVs. RVs within the same family (indexed by *l*) are uncorrelated. If all the RVs above are Gaussian, we say that the fundamental problem of EIT is in its standard form.

To the best of our knowledge, the problem of finding the pdf of sums of products of RVs has not yet been solved analytically in general. Therefore, it must be addressed numerically in order to compute quantities of interest, such as entropy, mutual information, and capacity.

### 5.2. Future Work

The research presented in this paper establishes a foundation for EIT within the broader field of EM-RFT, opening several potential avenues for further exploration. One natural extension of this work is to implement concrete, specialized systems for numerical computations. In particular, future research could focus on estimating mean and input covariance data using measurements or specialized computational models. The apparatus developed in this paper could then be applied to simulate various RFs and compute mutual information.

Nevertheless, much additional theoretical and foundational work remains to be conducted. For example, the following directions outline potential avenues for future research to further expand upon.

1. While the continuity condition on the mean and covariance functions provides a robust criterion, it could be further relaxed in future work, albeit at the expense of increasing the complexity of the theoretical framework. Additionally, many of the regularity conditions discussed could be optimized or refined to improve their applicability and precision.
2. Future investigations could explore additional modes of convergence that are stronger or weaker than m.s. convergence. Options such as almost sure convergence, convergence in probability, and convergence in distribution offer promising opportunities for enhancing the theoretical foundations of EM-RFT.
3. The scope of the stochastic integrals could also be expanded to include more complex forms, such as Wiener and Itô integrals. This would require randomizing the geometries of antennas and scatterers, potentially introducing new dynamics into the system. Notably, by treating the GF defined on a manifold $S_{b}$ as an RF, we have already undertaken an indirect form of geometric randomization. However, future work could focus on generalizing the EM GF method to accommodate more complex stochastic environments, presenting a promising avenue for further research.
4. Additionally, a spectral theory of EM RFs could be developed to capture the wavevector-frequency-domain structure of information transmission systems within a general stochastic framework. Such an extension would enable a deeper understanding of the interactions within these systems. However, currently the development of such spectral theory is hindered by the lack of sufficient symmetry in EM-RFT, mainly absence of homogeneity and isotropy.
5. Thus far, we have developed the correlation propagation theory of EM RFs under the assumption that information is injected via the input information RF ${\bar{\psi }}_{\mathrm{in}}$, as is typical in engineering applications such as communication and information processing systems. However, for certain advanced applications, it may also be of interest to analyze how correlation and pseudo-correlation data propagate through a stochastic EM system when input information is injected via one or more of the five EM RF GFs. While the methods developed in this paper are expected to remain applicable in such cases, the complexity and length of the analysis would likely increase significantly.
6. A significant advancement would be to develop the entire EM-RFT in the time domain, transitioning from the current space–frequency theory in (x,ω) to a framework based on (x,t). This shift would offer new insights and facilitate more comprehensive modeling of dynamic systems as they evolve over time, thereby enriching the overall understanding of EM information transmission.
7. Moreover, using operator norms and their identities to derive upper bounds on information measures, as outlined in [125], can provide fundamental insights. These bounds are essential for understanding the interaction between physics—specifically ETT—and information in practical systems. They also play a crucial role in guiding the optimization and design of such systems.

The author hopes that the publication of the present work will stimulate others to work along some or all of these promising research directions.

## Figures and Tables

**Table 1:** Definition of various geometric spaces used in this paper. Here, b∈{t,s,r} and the notations $S^{b}$ and $S_{b}$ are synonymous. The symbol ≈ means isomorphic in any given coordinate system.

Space	Description
$TS_{x}^{b}$	The tangent space of $S_{b}$ based at $x\in S_{b}$. Here, $TS_{x}^{b}\approx R^{2}$.
$TS^{b}$	The tangent bundle of $S_{b}$, defined as $TS^{b}:={⋃}_{x\in S_{b}}\left(x,TS_{x}^{b}\right)$.
$\tilde{T}S_{x}^{b}$	The complexified tangent space of $S_{b}$ based at $x\in S_{b}$. Here, $\tilde{T}S_{x}^{b}\approx C^{2}$.
$\tilde{T}S^{b}$	The complexified tangent bundle of $S_{b}$, defined as $\tilde{T}S^{b}:={⋃}_{x\in S_{b}}\left(x,\tilde{T}S_{x}^{b}\right)$.
$DS_{x,x^{\prime }}^{b}$	The complexified dyad space of $S_{b}$, based at $\left(x,x^{\prime }\right)\in S_{b}\times S_{b},$ defined as the set of maps $g:TS^{b}\times TS^{b}\to C$. Here $DS_{x,x^{\prime }}^{b}\approx C^{2\times 2}$.
$DS_{b}$	The complexified dyad bundle of $S_{b}$, defined as $DS_{b}:={⋃}_{\left(x,x^{\prime }\right)\in S_{b}\times S_{b}}\left(x,x^{\prime },DS_{x,x^{\prime }}^{b}\right)$.
$DS_{b}^{2\times 2}$	The complexified superdyad bundle of $S_{b}$, defined as $DS_{b}:={⋃}_{\left(x,x^{\prime }\right)\in S_{b}\times S_{b}}\left(x,x^{\prime },{\left[DS_{x,x^{\prime }}^{b}\right]}_{2\times 2}\right)$, where ${\left[DS_{x,x^{\prime }}^{b}\right]}_{2\times 2}$ is a superdyad based at $\left(x,x^{\prime }\right)\in S_{b}\times S_{b}$.
$V_{s}^{b}$	The space $\left\{\bar{f}|\bar{f}:S\subset R^{3}\to \tilde{T}S^{b}\right\}$ of complex-valued surface vector fields on the 2-manifolds $S_{b}$. Examples include $J_{e}^{b}$ or $J_{m}^{b}$.
$V_{v}$	The space $\left\{\bar{f}|\bar{f}:V\subset R^{3}\to C^{3}\right\}$ of complex-valued 3-dimensional vector fields on $V:=R^{3}\setminus {⋃}_{b}V_{b}\times R^{+}$. Examples are $E_{\mathrm{rad}}$ or $H_{s}$.
$D_{s}^{b}$	The space $\left\{\bar{\bar{f}}|\bar{\bar{f}}:S\subset R^{3}\to DS_{b}\right\}$ of complex-valued surface dyadic fields on the 2-manifolds $S_{b}$. Examples are ${\bar{F}}_{\mathrm{ee}}^{b}$ or ${\bar{F}}_{\mathrm{em}}^{b}$.
$D_{v}$	The space $\left\{\bar{\bar{f}}|\bar{\bar{f}}:V\subset R^{3}\to C^{3\times 3}\right\}$ of complex-valued 3-dimensional dyadic fields on $V:=R^{3}\setminus {⋃}_{b}V_{b}\times R^{+}$. Examples include ${\bar{G}}_{\mathrm{ee}}$ or ${\bar{G}}_{\mathrm{mm}}$.

**Table 2:** The domain and range of all deterministic Cartesian field quantities in EM-RFT. Here, b∈{t,s,r} and d∈{ee,em,me,mm}.

Deterministic Field	Domain	Codomain
$e\left(x,\omega \right)\in V_{s}^{t}$	$S_{t}\times R^{+}$	$C^{2}$
$h\left(x,\omega \right)\in V_{s}^{t}$	$S_{t}\times R^{+}$	$C^{2}$
$E_{\mathrm{rad}}\left(x,\omega \right)\in V_{v}$	$\left(R^{3}\setminus {⋃}_{b}V_{b}\right)\times R^{+}$	$C^{3}$
$H_{\mathrm{rad}}\left(x,\omega \right)\in V_{v}$	$\left(R^{3}\setminus {⋃}_{b}V_{b}\right)\times R^{+}$	$C^{3}$
$E_{s}\left(x,\omega \right)\in V_{v}$	$\left(R^{3}\setminus {⋃}_{b}V_{b}\right)\times R^{+}$	$C^{3}$
$H_{s}\left(x,\omega \right)\in V_{v}$	$\left(R^{3}\setminus {⋃}_{b}V_{b}\right)\times R^{+}$	$C^{3}$
$J_{b}\left(x,\omega \right)\in V_{s}^{b}$	$S_{b}\times R^{+}$	$C^{2}$
${\bar{F}}_{d}^{b}\left(x,x^{\prime },\omega \right)\in D_{s}^{b}$	$S_{b}\times R^{+}$	$C^{2\times 2}$
${\bar{G}}_{d}\left(x,x^{\prime },\omega \right)\in D_{v}$	$\left(R^{3}\setminus {⋃}_{b}V_{b}\right)\times {⋃}_{b}S_{b}\times R^{+}$	$C^{3\times 3}$
${\hat{\alpha }}_{i}\left(x\right)\in {⋃}_{b}TS_{x}^{b},i\in \left\{1,2\right\}$	${⋃}_{b}S_{b}$	$R^{3}$

**Table 3:** The domain and range of all higher-order superarrays (supervectors, superdyads) used in this paper. The definitions of spaces such as $\tilde{T}S^{b}$ and $DS_{b}^{2\times 2}$ are given in Table 1. Here, b∈{t,s,r} and $b^{\prime }\in \left\{t,s\right\}$.

Field	Domain	Codomain
${\bar{J}}_{b}\left(x,\omega \right)$	$S_{b}\times R^{+}$	$\tilde{T}S^{b}\times \tilde{T}S^{b}\approx C^{4}$
${\bar{\psi }}_{\mathrm{in}}\left(x,\omega \right)$	$S_{t}\times R^{+}$	$\tilde{T}S^{t}\times \tilde{T}S^{t}\approx C^{4}$
${\bar{\psi }}_{\mathrm{rad}}\left(x,\omega \right)$	$\left(R^{3}\setminus {⋃}_{b}V_{b}\right)\times R^{+}$	$C^{6}$
${\bar{\psi }}_{s}\left(x,\omega \right)$	$\left(R^{3}\setminus \;{⋃}_{b}V_{b}\right)\times R^{+}$	$C^{6}$
${\bar{\bar{F}}}_{b}\left(x,x^{\prime },\omega \right)$	$S_{b}\times S_{b}\times R^{+}$	$DS_{b}^{2\times 2}\approx C^{4\times 4}$
$\bar{\bar{G}}\left(x,x^{\prime },\omega \right)$	$\left(R^{3}\setminus {⋃}_{b}V_{b}\right)\times {⋃}_{b}S_{b}\times R^{+}$	$C^{6\times 6}$
${\bar{\bar{G}}}_{b^{\prime }}\left(x,x^{\prime },\omega \right)$	$\left(R^{3}\setminus {⋃}_{b}V_{b}\right)\times S_{b^{\prime }}\times R^{+}$	$C^{6\times 6}$

**Table 4:** Important function sets used in this paper.

Description	Symbol	Definition
Input RFs	I	$\left\{{\bar{\psi }}_{\mathrm{in}}\right\}$
EM GFs	W	$\left\{{\bar{\bar{F}}}_{b},{\bar{\bar{G}}}_{b^{\prime }}|b\in \left\{t,s,r\right\},b^{\prime }\in \left\{t,s\right\}\right\}$
Response RFs	Q	$\left\{{\bar{J}}_{b},{\bar{\psi }}_{c}|b\in \left\{t,s,r\right\},c\in \left\{\mathrm{rad},s\right\}\right\}$
EM RFs	V	I∪W∪Q∪V

**Table 5:** All stochastic response functions derived in this paper arranged in ascending order of complexity. Indices run as i,j∈{1,2,3,4}.

Response Function	Name	Type
${\bar{\bar{B}}}^{\mathrm{los}}$	LoS response function	2-point Green’s function
${\bar{\bar{B}}}^{\mathrm{nlos}}$	NLoS response function	2-point Green’s function
${\bar{\bar{K}}}_{ij}^{\mathrm{nlos}}$	Reduced NLoS correlation propagator	2-point Green’s function
${\bar{\bar{K}}}_{ij}^{\mathrm{los}}$	Reduced LoS correlation propagator	2-point Green’s function
${\bar{\bar{K}}}_{ij}^{\mathrm{nlos}/\mathrm{los}}$	Reduced NLoS/LoS correlation propagator	2-point Green’s function
${\bar{\bar{K}}}_{ij}^{\mathrm{los}/\mathrm{nlos}}$	Reduced LoS/NLoS correlation propagator	2-point Green’s function
${\bar{\bar{K}}}_{ij}$	Reduced total correlation propagator	2-point Green’s function
${\bar{\bar{K^{\prime }}}}_{ij}^{\mathrm{nlos}}$	Reduced NLoS pseudo-correlation propagator	2-point Green’s function
${\bar{\bar{K^{\prime }}}}_{ij}^{\mathrm{los}}$	Reduced LoS pseudo-correlation propagator	2-point Green’s function
${\bar{\bar{K^{\prime }}}}_{ij}^{\mathrm{nlos}/\mathrm{los}}$	Reduced NLoS/LoS pseudo-correlation propagator	2-point Green’s function
${\bar{\bar{K^{\prime }}}}_{ij}^{\mathrm{los}/\mathrm{nlos}}$	Reduced LoS/NLoS pseudo-correlation propagator	2-point Green’s function
${\bar{\bar{K^{\prime }}}}_{ij}$	Reduced total pseudo-correlation propagator	2-point Green’s function
${\bar{\bar{S}}}_{\mathrm{los}}$	Stochastic LoS system response function	4-point Green’s function
${\bar{\bar{S}}}_{\mathrm{nlos}}$	Stochastic NLoS system response function	6-point Green’s function
${\bar{\bar{P}}}_{ij}^{\mathrm{los}}$	Stochastic LoS correlation propagator	8-point Green’s function
${\bar{\bar{P}}}_{ij}^{\prime \mathrm{los}}$	Stochastic LoS pseudo-correlation propagator	8-point Green’s function
${\bar{\bar{P}}}_{ij}^{\mathrm{los}/\mathrm{nlos}}$	Stochastic LoS/NLoS correlation propagator	10-point Green’s function
${\bar{\bar{P}}}_{ij}^{\mathrm{nlos}/\mathrm{los}}$	Stochastic NLoS/LoS correlation propagator	10-point Green’s function
${\bar{\bar{P}}}_{ij}^{\prime \mathrm{los}/\mathrm{nlos}}$	Stochastic LoS/NLoS pseudo-correlation propagator	10-point Green’s function
${\bar{\bar{P}}}_{ij}^{\prime \mathrm{nlos}/\mathrm{los}}$	Stochastic NLoS/LoS pseudo-correlation propagator	10-point Green’s function
${\bar{\bar{P}}}_{ij}^{\mathrm{nlos}}$	Stochastic NLoS correlation propagator	12-point Green’s function
${\bar{\bar{P}}}_{ij}^{\prime \mathrm{nlos}}$	Stochastic NLoS pseudo-correlation propagator	12-point Green’s function

**Table 6:** Regularity conditions for Theorem 2 (deterministic EM system).

Component	Quantity	Regularity Condition	Singularity Behavior
Input RF	${\bar{\psi }}_{\mathrm{in}}\left(x,\omega ,\varpi \right)$	Continuous mean on $S_{t}$; continuous covariance on $S_{t}\times S_{t}$.	Nonsingular (input field defined only on smooth compact manifold $S_{t}$).
Medium GF	$\bar{\bar{G}}\left(x,x^{\prime },\omega \right)$	Piecewise continuous and bounded for $\left(x,x^{\prime }\right)\in V_{\mathrm{ex}}\times S$, with $S={⋃}_{b}S_{b}$.	**Singular only at** $x=x^{\prime }$; otherwise smooth (e.g., vacuum GF is $C^{\infty }$ for $x\neq x^{\prime }$).
Current GFs	${\bar{\bar{F}}}_{b}\left(x,x^{\prime },\omega \right),\;b\in \left\{t,s,r\right\}$	Can be approximated arbitrarily well by smooth kernels (Theorem A7).	**Nonsingular for all** $x,x^{\prime }\in S_{b}$; continuous even when $x=x^{\prime }$.
Domains	$S_{b}$ (transmit/scatter/receive), $V_{\mathrm{ex}}$	$S_{b}$ are compact, closed 2-manifolds; $V_{\mathrm{ex}}=R^{3}\setminus {⋃}_{b}V_{b}$ excludes diagonal $x=x^{\prime }$ for medium GFs.	No singularities introduced by domain geometry.

**Table 7:** Regularity conditions for Theorem 3 (standard stochastic EM system).

Component	Quantity	Regularity Condition	Singularity Behavior
Input RF	${\bar{\psi }}_{\mathrm{in}}\left(x,\omega ,\varpi \right)$	Continuous mean, covariance, and pseudo-covariance on $S_{t}\times S_{t}$.	Nonsingular by definition on smooth manifold $S_{t}$.
Stochastic GFs	${\bar{\bar{F}}}_{b}\left(x,x^{\prime },\omega ,\varpi \right),\;b\in \left\{t,s,r\right\}$	Mean, covariance, and pseudo-covariance continuous on $S_{b}\times S_{b}$.	**Nonsingular for all** $x,x^{\prime }\in S_{b}$; continuity guaranteed even at $x=x^{\prime }$ by smooth approximation (Theorem A7).
Stochastic GFs	${\bar{\bar{G}}}_{b^{\prime }}\left(x,x^{\prime },\omega ,\varpi \right),\;b^{\prime }\in \left\{t,s\right\}$	Mean, covariance, and pseudo-covariance continuous on $V_{\mathrm{ex}}\times S_{b^{\prime }}$.	**Singular only at** $x=x^{\prime }$; excluded from $V_{\mathrm{ex}}$, ensuring m.s. integrability.
Independence	—	All five stochastic GFs are mutually stochastically independent.	Not applicable.
Domains	$S_{b}$, $V_{\mathrm{ex}}$	Same compactness and smoothness as deterministic case.	No geometric singularities.

**Table 8:** Reduction of the 128 correlation/pseudo-correlation propagators under common physical assumptions. Each row specifies a condition and its effect on the eight propagator types. The symbol “≡” denotes functional equality; “0” denotes identically zero. The symbol • denotes any of the eight propagator types (los, nlos, los/nlos, nlos/los, and their pseudo-counterparts).

Physical Assumption	Effect on Propagators	Resulting Count
Proper input field and proper GFs	All pseudo-propagators vanish: $P_{ij}^{\prime •}\equiv 0$	64 (correlation only)
Reciprocal medium (linear, passive, time-invariant)	$\bar{\bar{G}}\left(x,x^{\prime },\omega \right)={\bar{\bar{G}}}^{⊤}\left(x^{\prime },x,\omega \right)$	
	$\Rightarrow \;P_{ij}^{\mathrm{los}/\mathrm{nlos}}={\left(P_{ji}^{\mathrm{nlos}/\mathrm{los}}\right)}^{\;*}$	Cross-terms halved; 96 independent
Identical Tx/Rx ports (e.g., full-duplex monostatic)	$P_{ij}^{\mathrm{los}}=P_{ij}^{\mathrm{nlos}}$, $P_{ij}^{\mathrm{los}/\mathrm{nlos}}=P_{ij}^{\mathrm{nlos}/\mathrm{los}}$	Further reduction (problem-dependent)
Scalar (co-polarized) system: only $E_{x}$–$E_{x}$ coupling	Off-diagonal indices vanish: $P_{ij}^{•}\equiv 0$ for i≠j	16 (4 diagonal index pairs × 4 propagator types)
All of the above combined	Only $P_{ii}^{\mathrm{los}}$ and $P_{ii}^{\mathrm{nlos}}$ survive; all pseudo-propagators zero	8 non-zero propagators

## Data Availability

No data were used in conducting the study presented in this paper. An earlier draft of this research was posted on [www.techrxiv.org] [https://www.techrxiv.org/users/875564/articles/1255540-a-random-field-theory-of-electromagnetic-information-the-response-function-approach], 5 January 2025.

## Notes

1 All will be rigorously defined below.

2 Nevertheless, we believe that IFT warrants greater attention within the field of information theory.

3 For example, full-wave methods such as the Finite-Element Method (FEM) [65], the Finite-Difference Time-Domain (FDTD) method [66], and the Method of Moments (MoM) [67,68,69] are commonly used. However, these methods demand substantial 3D mesh discretization, making them particularly challenging when applied to large-scale problems, especially in scenarios requiring multiple iterations for statistical analysis.

4 Numerical integrals involving EM GFs within our EM-RFT framework are relatively straightforward to compute, as they require integration over compact domains with integrands that are consistently continuous, often smooth, and occasionally analytic.

5 See also Section 2.2 for more remarks on the EM frequency-domain model.

6 The reason behind this is that only fields tangential to the surface $S_{t}$ produce radiating currents since current GFs are based on EM boundary conditions and the latter are formulated such that they involve tangential components only; see [44,45,69,93,104,105]

7 See Appendix A for the definition of a supervector.

8 In some scenarios, information can be injected into the communications system through the scattering cluster $S_{s}$, e.g., via an RIS. However, if this is the case one may just remove that part of $S_{s}$ where information is injected into the system and include it as a (passive) part of the transmit array $S_{t}$ itself. Therefore, and without loss of generality, information is injected mainly through the $S_{t}$ system.

9 See Appendix A for the definition of a superdyad.

10 See Appendix C.

11 See Appendix A.

12 The derivation in [88] assumed a mixed perfect electric conductor and dielectric object system, but the general structure of the response function given in (11) is very general and applies for all linear EM boundary conditions such as the generalized impedance boundary condition. In this paper, we consider the EM boundary condition a black box whose input–output relation is given by relations of the form (12).

13 In engineering electromagnetics, these types of dyadic GFs are extensively used for building computational full-wave analysis models of devices and systems comprising a combination of PEC and dielectric boundary conditions, e.g., see [65,67,68,70,110].

14 Recall that for simplicity we do not label the unit vectors ${\hat{\alpha }}_{i}\left(x\right)$ with *b* when $x\in S_{b}$.

15 See Appendix G for definition of this type of invariance.

16 Here, without loss of generality, we assume that both the electric and magnetic fields in ${\bar{\psi }}_{\mathrm{in}}$ are excited by the same random variable. This assumption is realistic because, in Maxwell’s EMT, a time-varying electric field generates a time-varying magnetic field and vice versa, ensuring both fields are excited simultaneously [44,56,93]. However, if the need arises for the rather uncommon scenario where electric and magnetic fields are excited differently, we can apply the principle of superposition to two different pairs of electric and magnetic fields.

17 See the proof of Theorem 8.

18 The special case examined in [19] did not utilize Definition A14, which, to the best of our knowledge, is introduced here for the first time. Instead, the approximation hierarchy in [19] directly addressed the Shannon capacity of a Gaussian channel, whose general form is well established. Conceptually, however, there is some similarity between that approach and the one proposed here, as both aim to capture the entropy content associated with a finite number of degrees of freedom in a field, followed by taking the limit as the number of degrees of freedom becomes large. This process bears resemblance to the thermodynamic limit in quantum field theory and statistical mechanics [80].

19 From Corollary 2, we know that if the input information RF is Gaussian, all induced RFs are Gaussian as well. It is straightforward to observe that the input and output vectors ${\bar{x}}_{\mathrm{in}}$ and ${\bar{x}}_{\mathrm{out}}$, as defined in Equation (81), are jointly Gaussian. Furthermore, their real-vector representations, ${\bar{x}}_{\mathrm{in}}^{\left(r\right)}$ and ${\bar{x}}_{\mathrm{out}}^{\left(r\right)}$, are also jointly Gaussian, with the pdf of the entire input–output joint vector fully determined by the mean and covariance data at the input along with the EM GFs.

20 See also Appendix Q for a partial literature review and Section 5.1 for our assessment.

## Appendix A. Mathematical Notation and Background

The formalism developed in this paper necessitates meticulous attention to mathematical notation as it integrates concepts and structures from diverse disciplines in mathematics, engineering, and physics. Developing a robust mathematical theory of EM information requires working with an extensive array of data, necessitating various mathematical operations on these arrays. In the following sections, we provide a concise summary of the mathematical notations employed throughout this paper.

Before beginning, we emphasize a significant potential source of confusion in this paper. In EM-RFT, it is unavoidable to combine EM quantities with terms from probability theory. This requires using ω to represent frequency, while in probability theory, the same symbol denotes an element of the sample or state space Ω. To avoid ambiguity, we introduce the notation ϖ∈Ω for the latter case. This notational convention is consistently applied throughout the paper by adhering to the following rule:
We emphasize that a quantity is an RF or random variable by consistently displaying explicit notational dependence on ϖ∈Ω.
Accordingly, a random variable is denoted as X(ϖ), a generic RF is represented as g(x,ϖ), and a frequency-dependent EM RF is expressed as g(x,ω,ϖ), where $x\in R^{d}$ and $\omega \in R^{+}$.

We now explain all of the mathematical data types dealt with in EM-RFT. We start with the two fundamental types of Cartesian arrays utilized in this paper:

1. *Vectors*: $a=\sum_{i=1}^{3}a^{i}{\hat{x}}_{i}$, where ${\hat{x}}_{i},i=1,2,3,$ are three orthonormal unit vectors forming the basis for $R^{3}$. Here, $a^{i}\in K$, where K is either R or C.
2. *Dyads*: $\bar{a}=\sum_{i=1}^{3}\sum_{j=1}^{3}a^{ij}{\hat{x}}_{i}{\hat{x}}_{j}$, where ${\hat{x}}_{i}{\hat{x}}_{j}$ is a shorthand notation for ${\hat{x}}_{i}⊗{\hat{x}}_{j}$, the tensor product of ${\hat{x}}_{i}$ and ${\hat{x}}_{j}$, and $a^{ij}\in K$. Since for a given coordinate system each dyad is equivalent to a 3×3 matrix, we often write $\bar{a}\in K^{3\times 3}$.

The dot product operation between two vectors $a\cdot b=\sum_{i=1}^{3}a^{i}b^{i}$ is extended to dyads in the usual way. We define (ab)·c:=a(b·c) and c·(ab):=(c·a)b, and extend the definition to combinations like ${\hat{x}}_{i}{\hat{x}}_{j},i,j=1,2,3,$ through the linearity of the dot product operations.

In order the simplify the analysis of EM structures, we introduce two additional Cartesian structures:

1. *Supervectors*: $\bar{a}={\left[\begin{array}{ccc} a_{1} & \ldots & a_{N} \end{array}\right]}^{⊤}$, which is an N×1 array of Cartesian vectors with $a_{i}\in K^{3}$. We note that ultimately $\bar{a}\in K^{3N\times 1}$.
2. *Superdyads*: here we define
  (A1) $$\bar{\bar{a}}=\left[\begin{array}{ccc} {\bar{a}}_{11} & \ldots & {\bar{a}}_{1N}\\ \vdots & \vdots & \vdots \\ {\bar{a}}_{N1} & \ldots & {\bar{a}}_{NN} \end{array}\right],$$
  which is an N×N array of Cartesian dyads with ${\bar{a}}_{ij}\in K^{3\times 3}$ for i,j=1,…,N. Note that eventually we have $\bar{\bar{a}}\in K^{3N\times 3N}$.
3. *Ordinary arrays*: To simplify the notation, we avoid introducing specialized symbols to distinguish supervectors and superdyads from other types of arrays. The following should be noted:
  - Ordinary vectors are denoted as $\bar{a}$ and ordinary matrices as $\bar{\bar{a}}$.
  - An exception is covariance/correlation and pseudo-covariance/correlation matrices, which we write as $C_{g},R_{g}$ and $C_{g}^{\prime },R_{g}^{\prime }$ for an RF *g*, respectively.
  - Another exception arises with the EM dyads ${\bar{G}}_{d}$, where d∈{ee,em,me,mm}. While these are 3×3 Cartesian arrays, they are *not* denoted as ${\bar{\bar{G}}}_{d}$ to align with the mainstream conventions in EMT.
  - It is important to note that operations involving ordinary arrays are defined differently from those involving supervectors and superdyads (see Remark A1).

In this paper, the vast majority of the supervectors and superdyads we deal with are 2×1 and 2×2 arrays of vectors and dyads, respectively, leading to $\bar{a}\in K^{6\times 1}$ and $\bar{\bar{a}}\in K^{6\times 6}$.

**Remark** **A1.**
*Careful attention must be paid to array multiplication operations involving supervectors and superdyads. In general, the matrix–vector product $\bar{\bar{a}}\;\bar{b}$ and matrix–matrix product $\bar{\bar{a}}\;\bar{\bar{b}}$ are defined as usual in linear algebra. However, when multiplying supervectors and/or superdyads, we explicitly insert a dot product operation, such as in $\bar{\bar{a}}\cdot \bar{b}$ and $\bar{\bar{a}}\cdot \bar{\bar{b}}$. This notation indicates that we are multiplying the Cartesian vector and dyadic entries of $\bar{a}$ and $\bar{\bar{a}}$, respectively, rather than the elements $a^{i}$ and $b^{ij}$, etc. For example, the notation $\bar{\bar{a}}\cdot \bar{b}$ signifies that $\bar{\bar{a}}$ is a superdyad, $\bar{b}$ is a supervector, and the array multiplication is that of a 2×2 array of dyads with a 2×1 array of vectors, with each individual multiplication operation performed according to the dyad/vector dot product rule. A similar process take place with the operation $\bar{\bar{a}}\cdot \bar{\bar{b}}$ involving multiplying two superdyads. On the other hand, when writing $\bar{\bar{a}}\;\bar{b}$ and $\bar{\bar{a}}\;\bar{\bar{b}}$, then the corresponding operations will be those associated with the full arrays of dimensions $K^{3N\times 1}$ and $K^{3N\times 3N}$, respectively, as usual in matrix algebra.*

In addition to the mathematical notation issues above, the following general theorem is used in this paper whose proof can be found in [87,111,112].

**Theorem** **A1** (Continuity under the integral sign)**.** 
*Let (X,F,μ) be a measure space and let T be a topological space. Consider a function f:X×T→R such that:*

1. *For every t∈T, the function x↦f(x,t) is in $L^{1}\left(\mu \right)$ with respect to F.*
2. *For almost every x∈X, the function t↦f(x,t) is continuous on T.*
3. *There exists an integrable function $g\in L^{1}\left(\mu \right)$ such that |f(x,t)|≤g(x) for all t∈T and almost every x∈X.*

*Then, for $t_{0}\in T$,*

(A2)
$$\lim_{t\to t_{0}}\int_{X}f\left(x,t\right)\;d\mu \left(x\right)=\int_{X}\lim_{t\to t_{0}}f\left(x,t\right)\;d\mu \left(x\right).$$

A list of all abbreviations used in this paper is provided in Table A1.

arrange items alphatically:

**Table A1:** List of all abbreviations used in this paper.

Abbreviation	Meaning
EIT	Electromagnetic Information Theory
EM	Electromagnetic/Electromagnetics/Electromagnetism
EM-RFT	Electromagnetic Random Field Theory
EMT	Electromagnetic Theory
GF	Green’s Function
KL	Karhunen–Loève
KLE	Karhunen–Loève Expansion
LoS	Line of Sight
MC	Monte Carlo
MIMO	Multiple-Input Multiple-Output
m.s.	Mean square
NF	Near Field
NLoS	Non-Line-of-Sight
pdf	Probability density function
RF	Random Field
RFT	Random Field Theory
RV	Random Variable
SISO	Single-Input Single-Output

## Appendix B. Real Vector-Valued Random Fields

Here, we provide a quick overview of vector-valued RFs, i.e., RFs taking values in a *q*-dimensional Euclidean space $R^{q},q\in N$. While the theory of scalar RFs and their Karhunen–Loève expansions is well known [115,126], the literature on vector RFs is scarce. Some of the references discussing the topic include [127,128,129,130]. In what follows, we provide a resume of the minimum needed machinery required to understand the stochastic theory developed in the main text.

Let $L^{2}\left(M,R^{q}\right)$ be the space of square-integrable functions on the set $M\subset R^{d}$, where q,d∈N. In most applications, *M* can be given the structure of a smooth manifold or, more generally, a pointwise smooth manifold [115]. The linear space $L^{2}\left(M,R^{q}\right)$ is equipped with a real inner product $\langle \bar{u},\bar{v}\rangle$, for any two vector fields $\bar{u},\bar{v}:M\to R^{q}$, defined by:

(A3) $${\langle \bar{u}\left(\bar{x}\right),\bar{v}\left(\bar{x}\right)\rangle }_{L^{2}\left(M,R^{q}\right)}:=\int_{\bar{x}\in M}\bar{u}{\left(\bar{x}\right)}^{⊤}\bar{v}\left(\bar{x}\right)d^{d}x,$$

where ⊤ denotes the transpose operation. Note that each vector field $\bar{u}\left(\bar{x}\right)\in L^{2}\left(M,R^{q}\right)$ can be written as

(A4) $$\bar{u}={\left[\begin{array}{cccc} u_{1}\left(\bar{x}\right) & u_{2}\left(\bar{x}\right) & \cdots & u_{q}\left(\bar{x}\right) \end{array}\right]}^{⊤},$$

with $u_{i}\left(\bar{x}\right)\in L^{2}\left(M,R\right)$.

**Definition** **A1** (Vector RF)**.** 
*Let (Ω,F,P) be a complete probability space, where *Ω* is the state space (or set of outcomes), F is a σ-algebra of events, and P is the associated probability measure. Let $M\subset R^{d}$ be a d-dimensional domain. A d-dimensional vector-valued RF $\bar{f}$ is a measurable map $\bar{f}:\Omega \to L^{2}\left(M,R^{q}\right)$, where d,q∈N.*

If the domain of the vector RF *M* has dimension *d* while its range has dimension *q*, we say that the dimension of the vector RF is (d,q). We can further emphasize this information by expressing it in forms such as $\left(M,R^{q}\right)$, $\left(R^{d},R^{q}\right)$, etc., depending on the context. For example, a vector-valued random process (where the domain is, for example, time) has type $\left(R,R^{q}\right)$. If needed, we emphasize the randomness of a given vector field $\bar{f}$ by writing it as $\bar{f}\left(\bar{x},\varpi \right)$, where ϖ∈Ω. The symbol ϖ should not be confused with the radian frequency ω.

To work with RFs in EIT, we need to consider mean and covariance functions (defined below) that satisfy minimum regularity conditions, as required by second-order RFT. An appropriate function space for this purpose is $L_{P}^{2}\left[\Omega ,L^{2}\left(M,R^{q}\right)\right]$, defined as follows.

**Definition** **A2.**
*$L_{P}^{2}\left[\Omega ,L^{2}\left(M,R^{q}\right)\right]$ is the space of all strongly measurable functions $\bar{f}:\Omega \to L^{2}\left(M,R^{q}\right)$, whose inner product is defined by the formula*

(A5)
$$\begin{array}{cc} \langle \bar{u}\left(\bar{x},\varpi \right), & \bar{v}\left(\bar{x},\varpi \right)\rangle_{L_{P}^{2}\left[\Omega ,L^{2}\left(M,R^{q}\right)\right]}:=\int_{\varpi \in \Omega }{\langle \bar{u}\left(\bar{x}\right),\bar{v}\left(\bar{x}\right)\rangle }_{L^{2}\left(M,R^{q}\right)}dP(\varpi ), \end{array}$$

*for all $\bar{u},\bar{v}\in L_{P}^{2}\left[\Omega ,L^{2}\left(M,R^{q}\right)\right]$.*

A fundamental assumption in the EM-RFT developed in this paper is that all RFs satisfy at least the following regularity condition:

(A6) $$\bar{f}\left(\bar{x},\varpi \right)\in L_{P}^{2}\left[\Omega ,L^{2}\left(M,R^{q}\right)\right].$$

However, note that EM RFs can meet stricter regularity conditions, including various levels of differentiability. A detailed examination of such natural definitions is provided in [127]. In the main text, we impose stronger conditions than mere square integrability, such as continuity of the mean and covariance functions. A comprehensive investigation of these conditions and related issues, however, lies beyond the scope of this paper.

**Definition** **A3** (Mean and covariance functions)**.** 
*Consider a vector RF $\bar{u}\left(\bar{x},\varpi \right)\in L_{P}^{2}$$\left[\Omega ,L^{2}\left(M,R^{q}\right)\right]$. The mean of $\bar{u}$, denoted by $\mu_{\bar{u}}$, is defined as $E\{\bar{u}\left(\bar{x},\varpi \right)\}$ where*

(A7)
$$E\left\{\bar{u}\left(\bar{x},\varpi \right)\right\}:={\left[\begin{array}{ccc} E\{u_{1}\left(\bar{x},\varpi \right)\} & \cdots & E\{u_{q}\left(\bar{x},\varpi \right)\} \end{array}\right]}^{⊤}.$$

*The covariance of the two vector RFs $\bar{u}\left(\bar{x},\varpi \right)$ and $\bar{v}\left(\bar{x},\varpi \right)\in L_{P}^{2}\left[\Omega ,L^{2}\left(M,R^{q}\right)\right]$, denoted by $\mathrm{Cov}[\bar{u}\left(\bar{x},\varpi \right),\bar{v}\left(\bar{x},\varpi \right)]$, generates a q×q matrix whose general structure is given by Formula *(A8)*.*

(A8)
$$\mathrm{Cov}\left[\bar{u}\left(\bar{x},\varpi \right),\bar{v}\left(\bar{x},\varpi \right)\right]:=\left(\begin{array}{cccc} \mathrm{Cov}[u_{1}\left(\bar{x},\varpi \right),v_{1}\left(\bar{x},\varpi \right)] & \mathrm{Cov}[u_{1}\left(\bar{x},\varpi \right),v_{2}\left(\bar{x},\varpi \right)] & \cdots & \mathrm{Cov}[u_{1}\left(\bar{x},\varpi \right),v_{q}\left(\bar{x},\varpi \right)]\\ \mathrm{Cov}[u_{2}\left(\bar{x},\varpi \right),v_{1}\left(\bar{x},\varpi \right)] & \mathrm{Cov}[u_{2}\left(\bar{x},\varpi \right),v_{2}\left(\bar{x},\varpi \right)] & \cdots & \mathrm{Cov}[u_{2}\left(\bar{x},\varpi \right),v_{q}\left(\bar{x},\varpi \right)]\\ \vdots & \vdots & \vdots & \vdots \\ \mathrm{Cov}[u_{q}\left(\bar{x},\varpi \right),v_{1}\left(\bar{x},\varpi \right)] & \mathrm{Cov}[u_{q}\left(\bar{x},\varpi \right),v_{2}\left(\bar{x},\varpi \right)] & \cdots & \mathrm{Cov}[u_{q}\left(\bar{x},\varpi \right),v_{q}\left(\bar{x},\varpi \right)] \end{array}\right).$$

*Each element of the q×q covariance matrix is defined by*

(A9)
$$\begin{array}{c} \mathrm{Cov}\left[u_{i}\left(\bar{x},\varpi \right),v_{j}\left(\bar{x},\varpi \right)\right]:=E\left\{\left[u_{i}\left(\bar{x},\varpi \right)-E\left\{u_{i}\left(\bar{x},\varpi \right)\right\}\right]\left[v_{j}\left(\bar{x},\varpi \right)-E\left\{v_{j}\left(\bar{x},\varpi \right)\right\}\right]\right\}, \end{array}$$

*for i,j=1,2,…,q.*

**Proposition** **A1.**
*Under the conditions of Definition A3, it can be shown that*

(A10)
$$\begin{array}{c} E\left\{\bar{u}\left(\bar{x},\varpi \right)\right\}\in L^{2}\left(M,R^{q}\right),\\ \mathrm{Cov}\left[u_{i}\left(\bar{x},\varpi \right),v_{j}\left(\bar{x},\varpi \right)\right]\in L^{2}\left(M\times M,R\right),\\ \mathrm{Cov}\left[\bar{u}\left(\bar{x},\varpi \right),\bar{v}\left(\bar{x},\varpi \right)\right]\in L^{2}\left(M\times M,R^{q\times q}\right). \end{array}$$

The space of q×q-matrix-valued fields $L^{2}\left(M\times M,R^{q\times q}\right)$ is equipped with the inner product

(A11) $$\begin{array}{c} {\langle {\bar{\bar{B}}}_{1},{\bar{\bar{B}}}_{2}\rangle }_{L^{2}\left(M\times M,R^{q\times q}\right)}=\int_{\bar{x}\in M}\int_{\bar{y}\in M}1_{q}^{⊤}{\bar{\bar{B}}}_{1}\left(\bar{x},\bar{y}\right)\circ {\bar{\bar{B}}}_{2}\left(\bar{x},\bar{y}\right)1_{q}d^{d}xd^{d}y, \end{array}$$

for any ${\bar{\bar{B}}}_{1}\left(\bar{x},\bar{y}\right),{\bar{\bar{B}}}_{2}\left(\bar{x},\bar{y}\right)\in L^{2}\left(M\times M,R^{q\times q}\right)$, where $1_{q}$ is an q×1 vector of ones and ‘∘’ is the Hadamard product. It can be shown that if ${\left\{{\bar{\varphi }}_{n}\left(\bar{x}\right)\right\}}_{n\in N}$ is a complete orthonormal basis for $L^{2}\left(M,R^{q}\right)$, then ${\left\{{\bar{\varphi }}_{n}\left(\bar{x}\right){\bar{\varphi }}_{m}^{⊤}\left(\bar{x}\right)\right\}}_{n,m\in N}$ is a complete orthonormal basis for $L^{2}\left(M\times M,R^{q\times q}\right)$.^21^ This provides a direct method for constructing bases for the covariance matrix fields of vector RFs, an important process in the analysis of concrete examples.

We now address the topic of establishing the Karhunen–Loève expansion for vector RFs [53,131].

**Definition** **A4** (Covariance operator of a second-order RF)**.** 
*Let $\bar{u}$ be a second-order RF as above. Consider the operator*

(A12)
$$C_{\bar{u}}:L^{2}\left(M,R^{q}\right)\to L^{2}\left(M,R^{q}\right),$$

*defined by the Hilbert–Schmidt integral operator expression:*

(A13)
$$C_{\bar{u}}\left[\bar{v}\right]\left(\bar{x}\right):=\int_{M}\mathrm{Cov}\left[\bar{u}\left(\bar{x},\varpi \right),\bar{u}\left(\bar{y},\varpi \right)\right]\bar{v}\left(\bar{y}\right)\;d^{d}y,$$

*for any $\bar{v}\left(\bar{x}\right)\in L^{2}\left(M,R^{q}\right)$. The operator $C_{\bar{u}}$ is called the covariance operator associated with the RF $\bar{u}$.*

**Theorem** **A2** (Properties of the covariance operator)**.** 
*The covariance operator *(A13)* is a non-negative, symmetric, trace-class operator [127]. Consequently, it admits a set of orthonormal eigenfunctions ${\left\{{\bar{\varphi }}_{n}\left(\bar{x}\right)\right\}}_{n\in N}\in L^{2}\left(M,R^{q}\right)$, with eigenvalues $\lambda_{1}\geq \lambda_{2}\geq \cdots \geq 0$, satisfying*

(A14)
$$C_{\bar{u}}\left[{\bar{\varphi }}_{n}\right]\left(\bar{x}\right)=\lambda_{n}{\bar{\varphi }}_{n}\left(\bar{x}\right).$$

*In fact, the set ${\left\{{\bar{\varphi }}_{n}\left(\bar{x}\right)\right\}}_{n\in N}$ forms a complete orthonormal basis in the sense that for any $\bar{v}\left(\bar{x}\right)\in L^{2}\left(M,R^{q}\right)$, the following expansion holds:*

(A15)
$$\bar{v}\left(\bar{x}\right)=\sum_{n\in N}\lambda_{n}{\langle \bar{v}\left(\bar{x}\right),{\bar{\varphi }}_{n}\left(\bar{x}\right)\rangle }_{L^{2}\left(M,R^{q}\right)}{\bar{\varphi }}_{n}\left(\bar{x}\right).$$

The Karhunen–Loève expansion theorem extends the deterministic vector-valued function expansion in (A15) to the case of vector-valued RFs.

**Theorem** **A3** (The Karhunen–Loève expansion)**.** 
*Let $\bar{f}\left(\bar{x},\varpi \right)\in L_{P}^{2}\left[\Omega ,L^{2}\left(M,R^{q}\right)\right]$ be a vector RF. The Karhunen–Loève expansion (KLE) of this RF is given by the following series:*

(A16)
$$\bar{f}\left(\bar{x},\varpi \right)=E\left\{\bar{f}\left(\bar{x},\varpi \right)\right\}+\sum_{n\in N}\sqrt{\lambda_{n}}X_{n}\left(\varpi \right){\bar{\varphi }}_{n}\left(\bar{x}\right),$$

*where*

(A17)
$$X_{n}\left(\varpi \right)=\int_{M}{\left[\bar{f}\left(\bar{x},\varpi \right)-E\left\{\bar{f}\left(\bar{x},\varpi \right)\right\}\right]}^{⊤}{\bar{\varphi }}_{n}\left(\bar{x}\right)\;d^{d}x$$

*are random variables satisfying*

(A18)
$$E\left\{X_{n}\left(\varpi \right)X_{m}\left(\varpi \right)\right\}=\delta_{nm},\;\;E\left\{X_{n}\left(\varpi \right)\right\}=0,$$

*for all n,m∈N, where $\delta_{nm}$ is the Kronecker delta function.*

The integral (A17) can be defined as a stochastic integral, for example in the sense of Definition A5. The convergence speed of expansions (A15) and (A16) depends on the regularity properties of the covariance matrix of the RF [127].

The KLE enjoys a key optimality property in the sense that it is the best expansion that can be obtained when truncating to a finite number of terms. In order to understand the following theorem, we note that the following Hilbert space isomorphisms hold^22^:

(A19) $$L^{2}\left(M,R^{q}\right)⊗L_{P}^{2}\left(M,R\right)\approx L_{P}^{2}\left[\Omega ,L^{2}\left(M,R^{q}\right)\right],$$

(A20) $$H_{N}⊗L_{P}^{2}\left(M,R\right)\approx L_{P}^{2}\left[\Omega ,H_{N})\right].$$

**Theorem** **A4** (Optimality of the Karhunen–Loève expansion)**.** 
*Let $H_{N}\subset L^{2}\left(M,R^{q}\right)$ be a finite-dimensional subspace with dimension N. The following operator*

(A21)
$$P_{H_{N}⊗L_{P}^{2}\left(M,R\right)}:L^{2}\left(M,R^{q}\right)⊗L_{P}^{2}\left(M,R\right)\to H_{N}⊗L_{P}^{2}\left(M,R\right)$$

*is an orthogonal projection operator. Consider the RF*

(A22)
$$\bar{u}\left(\bar{x},\varpi \right)\in L^{2}\left(M,R^{q}\right)⊗L_{P}^{2}\left(M,R\right),$$

*with $E\{\bar{u}\left(\bar{x},\varpi \right)\}=0$. Then the following holds:*

(A23)
$$\begin{array}{c} \underset{\begin{array}{c} H_{N}\subset L^{2}\left(M,R^{q}\right)\\ \dim(H_{N})=N \end{array}}{\inf}{∥\bar{u}\left(\bar{x}\right)-P_{H_{N}⊗L_{P(M,R)}^{2}}\bar{u}\left(\bar{x}\right)∥}_{L^{2}\left(M,R^{q}\right)⊗L_{P}^{2}\left(M,R\right)}={\left(\sum_{n=N+1}^{\infty }\lambda_{n}\right)}^{\frac{1}{2}}, \end{array}$$

*where the infimum is achieved only when $H_{N}=\mathrm{span}\left\{{\bar{\varphi }}_{1},{\bar{\varphi }}_{2},\ldots ,{\bar{\varphi }}_{N}\right\}$.*

We now turn to some of the geometric properties of RFs. We assume the reader is familiar with mean square (m.s.) convergence of sequences of random variables, e.g., the concepts of m.s. continuity (see [33,43,114,115] for definitions related to scalar RFs). An *n*-dimensional vector RF $\bar{f}\left(\bar{x}\right)$ is m.s. continuous, m.s. differentiable, etc., if and only if each component function $f^{i}\left(\bar{x}\right)$, i=1,…,n, is m.s. continuous, m.s. differentiable, etc.

The following theorem provides a powerful method for establishing m.s. continuity and differentiability.

**Theorem** **A5** (Condition for m.s. continuity)**.** 
*Let $f(\bar{x},\varpi )$ be a real-valued vector RF defined on a domain $V\subset R^{n}$. The field is m.s. continuous at ${\bar{x}}_{0}\in V$ if and only if*

1. *The mean function $\bar{x}\mapsto E\left\{f(\bar{x},\varpi )\right\}$ is continuous at ${\bar{x}}_{0}\in V$.*
2. *The covariance function $\left(\bar{x},{\bar{x}}^{\prime }\right)\mapsto C_{f}\left(\bar{x},{\bar{x}}^{\prime }\right)$ is continuous at $\left(\bar{x},{\bar{x}}^{\prime }\right)=\left({\bar{x}}_{0},{\bar{x}}_{0}\right)$.*

Next, we consider m.s. integration, a simple and efficient method for constructing new RFs from existing ones. Note that while m.s. integration is well known within the context of random processes (RF with dimension (1,1), they are less known for RFs, especially vector real fields of generic order (d,q). In what follows, we provide the most general definition followed by few key theorems pertinent to constructions of EM RFs to be developed throughout this work.

**Definition** **A5** (Mean square integration)**.** 
*Let $D\subset R^{n}$ and $V\subset R^{r}$ be two domains where n,r∈N. Let $f(\bar{x},\varpi )$ be a real-valued vector RF defined on D. Consider a real-valued deterministic function $K\left(\bar{x},\bar{y}\right),\bar{x}\in V,\bar{y}\in D$. Assume that for every $\bar{x}\in V$, $K(\bar{x},\bar{y})$ is bounded and piecewise continuous for all $\bar{y}\in D$. We say that the integral*

(A24)
$$g\left(\bar{x}\right)=\int_{\bar{y}\in D}d^{n}yK\left(\bar{x},\bar{y}\right)f\left(\bar{y},\varpi \right)$$

*exists in the m.s. sense if the limit of the sum*

(A25)
$$g_{m}\left(\bar{x},\varpi \right)=\sum_{i=1}^{m}K\left(\bar{x},{\bar{y}}_{i}\right)f\left({\bar{y}}_{i},\varpi \right)\Delta {\bar{y}}_{i}$$

*exists for $m\overset{m.s.}{\to }\infty$. The quantity $\Delta {\bar{y}}_{i}$ in *(A25)* is an infinitesimal volume in $R^{n}$ and it is understood that the measure of the largest volume of $\Delta {\bar{y}}_{i},i=1,\ldots ,m,$ goes to zero as m→∞. The above limit is called the m.s. integral of the RF $f(\bar{y},\varpi )$ relative to the kernel function $K(\bar{x},\bar{y})$. We write $g\left(\bar{x},\varpi \right)=\lim_{m\underset{m.s.}{\to }\infty }g_{m}\left(\bar{x}\right),$ in which it is said that the RF $f(\bar{y},\varpi )$ is integrable in the m.s. sense.*

The key point behind Definition A5 is the following. If the mean-square integral of a given RF exists, we can assert that, relative to a suitably well-behaved kernel function, an RF $f(\bar{x},\varpi )$ defined on a domain $D\subset R^{n}$ that is mean-square integrable, as per Definition A5, yields a new RF $g(\bar{x},\varpi )$ on the domain *V*. Then m.s. integration allows one to produce new RFs from given ones.

The following proposition provides a useful method to compute the mean and covariance of the new RF in terms of the corresponding data of the original field.

**Proposition** **A2** (Mean and covariance of integrated random fields)**.** 
*Consider a real-valued vector RF $g(\bar{x},\varpi )$ obtained from another real-valued RF $f(\bar{x},\varpi )$ through a process of stochastic integration relative to a kernel function $K(\bar{x},\bar{y})$ as per Definition A5. The mean and covariance of $g(\bar{x},\varpi )$ can be expressed as follows. The mean of $g(\bar{x},\varpi )$ is given by:*

(A26)
$$E\left\{g\left(\bar{x},\varpi \right)\right\}=\int_{D}K\left(\bar{x},\bar{y}\right)E\left\{f\left(\bar{y},\varpi \right)\right\}\;d^{n}y.$$

*The covariance of $g(\bar{x},\varpi )$ is given by:*

(A27)
$$C_{g}\left(\bar{x},{\bar{x}}^{\prime }\right)=\int_{D}\int_{D}K\left(\bar{x},\bar{y}\right)K\left({\bar{x}}^{\prime },{\bar{y}}^{\prime }\right)C_{f}\left(\bar{y},{\bar{y}}^{\prime }\right)\;d^{n}y\;d^{n}y^{\prime }.$$

*Here, $C_{f}\left(\bar{y},{\bar{y}}^{\prime }\right)$ denotes the covariance.*

The following key theorem provides a necessary and sufficient condition to efficiently determine whether a given RF is m.s. integrable relative to a given kernel function.

**Theorem** **A6** (Condition of stochastic integrability)**.** 
*Let $f(\bar{x},\varpi )$ be a complex RF and $K(\bar{x},\bar{y})$ a complex kernel function defined on $\bar{x}\in V\subset R^{r}$ and $\bar{y}\in D\subset R^{n}$. The field $f(\bar{x},\varpi )$ is said to be m.s. integrable on V, in the sense of Definition A5, if and only if*

(A28)
$$\int_{D}d^{n}x^{\prime }\;K\left(\bar{x},{\bar{x}}^{\prime }\right)E\left\{f({\bar{x}}^{\prime })\right\}<\infty ,$$

(A29)
$$\int_{D}\int_{D}d^{n}y\;d^{n}y^{\prime }\;K\left(\bar{x},\bar{y}\right)K^{*}\left(\bar{x},{\bar{y}}^{\prime }\right)C_{f}\left(\bar{y},{\bar{y}}^{\prime }\right)<\infty ,$$

*for all $\bar{x}\in V$.*

Note that for the study of m.s. integrals of complex-valued RFs, only the correlation properties are needed, while the pseudo-correlation or pseudo-covariance are not required.

Definitions and results related to stochastic integration, including Definition A5 and Theorems A2 and A6, are elaborated in [43,54,114]. These developments build on foundational results on RFs, e.g., see [53,131,132,133,134].

## Appendix C. Representations of Surface Fields: Current Distributions and Current Green’s Functions

We present several key aspects regarding the description of field behavior on 2-manifolds, such as $S_{t},S_{s},S_{r}$. For additional background on the surface current distributions and dyadic surface current GFs utilized throughout this paper, refer to [40,41,42,45,88].

Locally, the surface current distributions $J_{e}^{b}\left(x,\omega \right)$ and $J_{m}^{b}\left(x,\omega \right)$ may be expanded in terms of the local tangent space bases (3) as follows:

(A30) $$\begin{array}{c} J_{e}^{b}\left(x\right)={\hat{\alpha }}^{1}\left(x\right)J_{e}^{b,1}\left(x\right)+{\hat{\alpha }}^{2}\left(x\right)J_{e}^{b,2}\left(x\right),\;J_{m}^{b}\left(x\right)={\hat{\alpha }}^{1}\left(x\right)J_{m}^{b,1}\left(x\right)+{\hat{\alpha }}^{2}\left(x\right)J_{m}^{b,2}\left(x\right), \end{array}$$

or more compactly in terms of non-Cartesian bases:

(A31) $${\bar{J}}_{b}\left(x,\omega \right)=\left[\begin{array}{c} J_{e}^{b,1}\left(x,\omega \right)\\ J_{e}^{b,2}\left(x,\omega \right)\\ J_{m}^{b,1}\left(x,\omega \right)\\ J_{m}^{b,2}\left(x,\omega \right) \end{array}\right]\in C^{4},\;x\in S_{b},\;b\in \left\{t,s,r\right\}.$$

An alternative representation is the following:

(A32) $${\bar{J}}_{b}\left(x,\omega \right)=\left[\begin{array}{cc} J_{e}^{b,1}\left(x,\omega \right) & J_{e}^{b,2}\left(x,\omega \right)\\ J_{m}^{b,1}\left(x,\omega \right) & J_{m}^{b,2}\left(x,\omega \right) \end{array}\right]\left[\begin{array}{c} {\hat{\alpha }}^{1}\left(x\right)\\ {\hat{\alpha }}^{2}\left(x\right) \end{array}\right],\;x\in S_{b}.$$

In other words, given a local choice for the tangent system ${\hat{\alpha }}^{s}\left(x_{i}\right),s=1,2,$ on the three manifolds $S_{t}$, $S_{s}$, and $S_{r}$, the complete information about the surface current distributions on the transmitter, scatterers, and the receiver can be captured by the three complex 2×2 matrices appearing in (A32).

The current GFs can be locally represented in the following form:

(A33) $${\bar{F}}_{b}\left(x,x^{\prime },\omega \right)=\sum_{i=1}^{2}\sum_{j=1}^{2}{\hat{\alpha }}_{i}{\hat{\alpha }}_{j}F_{ij}\left(x,x^{\prime },\omega \right),$$

for b∈{t,s,r}. Note that there also exists a 3D dyadic representation of (A33) in the form [41]

(A34) $${\bar{F}}_{b}\left(x,x^{\prime },\omega \right)=\sum_{i=1}^{3}\sum_{j=1}^{3}{\hat{x}}_{i}{\hat{x}}_{j}F_{ij}^{3D}\left(x,x^{\prime },\omega \right),$$

for b∈{t,s,r}. Transformations between the 2D and 3D forms (A33) and (A34) are possible. In this paper, we focus on 2D representations as they provide the minimal complexity needed to understand the basic EM structure of the problem. In contrast, 3D representations of current GFs contain some redundant information.

The following theorem is fundamental in the theory of EM current GFs (for a proof, see [41,88]):

**Theorem** **A7** (Smooth approximation of current Green’s functions)**.** 
*Consider a current component $\bar{J}\left(x,\omega \right)$ obtained in terms of a current GF $\bar{\bar{F}}\left(x,x^{\prime },\omega \right)$ and input field $\bar{E}\left(x,\omega \right)$ as follows:*

(A35)
$$\bar{J}\left(x,\omega \right)=\int_{x^{\prime }\in S}d^{2}x^{\prime }\;\bar{\bar{F}}\left(x,x^{\prime },\omega \right)\cdot \bar{E}\left(x^{\prime },\omega \right).$$

*Then there exists a a sequence of functions ${\bar{\bar{F}}}_{l}\left(x,x^{\prime },\omega \right),l\in N,$ approximating the GF $\bar{\bar{F}}\left(x,x^{\prime },\omega \right)$ in the sense that the following two conditions are satisfied:*

1. *${\bar{\bar{F}}}_{l}\left(x,x^{\prime },\omega \right)$ is smooth for all $x,x^{\prime }\in S,\;l\in N$.*
2. $\bar{J}\left(x,\omega \right)=\lim_{l\to \infty }\int_{x^{\prime }\in S}d^{2}x^{\prime }\;{\bar{\bar{F}}}_{l}\left(x,x^{\prime },\omega \right)\cdot \bar{E}\left(x^{\prime },\omega \right).$

*The limit in condition (2) above can be defined by a proper current and current GF norm.*

Based on the decompositions (6), we can split each surface field into sums as follows:

(A36) $$\begin{array}{c} {\bar{\psi }}_{\mathrm{in}}\left(x,\omega \right)=\sum_{n=1}^{N_{t}}{\bar{\psi }}_{\mathrm{in}}^{n}\left(x,\omega \right),\;{\bar{J}}_{b}\left(x,\omega \right)=\sum_{n=1}^{N_{b}}{\bar{J}}_{b}^{n}\left(x,\omega \right), \end{array}$$

for b∈{t,s,r}. Here, ${\bar{\psi }}_{\mathrm{in}}^{n}\left(x,\omega \right):={\bar{\psi }}_{\mathrm{in}}\left(x,\omega \right)U\left(x,S_{t}^{n}\right)$ and ${\bar{J}}_{b}^{n}\left(x,\omega \right):={\bar{J}}_{b}\left(x,\omega \right)U\left(x,S_{b}^{n}\right)$, where

(A37) $$U\left(x,S\right):=\left\{\begin{array}{c} 1,\;\;\;\;x\in S,\\ 0,\;\;\;\;x\notin S. \end{array}\right.$$

Note that the surface current distribution supervector ${\bar{J}}_{b}\left(x,\omega \right)$ in the expansion (A36) already incorporates EM mutual coupling effects. In terms of the surface current GF, mutual coupling can be further clarified by expanding it into a sum of no-mutual-coupling and mutual-coupling terms, as follows:

(A38) $${\bar{F}}_{b}\left(x,x^{\prime },\omega \right)=\sum_{p=1}^{N_{b}}{\bar{F}}_{p,0}^{b}\left(x,x^{\prime },\omega \right)+\sum_{p=1}^{N_{b}}\delta {\bar{F}}_{p}^{b}\left(x,x^{\prime },\omega \right)+\sum_{p,q=1,\;p\neq q}^{N_{b}}\delta {\bar{F}}_{pq}^{b}\left(x,x^{\prime },\omega \right).$$

The first term on the RHS of (A38), ${\bar{F}}_{p,0}^{b}\left(x,x^{\prime },\omega \right)$, represents the isolated *p*th element’s surface current GF associated with $S_{n}^{b}$ (excluding mutual coupling effects) and is nonzero only for $\left(x,x^{\prime }\right)\in S_{b}^{p}\times S_{b}^{p}$. The second term on the RHS, $\delta {\bar{F}}_{p}^{b}\left(x,x^{\prime },\omega \right)$, accounts for the self-interaction mutual coupling GF of the *p*th element, and is also nonzero only for $\left(x,x^{\prime }\right)\in S_{b}^{p}\times S_{b}^{p}$. The third term, $\delta {\bar{F}}_{pq}^{b}\left(x,x^{\prime },\omega \right)$, represents the cross-interaction GFs, quantifying the mutual coupling GF effect of the *q*th element on the *p*th element, and is nonzero only for $\left(x,x^{\prime }\right)\in S_{b}^{p}\times {⋃}_{n=1,n\neq p}^{N_{b}}S_{b}^{p}$. For more information on the analysis of mutual coupling using GFs, see [45,60,135,136,137,138,139].

## Appendix D. Electromagnetic Dyadic Green’s Functions in the Frequency Domain

In the frequency domain, all dynamic quantities vary sinusoidally in time as exp(−iωt) and all fields and currents become complex [93]. The general form of a frequency-domain EM dyadic GF $\bar{G}\left(x,x^{\prime },\omega \right)$ is the following

(A39) $$\bar{G}\left(x,x^{\prime },\omega \right)=\sum_{i,j=1}^{3}{\hat{x}}_{i}{\hat{x}}_{j}G^{ij}\left(x,x^{\prime },\omega \right),$$

where as usual $x=\sum_{i}^{3}{\hat{x}}_{i}x^{i}$ and ${\hat{x}}_{i}{\hat{x}}_{j}$ is a tensor (dyadic) product of the two Cartesian orthonormal vectors ${\hat{x}}_{i}$ and ${\hat{x}}_{j}$. Note that $G^{ij}={\hat{x}}_{j}\cdot \bar{G}\cdot {\hat{x}}_{i}$. Fix a Fourier mode exp(ik·x−iωt), where k is the wavevector. Then the dispersion relation of the vacuum is |k|=k, where k=ω/c and *c* is the speed of light [140]. The electric field/electric current dyadic GF is given by [109]

(A40) $${\bar{G}}_{\mathrm{ee}}^{0}\left(x,x^{\prime },\omega \right)=\gamma_{\omega }\frac{1}{ikR}\left(\bar{I}-\hat{R}⊗\hat{R}\right)e^{ikR}-\gamma_{\omega }\left[\frac{1}{{\left(ikR\right)}^{2}}-\frac{1}{{\left(ikR\right)}^{3}}\right]\left(\bar{I}-3\hat{R}⊗\hat{R}\right)e^{ikR},$$

where $\gamma_{\omega }:=-\mu_{0}\omega k/4\pi ,R:=x-x^{\prime },\hat{R}:=R/\left|R\right|$. The 3-dimensional tensor $\bar{I}$ is the unit dyad. The radiated field can be expressed in terms of this GF using the formula [44]:

(A41) $$E\left(x,\omega \right)=\int_{x^{\prime }\in S_{t}}d^{2}x^{\prime }\;{\bar{G}}_{\mathrm{ee}}^{0}\left(x-x^{\prime },\omega \right)\cdot J_{e}\left(x^{\prime },\omega \right),$$

where we are interested in the exterior region $R^{3}\setminus S_{t}∋x$ but the integration is performed over the entire surface $S_{t}∋x^{\prime }$. Expressions for the remaining EM dyadic GFs can be found in [109].

## Appendix E. Reciprocity in Electromagnetic Media and Its Implication for the Superdyadic Green Function

In linear, time-invariant media obeying Lorentz reciprocity, the electromagnetic field satisfies fundamental symmetry constraints. These constraints manifest as specific relations among dyadic Green functions that connect source currents to radiated fields. To formulate these relations in full generality, we introduce the superdyadic Green function that maps electric and magnetic current sources to the resulting electric and magnetic fields. Following standard electromagnetic theory, this object is defined as

(A42) $$\bar{\bar{G}}\left(x,x^{\prime },\omega \right)=\left[\begin{array}{cc} {\bar{G}}_{\mathrm{ee}}\left(x,x^{\prime },\omega \right) & {\bar{G}}_{\mathrm{em}}\left(x,x^{\prime },\omega \right)\\ {\bar{G}}_{\mathrm{me}}\left(x,x^{\prime },\omega \right) & {\bar{G}}_{\mathrm{mm}}\left(x,x^{\prime },\omega \right) \end{array}\right],$$

where ${\bar{G}}_{\mathrm{ee}}$ and ${\bar{G}}_{\mathrm{mm}}$ are 3×3 dyadics representing electric-field–electric-current and magnetic-field–magnetic-current responses, respectively, while ${\bar{G}}_{\mathrm{em}}$ and ${\bar{G}}_{\mathrm{me}}$ describe cross-coupling between electric and magnetic sources.

For a reciprocal medium—characterized by symmetric constitutive tensors $\bar{\varepsilon }={\bar{\varepsilon }}^{⊤}$, $\bar{\mu }={\bar{\mu }}^{⊤}$, and vanishing magneto-electric coupling—Lorentz reciprocity implies the following set of symmetry relations [109]:

(A43a) $$\begin{array}{cc} {\bar{G}}_{\mathrm{ee}}\left(x,x^{\prime },\omega \right) & ={\bar{G}}_{\mathrm{ee}}^{⊤}\left(x^{\prime },x,\omega \right), \end{array}$$

(A43b) $$\begin{array}{cc} {\bar{G}}_{\mathrm{mm}}\left(x,x^{\prime },\omega \right) & ={\bar{G}}_{\mathrm{mm}}^{⊤}\left(x^{\prime },x,\omega \right), \end{array}$$

(A43c) $$\begin{array}{cc} {\bar{G}}_{\mathrm{em}}\left(x,x^{\prime },\omega \right) & =-\;{\bar{G}}_{\mathrm{me}}^{⊤}\left(x^{\prime },x,\omega \right). \end{array}$$

These identities reflect the fact that the electric and magnetic subsystems are individually self-reciprocal, while the cross-coupling terms are anti-reciprocal due to the dual structure of Maxwell’s equations. The three relations (A43a)–(A43c) can be compactly expressed using the signature superdyadic $\bar{\bar{\Omega }}$ in (A47). The unified reciprocity condition then reads

(A44) $$\bar{\bar{G}}\left(x,x^{\prime },\omega \right)=\bar{\bar{\Omega }}\cdot {\bar{\bar{G}}}^{⊤}\left(x^{\prime },x,\omega \right)\cdot \bar{\bar{\Omega }}$$

since $\bar{\bar{\Omega }}$ is symmetric. Equation (A44) is the most general form of reciprocity for the full electromagnetic Green function operator. It holds for any reciprocal medium, regardless of inhomogeneity or anisotropy, provided the constitutive tensors are symmetric.

## Appendix F. Reciprocity Theorem for the Antenna Current Green Function

In linear, time-invariant antenna systems embedded in reciprocal media, the antenna current Green function (ACGF) exhibits fundamental symmetry relations that govern the response of the antenna to external excitations. This section establishes these relations in their most general form.

**Theorem** **A8** (ACGF Reciprocity)**.** 
*Consider an antenna system embedded in a linear, time-invariant medium satisfying Lorentz reciprocity. The medium is characterized by symmetric constitutive tensors $\bar{\bar{\varepsilon }}={\bar{\bar{\varepsilon }}}^{⊤}$ and $\bar{\bar{\mu }}={\bar{\bar{\mu }}}^{⊤}$, with no magneto-electric coupling. These conditions are satisfied by both lossy and lossless reciprocal media. For such a system, the dyadic components of the antenna current Green function satisfy the following symmetry relations:*

(A45a)
$$\begin{array}{cc} {\bar{F}}_{\mathrm{ee}}\left(x,x^{\prime }\right) & ={\bar{F}}_{\mathrm{ee}}^{⊤}\left(x^{\prime },x\right), \end{array}$$

(A45b)
$$\begin{array}{cc} {\bar{F}}_{\mathrm{mm}}\left(x,x^{\prime }\right) & ={\bar{F}}_{\mathrm{mm}}^{⊤}\left(x^{\prime },x\right), \end{array}$$

(A45c)
$$\begin{array}{cc} {\bar{F}}_{\mathrm{em}}\left(x,x^{\prime }\right) & =-\;{\bar{F}}_{\mathrm{me}}^{⊤}\left(x^{\prime },x\right). \end{array}$$

*The superdyadic ACGF is defined as*

(A46)
$$\bar{\bar{F}}\left(x,x^{\prime }\right)=\left[\begin{array}{cc} {\bar{F}}_{\mathrm{ee}}\left(x,x^{\prime }\right) & {\bar{F}}_{\mathrm{em}}\left(x,x^{\prime }\right)\\ {\bar{F}}_{\mathrm{me}}\left(x,x^{\prime }\right) & {\bar{F}}_{\mathrm{mm}}\left(x,x^{\prime }\right) \end{array}\right].$$

*Defining the signature superdyadic*

(A47)
$$\bar{\bar{\Omega }}=\left[\begin{array}{cc} \bar{I} & 0\\ 0 & -\bar{I} \end{array}\right],$$

*where $\bar{I}$ denotes the 3×3 identity dyadic and 0 the zero dyadic; the reciprocity relations take the compact form*

(A48)
$$\bar{\bar{F}}\left(x,x^{\prime }\right)=\bar{\bar{\Omega }}\cdot {\bar{\bar{F}}}^{⊤}\left(x^{\prime },x\right)\cdot {\bar{\bar{\Omega }}}^{⊤}.$$

*Note that ${\bar{\bar{\Omega }}}^{⊤}=\bar{\bar{\Omega }}$ since it is symmetric.*

**Proof.** The proof proceeds by applying the Lorentz reciprocity theorem to two distinct excitation states of the antenna system and expressing the reactions in terms of the ACGF. Note that the proof holds for any reciprocal medium, regardless of losses, since the Lorentz reciprocity theorem requires only symmetric constitutive tensors. Consider two arbitrary incident field distributions, denoted State *A* and State *B*, with corresponding induced currents:

(A49) $$\begin{array}{cc} \mathrm{State}\;A: & \;\left\{E_{\mathrm{inc}}^{a}\left(x\right),H_{\mathrm{inc}}^{a}\left(x\right)\right\}\to \left\{J_{e}^{a}\left(x\right),J_{m}^{a}\left(x\right)\right\}, \end{array}$$

(A50) $$\begin{array}{cc} \mathrm{State}\;B: & \;\left\{E_{\mathrm{inc}}^{b}\left(x\right),H_{\mathrm{inc}}^{b}\left(x\right)\right\}\to \left\{J_{e}^{b}\left(x\right),J_{m}^{b}\left(x\right)\right\}. \end{array}$$

According to the Lorentz reciprocity theorem for surface sources and fields on *S*, the reaction of field *A* on source *B* equals the reaction of field *B* on source *A*:

(A51) $$\begin{array}{c} {\;\int \int \;}_{S}d^{2}x\;\left[E_{\mathrm{inc}}^{a}\left(x\right)\cdot J_{e}^{b}\left(x\right)-H_{\mathrm{inc}}^{a}\left(x\right)\cdot J_{m}^{b}\left(x\right)\right]={\;\int \int \;}_{S}d^{2}x\;\left[E_{\mathrm{inc}}^{b}\left(x\right)\cdot J_{e}^{a}\left(x\right)-H_{\mathrm{inc}}^{b}\left(x\right)\cdot J_{m}^{a}\left(x\right)\right]. \end{array}$$

Expressing the currents via the ACGF yields:

(A52) $$\begin{array}{cc} J_{e}^{b}\left(x\right) & ={\;\int \int \;}_{S}d^{2}x^{\prime }\;\left[{\bar{F}}_{\mathrm{ee}}\left(x,x^{\prime }\right)\cdot E_{\mathrm{inc}}^{b}\left(x^{\prime }\right)+{\bar{F}}_{\mathrm{em}}\left(x,x^{\prime }\right)\cdot H_{\mathrm{inc}}^{b}\left(x^{\prime }\right)\right], \end{array}$$

(A53) $$\begin{array}{cc} J_{m}^{b}\left(x\right) & ={\;\int \int \;}_{S}d^{2}x^{\prime }\;\left[{\bar{F}}_{\mathrm{me}}\left(x,x^{\prime }\right)\cdot E_{\mathrm{inc}}^{b}\left(x^{\prime }\right)+{\bar{F}}_{\mathrm{mm}}\left(x,x^{\prime }\right)\cdot H_{\mathrm{inc}}^{b}\left(x^{\prime }\right)\right], \end{array}$$

with analogous expressions for $J_{e}^{a}\left(x\right)$ and $J_{m}^{a}\left(x\right)$. Substituting (A52) and (A53) into the left-hand side of (A51) gives:

(A54) $$\begin{array}{cc} R_{AB} & ={\;\int \int \;}_{S}d^{2}x{\;\int \int \;}_{S}d^{2}x^{\prime }\;E_{\mathrm{inc}}^{a}\left(x\right)\cdot {\bar{F}}_{\mathrm{ee}}\left(x,x^{\prime }\right)\cdot E_{\mathrm{inc}}^{b}\left(x^{\prime }\right)+{\;\int \int \;}_{S}d^{2}x{\;\int \int \;}_{S}d^{2}x^{\prime }\;E_{\mathrm{inc}}^{a}\left(x\right)\cdot {\bar{F}}_{\mathrm{em}}\left(x,x^{\prime }\right)\cdot H_{\mathrm{inc}}^{b}\left(x^{\prime }\right)\\ \; & \;-{\;\int \int \;}_{S}d^{2}x{\;\int \int \;}_{S}d^{2}x^{\prime }\;H_{\mathrm{inc}}^{a}\left(x\right)\cdot {\bar{F}}_{\mathrm{me}}\left(x,x^{\prime }\right)\cdot E_{\mathrm{inc}}^{b}\left(x^{\prime }\right)-{\;\int \int \;}_{S}d^{2}x{\;\int \int \;}_{S}d^{2}x^{\prime }\;H_{\mathrm{inc}}^{a}\left(x\right)\cdot {\bar{F}}_{\mathrm{mm}}\left(x,x^{\prime }\right)\cdot H_{\mathrm{inc}}^{b}\left(x^{\prime }\right). \end{array}$$

The right-hand side $R_{BA}$ has a similar expansion with indices *a* and *b* interchanged. Since (A51) must hold for all possible incident fields $E_{\mathrm{inc}}^{a,b}$ and $H_{\mathrm{inc}}^{a,b}$, the coefficients of each independent field product must match on both sides. The details are as follows:

1. *Electric–electric terms:* Equating coefficients of $E_{\mathrm{inc}}^{a}\left(x\right)\cdot E_{\mathrm{inc}}^{b}\left(x^{\prime }\right)$ yields ${\bar{F}}_{\mathrm{ee}}\left(x,x^{\prime }\right)={\bar{F}}_{\mathrm{ee}}^{⊤}\left(x^{\prime },x\right),$ where the transpose arises from the identity $a\cdot \bar{D}\cdot b=b\cdot {\bar{D}}^{⊤}\cdot a$ and the exchange of integration variables $x↔x^{\prime }$.
2. *Magnetic–magnetic terms:* For $H_{\mathrm{inc}}^{a}\left(x\right)\cdot H_{\mathrm{inc}}^{b}\left(x^{\prime }\right)$, we obtain ${\bar{F}}_{\mathrm{mm}}\left(x,x^{\prime }\right)={\bar{F}}_{\mathrm{mm}}^{⊤}\left(x^{\prime },x\right),$ with both sides carrying a negative sign that cancels.
3. *Electric–magnetic cross terms:* Matching coefficients of $E_{\mathrm{inc}}^{a}\left(x\right)\cdot H_{\mathrm{inc}}^{b}\left(x^{\prime }\right)$ gives ${\bar{F}}_{\mathrm{em}}\left(x,x^{\prime }\right)=-\;{\bar{F}}_{\mathrm{me}}^{⊤}\left(x^{\prime },x\right),$ where the minus sign originates from the opposite signs with which these terms appear in the reaction integrals.
4. *Magnetic–electric cross terms:* The relation for $H_{\mathrm{inc}}^{a}\left(x\right)\cdot E_{\mathrm{inc}}^{b}\left(x^{\prime }\right)$ yields ${\bar{F}}_{\mathrm{me}}\left(x,x^{\prime }\right)=-\;{\bar{F}}_{\mathrm{em}}^{⊤}\left(x^{\prime },x\right),$ which is equivalent to ${\bar{F}}_{\mathrm{em}}\left(x,x^{\prime }\right)=-\;{\bar{F}}_{\mathrm{me}}^{⊤}\left(x^{\prime },x\right)$ upon transposition and argument exchange.

Thus, the component relations (A45a)–(A45c) are established. Note that the proof does not assume losslessness; it requires only the symmetry of constitutive tensors, which defines reciprocity. To verify the compact form (A48), compute:

(A55) $$\bar{\bar{\Omega }}\cdot {\bar{\bar{F}}}^{⊤}\left(x^{\prime },x\right)\cdot {\bar{\bar{\Omega }}}^{⊤}=\left[\begin{array}{cc} \bar{I} & 0\\ 0 & -\bar{I} \end{array}\right]\cdot \left[\begin{array}{cc} {\bar{F}}_{\mathrm{ee}}^{⊤}\left(x^{\prime },x\right) & {\bar{F}}_{\mathrm{me}}^{⊤}\left(x^{\prime },x\right)\\ {\bar{F}}_{\mathrm{em}}^{⊤}\left(x^{\prime },x\right) & {\bar{F}}_{\mathrm{mm}}^{⊤}\left(x^{\prime },x\right) \end{array}\right]\cdot \left[\begin{array}{cc} \bar{I} & 0\\ 0 & -\bar{I} \end{array}\right]=\left[\begin{array}{cc} {\bar{F}}_{\mathrm{ee}}^{⊤}\left(x^{\prime },x\right) & -{\bar{F}}_{\mathrm{me}}^{⊤}\left(x^{\prime },x\right)\\ -{\bar{F}}_{\mathrm{em}}^{⊤}\left(x^{\prime },x\right) & {\bar{F}}_{\mathrm{mm}}^{⊤}\left(x^{\prime },x\right) \end{array}\right].$$

Direct comparison with $\bar{\bar{F}}\left(x,x^{\prime }\right)$ from (A46) confirms that (A55) equals $\bar{\bar{F}}\left(x,x^{\prime }\right)$ precisely when the component relations (A45a)–(A45c) hold. Since $\bar{\bar{\Omega }}$ is symmetric (${\bar{\bar{\Omega }}}^{⊤}=\bar{\bar{\Omega }}$), the compact form can equivalently be written as in (A48). This completes the proof.    □

The reciprocity relations (A45) reveal that the diagonal components ${\bar{F}}_{\mathrm{ee}}$ and ${\bar{F}}_{\mathrm{mm}}$ are individually symmetric under exchange of observation and source points combined with dyadic transposition. In contrast, the off-diagonal components exhibit anti-symmetry with respect to the same operation, as indicated by the minus sign in (A45c). This structure mirrors that of the classical electromagnetic dyadic Green functions, confirming that the ACGF inherits the fundamental symmetries of Maxwell’s equations in reciprocal media.

## Appendix G. Reciprocity-Induced Symmetry of Stochastic Superdyadics

**Proposition** **A3.**
*Consider a standard stochastic EM transmission system (Definition 4) in which all medium and antenna Green’s functions satisfy the reciprocity condition*

(A56)
$$\bar{\bar{X}}\left(x,x^{\prime }\right)=\bar{\bar{\Omega }}\cdot {\bar{\bar{X}}}^{⊤}\left(x^{\prime },x\right)\cdot \bar{\bar{\Omega }},$$

*where $\bar{\bar{X}}$ denotes any of ${\bar{\bar{G}}}_{t}$, ${\bar{\bar{G}}}_{s}$, ${\bar{\bar{F}}}_{t}$, ${\bar{\bar{F}}}_{s}$, or ${\bar{\bar{F}}}_{r}$, and $\bar{\bar{\Omega }}=\left[\begin{array}{cc} \bar{I} & 0\\ 0 & -\bar{I} \end{array}\right]$ is the signature superdyad. Then the stochastic system superdyadics satisfy the following distributional symmetries:*

(A57)
$$\begin{array}{cc} {\bar{\bar{S}}}_{\mathrm{los}}^{⊤}( & x,x_{1},x_{4},x_{5})\overset{d}{=}\bar{\bar{\Omega }}\cdot {\bar{\bar{S}}}_{\mathrm{los}}\left(x_{5},x_{4},x_{1},x\right)\cdot \bar{\bar{\Omega }}, \end{array}$$

(A58)
$$\begin{array}{cc} {\bar{\bar{S}}}_{\mathrm{nlos}}^{⊤}( & x,x_{1},x_{2},x_{3},x_{4},x_{5})\overset{d}{=}\bar{\bar{\Omega }}\cdot {\bar{\bar{S}}}_{\mathrm{nlos}}\left(x_{5},x_{4},x_{3},x_{2},x_{1},x\right)\cdot \bar{\bar{\Omega }}, \end{array}$$

*where the notation $X\overset{d}{=}Y$ means that the random superdyadics X and Y are equal in distribution, i.e., they induce the same probability law on the space of tensor-valued random fields.*

**Proof.** We prove (A57); the proof of (A58) is analogous. From (55), ${\bar{\bar{S}}}_{\mathrm{los}}\left(x,x_{1},x_{4},x_{5}\right)={\bar{\bar{F}}}_{r}\left(x,x_{1}\right)\cdot {\bar{\bar{G}}}_{t}\left(x_{1},x_{4}\right)\cdot {\bar{\bar{F}}}_{t}\left(x_{4},x_{5}\right).$ Taking the transpose and using the dyadic identity ${\left(\bar{A}\cdot \bar{B}\cdot \bar{C}\right)}^{⊤}={\bar{C}}^{⊤}\cdot {\bar{B}}^{⊤}\cdot {\bar{A}}^{⊤},$ we obtain ${\bar{\bar{S}}}_{\mathrm{los}}^{⊤}\left(x,x_{1},x_{4},x_{5}\right)={\bar{\bar{F}}}_{t}^{⊤}\left(x_{4},x_{5}\right)\cdot {\bar{\bar{G}}}_{t}^{⊤}\left(x_{1},x_{4}\right)\cdot {\bar{\bar{F}}}_{r}^{⊤}\left(x,x_{1}\right).$ By reciprocity (A56), each factor satisfies ${\bar{\bar{X}}}^{⊤}\left(u,v\right)=\bar{\bar{\Omega }}\cdot \bar{\bar{X}}\left(v,u\right)\cdot \bar{\bar{\Omega }}.$ Substituting and using the mutual independence of all Green’s functions (so that the joint distribution is preserved under this transformation), we obtain

(A59) $$\begin{array}{cc} {\bar{\bar{S}}}_{\mathrm{los}}^{⊤}\left(x,x_{1},x_{4},x_{5}\right) & \overset{d}{=}\bar{\bar{\Omega }}\cdot {\bar{\bar{F}}}_{t}\left(x_{5},x_{4}\right)\cdot \bar{\bar{\Omega }}\cdot \bar{\bar{\Omega }}\cdot {\bar{\bar{G}}}_{t}\left(x_{4},x_{1}\right)\cdot \bar{\bar{\Omega }}\cdot \bar{\bar{\Omega }}\cdot {\bar{\bar{F}}}_{r}\left(x_{1},x\right)\cdot \bar{\bar{\Omega }}\\ \; & \overset{d}{=}\bar{\bar{\Omega }}\cdot {\bar{\bar{F}}}_{t}\left(x_{5},x_{4}\right)\cdot {\bar{\bar{G}}}_{t}\left(x_{4},x_{1}\right)\cdot {\bar{\bar{F}}}_{r}\left(x_{1},x\right)\cdot \bar{\bar{\Omega }}\\ \; & \overset{d}{=}\bar{\bar{\Omega }}\cdot {\bar{\bar{S}}}_{\mathrm{los}}\left(x_{5},x_{4},x_{1},x\right)\cdot \bar{\bar{\Omega }}, \end{array}$$

which is (A57). The NLoS case follows by applying the same steps to the five-factor product in (56).    □

One might wonder why mutual independence of the Green’s functions is invoked here. The reciprocity condition is applied to each Green’s function separately, replacing, for example, ${\bar{\bar{F}}}_{t}^{⊤}\left(x_{4},x_{5},\varpi \right)$ with $\bar{\bar{\Omega }}\cdot {\bar{\bar{F}}}_{t}\left(x_{5},x_{4},\varpi \right)\cdot \bar{\bar{\Omega }}$—a pointwise (sample-path) transformation of that single random dyadic. Mutual independence ensures that such transformations do not induce artificial statistical couplings among the factors in the product defining ${\bar{\bar{S}}}_{\mathrm{los}}^{⊤}$. Specifically, because the joint distribution of $\left({\bar{\bar{F}}}_{t},{\bar{\bar{G}}}_{t},{\bar{\bar{F}}}_{r}\right)$ factorizes into the product of its marginals, the joint law of the transformed triple $\left(\bar{\bar{\Omega }}\cdot {\bar{\bar{F}}}_{t}\cdot \bar{\bar{\Omega }},\;\bar{\bar{\Omega }}\cdot {\bar{\bar{G}}}_{t}\cdot \bar{\bar{\Omega }},\;\bar{\bar{\Omega }}\cdot {\bar{\bar{F}}}_{r}\cdot \bar{\bar{\Omega }}\right)$ also factorizes into the product of the transformed marginals. Consequently, the statistical structure of the full tensor product is preserved under the transformation, and the resulting expression has the same distribution as $\bar{\bar{\Omega }}\cdot {\bar{\bar{S}}}_{\mathrm{los}}\left(x_{5},x_{4},x_{1},x\right)\cdot \bar{\bar{\Omega }}$. Without mutual independence, the transformation of one Green function could alter the conditional distribution of another, breaking the equality in distribution.

## Appendix H. Complex-Valued Random Fields and Their Real and Complex Vector Representations

We begin by introducing one of the primary mathematical data structures used throughout this paper: the real and complex vector equivalent forms. Following this, we present the concept of proper and improper RFs, which is projected to be fundamental to both the theoretical analysis and the simulation of EM RFs developed herein. For additional background on complex RVs and the notion of properness, see [85,119,141,142,143,144,145].

**Definition** **A6** (Real and complex vector field representation)**.** 
*Let $g:\Omega \to L^{2}\left(R^{d},C^{n\times m}\right)$ be a complex-valued RF, where d,n,m∈N, and $C^{n\times 1}$ is identified with $C^{n}$. Specifically, g∈V may represent a vector, supervector, dyad, superdyad, or a general complex array (see Table 4 and Appendix A, Appendix C, and Appendix D). The vectorized form $g^{\left(v\right)}$ is defined as follows. If m=1 or n=1, then $g^{\left(v\right)}$ corresponds to the $C^{n\times 1}$ list of g or the $C^{1\times m}$ list of $g^{⊤}$, respectively. In other cases, $g^{\left(v\right)}$ is the vectorization of g, represented as an nm×1 array constructed by sequentially tracing the elements of $g\left(x,\varpi \right)\in C^{n\times m}$ either row by row or column by column. The real vector representation of g, denoted by $g^{\left(r\right)}$, is defined as its vectorized form with real and imaginary components arranged sequentially. Specifically, we have*

g(r):=Reg(v)Img(v)⊤.

*The complex vector representation of g, denoted by $g^{\left(c\right)}$, is given by $g^{\left(c\right)}:=g^{\left(v\right)}.$*

For example, if E(x,ω,ϖ) is the complex-valued vector electric RF, then its complex vector representation is $E^{\left(c\right)}={\left[\begin{array}{ccc} E^{1} & E^{2} & E^{3} \end{array}\right]}^{⊤}$, while the real vector representation is given by the augmented column array $E^{\left(r\right)}$ defined as

ReE1ReE2ReE3ImE1ImE2ImE3⊤.

We are especially interested in all those complex-valued RFs $\bar{f}$ that emerge as the complex vector representation of some of the EM RFs appearing in our theory, i.e., in all $\bar{f}$ such that there exist an RF g∈V (see Table 4) and $\bar{f}=g^{\left(c\right)}$. A comprehensive list of all vectorized forms of the EM quantities considered in this paper is provided in Appendix J. To transform a given g∈V into its real and complex vector representations, we employ the procedure outlined in Algorithm A1.

**Definition** **A7** (Covariance and pseudo-covariance matrices of a complex-valued vector random field)**.** 
*Let $\bar{v}\in \left\{\bar{f}|\bar{f}:\Omega \to L^{2}\left(R^{d},C^{n}\right)\right\}$, where n∈N. The complex covariance matrix of the complex-valued vector RF $\bar{v}$ is defined as*

(A60)
$$C_{\bar{v}\bar{v}}:=E\left[\left(\bar{v}-E\left\{\bar{v}\right\}\right){\left(\bar{v}-E\left\{\bar{v}\right\}\right)}^{†}\right].$$

*On the other hand, the pseudo-covariance matrix, denoted by $C_{\bar{v}\bar{v}}^{\prime }$, is defined by replacing Hermitian transposition with transposition in the definition above, i.e., we have*

(A61)
$$C_{\bar{v}\bar{v}}^{\prime }:=E\left[\left(\bar{v}-E\left\{\bar{v}\right\}\right){\left(\bar{v}-E\left\{\bar{v}\right\}\right)}^{⊤}\right].$$

*The correlation and pseudo-correlation matrices are defined by*

(A62)
$$R_{\bar{v}\bar{v}}:=E\left[\bar{v}{\bar{v}}^{†}\right],\;\;R_{\bar{v}\bar{v}}^{\prime }:=E\left[\bar{v}{\bar{v}}^{⊤}\right],$$

*respectively.*

The terminology of proper complex RV and pseudo-covariance appears to have been first introduced in [141]. The pseudo-covariance matrix is also referred to as the relation matrix [142] or the complementary covariance matrix [85]. It can be shown that the covariance matrix is hermitian and positive semidefinite, while the pseudo-covariance is a symmetric matrix.

The following proposition presents a natural extension of Theorem A2 to complex-valued vector RFs and is utilized throughout this paper.

**Algorithm A1** Construction of Real and Complex Vector Representations of Generic EM-RF *g*
1. Let $S_{g}:=\left\{g_{i}\;\mathrm{is}\;a\;\mathrm{complex}\;\mathrm{element}\;\mathrm{of}\;g,i\in N\right\}$ denote the set of all complex entries in the RF g∈V, where $|S_{g}|=N_{g}$. Establish an injective mapping $i\mapsto g_{i}=\varphi \left(i\right)$ as follows: (A63) $$\varphi :\left\{1,2,\ldots ,N_{g}\right\}\to S_{g},$$ where (A64) $$\varphi \left(i\right):=g_{\sigma (i)},$$ and σ is a permutation of the set $\left\{1,2,\ldots ,N_{g}\right\}$. This defines a one-to-one correspondence between the *complex* elements of *g* and the set $\left\{1,2,\ldots ,N_{g}\right\}$. Specifically, $N_{g}$ is the product of the dimensions of the codomain provided in Table 3, which matches the dimension of the codomain in Table A4. 2. To construct $g^{\left(c\right)}$, each complex element $g_{i}\in S_{g}$ will be mapped into a position (dimension) in the space $C^{N_{g}}$. Let $\pi_{i}:C^{N}\to C,i\leq N$, be the standard projection map of a vector in $C^{N}$. The most natural construction of $g^{\left(c\right)}$ is the following: (A65) $$\pi_{i}\left[g^{\left(c\right)}\right]:=g_{\varphi (i)},$$ for $i=1,2,\ldots ,N_{g}$. 3. To construct $g^{\left(r\right)}$, we need two projection maps defined as follow (A66) πig(r):=Regφ(i),πNg+ig(r):=Imgφ(i), for $i=1,2,\ldots ,N_{g}$.

**Proposition** **A4.**
*Under the same conditions specified in Theorem A2, let g be a complex-valued RF. The mean is computable through an equation identical to *(A26)*, while *(A27)* is modified to*

(A67)
$$C_{g}\left(\bar{x},{\bar{x}}^{\prime }\right)=\int_{D}\int_{D}K\left(\bar{x},\bar{y}\right)K^{*}\left({\bar{x}}^{\prime },{\bar{y}}^{\prime }\right)C_{f}\left(\bar{y},{\bar{y}}^{\prime }\right)\;d^{n}y\;d^{n}y^{\prime },$$

*Additionally, the pseudo-covariance of $g(\bar{x},\varpi )$ is given by:*

(A68)
$$C_{g}^{\prime }\left(\bar{x},{\bar{x}}^{\prime }\right)=\int_{D}\int_{D}K\left(\bar{x},\bar{y}\right)K\left({\bar{x}}^{\prime },{\bar{y}}^{\prime }\right)C_{f}^{\prime }\left(\bar{y},{\bar{y}}^{\prime }\right)\;d^{n}y\;d^{n}y^{\prime }.$$

*Here, $C_{f}\left(\bar{y},{\bar{y}}^{\prime }\right)$ and $C_{f}^{\prime }\left(\bar{y},{\bar{y}}^{\prime }\right)$ are the covariance and the pseudo-covariance of the original RF $f(\bar{x},\varpi )$.*

**Proof.** Using the injective mappings provided in Algorithm A1, we transition between real and complex vector representations, employing Definition A7 to establish the complex covariance and pseudo-covariance. Applying Theorem A2 subsequently yields the necessary expressions (A26) for the mean and (A67), and (A68) for the covariance and pseudo-covariance in the complex vector RF case. □

The following definition is essential for extending key information-theoretic measures and other quantities reliant on multivariate probability distributions to complex-valued EM RFs.

**Definition** **A8** (Probability density function of a complex-valued RF)**.** 
*Consider a second-order complex-valued RF g as given in Definition A6. The probability density function (pdf) of $\bar{v}$ is defined as the multivariate pdf of the vector $g^{\left(r\right)}$.*

Therefore, all EM currents, fields, and GFs in Appendix C and Appendix D can be assigned a probability density function (pdf) in an unambiguous manner as the joint pdf of the real vector representation of the quantity under consideration. Other probabilistic measures, such as entropy, mutual information, and conditional probability, which depend on the pdf of a complex-valued RF, can be computed in terms of this pdf and are similarly defined in relation to $g^{\left(r\right)}$.

**Definition** **A9** (Circularity and properness of a complex-valued random field)**.** 
*Consider a second-order complex-valued vector RF g as defined in Definition A6, and let $D\subseteq R^{d}$. We say that g is circular on D if, for all $\bar{x}\in D$, the random variable $g(\bar{x})$ has the same pdf as $e^{i2\pi \theta }g\left(\bar{x}\right)$, where θ∈[0,1). Otherwise, we say that g is non-circular. If the pseudo-covariance matrix of g exists and is identically zero for all $\bar{x}\in D$, we say that g is proper on D. Otherwise, we say that g is improper.*

For second-order RFs, circularity implies properness. An important special case is the Gaussian RF, where properness combined with a zero mean implies circularity.

In the following proposition, which will be extensively utilized throughout this paper, we establish a relationship between the complex correlation and pseudo-covariance matrices defined in Definition A7 for the complex vector representation $g^{\left(c\right)}$, and the original covariance matrix for the corresponding real vector representation $g^{\left(r\right)}$, as initially introduced in Appendix B and expressed in Equation (A8).

**Proposition** **A5.**
*If we expand $\bar{v}$ in terms of its real and imaginary parts, i.e., $\bar{v}={\bar{v}}_{r}+i{\bar{v}}_{i}$, where v¯r:=Rev¯ and v¯i:=Imv¯, then we can re-express the n×1 complex random vector $\bar{v}$ as the 2n×1 real random vector ${\bar{v}}^{\left(r\right)}={\left(\begin{array}{cc} {\bar{v}}_{r} & {\bar{v}}_{i} \end{array}\right)}^{⊤}.$ The covariance matrix of the real-valued vector RF ${\bar{v}}^{\left(r\right)}$ is given by*

(A69)
Cv¯rv¯rCv¯rv¯iCv¯iv¯rCv¯iv¯i=12ReCu¯u¯+Cu¯u¯′ImCu¯u¯′−Cu¯u¯ImCu¯u¯+Cu¯u¯′ReCu¯u¯−Cu¯u¯′,

*where $\bar{u}:={\bar{v}}^{\left(c\right)}$.*

Table A2 summarizes the necessary expressions and their definitions. The system of Equation (A69) provides an algorithm to compute the correlation between the real and imaginary parts of complex random vectors based on the latter’s correlation and pseudo-correlation matrices and will be used in this paper to link the complex representation of vector RFs with their real representations.

**Table A2:** The covariance matrices associated with complex random vectors expressed in terms of the complex correlation and pseudo-correlation matrices. Here $\bar{u}={\bar{v}}^{\left(c\right)},\bar{v}={\bar{v}}_{r}+i{\bar{v}}_{i}$.

Correlation	Definition	Expression
$C_{{\bar{v}}_{r}{\bar{v}}_{r}}$	$E\left[\left({\bar{v}}_{r}-E\left\{{\bar{v}}_{r}\right\}\right){\left({\bar{v}}_{r}-E\left\{{\bar{v}}_{r}\right\}\right)}^{⊤}\right]$	12Re[Cu¯u¯+Cu¯u¯′]
$C_{{\bar{v}}_{i}{\bar{v}}_{i}}$	$E\left[\left({\bar{v}}_{i}-E\left\{{\bar{v}}_{i}\right\}\right){\left({\bar{v}}_{i}-E\left\{{\bar{v}}_{i}\right\}\right)}^{⊤}\right]$	12Re[Cu¯u¯−Cu¯u¯′]
$C_{{\bar{v}}_{r}{\bar{v}}_{i}}$	$E\left[\left({\bar{v}}_{r}-E\left\{{\bar{v}}_{r}\right\}\right){\left({\bar{v}}_{i}-E\left\{{\bar{v}}_{i}\right\}\right)}^{⊤}\right]$	12Im[Cu¯u¯′−Cu¯u¯]
$C_{{\bar{v}}_{i}{\bar{v}}_{r}}$	$E\left[\left({\bar{v}}_{i}-E\left\{{\bar{v}}_{i}\right\}\right){\left({\bar{v}}_{r}-E\left\{{\bar{v}}_{r}\right\}\right)}^{⊤}\right]$	12Im[Cu¯u¯′+Cu¯u¯]

Note that for a complex-valued RF $g(\bar{x})$, geometric concepts such as m.s. continuity and differentiability can be readily defined by first constructing the real vector representation $g^{\left(r\right)}\left(\bar{x}\right)$, and then applying the corresponding standard definitions from the real vector RF case to it. The following lemma, which will be used extensively, provides a useful and efficient method for establishing the m.s. continuity of a complex-valued RF.

**Lemma** **A1.**
*Let g be a complex-valued RF with covariance and pseudo-covariance matrices $C_{g}\left(\bar{x},{\bar{x}}^{\prime }\right)$ and $C_{g}^{\prime }\left(\bar{x},{\bar{x}}^{\prime }\right)$, respectively, as defined in Definition A7. Let the following two conditions are satisfied:*

1. *The mean $E\{g\left(\bar{x}\right)\}$ is continuous for every ${\bar{x}}_{0}$ in the domain of $g({\bar{x}}_{0})$.*
2. *The covariance and pseudo-covariance $C_{g}$ and $C_{g}^{\prime }$ are diagonally continuous, i.e., continuous as $\bar{x},{\bar{x}}^{\prime }\to {\bar{x}}_{0}$ for every ${\bar{x}}_{0}$ in the domain of g.*

*Then the complex-valued RF $g(\bar{x})$ is m.s. continuous.*

**Proof.** This follows directly from the fact that the sum of two continuous functions is continuous. Applying this to (A69) and invoking Theorem A5 immediately proves the lemma. □

## Appendix I. Covariance/Pseudo-Covariance and Correlation/Pseudo-Covariance of Complex-Valued Random Fields

**Definition** **A10** (Notation for covariance/pseudo-covariance and correlation/pseudo- correlation functions)**.** 
*Consider a generic EM RF g∈V, where V is defined in Table 2. The covariance and pseudo-covariance, denoted as $C_{g}$ and $C_{g}^{\prime }$, respectively, always signify these quantities relative to the complex vector representation $g^{\left(c\right)}$, as outlined in Definition A7. Thus, throughout this paper, we have $C_{g}:=C_{g^{\left(c\right)}}$ and $C_{g}^{\prime }:=C_{g^{\left(c\right)}}^{\prime }$. The same convention is applied to the correlation and pseudo-correlation matrices. Specifically, we have $R_{g}:=R_{g^{\left(c\right)}}$ and $R_{g}^{\prime }:=R_{g^{\left(c\right)}}^{\prime }$.*

**Remark** **A2.**
*Definition A10 introduces a significant simplification in the notation used throughout this paper. While the theory of EM RFs is ultimately developed with respect to real RFs (Appendix B), calculations in EM-RFT are typically performed using complex-valued vector representations, including vectors, supervectors, dyads, and superdyads. To streamline the notation, we adopt the convention outlined in Definition A10, whereby all covariance and pseudo-covariance functions for EM RFs are expressed relative to complex representations (Appendix H). This includes superdyadic quantities such as $\bar{\bar{G}}$ and ${\bar{\bar{F}}}_{b}$. If it becomes necessary to explicitly refer to covariance data for real RFs, the notation $C_{g^{\left(r\right)}}$ is used. (By definition, real RFs do not possess pseudo-covariance.)*

**Remark** **A3** (Relation between correlation/pseudo-correlation and covariance/pseudo- covariance matrices of a generic vector complex-valued random field)**.** 
*The covariance matrix of a complex-valued vector RF $\bar{v}\left(x\right)$, denoted by $C_{\bar{v}}\left(x,x^{\prime }\right)$, can be directly expressed in terms of the correlation matrix $R_{\bar{v}}\left(x,x^{\prime }\right)$ if the mean values of the fields are known, using the relation:*

(A70)
$$C_{\bar{v}}\left(x,x^{\prime }\right)=R_{\bar{v}}\left(x,x^{\prime }\right)-E\left\{(\bar{v}\left(x\right)\right\}E{\left\{\bar{v}\left(x^{\prime }\right)\right\}}^{†},$$

*and, similarly, for the case of pseudo-covariance and pseudo-correlation matrices we have*

(A71)
$$C_{\bar{v}}^{\prime }\left(x,x^{\prime }\right)=R_{\bar{v}}^{\prime }\left(x,x^{\prime }\right)-E\left\{(\bar{v}\left(x\right)\right\}E{\left\{\bar{v}\left(x^{\prime }\right)\right\}}^{⊤}.$$

*For the relationship between the covariance and pseudo-covariance of a complex-valued vector RF and the covariance matrices of real-valued vector RFs, refer to Appendix H and Table A2.*

**Remark** **A4.**
*Using the complex vector representation in Appendix H, the correlation matrix of the current RF ${\bar{J}}_{b}\left(x,\omega ,\varpi \right)$, as given by Equations *(61)* and *(62)*, can be expressed compactly as*

(A72)
$$R_{{\bar{J}}_{b}}\left(x,x^{\prime },\omega \right)=E\left\{{\bar{J}}_{b}^{\left(c\right)}\left(x,\omega ,\varpi \right){\bar{J}}_{b}^{(c),†}\left(x^{\prime },\omega ,\varpi \right)\right\},$$

*while the corresponding pseudo-correlation matrix can be computed through the slightly different expression*

(A73)
$$R_{{\bar{J}}_{b}}^{\prime }\left(x,x^{\prime },\omega \right)=E\left\{{\bar{J}}_{b}^{\left(c\right)}\left(x,\omega ,\varpi \right){\bar{J}}_{b}^{(c),⊤}\left(x^{\prime },\omega ,\varpi \right)\right\},$$

*for b∈{t,r,s}. Similarly, for the RF $\psi_{d}\left(x,\omega ,\varpi \right),d\in \left\{\mathrm{in},\mathrm{rad},s\right\},$ the correlation and pseudo-correlation matrices can be computed through the relations^23^*

(A74)
$$\begin{array}{c} R_{{\bar{\psi }}_{d}}\left(x,x^{\prime },\omega \right):=E\left\{{\bar{\psi }}_{d}^{\left(c\right)}\left(x,\omega ,\varpi \right){\bar{\psi }}_{d}^{(c),†}\left(x^{\prime },\omega ,\varpi \right)\right\},\\ R_{{\bar{\psi }}_{d}}^{\prime }\left(x,x^{\prime },\omega \right):=E\left\{{\bar{\psi }}_{d}^{\left(c\right)}\left(x,\omega ,\varpi \right){\bar{\psi }}_{d}^{(c),⊤}\left(x^{\prime },\omega ,\varpi \right)\right\}. \end{array}$$

*For the surface current GF RFs, the correlation and pseudo-correlation matrices are given by the expressions:*

(A75)
$$\begin{array}{cc} R_{{\bar{\bar{F}}}_{b}}\left(x_{1},x_{1}^{\prime },x_{2},x_{2}^{\prime },\omega \right) & :=E\left\{{\bar{\bar{F}}}_{b}^{\left(c\right)}\left(x_{1},x_{1}^{\prime },\omega ,\varpi \right){\bar{\bar{F}}}_{b}^{(c),†}\left(x_{2},x_{2}^{\prime },\omega ,\varpi \right)\right\},\\ R_{{\bar{\bar{F}}}_{b}}^{\prime }\left(x_{1},x_{1}^{\prime },x_{2},x_{2}^{\prime },\omega \right) & :=E\left\{{\bar{\bar{F}}}_{b}^{\left(c\right)}\left(x_{1},x_{1}^{\prime },\omega ,\varpi \right){\bar{\bar{F}}}_{b}^{(c),⊤}\left(x_{2},x_{2}^{\prime },\omega ,\varpi \right)\right\}. \end{array}$$

*Finally, for the medium GF RFs, we also have the analogous formulas:*

(A76)
$$\begin{array}{cc} R_{{\bar{\bar{G}}}_{b^{\prime }}}\left(x_{1},x_{1}^{\prime },x_{2},x_{2}^{\prime },\omega \right) & :=E\left\{{\bar{\bar{G}}}_{b^{\prime }}^{\left(c\right)}\left(x_{1},x_{1}^{\prime },\omega ,\varpi \right){\bar{\bar{G}}}_{b^{\prime }}^{(c),†}\left(x_{2},x_{2}^{\prime },\omega ,\varpi \right)\right\},\\ R_{{\bar{\bar{G}}}_{b^{\prime }}}^{\prime }\left(x_{1},x_{1}^{\prime },x_{2},x_{2}^{\prime },\omega \right) & :=E\left\{{\bar{\bar{G}}}_{b^{\prime }}^{\left(c\right)}\left(x_{1},x_{1}^{\prime },\omega ,\varpi \right){\bar{\bar{G}}}_{b^{\prime }}^{(c),⊤}\left(x_{2},x_{2}^{\prime },\omega ,\varpi \right)\right\}, \end{array}$$

*for $b^{\prime }\in \left\{t,s\right\}$.*

**Definition** **A11** (The superdyadic correlation/pseudo-correlation matrix of a supervector)**.** 
*Consider a generic supervector RF g∈Q∪I (see Table 4). The superdyadic correlation and pseudo-correlation matrices are defined as the following superdyads:*

(A77)
$$\begin{array}{c} {\bar{\bar{R}}}_{g}\left(x,x^{\prime },\omega \right):=E\left\{g\left(x,x^{\prime },\omega ,\varpi \right)g^{†}\left(x,x^{\prime },\omega ,\varpi \right)\right\},\\ {\bar{\bar{R}}}_{g}^{\prime }\left(x,x^{\prime },\omega \right):=E\left\{g\left(x,x^{\prime },\omega ,\varpi \right)g^{⊤}\left(x,x^{\prime },\omega ,\varpi \right)\right\}, \end{array}$$

*respectively.*

Similar definitions apply to the reduced covariance and pseudo-covariance of a supervector. For the important case of ${\bar{\psi }}_{\mathrm{in}}$, the superdyadic correlation and pseudo-correlation matrices are given by

(A78) $$\begin{array}{cc} \; & {\bar{\bar{R}}}_{{\bar{\psi }}_{\mathrm{in}}}\left(x,x^{\prime },\omega \right):=E\left\{{\bar{\psi }}_{\mathrm{in}}\left(x,\omega ,\varpi \right)\;{\bar{\psi }}_{\mathrm{in}}^{†}\left(x^{\prime },\omega ,\varpi \right)\right\},\\ \; & {\bar{\bar{R}}}_{{\bar{\psi }}_{\mathrm{in}}}^{\prime }\left(x,x^{\prime },\omega \right):=E\left\{{\bar{\psi }}_{\mathrm{in}}\left(x,\omega ,\varpi \right)\;{\bar{\psi }}_{\mathrm{in}}^{⊤}\left(x^{\prime },\omega ,\varpi \right)\right\}, \end{array}$$

respectively. The superdyads ${\bar{\bar{R}}}_{{\bar{\psi }}_{\mathrm{in}}}$ and ${\bar{\bar{R}}}_{{\bar{\psi }}_{\mathrm{in}}}^{\prime }$ are 2×2 dyadic-valued arrays, whereas the corresponding matrices $R_{{\bar{\psi }}_{\mathrm{in}}}$ and $R_{{\bar{\psi }}_{\mathrm{in}}}^{\prime }$ are 4×4 complex-valued arrays. Generally, knowledge of $R_{g}$ is equivalent to knowledge of ${\bar{\bar{R}}}_{g}$ (and similarly for pseudo-correlation). In this paper, reduced matrices will only be used in conjunction with the main theorem of Section 3.7.

## Appendix J. Complete List of the Real Vector Representations of All Electromagnetic RFs in EM Information Transmission Systems

Our objective here is to outline a procedure in which all the EM supervector and superdyadic fields, as collected in Table 4, can be promoted into RFs and subsequently expressed in a unified form. This form corresponds to the real vector RF model described in Appendix B, utilizing the real vector representation introduced in Definition A6. We provide a comprehensive list of all real vector representations necessary for the EM-RFT presented in this paper. For brevity, we omit the complex vector representations, as they are self-evident.

In this paper, the generic complex-valued field g∈V can have different types (see Appendix A). The complete list is given by set V, defined in Table 4. In Table A3, we have compiled the main information regarding the structure of the data pertinent to the real vector RF forms $g^{\left(r\right)}$ of all currents, fields, and GFs relevant to stochastic EM information transmission systems. This includes specification of the domain and codomain of each quantity. Table A4 contains the corresponding data structures for the complex vector representations $g^{\left(c\right)}$.

**Table A3:** The domain and range of all real vector RF models $g^{\left(r\right)}$ of the EM supervector or superdyadic arrays *g* used in this paper. Here, b∈{t,s,r}, $b^{\prime }\in \left\{t,s\right\}$, and d∈{rad,s}.

Field	Domain	Codomain
$J_{b}^{\left(r\right)}\left(x,\omega \right)$	$S_{b}\times R^{+}$	$R^{8}$
$\psi_{\mathrm{in}}^{\left(r\right)}\left(x,\omega \right)$	$S_{t}\times R^{+}$	$R^{8}$
$\psi_{d}^{\left(r\right)}\left(x,\omega \right)$	$\left(R^{3}\setminus {⋃}_{b}V_{b}\right)\times R^{+}$	$R^{12}$
$F_{b}^{\left(r\right)}\left(x,x^{\prime },\omega \right)$	$S_{b}\times S_{b}\times R^{+}$	$R^{32}$
$G_{b^{\prime }}^{\left(r\right)}\left(x,x^{\prime },\omega \right)$	$\left(R^{3}\setminus {⋃}_{b}V_{b}\right)\times S_{b^{\prime }}\times R^{+}$	$R^{72}$

**Table A4:** The domain and range of all complex vector representations $g^{\left(c\right)}$ of the EM RF supervector or superdyadic arrays *g* used in this paper obtained from the corresponding quantities in Table A3. Here, b∈{t,s,r} and d∈{rad,s}.

Field	Domain	Codomain
${\bar{J}}_{b}^{\left(c\right)}\left(x,\omega \right)$	$S_{b}\times R^{+}$	$C^{4}$
${\bar{\psi }}_{\mathrm{in}}^{\left(c\right)}\left(x,\omega \right)$	$S_{t}\times R^{+}$	$C^{4}$
${\bar{\psi }}_{d}^{\left(c\right)}\left(x,\omega \right)$	$\left(R^{3}\setminus {⋃}_{b}V_{b}\right)\times R^{+}$	$C^{6}$
${\bar{\bar{F}}}_{b}^{\left(c\right)}\left(x,x^{\prime },\omega \right)$	$S_{b}\times S_{b}\times R^{+}$	$C^{18}$
${\bar{\bar{G}}}_{b^{\prime }}^{\left(c\right)}\left(x,x^{\prime },\omega \right)$	$\left(R^{3}\setminus {⋃}_{b}V_{b}\right)\times S_{b^{\prime }}\times R^{+}$	$C^{36}$

We start with the input complex RF ${\bar{\psi }}_{\mathrm{in}}\left(x,\omega \right)$. The equivalent real vector RF can be expressed as

(A79) ψ¯in(r)(x,ω,ϖ):=Ree1(x,ω,ϖ)Ree2(x,ω,ϖ)Reh1(x,ω,ϖ)Reh2(x,ω,ϖ)Ime1(x,ω,ϖ)Ime2(x,ω,ϖ)Imh1(x,ω,ϖ)Imh2(x,ω,ϖ)∈R8.

The corresponding covariance function of the vector RF $\psi_{\mathrm{in}}^{\left(r\right)}$ is the map

(A80) $$C_{{\bar{\psi }}_{\mathrm{in}}^{\left(r\right)}}:S_{t}\times S_{t}\times R^{+}\to R^{8\times 8}.$$

The induced current vectors ${\bar{J}}_{b}$ (see Appendix C) can be expressed as

(A81) J¯b(r)(x,ω,ϖ):=ReJeb,1(x,ω,ϖ)ReJeb,2(x,ω,ϖ)ReJmb,1(x,ω,ϖ)ReJmb,2(x,ω,ϖ)ImJeb,1(x,ω,ϖ)ImJeb,2(x,ω,ϖ)ImJmb,1(x,ω,ϖ)ImJmb,2(x,ω,ϖ)∈R8,

for b∈{t,s,r}. Similarly, the corresponding covariance function of the vector RF ${\bar{J}}_{b}^{\left(r\right)}$ is the map

(A82) $$C_{{\bar{J}}_{b}^{\left(r\right)}}:S_{b}\times S_{b}\times R^{+}\to R^{8\times 8}.$$

The radiation and scattered fields are written as

(A83) ψ¯d(r)(x,ω,ϖ):=ReEd1(x,ω,ϖ)ReEd2(x,ω,ϖ)ReEd3(x,ω,ϖ)ReHd1(x,ω,ϖ)ReHd2(x,ω,ϖ)ReHd3(x,ω,ϖ)ImEd1(x,ω,ϖ)ImEd2(x,ω,ϖ)ImEd3(x,ω,ϖ)ImHd1(x,ω,ϖ)ImHd2(x,ω,ϖ)ImHd3(x,ω,ϖ)∈R12,

for d∈{rad,s}. Here, the corresponding covariance function of the vector RF ${\bar{\psi }}_{d}^{\left(r\right)}$ is the map

(A84) $$C_{{\bar{\psi }}_{d}^{\left(r\right)}}:\left(R^{3}\setminus \;\underset{b}{⋃}V_{b}\right)\times \left(R^{3}\setminus \;\underset{b}{⋃}V_{b}\right)\times R^{+}\to R^{12\times 12}.$$

For the superdyadic response functions, we start with the current GF ${\bar{\bar{F}}}_{b}\left(x,x^{\prime },\omega \right)$. The equivalent real vector form, denoted by ${\bar{\bar{F}}}_{b}^{\left(r\right)}\left(x,x^{\prime },\omega \right)$, can be expressed as

(A85) F¯¯b(r)(x,x′,ω,ϖ):=ReFeeb,11(x,x′,ω,ϖ)⋮⋮⋮⋮⋮⋮ReFmmb,22(x,x′,ω,ϖ)ImFeeb,11(x,x′,ω,ϖ)⋮⋮⋮⋮⋮⋮ImFmmb,22(x,x′,ω,ϖ)∈R32.

The corresponding covariance function is a map with structure

(A86) $$C_{{\bar{\bar{F}}}_{b}^{\left(r\right)}}:S_{b}\times S_{b}\times S_{b}\times S_{b}\times R^{+}\to R^{32\times 32}$$

For the medium GF superdyad $\bar{\bar{G}}\left(x,x^{\prime },\omega ,\varpi \right)$, the real vector form, denoted by $G_{b^{\prime }}^{\left(r\right)},b^{\prime }\in \left\{t,s\right\}$, can be written as

(A87) G¯¯b′(r)(x,x′,ω,ϖ):=ReGee11,b′(x,x′,ω,ϖ)⋮⋮⋮⋮⋮⋮ReGmm33,b′(x,x′,ω,ϖ)ImGee11,b′(x,x′,ω,ϖ)⋮⋮⋮⋮⋮⋮ImGmm33,b′(x,x′,ω,ϖ)∈R72.

The corresponding covariance function is a map with structure

(A88) $$C_{{\bar{\bar{G}}}_{b^{\prime }}^{\left(r\right)}}:V_{\mathrm{ex}}\times S_{b^{\prime }}\times V_{\mathrm{ex}}\times S_{b^{\prime }}\times R^{+}\to R^{72\times 72}.$$

The basic structural information related to the covariance functions of the real vector RFs appearing in Table A3 is summarized in Table A5. We note that the size of the relevant real vector fields and their covariance matrices is quite large, especially for modeling the GFs. Since spatial regions will need to be discretized into grids, the overall size of the covariance matrix in actual computational examples is expected to be extremely large, particularly for the medium GF $\tilde{G}$ as it involves a three-dimensional region.

**Table A5:** The domain and range of all covariance functions of RFs in Table A3. Here, b∈{t,s,r}, $b^{\prime }\in \left\{t,s\right\}$, and d∈{rad,s}. Here $V_{\mathrm{ex}}$ is defined by (4).

Covariance Function	Domain	Codomain
$C_{J_{b}^{\left(r\right)}}\left(x,x^{\prime },\omega \right)$	$S_{b}\times S_{b}\times R^{+}$	$R^{8\times 8}$
$C_{\psi_{\mathrm{in}}^{\left(r\right)}}\left(x,x^{\prime },\omega \right)$	$S_{t}\times S_{t}\times R^{+}$	$R^{8\times 8}$
$C_{\psi_{d}^{\left(r\right)}}\left(x,x^{\prime },\omega \right)$	$V_{\mathrm{ex}}\times V_{\mathrm{ex}}\times R^{+}$	$R^{12\times 12}$
$C_{F_{b}^{\left(r\right)}}\left(x_{1},x_{1}^{\prime },x_{2},x_{2}^{\prime },\omega \right)$	$S_{b}\times S_{b}\times S_{b}\times S_{b}\times R^{+}$	$R^{32\times 32}$
$C_{G_{b^{\prime }}^{\left(r\right)}}\left(x_{1},x_{1}^{\prime },x_{2},x_{2}^{\prime },\omega \right)$	$V_{\mathrm{ex}}\times S_{b^{\prime }}\times V_{\mathrm{ex}}\times S_{b^{\prime }}\times R^{+}$	$R^{72\times 72}$

## Appendix K. Proof of Theorem 2

The proof of this theorem is straightforward, though somewhat technical and lengthy. For clarity and ease of comprehension, we will divide the proof into five distinct stages.

### Appendix K.1. Preliminary Considerations Regarding the Structures Involved

We first note that, formally speaking, all fundamental response function Formulas (12), (15), (18), (20), and (24) can be unified into the form:

(A89) $$\bar{R}\left(x,\omega ,\varpi \right)=\int_{x^{\prime }\in D}d^{2}x^{\prime }\;\bar{\bar{K}}\left(x,x^{\prime },\omega \right)\cdot \bar{E}\left(x^{\prime },\omega ,\varpi \right),$$

where $\bar{E}\left(x^{\prime },\omega ,\varpi \right)$ is a generic input RF and $\bar{\bar{K}}\left(x,x^{\prime },\omega \right)$ is some kernel function (given by one of the deterministic EM GFs), while $\bar{R}\left(x,\omega ,\varpi \right)$ is the corresponding response field and $x\in V\subset R^{3}$. The following is the detailed enumeration of all possible data structures occurring in (A89):

(A90) $$\begin{array}{cc} \; & \bar{E}\in \left\{{\bar{\psi }}_{\mathrm{in}},{\bar{\psi }}_{\mathrm{rad}},{\bar{\psi }}_{s},{\bar{J}}_{t},{\bar{J}}_{s}\right\},\;\bar{\bar{K}}\in \left\{\bar{\bar{G}},{\bar{\bar{F}}}_{t},{\bar{\bar{F}}}_{s},{\bar{\bar{F}}}_{r}\right\},\\ \; & \bar{R}\in \left\{{\bar{\psi }}_{\mathrm{rad}},{\bar{\psi }}_{s},{\bar{J}}_{t},{\bar{J}}_{s},{\bar{J}}_{r}\right\},\;D\in \left\{S_{t},S_{s},S_{r},S_{t}\cup S_{s}\cup S_{r}\right\},\;V\in \left\{S_{t},S_{s},S_{r},V_{\mathrm{ex}}\right\}, \end{array}$$

where $V_{\mathrm{ex}}$ is a defined by (4).

Equation (A89) is equivalent to multiple scalar integrals, each of the form

(A91) $$R\left(x,\omega ,\varpi \right)=\int_{x^{\prime }\in D}d^{2}x^{\prime }K\left(x,x^{\prime },\omega \right)E^{\prime }\left(x^{\prime },\omega ,\varpi \right).$$

Here, all possible combinations are exhausted by the following list of data structures associated with (A91):

(A92) $$\begin{array}{cc} \; & E\in \left\{\psi_{d,r}^{i}|i\in \left\{1,2\right\},d\in \left\{\mathrm{in},\mathrm{rad},s\right\},r\in \left\{e,m\right\}\right\},\\ \; & K\in \left\{G_{c}^{ij},F_{b,c}^{i^{\prime }j^{\prime }}|b\in \left\{t,s,r\right\},c\in \left\{\mathrm{ee},\mathrm{em},\mathrm{me},\mathrm{mm}\right\},i,j\in \left\{1,2,3\right\},i^{\prime },j^{\prime }\in \left\{1,2\right\}\right\},\\ \; & R\in \left\{J_{b,r}^{i},\psi_{d,r}^{i}|b\in \left\{t,s,r\right\},i\in \left\{1,2\right\},d\in \left\{\mathrm{rad},s\right\},r\in \left\{e,m\right\}\right\}. \end{array}$$

In other words, (A92) is the full unpacking of (A90) based on Table 3.

### Appendix K.2. Proving That *(A91)* Is Well-Defined as a Stochastic Integral

Here, we establish that for all cases enumerated in (A92), the integral expression (A91) is well-defined as a mean-square integral in the sense specified by Definition A5. We begin by noting that, since the three 2-manifolds $S_{t}$, $S_{s}$, and $S_{r}$ are assumed to be fixed (deterministic), the integral in (A91) can be expressed in the form (A24), as the latter only requires the integrand to be an RF. It remains to be demonstrated that the other regularity conditions are satisfied. We distinguish two cases:

- Case I: Here, $\bar{\bar{K}}\left(x,x^{\prime },\omega \right)$ denotes one of the three current GFs ${\bar{\bar{F}}}_{b}\left(x,x^{\prime },\omega \right)$, where b∈{t,s,r}. In this context, the response field ${\bar{J}}_{b}\left(x,\omega \right)$ is defined for $x\in V=S_{b}$, and $D=S_{b}$ as specified in Definition A5. Utilizing Theorem A7, we replace $F_{b}^{ij}\left(x,x^{\prime },\omega \right)$, where i,j∈{1,2}, with a smooth approximation kernel function for all b∈{t,s,r}. As a result, the kernel function is automatically piecewise continuous. Furthermore, since the integration region $D=S_{b}$ is compact, the piecewise continuous function $K(x,x^{\prime },\omega )$ achieves a global maximum for $x^{\prime }\in D$ and is thus bounded. Therefore, the two regularity properties required for the non-random kernel function in Definition A5—boundedness and piecewise continuity—are satisfied. Consequently, we can interpret the integral (A91) as a stochastic integration in the mean-square sense.
- Case II: Here, we consider the scenario where $\bar{\bar{K}}\left(x,x^{\prime },\omega \right)$ represents $\bar{\bar{G}}\left(x,x^{\prime },\omega \right)$. In this case, we have $V=V_{\mathrm{ex}}$ and $D={⋃}_{b}S_{b}$. Hence, the kernel $K(x,x^{\prime },\omega )$ is piecewise continuous for all $x^{\prime }\in D$ in the context of $G^{ij}\left(x,x^{\prime },\omega \right)$ whenever x∈V (see Remark 2).

We may summarize this stage as follows. The kernel function is given by

(A93) $$\bar{\bar{K}}\left(x,x^{\prime },\omega \right)=\left\{\begin{array}{c} {\bar{\bar{F}}}_{b}\left(x,x^{\prime },\omega \right),\;D=S_{b},V=S_{b}\;\;\;\;\;\left(\mathrm{Case}\;I\right)\\ \bar{\bar{G}}\left(x,x^{\prime },\omega \right),\;\;D=\underset{b}{⋃}S_{b},V=V_{\mathrm{ex}}\;\left(\mathrm{Case}\;\mathrm{II}\right) \end{array}\right.$$

and we proved that the map $\left(x,x^{\prime }\right)\mapsto \bar{\bar{K}}\left(x,x^{\prime },\omega \right)$ satisfies the regularity conditions given in Definition A5.

### Appendix K.3. Proving the Existence of the m.s. Stochastic Integral for a Given Input

In this stage, we demonstrate the existence of the mean-square (m.s.) stochastic integral (A91), defining the response function R(x,ω,ϖ) as an RF for x∈V when excited by a generic RF E(x,ω,ϖ) with continuous mean and covariance/pseudo-covariance functions. (In the final stage of the proof, we employ the results from this stage and finite induction to reach our concluding results.)

We utilize Theorem A6. Recall that from the previous stage in our proof, we have established that the map $\left(x,x^{\prime }\right)\mapsto K\left(x,x^{\prime },\omega \right)$ is continuous on its domain V×D. Furthermore, the mean function of the input RF, $x\mapsto E\{\psi_{\mathrm{in}}\left(x,\omega ,\varpi \right)\}$, and the covariance function $\left(x^{\prime },y^{\prime }\right)\mapsto C_{E}\left(x^{\prime },y^{\prime },\omega \right)$ are continuous on their respective domains by assumption at this stage. Hence, condition (A28) is immediately satisfied because *D* is compact. For condition (A29), it transforms into

(A94) $$\int_{D}\int_{D}d^{2}x^{\prime }\;d^{2}y^{\prime }\;K\left(x,x^{\prime }\right)K^{*}\left(x,y^{\prime }\right)C_{\psi_{\mathrm{in}}}\left(x^{\prime },y^{\prime }\right)<\infty ,$$

which holds in our case because the integrand is continuous in $x^{\prime }$ and $y^{\prime }$ for all x∈V, while *D* is compact. Therefore, by Theorem A6, the mean-square stochastic integral (A91) exists, and the generic response function R(x,ω,ϖ) becomes an RF for x∈V hence $\bar{R}\left(x,\omega ,\varpi \right)$ also becomes an RF. We summarize this stage as follows. The generic response field

(A95) $$\bar{R}\left(x,\omega ,\varpi \right)=\left\{\begin{array}{c} {\bar{J}}_{b}\left(x,\omega ,\varpi \right),\;\;b\in \left\{t,s,r\right\},x\in V=S_{b}\;\;\;\;\\ {\bar{\psi }}_{c}\left(x,\omega ,\varpi \right),\;\;c\in \left\{\mathrm{in},s\right\},\;x\in V=V_{\mathrm{ex}} \end{array}\right.$$

is a second-order RF.

### Appendix K.4. Proving That the Output RF Has Continuous Mean, Covariance, and Pseudo-Covariance Functions

In the previous stage, we established that if the input RF E(x,ω,ϖ) has continuous mean and covariance functions, then the response field R(x,ω,ϖ) is also an RF. Here, we demonstrate that in addition the response RF also possesses continuous mean and covariance/pseudo-covariance functions as well. If the pseudo-covariance of the input field is also continuous, then the response RF’s pseudo-covariance is continuous as well. These results are collected in Proposition A6.

**Proposition** **A6.**
*Consider an RF $\psi_{\mathrm{in}}$ with continuous mean, covariance, and pseudo-covariance. Let a response RF R be defined as in *(A91)*, with the response function K specified as above. Then, the response RF R will possess continuous mean, covariance, and pseudo-covariance functions.*

**Proof.** By Proposition A4, the mean, covariance, and pseudo-covariance functions of a second-order complex-valued vector RF, construed as an m.s. integral in the sense of Definition A5, are expressed by (A26), (A67), and (A68), respectively. We now invoke Theorem A1 to demonstrate the spatial continuity of these functions. Conditions 1 and 2 of that theorem are trivially satisfied in our case. For Condition 3, we observe that the integrands are continuous over a compact domain (as established in earlier stages of the proof), and the functions involving integration variables—namely x∈D for (A26), and $(x,x^{\prime })\in D\times D$ for (A27) and (A68)—attain a maximum value *M* on this domain. Given that the constant function with value *M* is integrable, Condition 3 is satisfied. Consequently, we conclude that when the input RF possesses continuous mean, covariance, and pseudo-covariance functions, the resulting RF also exhibits continuity in its corresponding functions. □

### Appendix K.5. Induction on All Stages

Finally, we synthesize the preceding steps to inductively establish that all output fields *R* listed in (A92) are indeed RFs possessing continuous mean and covariance functions. This result follows from carefully tracing all causal chains in the three processes (41), (42), and (43).

The above analysis is valid for all $\omega \in R^{+}$.

## Appendix L. Proof of Theorem 1

This follows directly from condition 2 in Definition 4 and Lemma A1, which asserts that mean-square continuity of a complex-valued RF is equivalent to the diagonal continuity of its corresponding mean, covariance and pseudo-covariance functions. We have the following three distinct cases:

1. For ${\bar{\psi }}_{\mathrm{in}}\left(x,\omega ,\varpi \right)$, the covariance function $C_{{\bar{\psi }}_{\mathrm{in}}}\left(x,x^{\prime },\omega \right)$ by assumption has the property that the map
  (A96) $$\left(x,x^{\prime }\right)\mapsto C_{{\bar{\psi }}_{\mathrm{in}}}\left(x,x^{\prime },\omega \right)$$
  is continuous on its entire domain, and therefore it is continuous for the special diagonal case $x=x^{\prime }\in S_{t}$. The above result holds equally for the pseudo-covariance function, following precisely the same details. Hence by Lemma A1, the RF under examination is m.s. continuous.
2. For ${\bar{\bar{F}}}_{b}\left(x,x^{\prime },\omega ,\varpi \right),b\in \left\{t,s,r\right\}$, the map
  (A97) $$\left(\left(x_{1},x_{1}^{\prime }\right),\left(x_{2},x_{2}^{\prime }\right)\right)\mapsto C_{{\bar{\bar{F}}}_{b}}\left(x_{1},x_{1}^{\prime },x_{2},x_{2}^{\prime },\omega \right)$$
  is continuous on its entire domain, and hence also continuous for the special diagonal case $\left(x_{1},x_{1}^{\prime }\right)=\left(x_{2},x_{2}^{\prime }\right)\in S_{b}\times S_{b}$. The above result holds equally for the pseudo-covariance function, following precisely the same details. Hence by Lemma A1, the RF under examination is m.s. continuous. [Note that by Theorem A7, the current GFs ${\bar{\bar{F}}}_{b}\left(x,x^{\prime },\omega ,\varpi \right),b\in \left\{t,s,r\right\}$, can always be assumed continuous in the deterministic case, including when $x=x^{\prime }$, in contrast to the medium GF $\bar{\bar{G}}\left(x,x^{\prime },\omega ,\varpi \right)$ (see below).]
3. For $\bar{\bar{G}}\left(x,x^{\prime },\omega ,\varpi \right)$, the function
  (A98) $$\left(\left(x_{1},x_{1}^{\prime }\right),\left(x_{2},x_{2}^{\prime }\right)\right)\mapsto C_{\bar{\bar{G}}}\left(x_{1},x_{1}^{\prime },x_{2},x_{2}^{\prime },\omega \right)$$
  is continuous for
  (A99) $$\left(\left(x_{1},x_{1}^{\prime }\right),\left(x_{2},x_{2}^{\prime }\right)\right)\in \left(V\times S\right)\times \left(V\times S\right),$$
  where $V=R^{3}\setminus {⋃}_{b}V_{b}$ and $S={⋃}_{b}S_{b}$, b∈{t,s,r}, and therefore it is continuous for the special diagonal case $\left(x_{1},x_{1}^{\prime }\right)=\left(x_{2},x_{2}^{\prime }\right)\in \left(V\times S\right)$. The above result holds equally for the pseudo-covariance function, following precisely the same details. Hence by Lemma A1, the RF under examination is m.s. continuous. [Note that even though $\bar{\bar{G}}\left(x,x^{\prime },\omega ,\varpi \right)$ is *not* continuous for $x=x^{\prime }$, this case is already excluded through our careful definition of the region *V* as the exterior domain of the medium GF $\bar{\bar{G}}\left(x,x^{\prime },\omega ,\varpi \right)$. See Remark 2.]

The above analysis is valid for all $\omega \in R^{+}$.

## Appendix M. Proof of Theorem 3

We employ the concept of m.s. integration, as used in the proof of Theorem 2. As before, all response RFs can be represented in the form given by (A89). This expression can be further decomposed into scalar equations, analogous to (A91), each involving sums of multiple scalar integrals, now expressed in a slightly modified form

(A100) $$R\left(x,\omega ,\varpi \right)=\int_{x^{\prime }\in D}d^{2}x^{\prime }K^{\prime }\left(x,x^{\prime },\omega \right)E^{\prime }\left(x^{\prime },\omega ,\varpi \right),$$

where we set

(A101) $$K^{\prime }\left(x,x^{\prime },\omega \right)=1,$$

while we define

(A102) $$E^{\prime }\left(x^{\prime },\omega ,\varpi \right):=K\left(x,x^{\prime },\omega ,\varpi \right)E\left(x^{\prime },\omega ,\varpi \right).$$

Here, $E(x^{\prime },\omega ,\varpi )$ denotes the input RF interacting with the system over the domain *D*, while the stochastic GF $K(x,x^{\prime },\omega ,\varpi )$ are as listed in Table 4.

The structure of the proof for the present theorem adheres to the same logical framework as that found in Theorem 2, albeit with certain modifications, which we delineate below. For the proof of the existence of second-order RF via an application of Theorem A5 (stage 3 in the proof of Theorem 2), once again, we must distinguish between two cases:

- Case I: The GF $K(x,x^{\prime },\omega ,\varpi )$ is replaced by $F_{b}\left(x,x^{\prime },\omega ,\varpi \right),b\in \left\{t,\;s,\;r\right\}$. We set $V=D=S_{b}$ and observe that, by Theorem A6, the stochastic integral in (A24) exists if and only if the mean and covariance conditions corresponding to (A28) and (A29), respectively, are satisfied. We now proceed to explicitly work out the latter condition (the former condition on the mean is similar), which, in view of (A100), takes the following form:
  (A103) $$\begin{array}{cc} \; & \int_{D}\int_{D}d^{2}x^{\prime }\;d^{2}y^{\prime }\;K^{\prime }\left(x,x^{\prime },\omega \right)K^{\prime *}\left(y,y^{\prime },\omega \right)C_{E^{\prime }}\left(x^{\prime },y^{\prime },\omega \right) \end{array}$$
  (A104) $$\begin{array}{cc} = & \int_{D}\int_{D}d^{2}x^{\prime }\;d^{2}y^{\prime }\;C_{E^{\prime }}\left(x^{\prime },y^{\prime },\omega \right) \end{array}$$
  (A105) $$\begin{array}{cc} = & \int_{D}\int_{D}d^{2}x^{\prime }\;d^{2}y^{\prime }\;C_{F_{b}}\left(x,x^{\prime },y,y^{\prime },\omega \right)C_{E}\left(x^{\prime },y^{\prime },\omega \right) \end{array}$$
  (A106) $$\begin{array}{cc} \; & <\infty . \end{array}$$
  The expression in (A103) derives from the condition stated in (A29), as specified in Theorem A6. The equality in (A104) follows from (A101), while (A105) arises from the stochastic independence between the stochastic GF and the interacting RF, as stipulated in Definition 4. Finally, the bound in (A106) is established through the continuity of the covariance functions $C_{F_{b}}\left(x,x^{\prime },y,y^{\prime },\omega \right)$ and $C_{E}\left(x^{\prime },y^{\prime },\omega \right)$, which are valid for all $x^{\prime },y^{\prime }\in S_{b}$ for each selection of the pair x,y∈V (as stipulated in Definition 4 and stages 1 and 2 in the proof of Theorem 2) and the compactness of $D=S_{b}$. Consequently, the integral in (A91) exists, resulting in an RF defined on $V=D=S_{b}$ for all induced currents $J_{b}\left(x,\omega ,\varpi \right)$.
- Case II: The GF $K(x,x^{\prime },\omega ,\varpi )$ is replaced by $G_{b^{\prime }}\left(x,x^{\prime },\omega ,\varpi \right),b^{\prime }\in \left\{t,s\right\}$. The proof for Case II follows a very similar structure compared with Case I above, with the key distinction that the resulting RFs $\psi_{\mathrm{rad}}\left(x,\omega ,\varpi \right)$ and $\psi_{s}\left(x,\omega ,\varpi \right)$ are defined for $x\in V=V_{\mathrm{ex}}=R^{3}\setminus {⋃}_{b}V_{b}$, while $D={⋃}_{b}S_{b}$.

Next, stages 4 and 5 of the proof of the current theorem follow a logic that closely resembles the corresponding stages in Theorem 2; thus, we omit the full details for the sake of brevity. However, note that for stage 5, induction is performed over the causal chains (52), (53), and (54). The analysis presented above is applicable for all $\omega \in R^{+}$.

## Appendix N. Proof of Theorem 4

Here, we present the detailed calculations necessary for the complete proof of Theorem 4. The central idea of the proof involves a trace technique to decouple the correlation flow, effectively restructuring it into a directed input–output relation. As shown below, this decoupling is achievable solely through the complete EM response functions introduced in Section 2.2.

The proof of Formula (64) is straightforward and is left for the reader. To prove the remaining formulas, we start by first deriving general expressions for the *ij*th element of the covariance/pseudo-covariance matrix in terms of the LoS and NLoS components of the received current. Substituting (25) into (61) yields:

(A107) $$\begin{array}{cc} R_{{\bar{J}}_{r}}^{ij}\left(x,x^{\prime },\omega \right) & =\;\;\;E\left\{{\bar{J}}_{r}^{†,\mathrm{los}}\left(x^{\prime },\omega ,\varpi \right)\cdot {\bar{a}}_{j}\;{\bar{a}}_{i}^{†}\cdot {\bar{J}}_{r}^{\mathrm{los}}\left(x,\omega ,\varpi \right)\right\}\\ \; & \;+E\left\{{\bar{J}}_{r}^{†,\mathrm{nlos}}\left(x^{\prime },\omega ,\varpi \right)\cdot {\bar{a}}_{j}\;{\bar{a}}_{i}^{†}\cdot {\bar{J}}_{r}^{\mathrm{nlos}}\left(x,\omega ,\varpi \right)\right\}\\ \; & \;+E\left\{{\bar{J}}_{r}^{†,\mathrm{nlos}}\left(x^{\prime },\omega ,\varpi \right)\cdot {\bar{a}}_{j}\;{\bar{a}}_{i}^{†}\cdot {\bar{J}}_{r}^{\mathrm{los}}\left(x,\omega ,\varpi \right)\right\}\\ \; & \;+E\left\{{\bar{J}}_{r}^{†,\mathrm{los}}\left(x^{\prime },\omega ,\varpi \right)\cdot {\bar{a}}_{j}\;{\bar{a}}_{i}^{†}\cdot {\bar{J}}_{r}^{\mathrm{nlos}}\left(x,\omega ,\varpi \right)\right\}. \end{array}$$

Similarly, substituting (25) into (62) gives the following expansion for the pseudo-correlation matrix element:

(A108) $$\begin{array}{cc} {R^{\prime }}_{{\bar{J}}_{r}}^{ij}\left(x,x^{\prime },\omega \right) & =\;\;\;E\left\{{\bar{J}}_{r}^{⊤,\mathrm{los}}\left(x^{\prime },\omega ,\varpi \right)\cdot {\bar{a}}_{j}\;{\bar{a}}_{i}^{⊤}\cdot {\bar{J}}_{r}^{\mathrm{los}}\left(x,\omega ,\varpi \right)\right\}\\ \; & \;\;\;+E\left\{{\bar{J}}_{r}^{⊤,\mathrm{nlos}}\left(x^{\prime },\omega ,\varpi \right)\cdot {\bar{a}}_{j}\;{\bar{a}}_{i}^{⊤}\cdot {\bar{J}}_{r}^{\mathrm{nlos}}\left(x,\omega ,\varpi \right)\right\}\\ \; & \;\;\;+E\left\{{\bar{J}}_{r}^{⊤,\mathrm{nlos}}\left(x^{\prime },\omega ,\varpi \right)\cdot {\bar{a}}_{j}\;{\bar{a}}_{i}^{⊤}\cdot {\bar{J}}_{r}^{\mathrm{los}}\left(x,\omega ,\varpi \right)\right\}\\ \; & \;\;\;+E\left\{{\bar{J}}_{r}^{⊤,\mathrm{los}}\left(x^{\prime },\omega ,\varpi \right)\cdot {\bar{a}}_{j}\;{\bar{a}}_{i}^{⊤}\cdot {\bar{J}}_{r}^{\mathrm{nlos}}\left(x,\omega ,\varpi \right)\right\}. \end{array}$$

Note that from the complex correlation and pseudo-correlation matrices of a complex vector field, whose elements are given by (A107) and (A108), we can always compute the full *real* correlation matrix of the real vector representation ${\bar{J}}_{r}^{\left(r\right)}$ using the formulas in Table A2. Consequently, there is no loss of generality when we focus on the use of correlation and pseudo-correlation of complex RFs in the following discussion.

By substituting (27) into the definition of correlation in (61), the *ij*th correlation matrix element involves first the following quantity, which we compute first:

(A109) $$\begin{array}{l} \;\;\;{\bar{J}}_{r}^{†,\mathrm{nlos}}\left(x^{\prime },\omega ,\varpi \right)\cdot {\bar{a}}_{j}\;{\bar{a}}_{i}^{†}\cdot {\bar{J}}_{r}^{\mathrm{nlos}}\left(x,\omega ,\varpi \right)\\ =\int_{S_{r}}d^{2}x_{1}\int_{S_{s}}d^{2}x_{2}\int_{S_{s}}d^{2}x_{3}\int_{S_{t}}d^{2}x_{4}\int_{S_{t}}d^{2}x_{5}\int_{S_{r}}d^{2}x_{1}^{\prime }\int_{S_{s}}d^{2}x_{2}^{\prime }\int_{S_{s}}d^{2}x_{3}^{\prime }\int_{S_{t}}d^{2}x_{4}^{\prime }\int_{S_{t}}d^{2}x_{5}^{\prime }\\ \times {\bar{\psi }}_{\mathrm{in}}^{†}\left(x_{5}^{\prime }\right)\cdot {\bar{\bar{S}}}_{\mathrm{nlos}}^{†}\left(x^{\prime },x_{1}^{\prime },x_{2}^{\prime },x_{3}^{\prime },x_{4}^{\prime },x_{5}^{\prime },\omega ,\varpi \right)\cdot {\bar{a}}_{j}\;{\bar{a}}_{i}^{†}\cdot {\bar{\bar{S}}}_{\mathrm{nlos}}\left(x,x_{1},x_{2},x_{3},x_{4},x_{5},\omega ,\varpi \right)\cdot {\bar{\psi }}_{\mathrm{in}}\left(x_{5},\omega ,\varpi \right)\\ =\int_{S_{r}}d^{2}x_{1}\int_{S_{s}}d^{2}x_{2}\int_{S_{s}}d^{2}x_{3}\int_{S_{t}}d^{2}x_{4}\int_{S_{t}}d^{2}x_{5}\int_{S_{r}}d^{2}x_{1}^{\prime }\int_{S_{s}}d^{2}x_{2}^{\prime }\int_{S_{s}}d^{2}x_{3}^{\prime }\int_{S_{t}}d^{2}x_{4}^{\prime }\int_{S_{t}}d^{2}x_{5}^{\prime }\\ \times \mathrm{Tr}\left\{{\bar{\psi }}_{\mathrm{in}}^{†}\left(x_{5}^{\prime },\omega ,\varpi \right)\cdot {\bar{\bar{S}}}_{\mathrm{nlos}}^{†}\left(x^{\prime },x_{1}^{\prime },x_{2}^{\prime },x_{3}^{\prime },x_{4}^{\prime },x_{5}^{\prime },\omega ,\varpi \right)\cdot {\bar{a}}_{j}\;{\bar{a}}_{i}^{†}\cdot {\bar{\bar{S}}}_{\mathrm{nlos}}\left(x,x_{1},x_{2},x_{3},x_{4},x_{5},\omega ,\varpi \right)\cdot {\bar{\psi }}_{\mathrm{in}}\left(x_{5},\omega ,\varpi \right)\right\}\\ =\int_{S_{r}}d^{2}x_{1}\int_{S_{s}}d^{2}x_{2}\int_{S_{s}}d^{2}x_{3}\int_{S_{t}}d^{2}x_{4}\int_{S_{t}}d^{2}x_{5}\int_{S_{r}}d^{2}x_{1}^{\prime }\int_{S_{s}}d^{2}x_{2}^{\prime }\int_{S_{s}}d^{2}x_{3}^{\prime }\int_{S_{t}}d^{2}x_{4}^{\prime }\int_{S_{t}}d^{2}x_{5}^{\prime }\\ \times \mathrm{Tr}\left\{{\bar{\bar{S}}}_{\mathrm{nlos}}^{†}\left(x^{\prime },x_{1}^{\prime },x_{2}^{\prime },x_{3}^{\prime },x_{4}^{\prime },x_{5}^{\prime },\omega ,\varpi \right)\cdot {\bar{a}}_{j}\;{\bar{a}}_{i}^{†}\cdot {\bar{\bar{S}}}_{\mathrm{nlos}}\left(x,x_{1},x_{2},x_{3},x_{4},x_{5},\omega ,\varpi \right)\cdot \left[{\bar{\psi }}_{\mathrm{in}}\left(x_{5},\omega ,\varpi \right)\;{\bar{\psi }}_{\mathrm{in}}^{†}\left(x_{5}^{\prime },\omega ,\varpi \right)\right]\right\}. \end{array}$$

For writing the second equality in (A109), we applied the trace trick by re-expressing the scalar as its own trace. We first outline the main idea informally then prove it rigoursly in Lemma A2. The equality

(A110) $${\bar{\psi }}_{\mathrm{in}}^{†}\cdot {\bar{\bar{S}}}_{\mathrm{nlos}}^{†}\cdot {\bar{a}}_{j}{\bar{a}}_{i}^{†}\cdot {\bar{\bar{S}}}_{\mathrm{nlos}}\cdot {\bar{\psi }}_{\mathrm{in}}=\mathrm{Tr}\left\{{\bar{\psi }}_{\mathrm{in}}^{†}\cdot {\bar{\bar{S}}}_{\mathrm{nlos}}^{†}\cdot {\bar{a}}_{j}{\bar{a}}_{i}^{†}\cdot {\bar{\bar{S}}}_{\mathrm{nlos}}\cdot {\bar{\psi }}_{\mathrm{in}}\right\}$$

holds because the quantity ${\bar{\psi }}_{\mathrm{in}}^{†}\cdot {\bar{\bar{S}}}_{\mathrm{nlos}}^{†}\cdot {\bar{a}}_{j}{\bar{a}}_{i}^{†}\cdot {\bar{\bar{S}}}_{\mathrm{nlos}}\cdot {\bar{\psi }}_{\mathrm{in}}$ is a scalar. Next, for justifying the third equality in (A109), we made use of the cyclic property of the trace operator, namely Tr{ABC}=Tr{BCA} valid for any matrices A,B,C with proper dimensions. Here, we have $A={\bar{\psi }}_{\mathrm{in}}^{†}$, $B={\bar{\bar{S}}}_{\mathrm{nlos}}^{†}\cdot {\bar{a}}_{j}{\bar{a}}_{i}^{†}\cdot {\bar{\bar{S}}}_{\mathrm{nlos}}$, $C={\bar{\psi }}_{\mathrm{in}}$. Nevertheless, the argument above is not fully rigorous because there is an ambiguity regarding how to compute the trace operation above. This is due to the lack of complete clarity on whether the trace trick was performed relative to superdyads or regular dyads. In fact, as the next lemma shows, it is performed with respect to *both*. The correct expression for all trace formulas used in this paper is also given by Formula (A112).

**Lemma** **A2.**
*Let $\bar{b}$ and $\bar{d}$ be two supervectors while $\bar{\bar{S}}$ is a superdyad. Then the following identity holds:*

(A111)
$${\bar{b}}^{†}\cdot \bar{\bar{S}}\cdot \bar{d}=\mathrm{Tr}\left\{{\bar{b}}^{†}\cdot \bar{\bar{S}}\cdot \bar{d}\right\}=\mathrm{Tr}\left\{\bar{\bar{S}}\cdot \left(\bar{d}\;{\bar{b}}^{†}\right)\right\},$$

*where we assume that matrix dimensions match up. Let $S_{d}$ be the space of superdyads (i.e., complex-dyadic-valued 2×2 matrices; see Appendix A). The effective trace operator $\mathrm{Tr}:S_{d}\to C$ used in *(A111)* is defined by the formula*

(A112)
$$\mathrm{Tr}:={\mathrm{Tr}}_{C^{3\times 3}}{\mathrm{Tr}}_{S_{d}},$$

*with ${\mathrm{Tr}}_{S_{d}}$ and ${\mathrm{Tr}}_{C^{3\times 3}}$ being the trace operations taken with respect to the space of superdyads and dyads, respectively.*

**Proof.** We note that the first equality in (A111) is self-evident. We therefore focus on proving the second equality. We write the general terms of the supervectors $\bar{b}$ and $\bar{d}$ as $b_{i_{1}}^{j_{1}}$ and $d_{i_{2}}^{j_{2}}$, while the general element of $\bar{\bar{S}}$ is of the form $S_{i_{1}i_{2}}^{j_{1}j_{2}}$. (Note that here lower and upper indices are not for labeling vector spaces and their duals.) Here, $i_{1},i_{2}\in \left\{1,2\right\}$ are the supervector/superdyad index, while j={1,2,3} are the vector/dyad index. We then have

(A113) $${\bar{b}}^{†}\cdot \bar{\bar{S}}\cdot \bar{d}=\sum_{i_{1}i_{2}j_{1}j_{2}}b_{i_{1}}^{*j_{1}}S_{i_{1}i_{2}}^{j_{1}j_{2}}d_{i_{2}}^{j_{2}}.$$

On the other hand, let $\bar{\bar{V}}:=\bar{d}\;{\bar{b}}^{†}$ and $\bar{\bar{D}}:=\bar{\bar{S}}\cdot \bar{\bar{V}}$. We can write the general element of $\bar{\bar{D}}$ as

(A114) $$D_{i_{1}i_{3}}^{j_{1}j_{3}}=\sum_{i_{2}j_{2}}S_{i_{1}i_{2}}^{j_{1}j_{2}}V_{i_{2}i_{3}}^{j_{2}j_{3}}.$$

Calculations of ${\mathrm{Tr}}_{\mathrm{superarray}}$ and ${\mathrm{Tr}}_{C^{3\times 3}}$ are then equivalent to summing over $i_{1}=i_{3}$ and $j_{1}=j_{3}$, respectively, leading to

(A115) $$\mathrm{Tr}\left\{\bar{\bar{S}}\cdot \bar{\bar{V}}\right\}=\sum_{i_{1}i_{2}j_{1}j_{2}}S_{i_{1}i_{2}}^{j_{1}j_{2}}V_{i_{2}i_{1}}^{j_{2}j_{1}}=\sum_{i_{1}i_{2}j_{1}j_{2}}S_{i_{1}i_{2}}^{j_{1}j_{2}}d_{i_{2}}^{j_{2}}b_{i_{1}}^{*j_{1}}.$$

Comparing (A113) and (A115) finally yields (A111). □

From Lemma A2 we can easily see that all expressions like (A109) below involving the operator Tr as defined by (A112) should give a complex scalar as a final output, as expected for the correlation value. Using (A109) and (61), the first term in the RHS of (A107) can be put into the following form:

(A116) $$\begin{array}{cc} R_{\bar{J}}^{ij,\mathrm{nlos}}\left(x,x^{\prime },\omega \right) & :=E\left\{{\bar{J}}_{r}^{†,\mathrm{nlos}}\left(x^{\prime },\omega ,\varpi \right)\cdot {\bar{a}}_{j}\;{\bar{a}}_{i}^{†}\cdot {\bar{J}}_{r}^{\mathrm{nlos}}\left(x,\omega ,\varpi \right)\right\}\\ = & \mathrm{Tr}\int_{S_{r}}d^{2}x_{1}\int_{S_{s}}d^{2}x_{2}\int_{S_{s}}d^{2}x_{3}\int_{S_{t}}d^{2}x_{4}\int_{S_{t}}d^{2}x_{5}\int_{S_{r}}d^{2}x_{1}^{\prime }\int_{S_{s}}d^{2}x_{2}^{\prime }\int_{S_{s}}d^{2}x_{3}^{\prime }\int_{S_{t}}d^{2}x_{4}^{\prime }\int_{S_{t}}d^{2}x_{5}^{\prime }\\ \; & \times {\bar{\bar{P}}}_{ij}^{\mathrm{nlos}}\left(x,x_{1},x_{2},x_{3},x_{4},x_{5},x^{\prime },x_{1}^{\prime },x_{2}^{\prime },x_{3}^{\prime },x_{4}^{\prime },x_{5}^{\prime },\omega \right)\cdot {\bar{\bar{R}}}_{{\bar{\psi }}_{\mathrm{in}}}\left(x_{5},x_{5}^{\prime },\omega \right), \end{array}$$

where the superdyad ${\bar{\bar{R}}}_{{\bar{\psi }}_{\mathrm{in}}}$ is defined by (A78) while

(A117) $$\begin{array}{c} {\bar{\bar{P}}}_{ij}^{\mathrm{nlos}}\left(x,x_{1},x_{2},x_{3},x_{4},x_{5},x^{\prime },x_{1}^{\prime },x_{2}^{\prime },x_{3}^{\prime },x_{4}^{\prime },x_{5}^{\prime },\omega \right):=E\left\{{\bar{\bar{S}}}_{\mathrm{nlos}}^{†}\left(x^{\prime },x_{1}^{\prime },x_{2}^{\prime },x_{3}^{\prime },x_{4}^{\prime },x_{5}^{\prime },\omega ,\varpi \right)\cdot {\bar{a}}_{j}\;{\bar{a}}_{i}^{†}\cdot {\bar{\bar{S}}}_{\mathrm{nlos}}\left(x,x_{1},x_{2},x_{3},x_{4},x_{5},\omega ,\varpi \right)\right\}. \end{array}$$

By employing the same procedure employed for deducing (A109), we can derive the following expression for the second term in the RHS of (A107):

(A118) $$\begin{array}{cc} \; & R_{\bar{J}}^{ij,\mathrm{los}}\left(x,x^{\prime },\omega \right):=E\left\{{\bar{J}}_{r}^{H,\mathrm{los}}\left(x^{\prime },\omega ,\varpi \right)\cdot {\bar{a}}_{j}\;{\bar{a}}_{i}^{†}\cdot {\bar{J}}_{r}^{\mathrm{los}}\left(x,\omega ,\varpi \right)\right\}\\ \; & =\mathrm{Tr}\int_{S_{r}}d^{2}x_{1}\int_{S_{t}}d^{2}x_{4}\int_{S_{t}}d^{2}x_{5}\int_{S_{r}}d^{2}x_{1}^{\prime }\int_{S_{t}}d^{2}x_{4}^{\prime }\int_{S_{t}}d^{2}x_{5}^{\prime }\;{\bar{\bar{P}}}_{ij}^{\mathrm{los}}\left(x,x_{1},x_{4},x_{5},x^{\prime },x_{1}^{\prime },x_{4}^{\prime },x_{5}^{\prime },\omega \right)\cdot R_{{\bar{\psi }}_{\mathrm{in}}}\left(x_{5},x_{5}^{\prime },\omega \right), \end{array}$$

where

(A119) $$\begin{array}{c} {\bar{\bar{P}}}_{ij}^{\mathrm{los}}\left(x,x_{1},x_{4},x_{5},x^{\prime },x_{1}^{\prime },x_{4}^{\prime },x_{5}^{\prime },\omega \right):=E\left\{{\bar{\bar{S}}}_{\mathrm{los}}^{†}\left(x^{\prime },x_{1}^{\prime },x_{4}^{\prime },x_{5}^{\prime },\omega ,\varpi \right)\cdot {\bar{a}}_{j}\;{\bar{a}}_{i}^{†}\cdot {\bar{\bar{S}}}_{\mathrm{los}}\left(x,x_{1},x_{4},x_{5},\omega ,\varpi \right)\right\}. \end{array}$$

Similarly, we can compute the third term in the RHS of (A107):

(A120) $$\begin{array}{cc} R_{\bar{J}}^{ij,\mathrm{los}/\mathrm{nlos}}\left(x,x^{\prime },\omega \right):= & E\left\{{\bar{J}}_{r}^{H,\mathrm{nlos}}\left(x^{\prime },\omega ,\varpi \right)\cdot {\bar{a}}_{j}\;{\bar{a}}_{i}^{†}\cdot {\bar{J}}_{r}^{\mathrm{los}}\left(x,\omega ,\varpi \right)\right\}\\ = & \mathrm{Tr}\int_{S_{r}}d^{2}x_{1}\int_{S_{t}}d^{2}x_{4}\int_{S_{t}}d^{2}x_{5}\int_{S_{r}}d^{2}x_{1}^{\prime }\int_{S_{s}}d^{2}x_{2}^{\prime }\int_{S_{s}}d^{2}x_{3}^{\prime }\int_{S_{t}}d^{2}x_{4}^{\prime }\int_{S_{t}}d^{2}x_{5}^{\prime }\\ \; & \times {\bar{\bar{P}}}_{ij}^{\mathrm{los}/\mathrm{nlos}}\left(x,x_{1},x_{4},x_{5},x^{\prime },x_{1}^{\prime },x_{2}^{\prime },x_{3}^{\prime },x_{4}^{\prime },x_{5}^{\prime },\omega \right)\cdot {\bar{\bar{R}}}_{{\bar{\psi }}_{\mathrm{in}}}\left(x_{5},x_{5}^{\prime },\omega \right), \end{array}$$

where

(A121) $$\begin{array}{c} {\bar{\bar{P}}}_{ij}^{\mathrm{los}/\mathrm{nlos}}\left(x,x_{1},x_{4},x_{5},x^{\prime },x_{1}^{\prime },x_{2}^{\prime },x_{3}^{\prime },x_{4}^{\prime },x_{5}^{\prime },\omega \right):=E\left\{{\bar{\bar{S}}}_{\mathrm{nlos}}^{†}\left(x^{\prime },x_{1}^{\prime },x_{2}^{\prime },x_{3}^{\prime },x_{4}^{\prime },x_{5}^{\prime },\omega ,\varpi \right)\cdot {\bar{a}}_{j}\;{\bar{a}}_{i}^{†}\cdot {\bar{\bar{S}}}_{\mathrm{los}}\left(x,x_{1},x_{4},x_{5},\omega ,\varpi \right)\right\}. \end{array}$$

Finally, the fourth term in the RHS of (A107) can be expressed as

(A122) $$\begin{array}{cc} R_{\bar{J}}^{ij,\mathrm{nlos}/\mathrm{los}}\left(x,x^{\prime },\omega \right):= & E\left\{{\bar{J}}_{r}^{H,\mathrm{los}}\left(x^{\prime },\omega ,\varpi \right)\cdot {\bar{a}}_{j}\;{\bar{a}}_{i}^{†}\cdot {\bar{J}}_{r}^{\mathrm{nlos}}\left(x,\omega ,\varpi \right)\right\}\\ = & \mathrm{Tr}\int_{S_{r}}d^{2}x_{1}\int_{S_{s}}d^{2}x_{2}\int_{S_{s}}d^{2}x_{3}\int_{S_{t}}d^{2}x_{4}\int_{S_{t}}d^{2}x_{5}\int_{S_{r}}d^{2}x_{1}^{\prime }\int_{S_{t}}d^{2}x_{4}^{\prime }\int_{S_{t}}d^{2}x_{5}^{\prime }\\ \;\;\;\;\;\; & \times {\bar{\bar{P}}}_{ij}^{\mathrm{nlos}/\mathrm{los}}\left(x,x_{1},x_{2},x_{3},x_{4},x_{5},x^{\prime },x_{1}^{\prime },x_{4}^{\prime },x_{5}^{\prime },\omega \right)\cdot {\bar{\bar{R}}}_{{\bar{\psi }}_{\mathrm{in}}}\left(x_{5},x_{5}^{\prime },\omega \right), \end{array}$$

where

(A123) $$\begin{array}{c} {\bar{\bar{P}}}_{ij}^{\mathrm{nlos}/\mathrm{los}}\left(x,x_{1},x_{2},x_{3},x_{4},x_{5},x^{\prime },x_{1}^{\prime },x_{4}^{\prime },x_{5}^{\prime },\omega \right):=E\left\{{\bar{S}}_{\mathrm{los}}^{†}\left(x^{\prime },x_{1}^{\prime },x_{4}^{\prime },x_{5}^{\prime },\omega ,\varpi \right)\cdot {\bar{a}}_{j}\;{\bar{a}}_{i}^{†}\cdot {\bar{\bar{S}}}_{\mathrm{nlos}}\left(x,x_{1},x_{2},x_{3},x_{4},x_{5},\omega ,\varpi \right)\right\}. \end{array}$$

By substituting (A116), (A118), (A120), and (A130) into (A107), we obtain the relation (65). Using exactly the same procedure outlined above, the pseudo-correlation matrix elements $R_{\bar{J}}^{\prime ij,\mathrm{nlos}}$, $R_{\bar{J}}^{\prime ij,\mathrm{los}}$, $R_{\bar{J}}^{\prime ij,\mathrm{los}/\mathrm{nlos}}$, and $R_{\bar{J}}^{\prime ij,\mathrm{nlos}/\mathrm{los}}$, as defined in (A73), are given by expressions identical to (A116), (A118), (A120), and (A130), respectively, but with the Hermitian operation † replaced by the transpose operation ⊤. Therefore, (66) can be derived. This completes the proof of Theorem 4.

## Appendix O. Proof of Theorem 5

We apply Fubini theorem to rearrange the integrals in (A116) and (A117) in order to obtain

(A124) $$\begin{array}{cc} R_{\bar{J}}^{ij,\mathrm{nlos}}\left(x,x^{\prime },\omega \right)=\; & \mathrm{Tr}\int_{S_{t}}d^{2}x_{5}\int_{S_{t}}d^{2}x_{5}^{\prime }\;{\bar{\bar{K}}}_{ij}^{\mathrm{nlos}}\left(x,x^{\prime },x_{5},x_{5}^{\prime },\omega \right)\cdot {\bar{\bar{R}}}_{{\bar{\psi }}_{\mathrm{in}}}\left(x_{5},x_{5}^{\prime },\omega \right), \end{array}$$

where

(A125) $$\begin{array}{cc} {\bar{\bar{K}}}_{ij}^{\mathrm{nlos}}\left(x,x^{\prime },x_{5},x_{5}^{\prime },\omega \right) & :=\int_{S_{r}}d^{2}x_{1}\int_{S_{s}}d^{2}x_{2}\int_{S_{s}}d^{2}x_{3}\int_{S_{t}}d^{2}x_{4}\int_{S_{r}}d^{2}x_{1}^{\prime }\int_{S_{s}}d^{2}x_{2}^{\prime }\int_{S_{s}}d^{2}x_{3}^{\prime }\int_{S_{t}}d^{2}x_{4}^{\prime }\\ \; & \times E\left\{{\bar{\bar{S}}}_{\mathrm{nlos}}^{†}\left(x^{\prime },x_{1}^{\prime },x_{2}^{\prime },x_{3}^{\prime },x_{4}^{\prime },x_{5}^{\prime },\omega ,\varpi \right)\cdot {\bar{a}}_{j}\;{\bar{a}}_{i}^{†}\cdot {\bar{\bar{S}}}_{\mathrm{nlos}}\left(x,x_{1},x_{2},x_{3},x_{4},x_{5},\omega ,\varpi \right)\right\}. \end{array}$$

Similarly, from (A118) and (A119) we obtain

(A126) $$\begin{array}{ccc} \; & R_{\bar{J}}^{ij,\mathrm{los}}\left(x,x^{\prime },\omega \right) & =\mathrm{Tr}\int_{S_{t}}d^{2}x_{5}\int_{S_{r}}d^{2}x_{1}^{\prime }\int_{S_{t}}d^{2}x_{4}^{\prime }\int_{S_{t}}d^{2}x_{5}^{\prime }\;{\bar{\bar{K}}}_{ij}^{\mathrm{los}}\left(x,x^{\prime },x_{5},x_{5}^{\prime },\omega \right)\cdot {\bar{\bar{R}}}_{{\bar{\psi }}_{\mathrm{in}}}\left(x_{5},x_{5}^{\prime },\omega \right), \end{array}$$

where

(A127) $$\begin{array}{cc} {\bar{\bar{K}}}_{ij}^{\mathrm{los}}\left(x,x^{\prime },x_{5},x_{5}^{\prime },\omega \right):= & \int_{S_{r}}d^{2}x_{1}\int_{S_{t}}d^{2}x_{4}\int_{S_{r}}d^{2}x_{1}^{\prime }\int_{S_{t}}d^{2}x_{4}^{\prime }\;E\left\{{\bar{\bar{S}}}_{\mathrm{los}}^{†}\left(x^{\prime },x_{1}^{\prime },x_{4}^{\prime },x_{5}^{\prime },\omega ,\varpi \right)\cdot {\bar{a}}_{j}\;{\bar{a}}_{i}^{†}\cdot {\bar{\bar{S}}}_{\mathrm{los}}\left(x,x_{1},x_{4},x_{5},\omega ,\varpi \right)\right\}. \end{array}$$

And from (A120) and (A121) we obtain

(A128) $$\begin{array}{cc} R_{\bar{J}}^{ij,\mathrm{los}/\mathrm{nlos}}\left(x,x^{\prime },\omega \right)\; & =\mathrm{Tr}\int_{S_{t}}d^{2}x_{5}\int_{S_{t}}d^{2}x_{5}^{\prime }\;{\bar{\bar{K}}}_{ij}^{\mathrm{los}/\mathrm{nlos}}\left(x,x^{\prime },x_{5},x_{5}^{\prime },\omega \right)\cdot {\bar{\bar{R}}}_{{\bar{\psi }}_{\mathrm{in}}}\left(x_{5},x_{5}^{\prime },\omega \right), \end{array}$$

where

(A129) $$\begin{array}{cc} {\bar{\bar{K}}}_{ij}^{\mathrm{los}/\mathrm{nlos}}\left(x,x^{\prime },x_{5},x_{5}^{\prime },\omega \right):= & \int_{S_{r}}d^{2}x_{1}\int_{S_{t}}d^{2}x_{4}\int_{S_{r}}d^{2}x_{1}^{\prime }\int_{S_{s}}d^{2}x_{2}^{\prime }\int_{S_{s}}d^{2}x_{3}^{\prime }\int_{S_{t}}d^{2}x_{4}^{\prime }\\ \; & \times E\left\{{\bar{\bar{S}}}_{\mathrm{nlos}}^{†}\left(x^{\prime },x_{1}^{\prime },x_{2}^{\prime },x_{3}^{\prime },x_{4}^{\prime },x_{5}^{\prime },\omega ,\varpi \right)\cdot {\bar{a}}_{j}\;{\bar{a}}_{i}^{†}\cdot {\bar{\bar{S}}}_{\mathrm{los}}\left(x,x_{1},x_{4},x_{5},\omega ,\varpi \right)\right\}. \end{array}$$

Finally, from (A130) and (A131) we arrive at

(A130) $$\begin{array}{c} R_{\bar{J}}^{ij,\mathrm{nlos}/\mathrm{los}}\left(x,x^{\prime },\omega \right)=\mathrm{Tr}\int_{S_{t}}d^{2}x_{5}\int_{S_{t}}d^{2}x_{5}^{\prime }\;{\bar{\bar{K}}}_{ij}^{\mathrm{nlos}/\mathrm{los}}\left(x,x^{\prime },x_{5},x_{5}^{\prime },\omega \right)\cdot {\bar{\bar{R}}}_{{\bar{\psi }}_{\mathrm{in}}}\left(x_{5},x_{5}^{\prime },\omega \right), \end{array}$$

where

(A131) $$\begin{array}{cc} {\bar{\bar{K}}}_{ij}^{\mathrm{nlos}/\mathrm{los}}\left(x,x^{\prime },x_{5},x_{5}^{\prime },\omega \right):= & \int_{S_{r}}d^{2}x_{1}\int_{S_{s}}d^{2}x_{2}\int_{S_{s}}d^{2}x_{3}\int_{S_{t}}d^{2}x_{4}\int_{S_{r}}d^{2}x_{1}^{\prime }\int_{S_{t}}d^{2}x_{4}^{\prime }\\ \; & \times E\left\{{\bar{S}}_{\mathrm{los}}^{†}\left(x^{\prime },x_{1}^{\prime },x_{4}^{\prime },x_{5}^{\prime },\omega ,\varpi \right)\cdot {\bar{a}}_{j}\;{\bar{a}}_{i}^{†}\cdot {\bar{\bar{S}}}_{\mathrm{nlos}}\left(x,x_{1},x_{2},x_{3},x_{4},x_{5},\omega ,\varpi \right)\right\}. \end{array}$$

Substituting (A124), (A126), (A128), and (A130) into (A107), making the changes $x_{5}\to x^{\prime \prime }$ and $x_{5}^{\prime }\to x^{\prime \prime \prime }$, we obtain (68) where

(A132) $$\begin{array}{c} {\bar{\bar{K}}}_{ij}\left(x,x^{\prime },x^{\prime \prime },x^{\prime \prime \prime },\omega \right)={\bar{\bar{K}}}_{ij}^{\mathrm{los}}\left(x,x^{\prime },x^{\prime \prime },x^{\prime \prime \prime },\omega \right)+{\bar{\bar{K}}}_{ij}^{\mathrm{nlos}}\left(x,x^{\prime },x^{\prime \prime },x^{\prime \prime \prime },\omega \right)+{\bar{\bar{K}}}_{ij}^{\mathrm{los}/\mathrm{nlos}}\left(x,x^{\prime },x^{\prime \prime },x^{\prime \prime \prime },\omega \right)+{\bar{\bar{K}}}_{ij}^{\mathrm{nlos}/\mathrm{los}}\left(x,x^{\prime },x^{\prime \prime },x^{\prime \prime \prime },\omega \right), \end{array}$$

This completes the proof of Theorem 5.

## Appendix P. Proof of Theorem 7

As per (25), the total received signal at the *m*th port must be decomposed into LoS and NLoS components denoted by $I_{m}^{\mathrm{los}}$ and $I_{m}^{\mathrm{nlos}}$, respectively. Therefore, the MIMO channel matrix element should be expanded as given in (84) with

(A133) $$H_{mn}^{\mathrm{los}}\left(\omega ,\varpi \right)={\left.\frac{I_{m}^{\mathrm{los}}\left(\omega ,\varpi \right)}{b_{\mathrm{in},n}\left(\omega ,\varpi \right)}\right|}_{b_{\mathrm{in},l}=0,l\neq n},\;\;\;H_{mn}^{\mathrm{nlos}}\left(\omega ,\varpi \right)={\left.\frac{I_{m}^{\mathrm{nlos}}\left(\omega ,\varpi \right)}{b_{\mathrm{in},n}\left(\omega ,\varpi \right)}\right|}_{b_{\mathrm{in},l}=0,l\neq n}.$$

We start with the LoS analysis first. Applying (76) and (78) in conjunction with (57) yields

(A134) $$\begin{array}{cc} I_{m}^{\mathrm{los}}\left(\omega ,\varpi \right)={\bar{b}}_{\mathrm{out},m}^{†}\cdot {\bar{J}}_{r}^{\mathrm{los}}\left(x_{m},\omega ,\varpi \right) & =\sum_{n=1}^{N_{t}}\int_{S_{r}}d^{2}x_{3}\int_{S_{t}}d^{2}x_{4}\;{\bar{b}}_{\mathrm{out},m}^{†}\cdot {\bar{\bar{S}}}_{\mathrm{los}}\left(x_{m},x_{3},x_{4},x_{n},\omega ,\varpi \right)\cdot {\bar{b}}_{\mathrm{in},n}b_{\mathrm{in},n}\left(\omega ,\varpi \right). \end{array}$$

Using (83) in (A134), we find

(A135) $$\begin{array}{c} H_{mn}^{\mathrm{los}}\left(\omega ,\varpi \right)=\int_{S_{r}}d^{2}x_{3}\int_{S_{t}}d^{2}x_{4}\;{\bar{b}}_{\mathrm{out},m}^{†}\cdot {\bar{\bar{S}}}_{\mathrm{los}}\left(x_{m},x_{3},x_{4},x_{n},\omega ,\varpi \right)\cdot {\bar{b}}_{\mathrm{in},n}. \end{array}$$

Substituting (55) into (A135), we arrive at

(A136) $$\begin{array}{c} H_{mn}^{\mathrm{los}}\left(\omega ,\varpi \right)=\int_{S_{r}}d^{2}x_{3}\int_{S_{t}}d^{2}x_{4}\;{\bar{b}}_{\mathrm{out},m}^{†}\cdot {\bar{\bar{F}}}_{r}\left(x_{m},x_{1},\omega ,\varpi \right)\cdot \bar{\bar{G}}\left(x_{3},x_{4},\omega ,\varpi \right)\cdot {\bar{\bar{F}}}_{t}\left(x_{4},x_{n},\omega ,\varpi \right)\cdot {\bar{b}}_{\mathrm{in},n}. \end{array}$$

Similarly, for the NLoS component, we employ (76) and (78) in conjunction with (58) to derive

(A137) $$\begin{array}{cc} I_{m}^{\mathrm{nlos}}\left(\omega ,\varpi \right)={\bar{b}}_{\mathrm{out},m}^{†}\cdot {\bar{J}}_{r}^{\mathrm{nlos}}\left(x_{m},\omega ,\varpi \right) & =\sum_{n=1}^{N_{t}}\int_{S_{r}}d^{2}x_{1}\int_{S_{s}}d^{2}x_{2}\int_{S_{s}}d^{2}x_{3}\int_{S_{t}}d^{2}x_{4}\\ \; & \times {\bar{b}}_{\mathrm{out},m}^{†}\cdot {\bar{\bar{S}}}_{\mathrm{nlos}}\left(x_{m},x_{1},x_{2},x_{3},x_{4},x_{n},\omega ,\varpi \right)\cdot {\bar{b}}_{\mathrm{in},n}b_{\mathrm{in},n}\left(\omega ,\varpi \right). \end{array}$$

Using (83) in (A137) gives

(A138) $$\begin{array}{c} H_{mn}^{\mathrm{nlos}}\left(\omega ,\varpi \right)=\int_{S_{r}}d^{2}x_{1}\int_{S_{s}}d^{2}x_{2}\int_{S_{s}}d^{2}x_{3}\int_{S_{t}}d^{2}x_{4}\;{\bar{b}}_{\mathrm{out},m}^{†}\cdot {\bar{\bar{S}}}_{\mathrm{nlos}}\left(x_{m},x_{1},x_{2},x_{3},x_{4},x_{n},\omega ,\varpi \right)\cdot {\bar{b}}_{\mathrm{in},n}. \end{array}$$

Substituting (56) into (A138), we obtain

(A139) $$\begin{array}{cc} H_{mn}^{\mathrm{nlos}}\left(\omega ,\varpi \right)= & \int_{S_{r}}d^{2}x_{1}\int_{S_{s}}d^{2}x_{2}\int_{S_{s}}d^{2}x_{3}\int_{S_{t}}d^{2}x_{4}\\ \; & \times {\bar{b}}_{\mathrm{out},m}^{†}\cdot {\bar{\bar{F}}}_{r}\left(x_{m},x_{1},\omega ,\varpi \right)\cdot \bar{\bar{G}}\left(x_{1},x_{2},\omega ,\varpi \right)\cdot {\bar{\bar{F}}}_{s}\left(x_{2},x_{3},\omega ,\varpi \right)\cdot \;\bar{\bar{G}}\left(x_{3},x_{4},\omega ,\varpi \right)\cdot {\bar{\bar{F}}}_{t}\left(x_{4},x_{n},\omega ,\varpi \right)\cdot {\bar{b}}_{\mathrm{in},n}. \end{array}$$

Substituting (A136) and (A139) into (84), Theorem 7 is proved.

## Appendix Q. Background on the Probability Distribution of Sums of Products of Independent Gaussian Random Variables

Compared to sums of independent random variables, the understanding of their products remains relatively underdeveloped. In various applications, such as channel modeling in wireless communications [146,147], understanding the statistical properties of the product of random variables is crucial. However, to the best of our knowledge, deriving the pdf of an arbitrary polynomial of several independent real Gaussian RVs with complex weights analytically remains an open problem. Even the simpler task of determining the pdf of an arbitrary monomial of RVs continues to be an area of active research, with significant progress made only in recent years.

The foundational work in this domain appears to have been initiated by Craig in 1936, who derived the characteristic function for the product of two Gaussian RVs [148]. While this function is expressed in a compact analytical form, deriving the pdf requires a Fourier integral evaluation, which has not been fully generalized despite recent advancements. Springer and Thompson addressed the problem of finding the pdf of the product of *n* identically distributed independent normal variables in 1966 [149]. By employing the Mellin transform and its inversion, they expressed the resulting distributions in terms of the Meijer G-function, a special function of mathematical physics. This technique was further extended to derive cdfs for products of *N* Gaussian RVs and to develop power-log series expansions using the more general Fox H-functions [150]. The distribution of the product of two correlated real Gaussian RVs with nonzero means and arbitrary variances was investigated in [151]. The authors obtained the exact pdf as an infinite series of modified Bessel functions of the second kind. Nadarajah and Pogány provided a definitive analytical solution for the pdf of the product of two correlated Gaussian RVs, expressing it in terms of Whittaker’s W-function and the modified Bessel function of the second kind [152]. Research has also extended to non-Gaussian RVs. For instance, the characteristic functions of the product of two Gaussian RVs, or when one is Gaussian and the other is a gamma RV, were explored in [153]. Similarly, ref. [154] derived the pdf of products of *n* Rayleigh RVs. However, results on arbitrarily weighted sums of products of RVs are much scarcer. The distribution of sums of identical products of two Gaussian RVs was addressed in [152], but to the best of our knowledge, analytical results for general (non-identical) monomials of independent RVs remain unavailable. For a recent overview of these developments, see [155].

## Appendix R. Entropy and Mutual Information for Random Fields

The construction of the pdf for any generic complex-valued EM RF *g* is fundamentally based on Definition A8 and the real vector representation outlined in Appendix H. Throughout the following, we assume the existence of a pdf for any given second-order EM RF g∈V, where V is defined in Table 2.

**Definition** **A12** (*n*-Point differential entropy of EM RFs)**.** 
*Let g∈V represent a generic frequency-domain EM RF. Assume that g(x) is defined for x∈D, with $D\subset R^{3}$ as the spatial domain, which is assumed to be topologically connected. Introduce n positions denoted by $x_{i}\in D$, where i∈{1,…,n}, such that for any i,j∈{1,…,n}, i≠j implies $x_{i}\neq x_{j}$. Define the augmented 3n×1 vector $\bar{x}$ as*

(A140)
$$\bar{x}:={\left[\begin{array}{cccc} x_{1} & x_{2} & \ldots & x_{n} \end{array}\right]}^{⊤}.$$

*We construct the real vector representation RF $g^{\left(r\right)}$, where $g^{\left(r\right)}$ has dimension q×1. Next, we define the nq×1 real random vector:*

(A141)
$$\bar{Y}\left(\bar{x}\right):={\left[\begin{array}{cccc} g^{\left(r\right)}\left(x_{1}\right) & g^{\left(r\right)}\left(x_{2}\right) & \cdots & g^{\left(r\right)}\left(x_{n}\right) \end{array}\right]}^{⊤}.$$

*The n-point differential entropy of the RF g over D at positions $\bar{x}$, denoted $H_{g}^{n}\left(\bar{x};D\right)$, is defined by:*

(A142)
$$H_{g}^{n}\left(\bar{x};D\right):=-\prod_{i=1}^{n}\int_{R}d^{q}x_{i}\;f_{\bar{Y}\left(\bar{x}\right)}\left(\bar{x}\right)\log f_{\bar{Y}\left(\bar{x}\right)}\left(\bar{x}\right),$$

*where*

(A143)
$$f_{\bar{Y}\left(\bar{x}\right)}\left(\bar{x}\right):=f_{Y^{1}\left(x_{1}\right)\ldots Y^{q}\left(x_{1}\right)Y^{1}\left(x_{2}\right)\ldots Y^{q}\left(x_{2}\right)\ldots Y^{1}\left(x_{n}\right)\ldots Y^{q}\left(x_{n}\right)}\left(\bar{x}\right)$$

*is the joint pdf of the nq random variables listed in vector *(A141)*. The averaged n-point entropy, denoted by $H_{g}^{n}\left(D\right)$, is computed with the help of the following formula:*

(A144)
$$H_{g}^{n}\left(D\right):=\frac{1}{\left|D\right|}\prod_{i=1}^{n}\int_{D}d^{q}x_{i}H_{g}^{n}\left(\bar{x};D\right),$$

*where |D| is the volume measure of the domain D.*

**Definition** **A13** (The (n,m)-point joint entropy of two EM RFs)**.** 
*Let $g_{1},g_{2}\in V$ represent two generic frequency-domain EM RFs. Assume that $g_{1}\left(x\right)$ and $g_{2}\left(x\right)$ are defined for $x\in D_{1}$ and $x\in D_{1}$, respectively with $D_{1,2}\subset R^{3}$ as two nonoverlapping spatial domain, where each is assumed to be topologically connected. Introduce n and m positions denoted by $x_{i}\in D_{1}$, where i∈{1,…,n}, and $y_{j}\in D_{2}$, where i∈{1,…,m}, such that all the points (in the two groups) are distinct from each other. Define the two augmented 3n×1 vector $\bar{x}$ as*

(A145)
$$\bar{x}:={\left[\begin{array}{cccc} x_{1} & x_{2} & \ldots & x_{n} \end{array}\right]}^{⊤},\;\bar{y}:={\left[\begin{array}{cccc} y_{1} & y_{2} & \ldots & y_{n} \end{array}\right]}^{⊤}.$$

*We construct two real vector representation RFs, $g_{1}^{\left(r\right)}$ and $g_{2}^{\left(r\right)}$, which have dimensions $q_{1}\times 1$ and $q_{2}\times 1$, respectively. Next, we define the $nq_{1}\times 1$ real random vector ${\bar{Y}}_{1}\left(\bar{x}\right)$ and another $mq_{2}\times 1$ real random vector ${\bar{Y}}_{2}\left(\bar{x}\right)$ as follows:*

(A146)
$$\begin{array}{cc} \; & {\bar{Y}}_{1}\left(\bar{x}\right):={\left[\begin{array}{cccc} g_{1}^{\left(r\right)}\left(x_{1}\right) & g_{1}^{\left(r\right)}\left(x_{2}\right) & \cdots & g_{1}^{\left(r\right)}\left(x_{n}\right) \end{array}\right]}^{⊤},\\ \; & {\bar{Y}}_{2}\left(\bar{x}\right):={\left[\begin{array}{cccc} g_{2}^{\left(r\right)}\left(y_{1}\right) & g_{2}^{\left(r\right)}\left(y_{2}\right) & \cdots & g_{2}^{\left(r\right)}\left(y_{n}\right) \end{array}\right]}^{⊤}. \end{array}$$

*We then form a third 2(n+m)×1 real vector*

(A147)
$$\bar{Z}\left(\bar{x},\bar{y}\right):={\left[\begin{array}{cc} {\bar{Y}}_{1}\left(\bar{x}\right) & {\bar{Y}}_{2}\left(\bar{y}\right) \end{array}\right]}^{⊤}.$$

*The (n,m)-point joint differential entropy of the RFs $g_{1}$ and $g_{2}$ over their respective domains $D_{1}$ and $D_{2}$, relative to positions $\bar{x}$ and $\bar{y}$, denoted $H_{g_{1}g_{2}}^{n}\left(\bar{x},\bar{y};D_{1},D_{2}\right)$, is defined by:*

(A148)
$$\begin{array}{cc} \; & H_{g_{1}g_{2}}^{n,m}\left(\bar{x},\bar{y};D_{1},D_{2}\right):=-\prod_{i=1}^{n}\int_{R}d^{q}x_{i}\prod_{j=1}^{m}\int_{R}d^{q}y_{j}\;f_{Z(\bar{x},\bar{y})}\left(\bar{x},\bar{y}\right)\log f_{\bar{Z}\left(\bar{x},\bar{y}\right)}\left(\bar{x},\bar{y}\right), \end{array}$$

*where*

(A149)
$$f_{\bar{Z}\left(\bar{x},\bar{y}\right)}\left(\bar{x},\bar{y}\right):=f_{{\bar{Y}}_{1}\left(\bar{x}\right){\bar{Y}}_{1}\left(\bar{y}\right)}\left(\bar{x},\bar{y}\right)$$

*is the joint pdf of the (n+m)q random variables listed in vector *(A147)* and the definition *(A143)* was utilized. The averaged (n,m)-point joint entropy, denoted by $H_{g_{1}g_{2}}^{n,m}\left(D_{1},D_{2}\right)$, can be computed using the following formula:*

(A150)
$$\begin{array}{cc} \; & H_{g_{1}g_{2}}^{n,m}\left(D_{1},D_{2}\right):=\frac{1}{|D_{1}\left|\right|D_{2}|}\prod_{i=1}^{n}\int_{D_{1}}d^{q}x_{i}\prod_{j=1}^{m}\int_{D_{2}}d^{q}y_{j}H_{g_{1}g_{2}}^{n}\left(\bar{x},\bar{y};D_{1},D_{2}\right), \end{array}$$

*where $|D_{1,2}|$ are the volume measures of the domains $D_{1,2}$.*

**Definition** **A14** (Mutual information between two EM RFs)**.** 
*Consider the two-EM-RF configuration described in Definition A13. The (n,m)-point mutual information is defined by*

(A151)
$$\begin{array}{cc} \; & I_{g_{1}g_{2}}^{n,m}\left(\bar{x}:\bar{y};D_{1}:D_{2}\right):=H_{g_{1}}^{n}\left(\bar{x};D_{1}\right)+H_{g_{2}}^{m}\left(\bar{y};D_{2}\right)-H_{g_{1}g_{2}}^{n,m}\left(\bar{x},\bar{y};D_{1},D_{2}\right). \end{array}$$

*The averaged mutual information is then defined by*

(A152)
$$\begin{array}{c} I_{g_{1}g_{2}}^{n,m}\left(D_{1}:D_{2}\right):=H_{g_{1}}^{n}\left(D_{1}\right)+H_{g_{2}}^{m}\left(D_{2}\right)-H_{g_{1}g_{2}}^{n,m}\left(D_{1},D_{2}\right). \end{array}$$

*Here, the quantities $H_{g_{1}}^{n}\left(D_{1}\right)$, $H_{g_{2}}^{m}\left(D_{2}\right)$, and $H_{g_{1}g_{2}}^{n,m}\left(D_{1},D_{2}\right)$ are computed according to the formulas provided in Definitions A12 and A13.*

## Appendix S. Synthetic Example: Non-Gaussian MIMO Channel from Gaussian Green’s Functions

We construct a minimal synthetic example that explicitly reflects the structure of Proposition 6 and Corollary 4. The example respects the physical KL decomposition of the LoS (4-factor) and NLoS (6-factor) response functions as derived in Equations (128) and (124), and demonstrates that even with Gaussian underlying random variables, the resulting channel is non-Gaussian.

Consider a point-port MIMO system where the received scalar channel *H* is given by

(A153) $$H=H^{\mathrm{los}}+H^{\mathrm{nlos}},$$

with each component modeled via truncated KL expansions consistent with the effective random variables defined in Proposition 3. We have two tyoes of terms:

1. *LoS component (2-mode truncation, 4-factor monomials):* Let the LoS contribution involve two KL terms, each a product of four mutually independent Gaussian RVs:
  (A154) $$H^{\mathrm{los}}=c_{1}\;X_{s_{1}}^{r}X_{p_{1}}^{g_{t}}X_{m_{1}}^{t}X_{n_{1}}^{\mathrm{in}}+c_{2}\;X_{s_{2}}^{r}X_{p_{2}}^{g_{t}}X_{m_{2}}^{t}X_{n_{2}}^{\mathrm{in}},$$
  where all *X*’s are i.i.d. N(0,1), and $c_{1},c_{2}\in C$ are deterministic coupling constants.
2. *NLoS component (3-mode truncation, 6-factor monomials):* Similarly, let the NLoS contribution involve three KL terms, each a product of six mutually independent Gaussian RVs:
  (A155) $$H^{\mathrm{nlos}}=d_{1}\;X_{s_{1}^{\prime }}^{r}X_{r_{1}^{\prime }}^{g_{s}}X_{q_{1}^{\prime }}^{s}X_{p_{1}^{\prime }}^{g_{t}}X_{m_{1}^{\prime }}^{t}X_{n_{1}^{\prime }}^{\mathrm{in}}+d_{2}\;X_{s_{2}^{\prime }}^{r}X_{r_{2}^{\prime }}^{g_{s}}X_{q_{2}^{\prime }}^{s}X_{p_{2}^{\prime }}^{g_{t}}X_{m_{2}^{\prime }}^{t}X_{n_{2}^{\prime }}^{\mathrm{in}}+d_{3}\;X_{s_{3}^{\prime }}^{r}X_{r_{3}^{\prime }}^{g_{s}}X_{q_{3}^{\prime }}^{s}X_{p_{3}^{\prime }}^{g_{t}}X_{m_{3}^{\prime }}^{t}X_{n_{3}^{\prime }}^{\mathrm{in}},$$
  with all *X*’s again i.i.d. N(0,1), independent across all indices and from those in $H^{\mathrm{los}}$, and $d_{1},d_{2},d_{3}\in C$ deterministic.

By construction, every constituent random variable is Gaussian, and all Green’s functions are modeled as Gaussian random fields (since their KL coefficients are Gaussian and independent). However, each term in *H* is a monomial of degree 4 or 6 in independent Gaussian variables. To evaluate the statistical moments, we use the fact that for independent zero-mean unit-variance Gaussian variables $X_{i}∼N\left(0,1\right)$, the expectation of a product factorizes over disjoint subsets, and even-order moments follow Wick’s theorem:

$$E\left[X_{i_{1}}\cdots X_{i_{2k}}\right]=\sum_{\mathrm{pairings}}\prod E\left[X_{i_{a}}X_{i_{b}}\right],$$

while odd-order moments vanish. For a product of two such variables, $Y=X_{1}X_{2}$, we have $E\left[Y^{2}\right]=E\left[X_{1}^{2}\right]E\left[X_{2}^{2}\right]=1$, and $E\left[Y^{4}\right]=E\left[X_{1}^{4}\right]E\left[X_{2}^{4}\right]=3\cdot 3=9$, since $E[X_{i}^{4}]=3$ for $X_{i}∼N\left(0,1\right)$. In contrast, a zero-mean complex circular Gaussian variable *Z* satisfies ${E[|Z|}^{4}{]=2(E[|Z|}^{2}{])}^{2}$. For a real-valued Gaussian *Y*, the analogous identity would be $E\left[Y^{4}\right]=3{\left(E\left[Y^{2}\right]\right)}^{2}=3$, which is violated here because $Y=X_{1}X_{2}$ is not Gaussian. Thus, $E\left[{\left(X_{1}X_{2}\right)}^{4}\right]=9\neq 3{\left(E[{\left(X_{1}X_{2}\right)}^{2}]\right)}^{2}=3$, violating the fourth-moment identity of Gaussian variables. Consequently, *H*—as a finite sum of such non-Gaussian monomials—is itself non-Gaussian.

## References

[1] Gallager R.G. (1968). Information Theory and Reliable Communication.

[2] Cover T.M., Thomas J.A. (2006). Elements of Information Theory.

[3] Franceschetti M., Marano S., Palmieri F. (2003). The role of entropy in wave propagation. Proceedings of the IEEE International Symposium on Information Theory Proceedings, Yokohama, Japan, 29 June–4 July 2003.

[4] Mikki S., Antar Y. Unifying electromagnetic and communication theories: A proposal for a new research program. Proceedings of the 2016 URSI International Symposium on Electromagnetic Theory (EMTS).

[5] Nielsen M., Chuang I.L. (2010). Quantum Computation and Quantum Information.

[6] Wilde M. (2017). Quantum Information Theory.

[7] Nakano T. (2013). Molecular Communication.

[8] Franceschetti M. (2012). Wave Theory of Information.

[9] Brillouin L. (2013). Science and Information Theory.

[10] Gabor D. (1946). Theory of communication. Part 1: The analysis of information. J. Inst. Electr. Eng.-III Radio Commun. Eng..

[11] Zhu J., Wan Z., Dai L., Debbah M., Poor H.V. (2024). Electromagnetic Information Theory: Fundamentals, Modeling, Applications, and Open Problems. IEEE Wirel. Commun..

[12] Migliore M.D. (2008). On Electromagnetics and Information Theory. IEEE Trans. Antennas Propag..

[13] Gruber F.K., Marengo E.A. (2008). New Aspects of Electromagnetic Information Theory for Wireless and Antenna Systems. IEEE Trans. Antennas Propag..

[14] Martini A., Massa A., Franceschetti M. (2009). Physical limits to the capacity of wide-band Gaussian MIMO channels. IEEE Trans. Wirel. Commun..

[15] Migliore M.D. (2020). Who Cares About the Horse? A Gentle Introduction to Information in Electromagnetic Theory. IEEE Antennas Propag. Mag..

[16] Franceschetti M., Migliore M.D., Minero P. (2009). The Capacity of Wireless Networks: Information-Theoretic and Physical Limits. IEEE Trans. Inf. Theory.

[17] Renzo M.D., Migliore M.D. (2024). Electromagnetic Signal and Information Theory. IEEE BITS Inf. Theory Mag..

[18] Franceschetti M. Wave diversity limits for wireless communication. Proceedings of the 2008 IEEE Antennas and Propagation Society International Symposium.

[19] Mikki S. (2023). The Shannon Information Capacity of an Arbitrary Radiating Surface: An Electromagnetic Approach. IEEE Trans. Antennas Propag..

[20] Lu S., Hui H.T., Bialkowski M. (2008). Optimizing MIMO Channel Capacities Under the Influence of Antenna Mutual Coupling. IEEE Antennas Wirel. Propag. Lett..

[21] Chiurtu N., Rimoldi B. (2000). Varying the antenna locations to optimize the capacity of multi-antenna Gaussian channels. Proceedings of the 2000 IEEE International Conference on Acoustics, Speech, and Signal Processing, Istanbul, Turkey, 5–9 June 2000.

[22] Yoo I., Imani M.F., Sleasman T., Pfister H.D., Smith D.R. (2019). Enhancing Capacity of Spatial Multiplexing Systems Using Reconfigurable Cavity-Backed Metasurface Antennas in Clustered MIMO Channels. IEEE Trans. Commun..

[23] Mikki S., Hanoon A., Persano J., Alzahed A., Antar Y., Aulin J. Theory of electromagnetic intelligent agents with applications to MIMO and DoA systems. Proceedings of the 2017 IEEE International Symposium on Antennas and Propagation USNC/URSI National Radio Science Meeting.

[24] Alzahed A., Mikki S., Antar Y. (2019). Nonlinear Mutual Coupling Compensation Operator Design Using a Novel Electromagnetic Machine Learning Paradigm. IEEE Antennas Wirel. Propag. Lett..

[25] Li R., Li D., Ma J., Feng Z., Zhang L., Tan S., Sha W.E.I., Chen H., Li E.P. (2023). An Electromagnetic Information Theory Based Model for Efficient Characterization of MIMO Systems in Complex Space. IEEE Trans. Antennas Propag..

[26] Liu D., Shi H., Yang T., Qiao Z. (2024). Enhanced Resolution Method for Electromagnetic Vortex Imaging Based on Electromagnetic Information Theory. IEEE Trans. Geosci. Remote Sens..

[27] Hayashi Y.I., Homma N., Mizuki T., Aoki T., Sone H., Sauvage L., Danger J.L. (2013). Analysis of Electromagnetic Information Leakage From Cryptographic Devices with Different Physical Structures. IEEE Trans. Electromagn. Compat..

[28] El Sayed W., Crovetti P., Moonen N., Lezynski P., Smolenski R., Leferink F. (2021). Electromagnetic Interference of Spread-Spectrum Modulated Power Converters in G3-PLC Power Line Communication Systems. IEEE Lett. Electromagn. Compat. Pract. Appl..

[29] Bai X., Tan S., Mikki S., Li E., Cui T.J. (2024). Information-theoretic Measures for Reconfigurable Metasurface-enabled Direct Digital Modulation Systems: An Electromagnetic Perspective. Prog. Electromagn. Res..

[30] Bai X., Tan S., Li R. Capacity Characterization for Binary RIS Aided MIMO Communication System Based on An Electromagnetic Scattering Model. Proceedings of the 2023 International Applied Computational Electromagnetics Society Symposium (ACES-China).

[31] You J., Ma Q., Zhang L., Liu C., Zhang J., Liu S., Cui T. (2023). Electromagnetic Metamaterials: From Classical to Quantum. Electromagn. Sci..

[32] Bai X., Li R., Tan S., Mikki S., Li E. (2024). An Electromagnetic Analysis of the Impact of Random Scattering and RISs on the Shannon Capacity of MIMO Communication Systems. IEEE Antennas Wirel. Propag. Lett..

[33] Adler R.J. (2010). The Geometry of Random Fields.

[34] Holevo A., Giovannetti V. (2012). Quantum channels and their entropic characteristics. Rep. Prog. Phys..

[35] Pizzo A., Torres A.d.J., Sanguinetti L., Marzetta T.L. (2022). Nyquist Sampling and Degrees of Freedom of Electromagnetic Fields. IEEE Trans. Signal Process..

[36] Pizzo A., Sanguinetti L., Marzetta T.L. (2022). Spatial Characterization of Electromagnetic Random Channels. IEEE Open J. Commun. Soc..

[37] Enßlin T.A. (2019). Information Theory for Fields. Ann. Der Phys..

[38] Wan Z., Zhu J., Zhang Z., Dai L., Chae C.B. (2023). Mutual Information for Electromagnetic Information Theory Based on Random Fields. IEEE Trans. Commun..

[39] Mikki S. (2019). The Antenna Spacetime System Theory of Wireless Communications. Proc. R. Soc. A Math. Phys. Eng. Sci..

[40] Mikki S., Antar Y. (2012). On the Fundamental Relationship Between the Transmitting and Receiving Modes of General Antenna Systems: A New Approach. IEEE Antennas Wirel. Propag. Lett..

[41] Mikki S., Antar Y. (2013). The antenna current Green’s function formalism–Part I. IEEE Trans. Antennas Propagat.

[42] Mikki S., Antar Y. (2013). The antenna current Green’s function formalism—Part II. IEEE Trans. Antennas Propagat.

[43] Christakos G. (2017). Space, time, space–time, randomness, and probability. Spatiotemporal Random Fields.

[44] Felsen L. (1994). Radiation and Scattering of Waves.

[45] Mikki S., Antar Y. (2016). New Foundations for Applied Electromagnetics: The Spatial Structure of Fields.

[46] Dong Z., Zeng Y. (2022). Near-Field Spatial Correlation for Extremely Large-Scale Array Communications. IEEE Commun. Lett..

[47] Mikki S., Clauzier S., Antar Y. (2019). A Correlation Theory of Antenna Directivity With Applications to Superdirective Arrays. IEEE Antennas Wirel. Propag. Lett..

[48] Zhang H., Shlezinger N., Guidi F., Dardari D., Eldar Y.C. (2023). 6G Wireless Communications: From Far-Field Beam Steering to Near-Field Beam Focusing. IEEE Commun. Mag..

[49] Cui M., Wu Z., Lu Y., Wei X., Dai L. (2023). Near-Field MIMO Communications for 6G: Fundamentals, Challenges, Potentials, and Future Directions. IEEE Commun. Mag..

[50] Mikki S., Antar Y. (2011). A Theory of Antenna Electromagnetic Near Field–Part I. IEEE Trans. Antennas Propag..

[51] Mikki S., Antar Y.M.M. (2011). A Theory of Antenna Electromagnetic Near Field—Part II. IEEE Trans. Antennas Propag..

[52] Mikki S., Sarkar D., Antar Y. (2019). Near-Field Cross-Correlation Analysis for MIMO Wireless Communications. IEEE Antennas Wirel. Propag. Lett..

[53] Loeve M. (1978). Probability Theory II.

[54] Hernández D.B. (1995). Lectures on Probability and Second Order Random Fields.

[55] Wald R. (2022). Advanced Classical Electromagnetism.

[56] Schwinger J., DeRaad L.L., Milton K., Tsai W.Y. (1998). Classical Electrodynamics.

[57] Jackson J. (1999). Classical Electrodynamics.

[58] Jakes W.C. (1994). Microwave Mobile Communications (An IEEE Press Classic Reissue).

[59] Mikki S., Antar Y. (2015). On Cross Correlation in Antenna Arrays with Applications to Spatial Diversity and MIMO Systems. IEEE Trans. Antennas Propag..

[60] Mikki S., Antar Y. (2015). Aspects of Generalized Electromagnetic Energy Exchange in Antenna Systems: A New Approach to Mutual Coupling. Proceedings of the 2015 9th European Conference on Antennas and Propagation (EuCAP), Lisbon, Portugal, 12–17 April 2015.

[61] Sarkar D., Srivastava K.V. MIMO Antenna Characterization in Realistic Propagation Scenario: Use of Infinitesimal Dipole Models with Cross-correlation Green’s Functions. Proceedings of the 2018 IEEE Indian Conference on Antennas and Propogation (InCAP).

[62] Sarkar D., Mikki S., Antar Y. Fast and Efficient Estimation of Spatial Correlation Characteristics of Co-Located Dual-polarized Massive MIMO Arrays in 5G Base Stations. Proceedings of the TEQIP III Sponsored International conference on Microwave Integrated Circuits, Photonics and Wireless Networks—2019, (IMICPW-2019).

[63] Mikki S., Aulin J. The stochastic electromagnetic theory of antenna-antenna cross-correlation in MIMO systems. Proceedings of the 12th European Conference on Antennas and Propagation (EuCAP 2018).

[64] Sadiku M.N.O. (2000). Numerical Techniques in Electromagnetics.

[65] Jin J. (2014). The Finite Element Method in Electromagnetics.

[66] Taflove A. (2005). Computational Electrodynamics: The Finite-Difference Time-Domain Method.

[67] Chew W., Tong M.S., Hu B. (2009). Integral Equation Methods for Electromagnetic and Elastic Waves.

[68] Gibson W.C. (2015). The Method of Moments in Electromagnetics.

[69] Harrington R.F. (2000). Field Computation by Moment Methods.

[70] Chew W., Jin J.M., Michielssen E., Song J. (2001). Fast and Efficient Algorithms in Computational Electromagnetics.

[71] Ghanem R.G., Spanos P.D. (2011). Stochastic Finite Elements: A Spectral Approach.

[72] Betz W., Papaioannou I., Straub D. (2014). Numerical methods for the discretization of random fields by means of the Karhunen–Loève expansion. Comput. Methods Appl. Mech. Eng..

[73] Stefanou G. (2009). The stochastic finite element method: Past, present and future. Comput. Methods Appl. Mech. Eng..

[74] Arregui-Mena J.D., Margetts L., Mummery P.M. (2016). Practical application of the stochastic finite element method. Arch. Comput. Methods Eng..

[75] Der Kiureghian A., Ke J.B. (1988). The stochastic finite element method in structural reliability. Probabilistic Eng. Mech..

[76] Frauenfelder P., Schwab C., Todor R.A. (2005). Finite elements for elliptic problems with stochastic coefficients. Comput. Methods Appl. Mech. Eng..

[77] Matthies H.G., Keese A. (2005). Galerkin methods for linear and nonlinear elliptic stochastic partial differential equations. Comput. Methods Appl. Mech. Eng..

[78] Shinozuka M., Yamazaki F. (2020). Stochastic finite element analysis: An introduction. Stochastic Structural Dynamics.

[79] Babuška I., Tempone R., Zouraris G.E. (2005). Solving elliptic boundary value problems with uncertain coefficients by the finite element method: The stochastic formulation. Comput. Methods Appl. Mech. Eng..

[80] Zeidler E. (2009). Quantum Field Theory I: Basics in Mathematics and Physics.

[81] Zeidler E. (2006). Quantum Field Theory II: Quantum Electrodynamics.

[82] Xu L., Mikki S. (2026). A Random Field-Theoretic Framework for Electromagnetic Information Computation. IEEE Trans. Antennas Propag..

[83] Klauder J., Sudarshan E.C.G. (2006). Fundamentals of Quantum Optics.

[84] Collin R. (2001). Foundations for Microwave Engineering.

[85] Taubock G. (2012). Complex-Valued Random Vectors and Channels: Entropy, Divergence, and Capacity. IEEE Trans. Inf. Theory.

[86] Lee J. (2012). Introduction to Smooth Manifolds.

[87] Appel W. (2007). Mathematics for Physics and Physicists.

[88] Mikki S. (2020). Generalized Current Green’s Function Formalism for Electromagnetic Radiation by Composite Systems. Prog. Electromagn. Res. B.

[89] Lee J.M. (2000). Introduction to Topological Manifolds.

[90] Kelley J. (2017). General Topology.

[91] Balanis C.A. (2024). Advanced Engineering Electromagnetics.

[92] Harrington R.F. (2001). Time-Harmonic Electromagnetic Fields.

[93] Bladel J. (2007). Electromagnetic Fields.

[94] Heath R., Lozano A. (2019). Foundations of MIMO Communication.

[95] Marzetta T.L., Larsson E.G., Yang H., Ngo H.Q. (2016). Fundamentals of Massive MIMO.

[96] Ishimaru A. (1997). Wave Propagation and Scattering in Random Media.

[97] Gonis A. (2000). Multiple Scattering in Solids.

[98] Hirsch M. (1976). Differential Topology.

[99] Guillemin V., Pollack A. (2010). Differential Topology.

[100] Burns K., Gidea M. (2005). Differential Geometry and Topology: With a View to Dynamical Systems.

[101] Dundas B.I. (2018). A Short Course in Differential Topology.

[102] Mukherjee A. (2015). Differential Topology.

[103] Lee J.M. (2019). Introduction to Riemannian Manifolds.

[104] Chew W.C. (1999). Waves and Fields in Inhomogenous Media.

[105] Geyi W. (2010). Foundations of Applied Electrodynamics.

[106] Schelkunoff S.A. (1943). A Mathematical Theory of Linear Arrays. Bell Syst. Tech. J..

[107] Schelkunoff S.A. (1984). Theory of antennas of arbitrary size and shape. Proc. IEEE.

[108] Schelkunoff S.A., Friss H.T. (1952). Antennas: Theory and Practice.

[109] Tai C.T. (1994). Dyadic Green Functions in Electromagnetic Theory.

[110] Kolundzija B.M., Djordjevic A.R. (2003). Electromagnetic Modeling of Composite Metallic and Dielectric Structures.

[111] Jones F. (2000). Lebesgue Integration on Euclidean Space.

[112] Lang S. (1993). Real and Functional Analysis.

[113] VanMarcke E. (2010). Random Fields: Analysis and Synthesis.

[114] Christakos G. (2005). Random Field Models in Earth Sciences.

[115] Adler R.J., Taylor J. (2007). Random Fields and Geometry.

[116] Taylor J.E. (2006). A Gaussian kinematic formula. Ann. Probab..

[117] Azaïs J.M., Wschebor M. (2005). On the distribution of the maximum of a Gaussian field with d parameters. Ann. Appl. Probab..

[118] Hristopulos D.T. (2020). Random Fields for Spatial Data Modeling.

[119] Schreier P.J., Scharf L.L. (2010). Statistical Signal Processing of Complex-Valued Data: The Theory of Improper and Noncircular Signals.

[120] Sarkar D., Srivastava K.V. (2018). Modified Cross Correlation Green’s Function With FDTD for Characterization of MIMO Antennas in Nonuniform Propagation Environment. IEEE Trans. Antennas Propag..

[121] Lathi B.P., Ding Z. (2019). Modern Digital and Analog Communication Systems.

[122] Telatar E. (1999). Capacity of Multi-antenna Gaussian Channels. Eur. Trans. Telecommun..

[123] Bourbaki N. (2004). Theory of Sets.

[124] Sheppard B. (2014). The Logic of Infinity.

[125] Mikki S. (2023). Near-Field Matching and Universal Limits on Electromagnetic Energy Transfer. Mathematics.

[126] Fontanella L., Ippoliti L., Subba Rao T., Subba Rao S., Rao C. (2012). Karhunen–Loéve Expansion of Temporal and Spatio-Temporal Processes. Time Series Analysis: Methods and Applications; Handbook of Statistics.

[127] Schwab C., Todor R.A. (2006). Karhunen–Loève approximation of random fields by generalized fast multipole methods. J. Comput. Phys..

[128] Perrin G., Soize C., Duhamel D., Funfschilling C. (2013). Karhunen–Loève expansion revisited for vector-valued random fields: Scaling, errors and optimal basis. J. Comput. Phys..

[129] De Iaco S., Posa D., Palma M. (2013). Complex-Valued Random Fields for Vectorial Data: Estimating and Modeling Aspects. Math. Geosci..

[130] Castrillon-Candas J., Kon M. (2022). Stochastic Functional Analysis and Multilevel Vector Field Anomaly Detection. arXiv.

[131] Loeve M. (1977). Probability Theory I.

[132] Gikhman I.I., Skorokhod A.V. (2004). The Theory of Stochastic Processes I.

[133] Gikhman I.I., Skorokhod A.V. (2004). The Theory of Stochastic Processes II.

[134] Gikhman I.I., Skorokhod A.V. (2007). The Theory of Stochastic Processes III.

[135] Henault S., Podilchak S.K., Mikki S., Antar Y. (2013). A Methodology for Mutual Coupling Estimation and Compensation in Antennas. IEEE Trans. Antennas Propag..

[136] Mikki S., Antar Y. (2015). A rigorous approach to mutual coupling in general antenna systems through perturbation theory. IEEE Antennas Wirel. Commun. Lett..

[137] Sarrazin F., Mikki S., Pouliguen P., Sharaiha A., Antar Y. (2016). An Upper Bound on Antenna Near-Field Deviations Caused by Mutual Coupling for Applications in Optimization Design Methods. IEEE Antennas Wirel. Propag. Lett..

[138] Kim Y.D., Kim H.J., Bae K.U., Park J.H., Myung N.H. (2015). A Hybrid UTD-ACGF Technique for DOA Finding of Receiving Antenna Array on Complex Environment. IEEE Trans. Antennas Propag..

[139] Kim Y.D., Yi D.W., Yang S.J., Chae H., Yu J.W., Myung N.H. (2017). Beam Pattern Analysis of Antenna Array on Complex Platform Using AEP Method Based on Hybrid UTD-ACGF Technique. IEEE Trans. Antennas Propag..

[140] Landau L.D., Lifshitz E., Pitaevskii L.P. (1984). Electrodynamics of Continuous Media.

[141] Neeser F.D., Massey J.L. (1993). Proper complex random processes with applications to information theory. IEEE Trans. Inf. Theory.

[142] Picinbono B. (1996). Second-order complex random vectors and normal distributions. IEEE Trans. Signal Process..

[143] Yoon Y.C., Leib H. (1997). Maximizing SNR in improper complex noise and applications to CDMA. IEEE Commun. Lett..

[144] Schreier P., Scharf L. (2003). Second-order analysis of improper complex random vectors and processes. IEEE Trans. Signal Process..

[145] Schreier P.J., Scharf L.L., Mullis C.T. (2005). Detection and estimation of improper complex random signals. IEEE Trans. Inf. Theory.

[146] Salo J., El-Sallabi H.M., Vainikainen P. (2006). Statistical Analysis of the Multiple Scattering Radio Channel. IEEE Trans. Antennas Propag..

[147] Karagiannidis G.K., Sagias N.C., Mathiopoulos P.T. (2007). N*Nakagami: A Novel Stochastic Model for Cascaded Fading Channels. IEEE Trans. Commun..

[148] Craig C.C. (1936). On the Frequency Function of *xy*. Ann. Math. Stat..

[149] Springer M.D., Thompson W.E. (1966). The Distribution of Products of Independent Random Variables. SIAM J. Appl. Math..

[150] Suess D., Kliesch M., Stojanac Z., Lu Y.M., Papadakis M., Van De Ville D. (2017). On the distribution of a product of N Gaussian random variables. Proceedings of the Wavelets and Sparsity XVII, San Diego, CA, USA, 6–9 August 2017.

[151] Cui G., Yu X., Iommelli S., Kong L. (2016). Exact Distribution for the Product of Two Correlated Gaussian Random Variables. IEEE Signal Process. Lett..

[152] Nadarajah S., Pogány T.K. (2015). On the distribution of the product of correlated normal random variables. Comptes Rendus. MathéMatique.

[153] Schoenecker S., Luginbuhl T. (2016). Characteristic Functions of the Product of Two Gaussian Random Variables and the Product of a Gaussian and a Gamma Random Variable. IEEE Signal Process. Lett..

[154] Salo J., El-Sallabi H., Vainikainen P. (2006). The distribution of the product of independent Rayleigh random variables. IEEE Trans. Antennas Propag..

[155] Gaunt R.E. (2022). The basic distributional theory for the product of zero mean correlated normal random variables. Stat. Neerl..

